# Xanthine–Dopamine Hybrid Molecules as Multitarget Drugs with Potential for the Treatment of Neurodegenerative Diseases

**DOI:** 10.3390/biom13071079

**Published:** 2023-07-05

**Authors:** Michał Załuski, Tadeusz Karcz, Anna Drabczyńska, Christin Vielmuth, Agnieszka Olejarz-Maciej, Monika Głuch-Lutwin, Barbara Mordyl, Agata Siwek, Grzegorz Satała, Christa E. Müller, Katarzyna Kieć-Kononowicz

**Affiliations:** 1 Department of Technology and Biotechnology of Drugs, Faculty of Pharmacy, Jagiellonian University Medical College, 30-688 Krakow, Poland; zaluski.michal@gmail.com (M.Z.); agnieszka.olejarz@uj.edu.pl (A.O.-M.); 2 PharmaCenter Bonn, Pharmaceutical Institute, Pharmaceutical & Medicinal Chemistry, University of Bonn, D-53121 Bonn, Germany; christin.vielmuth@uni-bonn.de (C.V.); christa.mueller@uni-bonn.de (C.E.M.); 3 Department of Pharmacobiology, Faculty of Pharmacy, Jagiellonian University Medical College, 30-688 Krakow, Poland; monika.gluch-lutwin@uj.edu.pl (M.G.-L.); barbara.mordyl@uj.edu.pl (B.M.); agat.siwek@uj.edu.pl (A.S.); 4 Department of Medicinal Chemistry, Maj Institute of Pharmacology, Polish Academy of Sciences, 31-343 Krakow, Poland; satala@if-pan.krakow.pl

**Keywords:** monoamine oxidase B inhibitors, adenosine A_2A_ receptor antagonists, PDE4/10 inhibitors, D_2_ dopamine receptor agonists

## Abstract

Multitarget drugs based on a hybrid dopamine–xanthine core were designed as potential drug candidates for the treatment of neurodegenerative diseases. Monoamine oxidase B (MAO-B) inhibitors with significant ancillary A_2A_ adenosine receptor (A_2A_AR) antagonistic properties were further developed to exhibit additional phosphodiesterase-4 and -10 (PDE4/10) inhibition and/or dopamine D_2_ receptor (D_2_R) agonistic activity. While all of the designed compounds showed MAO-B inhibition in the nanomolar range mostly combined with submicromolar A_2A_AR affinity, significant enhancement of PDE-inhibitory and D_2_R-agonistic activity was additionally reached for some compounds through various structural modifications. The final multitarget drugs also showed promising antioxidant properties in vitro. In order to evaluate their potential neuroprotective effect, representative ligands were tested in a cellular model of toxin-induced neurotoxicity. As a result, protective effects against oxidative stress in neuroblastoma cells were observed, confirming the utility of the applied strategy. Further evaluation of the newly developed multitarget ligands in preclinical models of Alzheimer’s and Parkinson’s diseases is warranted.

## 1. Introduction

Neurodegeneration is described as the progressive loss of structure and function of neurons leading to central nervous system dysfunction [1,2]. Although neurodegenerative diseases (NDs) are a clinically and pathologically heterogeneous group of disorders, including Parkinson’s disease (PD), Huntington’s disease (HD), and Alzheimer’s disease (AD), they share some general molecular mechanisms leading to a pathogenic cascade, which includes protein misfolding and aggregation, mitochondrial dysfunction, oxidative stress and free radical formation, and inflammation and deregulation of microRNAs [3,4,5,6,7,8].

The primary causes of neurodegeneration are still unclear, and a wide spectrum of potential biological targets is considered for the prevention of neurodegeneration and the treatment of neurodegenerative diseases. Therefore, a multitarget-drug approach, also described as polypharmacology, presents a strategy adopted by medicinal chemists to provide new molecules with increased therapeutic efficacy [9,10]. However, the prevalent management in various NDs is still based on a mono-target approach, and the primary drug targets are related to the modulation of neurotransmitter levels to compensate disturbances caused by the loss of neurons. Dopamine replacement therapy in PD, and acetylcholine esterase (AChE) inhibitors in AD, were milestones in the symptomatic therapy of these diseases; nevertheless, they are insufficient to prevent or stop the chronic progression of neurodegeneration [11,12].

From a medicinal chemistry perspective, multitarget drugs can interact with several targets, thereby improving therapeutic efficacy, attenuating side effects, and providing a more predictable pharmacokinetic profile than combination therapies [13,14]. Thus, the strategy to develop multitarget ligands is now intensively explored for ND, generating new molecules that simultaneously address various biological targets [10,15].

Monoamine oxidases (MAO) are enzymes involved in the metabolism of various amines, including neurotransmitters. There are two isoenzymes, MAO-A and MAO-B, that differ in substrate preference and tissue distribution. Elevated levels of MAO-B have been detected in the aging brain, resulting in increased MAO-B enzymatic activity around amyloid β-plaques in aged AD mice, reducing levels of neurotransmitters, in particular, dopamine [16,17]. Even worse, MAO-B may lead to an acceleration of the neurodegenerative process by generating potentially neurotoxic substances, such as dopaldehyde and hydrogen peroxide [18]. On the other hand, the neuroprotective effect of MAO-B inhibition may be also be associated with the regulation of the mitochondrial apoptosis system, maintenance of mitochondrial function, suppression of α-synuclein oligomerization and aggregation, increased expression of genes encoding antioxidant enzymes, and anti-apoptotic Bcl-2 and pro-survival neurotropic factors [19,20,21]. Those potential disease-modifying properties of MAO-B inhibitors were widely investigated to create new anti-ND agents designed as multitarget drugs, e.g., via combination of MAO-B inhibition and a blockade or activation of G-protein-coupled receptors or inhibition of AChE [22,23,24].

The blockade of adenosine A_2A_ receptors (A_2A_AR) enhances dopamine D_2_ receptor (D_2_R)-dependent signaling in the basal ganglia of the central nervous system. Therefore, the selective A_2A_AR antagonist istradefylline, which is based on an 8-styrylxanthine core, was approved in Japan (in 2013) and later on in the USA (in 2019) as an adjuvant therapeutic in combination with levodopa for the treatment of PD [25]. Besides the improvement in motor-related dysfunction in PD patients, A_2A_AR antagonists may exhibit neuroprotective effects by reducing oxidative stress, glutamate excitotoxity, and microglial reactivity [26,27,28]. Several in vivo models of neurodegenerative diseases indicated an improvement in cognitive impairment via a blockade of A_2A_AR. This effect was further enhanced by A_1_AR antagonism in dual A_1_/A_2A_AR blocking agents [29,30,31]. Also, in models of AD, A_2A_AR antagonists or a knockdown of A_2A_ARs showed neuroprotective effects [32,33]. Recently, the chronic administration of caffeine (1,3,7-trimethylxanthine), a non-selective AR antagonist, was found to increase information encoding and processing in the hippocampus of mice, indicating beneficial effects on memory and learning [34].

Phosphodiesterases (PDE) are a family of enzymes that degrade cyclic nucleotides via the hydrolysis of phosphodiester bonds. Eleven PDE family members (PDE1–PDE11) were identified, which show various substrate selectivities and biological profiles [35]. In the context of NDs, PDE4 and PDE10 seem to be particularly involved. PDE4 is a cAMP-specific enzyme and its selective inhibitors have been found to improve memory and cognition deficits in rodent AD models, associated with misfolded amyloid β protein. Moreover, PDE4 inhibitors may increase tyrosine hydroxylase phosphorylation, leading to an enhancement of dopamine biosynthesis and a protection of dopaminergic neurons [36,37,38]. PDE10 hydrolyzes both cAMP and cGMP, and high expression was detected in striatum. The dysregulation of this brain region may lead to motor dysfunction and cognition impairment. PDE10 inhibitors are intensively explored as drug candidates for HD, PD, and schizophrenia [39,40].

Dopamine D_2_ receptor agonists are applied as monotherapy or combined with L-DOPA, the precursor of dopamine, for the treatment of PD [41]. The enhancement of D_2_ neurotransmission in the nigrostratial pathway improves motor dysfunction. Moreover, recent studies showed potential neuroprotective and anti-inflammatory effects for D_2_R agonists [42,43]. The activation of D_2_R expressed on CD4^+^ T cells was suggested to protect against neuroinflammation and neurodegeneration in animal studies [44].

The object of the present study was the optimization of novel hybrid compounds targeting MAO-B and adenosine receptors as anti-neurodegenerative agents. The hybrid chemical scaffold shares two pharmacophore units: a xanthine part (including pyrimidine- or diazepine-containing tricyclic analogues) and a dopamine part. Chemical modifications focus on the *N*7-position of the xanthine core via the introduction of various substituents, such as alkyl or arylalkyl, or annelation leading to tricyclic structures. Moreover, a preliminary structure–activity relationship (SAR) analysis of xanthine derivatives with extended alkyl substituents at the *N*1- and the *N*3-position was initiated. The final compounds were tested in vitro for their inhibitory potency on MAO-B, PDE4, and PDE10, and for their interaction with adenosine and dopamine receptor subtypes.

Xanthine and dopamine fragments, which are present in designed compounds, were reported in the literature to act as potent radical scavengers [45,46]. Since oxidative stress is postulated as one of the factors contributing to neurodegeneration, the selected compounds of the current series were additionally tested in vitro for their potential to exhibit antioxidant properties. Furthermore, a cellular model was employed to investigate whether the newly designed compounds are able to provide neuronal protection against oxidative damage.

## 2. Results and Discussion

### 2.1. Chemistry

**Scheme 1:**
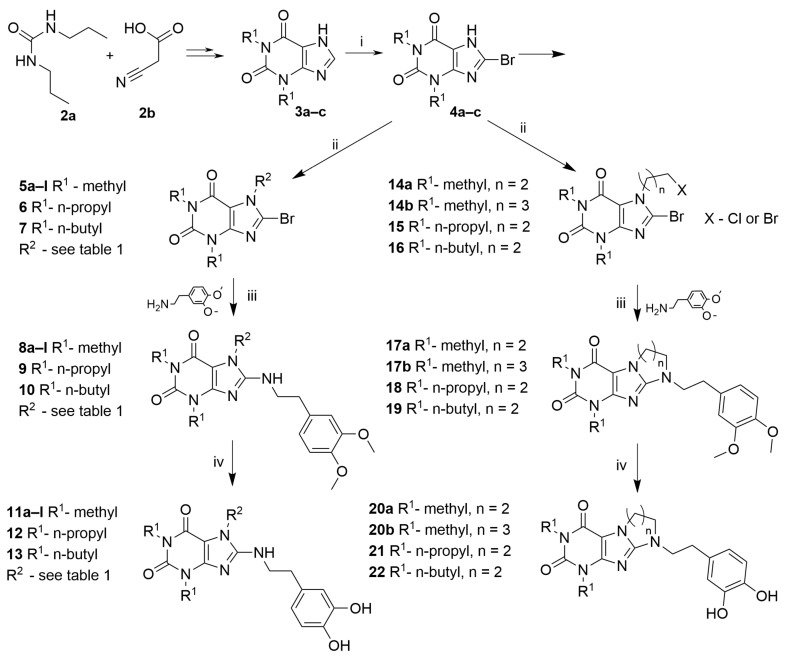
Synthesis of final compounds: (i) 40% HBr, NaClO_3_, CH_3_COOH, 60 °C; (ii) (aryl(oxy))alkyl bromide (or chloride/iodide)/1-bromo-3-chloropropane/1,4-dibromobutane, benzyltriethylammonium chloride (TEBA), K_2_CO_3_, acetone, reflux, 10 h; (iii) Me-Digol/DMF, reflux, (iv) a—40% HBr reflux, b—20% Na_2_CO_3_ to pH 8–9.

The synthetic strategy to obtain xanthine derivatives fused with a dopamine fragment included: first, the preparation of the xanthine part of the molecules, and then, the conjugation with a dopamine moiety whose hydroxy functions were protected by methyl groups (Scheme 1).

The synthesis of the xanthine scaffold started from commercially available theophylline (**3a**) for 1,3-dimethyl derivatives, or from 1,3-dibutylxanthine (**3c**) for 1,3-dibutyl analogues. The 1,3-dipropylxanthine (**3b**) was derived from 1,3-dipropylurea (**2a**), via a modified Traube synthesis. The 1,3-dialkylxanthines were oxidatively brominated in the 8-position (**4a**–**c**), and *N*7-alkylated according to previously described procedures [47,48]. For tricyclic compounds, dihalogenoalkanes were introduced in the *N*7-position, yielding 7-Cl(Br)-propyl- or -butyl-8-bromoxanthine derivatives **14a**–**b**, **15**, **16**. The reaction of 3,4-dimethoxyphenylethylamine with the xanthine core was carried out by refluxing the compounds in a suitable solvent (for details, see Experimental Protocols section). In this step, cyclic condensation was performed in DMF for tricyclic compounds, resulting in pyrimido- or diazepino-[2,1-*f*]purinedione derivatives **17a**–**b**, **18**, **19**.

The deprotection of the methoxy groups in the 3,4-dimethoxyphenylethylamino-substituted compounds was carried out via reflux with 48% or 40% HBr and alkalization with 20% Na_2_CO_3_ to pH 8–9, leading to the final products.

The structure of all compounds was confirmed by elemental analyses and UV, IR, and NMR spectra. The UV spectra showed a bathochromic shift of λ_max_ from 275 nm to about 290 nm (xanthine compounds) or from 275 nm to about 300 nm (tricyclic compounds), typical for 8-aminoxanthines [49]. Compounds showed IR absorption bands typical for xanthine derivatives [50], and in proton and carbon NMR spectra, adequate shifts could be observed. The purity of all tested compounds was at least 95%, determined by UPLC/MS. Spectroscopic data are presented in Supplementary Data.

### 2.2. Biological Evaluation

#### 2.2.1. Monoamine Oxidase Inhibition

The final compounds were evaluated in vitro for their human MAO-B (*h*MAO-B) inhibitory potencies, and selected structures were also investigated for human MAO-A (*h*MAO-A) inhibition. The results are presented in Table 1.

All structures displayed high MAO-B inhibitory potency, with *IC*_50_ values in the nanomolar concentration range. The results strongly support the utility of a hybrid dopamine–xanthine scaffold for activity towards MAO-B. Compound **11a**, a caffeine combined with a dopamine moiety, presented high MAO-B inhibitory potency (*IC*_50_ = 50.7 nM). The elongation of the alkyl chain in the *N*7-position (**11b**, **11e**, **11h**, **11i**) had no or only a slight impact on biological activity, maintaining potencies in the same range. The exception was compound **11c**, containing a propyl residue, which displayed three-fold lower inhibition potency. A similar effect was also observed for structures with branched alkyl substituents (**11d, 11f, 11g**). In contrast, compounds with aromatic residues at the *N*7-position (**11j, 11k, 11l**) showed comparable MAO-B inhibitory potencies to structures with linear alkyl chains. The modification of compound **11c** via the introduction of longer propyl (**12**) or butyl (**13**) substituents at the *N*1- and *N*3-positions slightly improved the inhibitory activity compared to the 1,3-dimethyl analogue.

Tricyclic derivatives (**20a**, **20b**, **21**, **22**) generally showed somewhat lower MAO-B inhibitory potency; however, the *IC*_50_ values were still in the nanomolar concentration range. The most active tricyclic compound, **20b** (*IC*_50_ = 101 nM) contained a 1,3-diazepino ring and a short methyl chain at the *N*1- and *N*3-positions.

Within the obtained library of compounds, only two compounds (**11i**, **11k**) showed significant MAO-A inhibitory activity (*IC*_50_ < 500 nM), indicating that the compounds were selective for MAO-B, as desired.

#### 2.2.2. Adenosine Receptors

The final compounds were tested in radioligand binding assays to evaluate their affinity for all four ARs subtypes (see Table 2) [51,52]. Rat brain cortical membrane preparations were used as a source of rat A_1_ (*r*A_1_) ARs, and rat brain striatal membrane preparations served as a source of rat A_2A_ (*r*A_2A_) ARs. For A_2B_- and A_3_AR assays, membrane preparations of Chinese hamster ovary (CHO) cells recombinantly expressing either human A_2B_ (*h*A_2B_) or human A_3_ (*h*A_3_) ARs were employed.

8-(3,4-Dihydroxyphenylethylamino)caffeine (**11a**) was found to be a moderately potent A_2A_AR ligand with additional micromolar affinity for the A_1_AR. The elongation of the alkyl chain at the *N*7-position (in **11b**, **11c**, **11e**, **11h**) resulted in increased affinity and selectivity for the A_1_AR subtype. Compound **11b** (8-(3,4-dihydroxyphenylethylamino)-7-ethyltheophylline) presented balanced, dual A_1_/A_2A_ AR affinity (A_1_AR, *K*_i_ = 479 nM; A_2A_AR, *K*_i_ = 672 nM). A significant decrease in affinity at both A_1_- and A_2A_ARs was observed for compound **11i** containing an *N*7-hexyl substituent. Furthermore, the *N*7-propyl derivative **11c** presented significantly lower affinity than the *N*7-ethyl (**11b**) and *N*7-butyl (**11e**) analogues at both A_1_- and A_2A_ARs. Interestingly, compounds with *N*7-propyl substituent and longer alkyl chains, such as di-propyl (**12**; A_1_ AR, *K*_i_ = 130 nM; A_2A_ AR, *K*_i_ = 833 nM) or di-butyl (**13**) fragments introduced at the *N*1,*N*3-positions, displayed higher affinity towards both A_1_- and A_2A_ARs than the *N*1,*N*3-dimethyl-substituted analogue. 

The replacement of a linear alkyl chain at the *N*7-position by a branched one (**11d**, **11f**, **11g**) resulted in low, micromolar affinity at both A_2A_- and A_1_ARs, with the exception of compound **11d**, displaying a sub-micromolar *K_i_* value at the A_1_AR. A comparable effect was observed in the group containing an aromatic phenyl ring connected by a short linear linker to the xanthine core at the *N*7-position (**11j**, **11k**, **11l**). Here, only compound **11l** with phenoxyethyl residue at the *N*7-position presented high affinity for the A_1_AR (*K*_i_ = 124 nM). 

In the group of tricyclic derivatives, compound **20a** (*N*9-(3,4-dihydroxyphenylehyl)-1,3-dimethyltetrahydroprymido[2,1*-f*]purinedione) was found to be a moderately potent A_2A_AR ligand (*K*_i_ = 507 nM). The enlargement of the third heterocyclic ring fused to the *f*-bond (**20b**) resulted in an increase in affinity for the A_2A_AR (*K*_i_ = 234 nM). A loss of selectivity and a significant enhancement of AR affinity was observed for compounds **21** and **22**, with a longer alkyl chain at the *N*1,*N*3-postions. Compound **21** showed triple A_1_/A_2A_/A_2B_AR affinity compared to compound **22** binding to all four AR subtypes with nanomolar affinities.

#### 2.2.3. Affinity at Dopamine Receptors

The final compounds were tested in radioligand displacement assays to evaluate their affinity to dopamine D_1_ and D_2_ receptors (see Table 2). Rat brain cortical membranes were used as source of native rat D_1_ receptor protein, and human D_2_ receptor was recombinantly expressed in HEK293 cells.

None of the tested compounds was found to interact with dopamine D_1_ receptor, at least at the highest tested concentration of 1 µM. Therefore, the presented compounds may be considered inactive or very weak D_1_ receptors ligands.

The dopamine D_2_ receptor affinity for final structures was determined to be in the micromolar range. The most potent compound **13** (D_2_R *K*_i_ = 4.39 µM) was 1,3-dibutyl-8-((3,4-dihydroxyphenethyl)amino)-7-propyl-3,7-dihydro-1*H*-purine-2,6-dione). Compounds **11i** and **20a** displayed lower affinity at the D_2_ receptor, with *K_i_* values of approximately 6.6 µM, showing various substitution patterns. Compound **11i** is a 7-hexyltheophylline derivative, while **20a** has a tricyclic scaffold based on a pyrimido[2,1-*f*]purinedione core with a butyl chain at *N*1 and *N*3. Other final compounds showed significantly lower affinity towards the dopamine D_2_ receptor.

**Figure 1:**
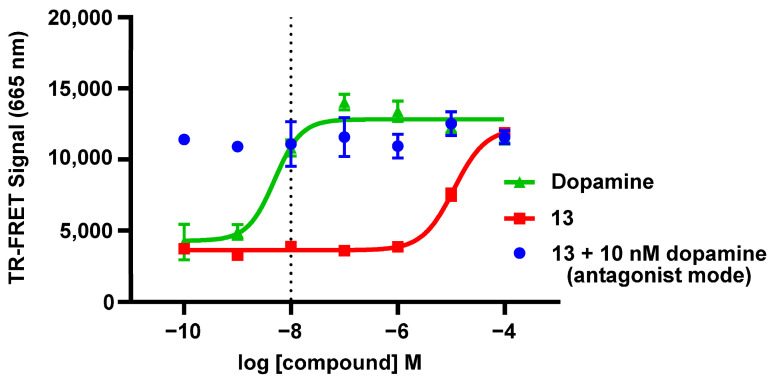
cAMP accumulation in forskolin-stimulated HEK293 cells expressing human dopamine D_2L_ receptors. cAMP production was measured using LANCE Ultra cAMP Detection Kit (PerkinElmer, Waltham, MA, USA), where measured TR-FRET signal is inversely proportional to cAMP levels in the cells. Dopamine, *EC*_50_ = 4.94 nM; 13, *EC*_50_ = 11.3 μM.

Compound **13**, as the most potent D_2_R ligand in this series, was evaluated for its intrinsic activity in a functional cAMP assay, and it showed agonistic properties with an *EC*_50_ value of 11.3 µM (Figure 1). This feature can be considered beneficial for the treatment of PD to elevate decreasing D_2_ transmission in the nigro-striatal pathway related to the neurodegenerative process.

#### 2.2.4. Phosphodiesterase Inhibition

Further biological evaluation of the presented compound library was performed determining inhibitory potencies against selected human PDE isoforms, namely, PDE4 and PDE10 (for results see Table 3).

PDE4B1 was most highly inhibited by 8-(3,4-dihydroxyphenethylamino)-7-isopropyl-1,3-dimethyl-1*H*-purine-2,6(3*H*,7*H*)-dione (**11d**, *IC*_50_ = 2.44 µM) among the tested structures. Slightly lower activities were determined for compounds **11b**, **11c**, **11e**, **11f**, **11g**, and **11h** with a short (2–5 carbons), straight, or branched alkyl chain in the *N*7-position displaying *IC*_50_ values in the range of 2.5–5 µM. Structures **11j** and **11k** with *N*7-benzyl and *N*7-phenylethyl substituent, respectively, showed an *IC*_50_ value around 3–5 µM, as did most of *N*7-alkyl-subsituted derivatives. All other modifications in the *N*7-position, including annelation via the *f*-bond, as well as elongation of alkyl chains in the *N*1- and *N*3-positions, resulted in a significant decrease in or loss of activity towards PDE4B1.

Regarding PDE10A1, the most active compound was **11d** (*IC*_50_ = 2.30 µM), which was also the most potent dual PDE4B1/PDE10A inhibitor. The introduction of a short, straight alkyl chain with 2–4 carbons in the *N*7-position (**11b**, **11c**, **11e**) maintained an *IC*_50_ value in the range of 3–5 µM. A similar inhibitory potency was also observed for compound **11k** with an *N*7-phenylethyl substituent. The modification of the linker between the xanthine core and the aromatic ring in the *N*7-position (**11j**, **11l**) resulted in a decrease in inhibitory potency in PDE10A. A significant reduction in inhibitory activity was also observed for the rest of the tested compounds, including the series of tricyclic structures, except for *N*1,*N*3-dipropyl derivative (**12**), which maintained the activity in a low micromolar range.

### 2.3. Determination of Antioxidant Activity

**Figure 2:**
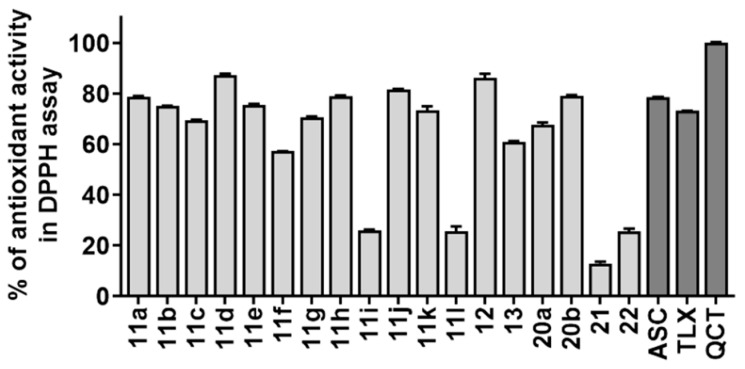
Antioxidant properties of xanthine derivatives evaluated in the DPPH test. All compounds were tested at 50 µM concentration. Results were normalized to the antioxidant activity measured for quercetin. ASC, ascorbic acid; TLX, trolox; QCT, quercetin.

The 2,2-diphenyl-1-picrylhydrazyl (DPPH) assay was used to determine the antioxidant properties of selected compounds using ascorbic acid, trolox, and quercetin as references. The study demonstrated that most of the tested xanthine derivatives have high capacity for DPPH reduction and that their free radical scavenging activity exceeds 50% of the antioxidant effect of reference quercetin tested at the same concentration (Figure 2 and Table 4). Among the evaluated structures, four compounds, **11i**, **11l**, **21**, and **22,** represented significantly lower antioxidative activity than quercetin. From a structural point of view, **21** and **22** share a common pyrimido[2,1-*f*]purinedione core with propyl or butyl substituents at the *N*1- and *N*3-position. Compounds **11i** and **11l**, containing hexyl or phenoxyethyl as *N*7-substituents, were the only structures in the groups of bicyclic xanthine derivatives that lacked antioxidant activity in the DPPH assay.

### 2.4. Neuroprotective Effects of Hybrid Compounds in a Cellular Model

Based on their promising antioxidant potency, selected xanthine derivatives were further tested for neuroprotective effects in an oxidative-stress-induced cell death model. Oxidative stress plays an important role in the degeneration of dopaminergic neurons in Parkinson’s disease (PD), but also in other neurodegenerative diseases [55]. Compounds with capacity to scavenge reactive oxygen species could potentially represent beneficial neuroprotective properties in neurodegeneration therapies [56].

For neuroprotection studies, a cellular model of neuroblastoma SH-SY5Y cells exposed to hydrogen peroxide was applied. Lactate dehydrogenase (LDH) release was used as a measure of cell death. First, the compounds (**11a**, **11c**, **12**, **13**, **20a**, **21**, and **22**) were tested for their possible intrinsic neurotoxicity to the SH-SY5Y cell line (see Supplementary Figure S1). One of the tested compounds (**13**) showed significant cytotoxicity at 50 µM concentration and was excluded from further evaluation. All other compounds were tested for cytoprotective effects against H_2_O_2_-induced toxicity in SH-SY5Y cells.

**Figure 3:**
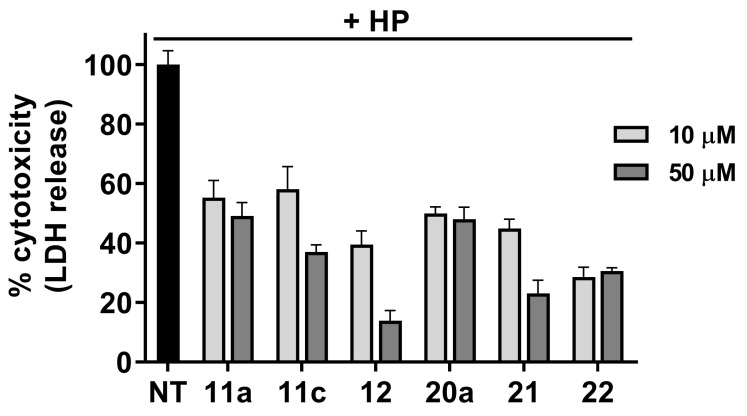
The ability of tested compounds to reduce the cytotoxicity of 300 μM hydrogen peroxide (HP) on dopaminergic neuronal cells (SH-SY5Y) as judged by LDH assay. Data represent the mean of two experiments performed in triplicate. Results are presented as percent of cytotoxicity induced by 300 µM hydrogen peroxide (HP) ± SEM. NT–vehicle control (1% DMSO).

The compounds presented protective effects, significantly decreasing cytotoxicity induced by the reactive oxygen species (Figure 3). The most potent compounds were **12**, **21**, and **22**. Dose-dependent responses were observed for **11c**, **12**, and **21**. Interestingly, the considered structures represented cytoprotective activity in SH-SY5Y cells exposed to oxidative stress, independent on the degree of their antioxidant potency determined in the DPPH assay. Although the antioxidant activity could be one of the functionalities that drive the protection against the accumulation of reactive oxygen species in neural cells, other mechanisms of action were also proposed for neuroprotective agents. For example, it has been widely reported that MAO-B inhibitors possess the ability to attenuate the toxic effects in progression of neurodegenerative diseases [21,57]. Since all of the compounds tested in neuroprotection experiments represented high inhibitory activity at MAO-B (*IC*_50_ values: 50.7–170 nM), the retaining of neuroprotective activity by compounds **21** and **22**, which lacked antioxidant properties, could potentially be attributed to their activity towards MAO-B.

## 3. Conclusions

A library of 19 novel dopaminyl-substituted derivatives of purinediones was synthesized. Systematic modification of the substituent in the *N*7-position of the xanthine core was performed, and SARs were analyzed regarding MAO-B, PDE4, and PDE10, adenosine and dopamine receptors. The hybrid dopaminyl–xanthine scaffold showed high MAO-B inhibitory potency with *IC*_50_ values in the range of 44 to 205 nM. On the other side, SARs for PDE inhibition and AR affinity were steeper, and more rigid structural requirements were observed. For PDE inhibitory activity, the presence of a short 2–4 carbon chain, or a benzyl or phenylethyl residue, in position *N*7 was required. AR affinity was increased by the introduction of a methyl substituent in position *N*7 or by annelation of a third ring to the *f*-bond. Although a dopaminyl fragment was present, all compounds showed low or only moderate affinity to dopamine receptors. For the most active compound **13**, D_2_R agonistic properties were confirmed, which is desired for the intended application, namely, the treatment of neurodegenerative diseases by multitarget drugs. In the light of the above, novel potent MAO-B inhibitors/adenosine receptor antagonists were identified in the current series, with ancillary activity at PDE isoforms. Although more balanced activity at all considered biological targets would be expected for optimized multitarget drugs, our early-stage study provided an insight into the key structural elements that may introduce additional beneficial properties to MAO-B/A_2A_AR dual compounds that may constitute for leading structures in further studies. An additional advantage of the discussed hybrid compounds is their antioxidant activity, determined in the DPPH assay, and neuroprotective properties observed in an oxidative-stress-induced neuroblastoma cell death model.

Those above outcomes justify the conclusion that the presented hybrid chemical scaffold may be considered a novel multitarget drug entity in the search for potent anti-neurodegenerative agents. The preference of the dopaminyl–xanthine core for MOA-B inhibition indicates its suitability for further structural optimization towards dual targeting MAO-B/PDE and MAO-B/AR agents.

## 4. Experimental Protocols

### 4.1. Chemistry

#### 4.1.1. Materials and Methods

All commercially available reagents and solvents were used without further purification. Melting points (mp.) were determined on a MEL-TEMP II (LD Inc., Kansas City, KS, USA) melting point apparatus and were uncorrected. IR spectra were taken as KBr discs on a Jasco FT/IR-410 spectrometer. UV spectra were recorded on Jasco UV-Vis V530 in concentration of 10^−5^ mol/L in methanol. ^1^H-NMR spectra were determined with a Varian Mercury 300 MHz apparatus in CDCl_3_ (3,4-dimethoxyphenylethyl derivatives) or in DMSO d_6_ (dopaminyl derivatives) using tetramethylsilane as an internal standard. ^13^C NMR data were recorded on a 75 MHz on Varian-Mercury-VX 300 MHz PFG spectrometer. The *J* values are reported in Hertz (Hz), and the splitting patterns are designated as follows: s (singlet), d (doublet), t (triplet), dd (doublet of doublets), dt (doublet of triplets), quin (quintet), and m (multiplet). Elemental analyses (CHN) were performed on an Elemental Vario-El III (Hanau, Germany) apparatus and were in accordance with theoretical values within ± 0.5%. The purity of the tested compounds was determined (%) on Waters TQD mass spectrometer coupled with a Waters ACQUITY UPLC system. Retention times (*t*_R_) are presented in minutes. The reactions were monitored via thin-layer chromatography (TLC) using aluminum sheets coated with silica gel 60F254 (Merck, Darmstadt, Germany) as developing system cyclohexane/dioxane 1:1. Spots were detected under UV light.

The synthesis and physicochemical properties of the compounds **14a**–**b**, **15**, and **16** were reported previously [58,59,60].

#### 4.1.2. General Procedure for the Synthesis of 1,3-Dialkyl-8-(3′,4′-dimethoxyphenylethyl)-amino-7-alkyl- (or phenylalkyl- or phenoxyethyl-) Xanthines

A mixture of 2 mmol of appropriate 7-alkyl, 7-phenylalkyl,7-phenoxyethyl-1,3-dialkyl-8-bromoxanthine, and 4 mmol of 3′4′-dimethoxyphenylethylamine was refluxed in 10 mL of Me-Digol or 10 mL of 1-butanol for 4–13 h. The products were separated by cooling or by adding water to the reaction mixture. The resulting solids were filtered off and recrystallized.

The yields and physical data of the 1,3-dialkyl-8-(3′,4′-dimethoxyphenylethyl-amino)-7-alkyl (phenylalkyl, phenoxyethyl)-xanthines (**8a**–**l**, **9**, **10**) are presented in Supplementary Data.

#### 4.1.3. General Procedure for the Synthesis of 1,3-Dialkyl-8-3′,4′-dihydroxyphenylaminoethyl-7-alkyl- (or phenylalkyl- or phenoxyalkyl-) Xanthines (**11a**–**l**, **12**, **13**)

A mixture of 1,3-dialkyl-8-3′,4′-dimethoxyphenylethyl-7-alkyl (phenylalkyl, phenoxyethyl) xanthine was refluxed with 10 mL of 48% HBr for 2–5 h. The products were separated after cooling and alkalization of acidic mixture by 20% Na_2_CO_3_ to pH 8–9, washing by water, and dried. All compounds were purified by recrystallization.

##### 8-((3,4-Dihydroxyphenethyl)amino)-1,3,7-trimethyl-3,7-dihydro-1*H*-purine-2,6-dione (**11a**)

Time: 3 h. Yield: 98%; crystallized from ethanol; mp: 204–206 °C; ^1^H NMR (300 MHz, DMSO-*d*_6_) δ ppm: 2.70 (t, *J* = 7.57 Hz, 2H, CH_2_CH_2_), 3.34 (s, 6H, N1CH_3_ + N7CH_3_), 3.38–3.42 (m, 2H, CH_2_CH_2_), 3.51 (s, 3H, N3CH_3_), 6,45 (d, *J* = 8.21 Hz, 1H, C5H, phe), 6.59–6.64 (m, 2H, C2H, C6H, phe), 7.05 (t, *J* = 5.39 Hz, 1H, NH), 8.68 (s, 2H, 2 × OH); ^13^C NMR (DMSO-*d*_6_) δ ppm: 154.4 (C4, phe), 153.3 (C6), 151.4 (C2), 148.8 (C4), 145.6 (C5), 144.0 (C3, phe), 130.6 (C1, phe), 119.8 (C2, phe), 116.5 (C5, phe), 115.6 (C6, phe), 102.8 (C_5_), 50.0 (NHCH_2_), 35.3 (NHCH_2_CH_2_), 30.1 (N1CH_3_), 29.7 (N3CH_3_), 27.6 (N7CH_3_); IR ν (cm^−1^): 3375 (NH), 3288 (OH), 1695 (CO), 1621 (CO); UPLC/MS purity 100.00%, *t*_R_ = 3.45, C_16_H_19_N_5_O_4_, MW 345.36, [M + H]^+^ 346.31. Anal. Elem.: C, 55.65; H, 5.55; N, 20.28; Found: C, 55.31; H, 5.55; N, 20.14.

##### 8-((3,4-Dihydroxyphenethyl)amino)-7-ethyl-1,3-dimethyl-3,7-dihydro-1*H*-purine-2,6-dione (**11b**)

Time: 5 h. Yield: 90%; crystallized from acetonitrile + ethanol; mp: 225–226 °C; ^1^H NMR (300 MHz, DMSO-*d*_6_) δ ppm: 1.13 (t, *J* = 7.05 Hz, 3H, CH_2_CH_3_), 2.68 (t, *J* = 7.44 Hz, 2H, CH_2_CH_2_), 3.16 (s, 3H, N1CH_3_), 3.35 (s, 3H, N3CH_3_), 3.42 (q, *J* = 6.54 Hz, 2H, CH_2_CH_2_), 4.00 (q, *J* = 6.80, Hz, 2H, N7CH_2_CH_3_), 6.44 (dd, *J* = 7.93 Hz, 1H, C5H, phe), 6.58–6.63 (m, 2H, C2H, C6H, phe), 7.08 (t, *J* = 5.06 Hz, 1H, NH), 8.68 (s, 2H, 2 × OH); ^13^C NMR (DMSO-*d*_6_) δ ppm: 153.4 (C4, phe), 153.1 (C6), 151.5 (C2), 149.1 (C4), 145.5 (C5), 144.0 (C3, phe), 130.6 (C1, phe), 119.8 (C2, phe), 116.6 (C5, phe), 115.9 (C6, phe), 101.5 (C5), 44.9 (NHCH_2_), 37.9 (N7CH_2_), 35.2 (NHCH_2_CH_2_), 29.7 (N1CH_3_), 27.7 (N3CH_3_), 15.2 (N7CH_2_CH_3_); IR ν (cm^−1^): 3342 (NH), 1684 (CO), 1645 (CO), 818 (aryl); UPLC/MS purity 100.00%, *t*_R_ = 3.79, C_17_H_21_N_5_O_4_, MW 359.39, [M + H]^+^ 360.27. Anal. Elem.: C, 56.82; H, 5.89; N, 19.49; Found: C, 56.55; H, 5.98; N, 19.47.

##### 8-((3,4-Dihydroxyphenethyl)amino)-1,3-dimethyl-7-propyl-3,7-dihydro-1*H*-purine-2,6-dione (**11c**)

Time: 3 h. Yield: 92%; crystallized from ethanol + H_2_O; mp: 200–203 °C; ^1^H NMR (300 MHz, DMSO-*d*_6_) δ ppm: 0.79 (t, *J* = 7.31 Hz, 3H, CH_2_CH_2_CH_3_); 1.54–1.61 (m, 2H, CH_2_CH_2_CH_3_); 2.68 (t, *J* = 7.44 Hz, 2H, CH_2_CH_2_); 3.16 (s, 3H, N1CH_3_),3.33 (s, 3H, N3CH_3_), 3.42 (q, *J* = 6.16 Hz, 2H, CH_2_CH_2_), 3.92 (t, *J* = 7.18 Hz, 2H, CH_2_CH_2_CH_3_), 6.44 (d, *J* = 7.93 Hz, 1H, C5H, phe), 6.59–6.63 (m, 2H, C2H, C6H, phe), 7.06 (t, *J* = 5.51 Hz, 1H, NH), 8.69 (d, *J* = 6.16 Hz, 2H, 2 × OH); ^13^C NMR (DMSO-*d*_6_) δ ppm: 153.7 (C4, phe), 153.0 (C6), 151.4 (C2), 149.1 (C4), 149.0 (C8), 147.7 (C3, phe), 132.4 (C1, phe), 121.1 (C2, phe), 113.1 (C5, phe), 112.3 (C6, phe), 101.8 (C5), 56.0 (N7CH_2_), 44.6 (NHCH_2_), 35.3 (NHCH_2_CH_2_), 29.7 (N_1_CH_3_), 28.4 (N_3_CH_3_), 21.2 (N7CH_2_CH_2_), 14.3 (N_7_CH_2_CH_2_CH_3_); IR ν (cm^−1^): 3370 (NH), 1682 (CO), 1642 (CO), UPLC/MS purity 100.00%, *t*_R_ = 4.14, C_18_H_23_N_5_O_4_, MW 373.41, [M + H]^+^ 374.36. Anal. Elem.: C, 57.90; H, 6.21; N, 18.76; Found: C, 57.96; H, 5.99; N, 18.69.

##### 8-((3,4-Dihydroxyphenethyl)amino)-7-isopropyl-1,3-dimethyl-3,7-dihydro-1*H*-purine-2,6-dione (**11d**)

Time: 5 h. Yield: 81%; crystallized from methanol; mp: 180–182 °C; ^1^H NMR (300 MHz, DMSO-*d*_6_) δ ppm: 1.41 (d, *J* = 6.92 Hz, 6H, CH(CH_3_)_2_), 2.68 (t, *J* = 7.44 Hz, 2H, CH_2_CH_2_), 3.18 (s, 3H, N1CH_3_), 3.32–3.44 (m, 5H, N3CH_3_ + CH_2_CH_2_), 4.44–4.51 (m, 1H, CH(CH_3_)_2_), 6.43 (dd, *J* = 7.95 Hz, 1H, C5H, phe), 6.57–6.62 (m, 2H, C2H, C6H, phe), 6.98 (s, 1H, NH), 8.64 (s, 1H, 4′OH), 8.72 (s, 1H, 3′OH); ^13^C NMR (DMSO-*d*_6_) δ ppm: 153.2 (C4, phe), 152.7 (C6), 151.4 (C2), 150.2 (C4), 145.5 (C8), 144.0 (C3, phe), 130.6 (C1, phe), 119.8 (C2, phe), 116.6 (C5, phe), 115.9 (C6, phe), 101.8 (C5), 49.1 (N7CH), 46.5 (NHCH_2_), 35.1 (NHCH_2_CH_2_), 29.8 (N1CH_3_), 28.2 (N3CH_3_), 21.2 (N7CH(CH_3_)_2_); IR ν (cm^−1^): 3666 (NH), 3366 (OH), 2970–2942 (alkyl), 1681 (CO), 1616 (CO), 889 (aryl); UPLC/MS purity 95.26%, *t*_R_ = 4.23, C_18_H_23_N_5_O_4_, MW 373.41, [M + H]^+^ 374.36. Anal. Elem.: C, 57.90; H, 6.21; N, 18.76; Found: C, 57.96; H, 5.99; N, 18.69.

##### 7-Butyl-8-((3,4-dihydroxyphenethyl)amino)-1,3-dimethyl-3,7-dihydro-1*H*-purine-2,6-dione (**11e**)

Time: 3 h. Yield: 99%; crystallized from ethanol + H_2_O; mp: 165–167 °C; ^1^H NMR (300 MHz, DMSO-*d*_6_) δ ppm: 0.84 (t, *J* = 7.31 Hz, 3H, CH_2_CH_2_CH_2_CH_3_), 1.14–1.24 (m, 2H, CH_2_CH_2_CH_2_CH_3_), 1.47–1.57 (m, 2H, CH_2_CH_2_CH_2_CH_3_), 2.68 (t, *J* = 7.44 Hz, 2H, CH_2_CH_2_), 3.15 (s, 3H, N1CH_3_), 3.35 (s, 3H, N3CH_3_), 3.42 (t, *J* = 6.16 Hz, 2H, CH_2_CH_2_), 3.56 (t, *J* = 7.18 Hz, 2H, CH_2_CH_2_CH_2_CH_3_), 5.55 (d, *J* = 7.95 Hz, 1H, C5H, phe), 6.58–6.62 (m, 2H, C2H, C6H, phe), 7.02 (t, *J* = 5.26 Hz, 1H, NH), 8.67 (s, 2H, 2OH); ^13^C NMR (DMSO-*d*_6_) δ ppm: 153.7 (C4, phe), 153.0 (C6), 151.5 (C2), 149.0 (C4), 145.5 (C8), 144.0 (C3, phe), 130.6 (C1, phe), 119.8 (C2_,_ phe), 116.6 (C5, phe), 115.9 (C6, phe), 101.8 (C5), 44.9 (NHCH_2_), 42.6 (N7CH_2_), 35.2 (NHCH_2_CH_2_), 31.7 (N1CH_3_), 29.7 (N3CH_3_), 27.7 (N7CH_2_CH_2_), 19.6 (N7CH_2_CH_2_CH_2_), 14.1 (N7CH_2_CH_2_CH_2_ CH_3_); IR ν (cm^−1^): 3377 (NH), 3298–3292 (OH), 2947–2869 (alkyl), 1683 (CO), 1637 (CO), 886 (aryl); UPLC/MS purity 99.44%, *t*_R_ = 4.65, C_19_H_25_N_5_O_4_, MW 387.44, [M + H]^+^ 388.32. Anal. Elem.: C, 58.90; H, 6.50; N, 18.08; Found: C, 59.00; H, 6.53; N, 18.28.

##### 8-((3,4-Dihydroxyphenethyl)amino)-7-isobutyl-1,3-dimethyl-3,7-dihydro-1*H*-purine-2,6-dione (**11f**)

Time: 2 h. Yield: 75%; crystallized from ethanol + H_2_O; mp: 201–203 °C; ^1^H NMR (300 MHz, DMSO-*d*_6_) δ ppm: 0.78 (d, *J* = 6.67 Hz, 6H, (CH_3_)_2_CHCH_2_), 1.99–2.04 (m, 1H, CH), 2.67 (t, *J* = 7.44 Hz, 2H, CH_2_CH_2_), 3.15 (s, 3H, N1CH_3_), 3.36–3.45 (m, 5H, N3CH_3_, CH_2_CH_2_), 3.77 (d, *J* = 7.44 Hz, 2H, CH_2_CH(CH_3_)_2_), 6.43 (dd, *J* = 7.95 Hz, 1H, C5H, phe), 6.58–6.62 (m, 2H, C2H, C6H, phe), 6.98 (s, 1H, NH), 8.50–8.80 (s, 2H, 2OH); ^13^C NMR (DMSO-*d*_6_) δ ppm: 154.0 (C4, phe), 153.0 (C6), 151.4 (C2), 149.0 (C4), 145.52 (C8), 144.0 (C3, phe), 130.5 (C1, phe), 119.8 (C2, phe), 116.5 (C3, phe), 115.9 (C6, phe), 102.2 (C5), 49.4 (N7CH_2_), 44.9 (NHCH_2_), 35.3 (NHCH_2_CH_2_), 29.7 (N1CH_3_), 28.6 (N3CH_3_), 27.7 (N7CH_2_CH), 19.6 (N7CH_2_CH(CH_3_)); IR ν (cm^−1^): 3389 (NH), 2958–2872 (alkyl), 1680 (CO), 1642 (CO), 752 (aryl); UPLC/MS purity 98.67%, *t*_R_ = 4.58, C_19_H_25_N_5_O_4_, MW 387.44, [M + H]^+^ 388.32. Anal. Elem.: C, 58.90; H, 6.50; N, 18.08; Found: C, 59.07; H, 6.69; N, 17.69.

##### 7-(Sec-butyl)-8-((3,4-dihydroxyphenethyl)amino)-1,3-dimethyl-3,7-dihydro-1*H*-purine-2,6-dione (**11g**)

Time: 2 h. Yield: 77%; crystallized from methanol; mp: 163–165 °C; ^1^H NMR (300 MHz, DMSO-*d*_6_) δ ppm: 0.65 (t, *J* = 7.31 Hz, 3H, CH(CH_3_)CH_2_CH_3_), 1.41 (d, *J* = 6.92 Hz, 3H, (CH(CH_3_)CH_2_CH_3_), 1.64–1.73, 1.98–2.06 (m, 2H, CH(CH_3_)CH_2_CH_3_), 2.67 (t, *J* = 7.44 Hz, 2H, CH_2_CH_2_), 3.17 (s, 3H, N1CH_3_), 3.37–3.45 (N3CH_3_, CH_2_CH_2_), 4.25 (6s, 1H, CH), 6.43 (dd, *J* = 7.95 Hz, 1H, C5H, phe), 6.58–6.62 (m, 2H, C2H, C6H, phe), 6.90 (s, 1H, NH), 8.50–8.80 (s, 2H, 2OH); ^13^C NMR (DMSO-*d*_6_) δ ppm: 154.0 (C4, phe), 152.7 (C6), 151.4 (C2), 150.2 (C4), 145.5 (C8), 144.0 (C3, phe), 130.6 (C1, phe), 119.8 (C2, phe), 116.6 (C5, phe), 114.9 (C6, phe), 101.8 (C5), 52.4 (N7CH), 49.1 (N7CH_2_CH_2_), 45.1(NHCH_2_), 35.2 (NHCH_2_CH_2_), 29.8 (N1CH_3_), 28.14 (N3CH_3_), 19.9 (N7CHCH_3_), 11.4 (N7CH(CH_3_)CH_2_CH_3_); IR ν (cm^−1^): 3376 (NH), 2969–2876 (alkyl), 1680 (CO), 1613 (CO), 754 (aryl); UPLC/MS purity 97.60%, *t*_R_ = 4.61, C_19_H_25_N_5_O_4_, MW 387.44, [M + H]^+^ 388.18. Anal. Elem.: C, 58.90; H, 6.50; N, 18.08; Found: C, 59.05; H, 6.55; N, 17.80.

##### 8-((3,4-Dihydroxyphenethyl)amino)-1,3-dimethyl-7-pentyl-3,7-dihydro-1*H*-purine-2,6-dione (**11h**)

Time: 4 h. Yield: 90%; crystallized from ethanol + H_2_O; mp: 173–175 °C; ^1^H NMR (300 MHz, DMSO-*d*_6_) δ ppm: 0.82 (t, *J* = 7.05 Hz, 3H, CH_2_CH_2_CH_2_CH_3_), 1.15–1.30 (m, 4H, CH_2_CH_2_CH_2_CH_2_CH_3_), 1.50–1.59 (m, 2H, CH_2_CH_2_CH_2_CH_2_CH_3_), 2.64 (t, *J* = 7.4 Hz, 2H, CH_2_CH_2_), 3.15 (s, 3H, N1CH3), 3.35 (s, 3H, N3CH_3_), 3.42 (q, *J* = 6.16 Hz, 2H, CH_2_CH_2_), 3.94 (t, *J* = 7.18 Hz, 2H, CH_2_CH_2_CH_2_CH_2_CH_3_), 6.43 (dd, *J* = 7.95 Hz, 1H, C5H, phe), 6.58–6.62 (m, 2H, C2H, C6H, phe), 7.03 (t, *J* = 5.64 Hz, 1H, NH), 8.63 (s, 1H, 4′OH), 8.71 (s, 1H, 3′OH); ^13^C NMR (DMSO-*d*_6_) δ ppm: 153.7 (C4, phe), 153.0 (C6), 151.4 (C2), 149.0 (C4), 145.5 (C8), 144.0 (C3, phe), 130.6 (C1, phe), 119.8 (C2, phe), 116.5 (C5, phe), 115.9 (C6, phe), 101.9 (C5), 44.9 (N7CH_2_), 44.7 (NHCH_2_), 35.2 (NHCH_2_CH_2_), 29.7 (N1CH_3_), 29.2 (N3CH_3_), 28.4 (N7CH_2_CH_2_), 27.7 (N7CH_2_CH_2_CH_2_), 22.3 (N7CH_2_CH_2_CH_2_CH_2_), 14.4 (N7CH_2_CH_2_CH_2_ CH_2_CH_3_); IR ν (cm^−1^): 3398 (NH), 2953–2860 (alkyl), 1692 (CO), 1644 (CO), 870 (aryl); UPLC/MS purity 97.88%, *t*_R_ = 5.15, C_20_H_27_N_5_O_4_, MW 401.47, [M + H]^+^ 402.42. Anal. Elem.: C, 59.84; H, 6.78; N, 17.44; Found: C, 60.02; H, 6.55; N, 17.20.

##### 8-((3,4-Dihydroxyphenethyl)amino)-7-hexyl-1,3-dimethyl-3,7-dihydro-1*H*-purine-2,6-dione (**11i**)

Time: 3 h. Yield: 81%; crystallized from ethanol; mp: 216–218 °C; ^1^H NMR (300 MHz, DMSO-*d*_6_) δ ppm: 0.83 (t, *J* = 6.66 Hz, 3H, CH_2_CH_2_CH_2_CH_2_CH_2_CH_3_), 1.22 (m, 6H, CH_2_CH_2_CH_2_CH_2_CH_2_CH_3_), 1.54 (m, 2H, CH_2_CH_2_CH_2_CH_2_CH_2_CH_3_), 2.67 (t, *J* = 7.44Hz, 2H, CH_2_CH_2_), 3.15 (s, 3H, N1CH_3_); 3.35 (s, 3H, N3CH_3_); 3.39–3.45 (m, 2H, CH_2_CH_2_); 3.95 (t, *J* = 7.18 Hz, 2H,N7CH_2_CH_2_CH_2_CH_2_CH_2_CH_3_), 6.43 (dd, *J* = 7.95 Hz, Hz, 1H, C5H, phe); 6.58–6.62 (m, 2H, C2H, C6H, phe); 7.03 (bs, 1H,NH), 8.67 (bs, 2H, 2 × OH); ^13^C NMR (DMSO-*d*_6_) δ ppm: 153.7 (C4, phe), 153.0 (C6), 151.4 (C2), 149.0 (C4), 145.5 (C8), 144.0 (C3, phe), 130.6 (C1, phe), 119.8 (C2, phe), 116.5 (C5, phe), 115.9 (C6, phe), 101.9 (C5), 44.9 (N7CH_2_), 42.8 (NHCH_2_), 35.2 (NHCH_2_CH_2_), 31.3 (N7CH_2_CH_2_), 29.7 (N1CH_3_), 29.5 (N3CH_3_), 27.7 (N7CH_2_CH_2_CH_2_), 25.9 (N7CH_2_CH_2_CH_2_ CH_2_), 22.5 (N7CH_2_CH_2_CH_2_ CH_2_CH_2_), 14.3 (N7CH_2_CH_2_CH_2_ CH_2_CH_2_CH_3_); IR ν (cm^−1^): 3337 (NH), 1694 (CO), 1653 (CO); UPLC/MS purity 99.23%, *t*_R_ = 5.67, C_21_H_29_N_5_O_4_, MW 415.49, [M + H]^+^ 416.38. Anal. Elem.: C, 60.71; H, 7.04; N, 16.86; Found: C, 60.92; H, 7.06; N, 17.04.

##### 7-Benzyl-8-((3,4-dihydroxyphenethyl)amino)-1,3-dimethyl-3,7-dihydro-1*H*-purine-2,6-dione (**11j**)

Time: 3 h. Yield: 95%; crystallized from ethanol + H_2_O; mp: 214–216 °C; ^1^H NMR (300 MHz, DMSO-*d*_6_) δ ppm: 2.67 (t, *J* = 7.31 Hz, 2H, CH_2_CH_2_); 3.14 (s, 3H, N1CH_3_); 3.36 (s, 3H, N3CH_3_); 3.47 (q, *J* = 6.28 Hz, 2H, CH_2_CH_2_); 5.25 (s, 2H, N7CH_2_); 6.39 (dd, *J* = 7.95 Hz, 1H, C5H, phe); 6.59–6.61 (m, 2H, C2H, C6H, phe); 7.15–7.31 (m, 6H, NH + C2H, C3H, C4H, C5H, C6H, benzyl); 8.63 (s, 1H, 4′OH); 8.71 (s, 1H, 3′OH); ^13^C NMR (DMSO-*d*_6_) δ ppm: 153.1 (C4, phe), 153.2 (C6), 151.4 (C2), 149.3 (C4), 145.5 (C8), 144.0 (C3, phe), 137.5 (C1, benzyl), 130.5 (C1, phe), 128.9 (C3/C5, benzyl), 127.7 (C4, benzyl), 127.5 (C2/C6, benzyl), 119.8 (C2, phe), 116.5 (C5, phe), 115.9 (C6, phe-ethyl), 101.7 (C5), 45.7(N7CH_2_), 44.3 (NHCH_2_), 35.1 (NHCH_2_CH_2_), 29.8 (N1CH_3_), 27.7 (N3CH_3_); IR ν (cm^−1^): 3358 (NH), 1683 (CO), 1645 (CO); UPLC/MS purity 95.85%, *t*_R_ = 4.75, C_22_H_23_N_5_O_4_, MW 421.46, [M + H]^+^ 422.36. Anal. Elem.: C, 62.70; H, 5.50; N, 16.62; Found: C, 62.69; H, 5.56; N, 16.76.

##### 8-((3,4-Dihydroxyphenethyl)amino)-1,3-dimethyl-7-phenethyl-3,7-dihydro-1*H*-purine-2,6-dione (**11k**)

Time: 5 h. Yield: 84%; crystallized from ethanol + H_2_O; mp: 180–182 °C; ^1^H NMR (300 MHz, DMSO-*d*_6_) δ ppm: 2.62 (t, *J* = 7.44 Hz, 2H, NHCH_2_CH_2_); 2.84 (t, *J* = 7.82 Hz, NHCH_2_CH_2_); 3.18 (s, 3H, N1CH_3_); 3.36–3.42 (m, 5H, N3CH_3_ + N7CH_2_CH_2_); 4.18 (t, *J* = 7.67 Hz, 2H, N7CH_2_CH_2_); 6.45 (dd, *J* = 7.95 Hz, 1H, C5H, phe); 6.59–6.63 (m, 2H, C2H, C6H, phe); 7.08 (t, *J* = 5.64 Hz, 1H, NH); 7.17 (m, 5H, C2H, C3H, C4H, C5H, C6H, phe); 8.64 (s, 1H, 4′OH); 8.73 (s, 1H, 3′OH); ^13^C NMR (DMSO-*d*_6_) δ ppm: 153.7 (C4, phe), 153.2 (C6), 151.5 (C2), 149.2 (C4), 145.6 (C8), 144.0 (C3, phe), 138.4 (C1, phe-ethyl), 130.6 (C1, phe), 129.4 (C3/C5, phe-ethyl), 128.7 (C2/C6, phe-ethyl), 126.9 (C4, phe-ethyl), 119.8 (C2, phe), 116.5 (C5, phe), 115.9 (C6, phe), 101.6 (C5), 44.9 (N7CH_2_), 44.1 (NHCH_2_), 35.6 (N7CH_2_CH_2_), 35.2 (NHCH_2_CH_2_), 29.8 (N_1_CH_3_), 27.7 (N3CH_3_); IR ν (cm^−1^): 3386 (NH), 1691 (CO), 1630 (CO); UPLC/MS purity 99.44%, *t*_R_ = 5.07, C_23_H_25_N_5_O_4_, MW 435.48, [M + H]^+^ 436.39. Anal. Elem.: C, 63.44; H, 5.79; N, 16.08; Found: C, 63.69; H, 5.81; N, 16.14.

##### 8-((3,4-Dihydroxyphenethyl)amino)-1,3-dimethyl-7-(2-phenoxyethyl)-3,7-dihydro-1*H*-purine-2,6-dione (**11l**)

Time: 4 h. Yield: 85%; crystallized from butanol; mp: 218–220 °C; ^1^H NMR (300 MHz, DMSO-*d*_6_) δ ppm: 2.71 (t, *J* = 8.07 Hz, 2H, CH_2_CH_2_); 3.16 (s, 3H, N1CH_3_); 3.32 (s, 3H, N3CH_3_); 3.46 (q, *J* = 6.16 Hz, 2H, CH_2_CH_2_); 4.14 (t, *J* = 5.90 Hz, 2H, N7CH_2_CH_2_O); 4.37 (t, *J* = 5.77 Hz, 2H, N7CH_2_CH_2_O); 6.47 (dd, J = 7.95 Hz, 1H, C5H, phe); 6.62 (d, *J* = 7.95 Hz, 2H, C2H, C6H, phe); 6.81–6.92 (m, 3H, C3H, C4H, C5H, phenoxy); 7.15–7.24 (m, 3H, NH + C2H, C6H, phenoxy); 8.65 (s, 1H, 4′OH); 8.74 (s, 1H, 3′OH); ^13^C NMR (DMSO-*d*_6_) δ ppm: 158.4 (C1, phe), 154.5 (C4), 153.2 (C6), 151.4 (C2), 149.2 (C4), 145.6 (C3, phe), 144.1 (C4, phenoxy), 130.5 (C1, phe), 130.0 (C3/C5, phenoxy), 121.3 (C2, phe), 116.5 (C5, phe), 116.0 (C5, phe), 114.8 (C2/C6, phenoxy), 101.8 (C5), 66.2 (CH_2_O), 44.9 (N7CH_2_), 42.4 (NHCH_2_), 35.2 (NHCH_2_CH_2_), 29.7 (N1CH_3_), 27.7 (N3CH_3_); IR ν (cm^−1^): 3383 (NH), 1693 (CO), 1642 (CO); UPLC/MS purity 98.33%, *t*_R_ = 5.09, C_23_H_25_N_5_O_5_, MW 451.48, [M + H]^+^ 452.41. Anal. Elem.: C, 61.19; H, 5.58; N, 15.51; Found: C, 60.79; H, 5.86; N, 15.22.

##### 8-((3,4-Dihydroxyphenethyl)amino)-1,3,7-tripropyl-3,7-dihydro-1*H*-purine-2,6-dione (**12**)

Time: 3 h. Yield: 33%; crystallized from methanol + H_2_O; mp: 106–108 °C; ^1^H NMR (300 MHz, DMSO-*d*_6_) δ ppm: 0.79–0.87 (m, 9H, 3 × CH_2_CH_2_CH_2_CH_3_), 1.44–1.72 (m, 6H, 3 × CH_2_CH_2_CH_3_), 2.67 (t, *J* = 7.57 Hz 2H, CH_2_CH_2_), 3.30–3.42 (m, 2H, CH_2_CH_2_), 3.76 (t, *J* = 7.23 Hz, 2H, N7CH_2_); 3.85–3.93 (m, 2H, N3CH_2,_ N1CH_2_), 6.43 (dd, *J* = 7.95 Hz, 1H, C5H, phe), 6.57–6.62 (m, 2H, C2H, C6H, phe), 7.84 (bs, 1H, NH), 8.60 (bs, 2H, 2 × OH); ^13^C NMR (DMSO-*d*_6_) δ ppm: 153.8 (C4, phe), 152.9 (C6), 151.0 (C2), 148.9 (C8), 145.5 (C3, phe), 130.6 (C1, phe), 119.7 (C2, phe), 116.6 (C5, phe), 115.9 (C6, phe), 101.9 (C5), 45.0 (N7CH_2_), 44.3 (N1CH_2_), 44.1 (N3CH_2_), 41.9 (NHCH_2_), 35.2 (NHCH_2_CH_2_), 22.9 (N7CH_2_CH_2_), 21.5 (N1CH_2_CH_2_), 21.4 (N3CH_2_CH_2_), 11.6 (N3CH_2_CH_2_CH_3_), 11.6 (N1CH_2_CH_2_CH_3_), 11.0 (N7CH_2_CH_2_CH_3_); IR ν (cm^−1^): 3438 (NH), 1681 (CO), 1638 (CO); UPLC/MS purity 97.39%, *t*_R_ = 6.18, C_22_H_31_N_5_O_4_, MW 429.52, [M + H]^+^ 430.40. Anal. Elem.: C, 61.52; H, 7.28; N, 16.31; Found: C, 58.95; H, 7.12; N, 15.69.

##### 1,3-Dibutyl-8-((3,4-dihydroxyphenethyl)amino)-7-propyl-3,7-dihydro-1*H*-purine-2,6-dione (**13**)

Time: 3 h. Yield: 74%; crystallized from ethanol + H_2_O; mp: 112–114 °C; ^1^H NMR (300 MHz, DMSO-*d*_6_) δ ppm: 0.71–0.91 (m, 9H, CH_2_CH_2_CH_3_ + 2 × CH_2_CH_2_CH_2_CH_3_), 1.18–1.34 (m, 4H, 2 × CH_2_CH_2_CH_2_CH_3_), 1.41–1.49 (m, 2H, CH_2_CH_2_CH_3_), 1.51–1.67 (m, 4H, 2 × CH_2_CH_2_CH_2_CH_3_), 2.67 (t, *J* = 7.44 Hz, 2H, CH_2_CH_2_), 3.31–3.39 (m, 2H, CH_2_CH_2_), 3.78 (t, *J* = 6.81 Hz, 2H, N7CH_2_), 3.88–3.94 (m, 2H, N3CH_2,_ N1CH_2_), 6.42 (d, *J* = 7.95 Hz, 1H, C5H, phe), 6.56–6.62 (m, 2H, C2H, C6H, phe), 7.07 (s, 1H, NH), 8.68 (s, 2H, 2 × OH); ^13^C NMR (DMSO-*d*_6_) δ ppm: 153.8 (C4, phe), 152.9 (C6), 150.9 (C2), 148.8 (C4), 145.5 (C8), 143.6 (C3, phe), 130.6 (C1, phe), 119.7 (C2, phe), 116.6 (C5, phe), 115.9 (C5, phe), 102.0 (C5), 44.9 (N7CH_2_), 44.1 (N1CH_2_), 42.4 (NHCH_2_), 35.2 (NHCH_2_CH_2_), 30.3 (N1CH_2_CH_2_), 30.1 (N3CH_2_CH_2_), 22.9 (N7CH_2_CH_2_), 20.1 (N1CH_2_CH_2_CH_2_), 19.8 (N3CH_2_CH_2_CH_2_), 14.2 (N3CH_2_CH_2_CH_2_CH_3_), 14.1 (N1CH_2_CH_2_CH_2_CH_3_), 11.0 (N7CH_2_CH_2_CH_3_); IR ν (cm^−1^): 3345 (NH), 1682 (CO), 1638 (CO); UPLC/MS purity 96.06%, *t*_R_ = 7.24, C_24_H_35_N_5_O_4_, MW 457.58, [M + H]^+^ 458.24. Anal. Elem.: C, 63.00; H, 7.71; N, 15.31; Found: C, 62.89; H, 7.54; N, 15.02.

#### 4.1.4. General Procedure for the Synthesis of N-9,10-3′,4′-dihydroxyphenylethyl- Substituted 1,3-dialkyl-6,7,8,9-tetrahydropyrimido-/-6,7,8,9-tetrahydro(10*H*)-1,3-diazepino [2,1-f]purine-2,4(1*H*,3*H*)-diones (**20a**–**b**, **21**, **22**)

A mixture of 1 mmol of *N*-9,10-3′,4′-dimethoxyphenylethyl-1,3-dialkyl-6,7,8,9-tetrahydropyrimido-, -6,7,8,9-tetrahydro(10*H*)-1,3-diazepino[2,1-*f*]purine-2,4(1*H*,3*H*)-diones, and 5 mL of 40% HBr was refluxed for 3–5 h. Compounds were obtained after cooling and alkalization by 20% Na_2_CO_3_ to pH 8–9, washing by water, filtered off, and dried. All compounds were purified by recrystallization from ethanol.

##### 9-(3,4-Dihydroxyphenethyl)-1,3-dimethyl-6,7,8,9-tetrahydropyrimido[2,1-*f*]purine-2,4(1*H*,3*H*)-dione (**20a**)

Yield: 71%; mp: 213–215 °C; ^1^H NMR (300 MHz, DMSO-*d*_6_) δ ppm: 1.99–2.09 (m, 2H, CH_2_CH_2_CH_2_); 2.70 (t, *J* = 7.40 Hz, 2H, CH_2_CH_2_); 3.17 (s, 3H, N3CH_3_); 3.26 (t, *J* = 5.52 Hz, 2H, CH_2_CH_2_); 3.33 (s, 3H, N1CH_3_); 3.59, *J* = 7.45 Hz, 2H, CH_2_CH_2_CH_2_); 4.03 (t, *J* = 5.93 Hz, 2H, CH_2_CH_2_CH_2_); 6.46–6.49 (dd, *J* = 7.95 Hz, 1H, C5H, phe); 6.61–6.65 (m, 2H, C2H, C6H, phe); 8.65 (s, 1H, 4′OH); 8.74 (s, 1H, 3′OH); ^13^C NMR (DMSO-*d*_6_) δ ppm: 153.0 (C4Phe); 151.7 (C4); 151.5 (C_2_); 148.8 (C9a); 145.6 (C10a); 144.1 (C3, phe); 130.2 (C1, phe); 119.8 (C2, phe); 116.6 (C5, phe); 116.0 (C6, phe); 102.8 (C4a); 51.4 (C_8_); 44. 8 (N9CH_2_); 41.8 (C6); 32.9 (N9CH_2_CH_2_); 29.8 (N3CH_3_); 27.6 (N1CH_3_); 21.2 (C7); IR ν (cm^−1^): 3365 (OH), 1687 (CO), 1622 (CO); UV λ_max_ (nm): 302; UPLC/MS purity 100.00%, *t*_R_ = 3.76, C_18_H_21_N_5_O_4_, MW 371,40, [M + H]^+^ 372.30. Anal. for C_18_H_21_N_5_O_4_: Calcd: C, 61.00; H, 6.58; N, 16.94. Found: C, 60.85; H, 6.91; N, 16.91.

##### 10-(3,4-Dihydroxyphenethyl)-1,3-dimethyl-7,8,9,10-tetrahydro-1*H*-[1,3]diazepino[2,1-*f*]purine-2,4(3*H*,6*H*)-dione (**20b**)

Yield: 91%; mp: 232–235 °C; ^1^H NMR (300 MHz, DMSO-*d*_6_) δ ppm: 1.73–2.05 (m, 4H, CH_2_CH_2_CH_2_CH_2_); 2.72 (t, *J* = 7.44 Hz, 2H, N10CH_2_CH_2_); 3.15 (s, 3H, N3CH_3_); 3.28 (d, *J* = 5.64 Hz, 2H, N10CH_2_CH_2_); 3.37 (s, 3H, N1CH_3_); 3.54 (t, *J* = 7.44 Hz, 2H, CH_2_CH_2_CH_2_CH); 4.13 (d, *J* = 5.64 Hz, 2H, CH_2_CH_2_CH_2_CH_2_); 6.47 (dd, *J* = 8.20 Hz, *J* = 7.95 Hz, 1H, C5H, phe); 6.60–6.63 (m, 2H, C2H, C6H, phe); 8.68 (s, 2H, 2OH); ^13^C NMR (DMSO-*d*_6_) δ ppm: 159.0 (C4, phe); 153.7 (C4); 151.4 (C2); 148.2 (C9a); 145.5 (C10a); 144.0 (C3, phe); 130.5 (C1, phe); 119.9 (C2, phe); 116.6 (C5, phe); 116.0 (C6, phe); 103.5 (C4a); 55.1 (C9); 51.6 (C8); 45.8 (N10CH_2_); 40.6 (C6); 33.2 (N10CH_2_CH_2_); 29.7 (N3CH_3_); 27.7 (N1CH_3_); 26.3 (C7); IR ν (cm^−1^): 3239 (OH), 1695 (CO), 1634 (CO); UV λ_max_ (nm): 300; UPLC/MS purity 100.00%, *t*_R_ = 4.43, C_19_H_23_N_5_O_4_, MW 385,42, [M + H]^+^ 386.33. Anal. for C_19_H_23_N_5_O_4_: Calcd: C, 61.00; H, 6.58; N, 16.94. Found: C, 60.85; H, 6.91; N, 16.91.

##### 9-(3,4-Dihydroxyphenethyl)-1,3-dipropyl-6,7,8,9-tetrahydropyrimido[2,1-*f*]purine-2,4(1*H*,3*H*)-dione (**21**)

Yield: 94%; mp: 206–208 °C; ^1^H NMR (300 MHz, DMSO-*d*_6_) δ ppm: 0.80–0.88 (m, 6H, 2 × CH_2_CH_2_CH_3_), 1.45–1.55 (m, 2H, N3CH_2_CH_2_CH_3_); 1.61–1.73 (m, 2H, N1CH_2_CH_2_CH_3_); 1.96–1.99 (m, 2H, CH_2_CH_2_CH_2_); 2.68 (t, *J* = 7.31 Hz, 2H, CH_2_CH_2_); 3.24 (t, *J* = 5.39 Hz, CH_2_CH_2_); 3.55 (t, *J* = 7.31 Hz, 2H, CH_2_CH_2_CH_2_); 3.76 (t, *J* = 7.44 Hz, 2H, N3CH_2_); 3.89 (t, *J* = 6.9 Hz, 2H, N1CH_2_); 4.01 (t, *J* = 5.27 Hz, 2H, CH_2_CH_2_CH_2_); 6.45 (dd, *J* = 7.95 Hz, *J* = 7.95 Hz, 1H, C5H, phe); 6.58–6.63, (m, 2H, C2H, C6H, phe); 8.68 (s, 2H, 2OH); ^13^C NMR (DMSO-*d*_6_) δ ppm: 152.9 (C4, phe); 151.7 (C4); 151.0 (C2); 148.7 (C9a); 145.6 (C10a); 144.0 (C3, phe); 130.3 (C1, phe); 119.7 (C2, phe); 116.6 (C5, phe); 116.0 (C6, phe); 102.2 (C4a); 51.5 (C8); 44.8 (N9CH_2_); 44.3 (N1CH_2_); 41.7 (N3CH_2_); 41.9 (C6); 32.8 (N9CH_2_CH_2_); 21.4 (C7); 21.3 (C7); 20.3 (N1CH_2_CH_2_); 21.2 (N3CH_2_CH_2_); 11.6 (N3CH_2_CH_2_CH_3_); 11.5 (N1CH_2_CH_2_CH_3_); IR ν (cm^−1^): 3393 (OH), 1685 (CO), 1645 (CO); UV λ_max_ (nm): 302; UPLC/MS purity 99.40%, *t*_R_ = 5.64, C_22_H_29_N_5_O_4_, MW 427,51, [M + H]^+^ 428.41. Anal. for C_22_H_29_N_5_O_4_: Calcd: C, 61.00; H, 6.58; N, 16.94. Found: C, 60.85; H, 6.91; N, 16.91.

##### 1,3-Dibutyl-9-(3,4-dihydroxyphenethyl)-6,7,8,9-tetrahydropyrimido[2,1-*f*]purine-2,4(1*H*,3*H*)-dione (**22**)

Yield: 97%; mp: 194–196 °C; ^1^H NMR (300 MHz, DMSO-*d*_6_) δ ppm: 0.85–0.92 (m, 6H, 2 × CH_2_CH_2_CH_2_CH_3_); 1.19–1.34 (m, 4H, 2 × CH_2_CH_2_CH_2_CH_3_); 1.42–1.52 (m, 2H, N3CH_2_CH_2_); 1.58–1.62 (m, 2H, N1CH_2_CH_2_); 1.98 (m, 2H, CH_2_CH_2_CH_2_); 2.68 (t, *J* = 7.31 Hz, 2H, CH_2_CH_2_); 3.25 (t, *J* = 5.39 Hz, 2H, CH_2_CH_2_); 3.55 (t, *J* = 7.31 Hz, 2H, CH_2_CH_2_CH_2_); 3.79 (t, *J* = 7.31 Hz, 2H, N3CH_2_); 3.92 (t, *J* = 6.92 Hz, 2H, N_1_CH_2_); 4.01 (t, *J* = 5.77 Hz, 2H, CH_2_CH_2_CH_2_); 6.45 (dd, *J* = 8,21 Hz, *J* = 7.95 Hz, 1H, C5H, phe); 6.58–6.63 (m, 2H, C2H, C6H, phe); 8.70 (s, 2H, 2OH); ^13^C NMR (DMSO-*d*_6_) δ ppm: 158.9 (C4, phe); 151.7 (C4); 150.9 (C2); 148.7 (C9a); 145.6; (C10a); 144.0 (C3, phe); 130.3 (C1, phe); 119.71 (C2, phe); 116.59 (C5, phe); 115.97 (C6, phe); 102.26 (C4a); 51.51 (C8); 44.81 (N9CH_2_); 42.4 (N1CH_2_); 41.7 (N3CH_2_); 40.6 (C6); 32.8 (N9CH_2_CH_2_); 30.3 (N1CH_2_CH_2_); 30.1 (N1CH_2_CH_2_); 21.2 (C7); 20.1 (N1CH_2_CH_2_CH_2_); 19.8 (N3CH_2_CH_2_CH_2_); 14.2 (N3CH_2_CH_2_CH_2_CH_3_); 10.1 (N1CH_2_CH_2_CH_2_CH_3_); IR ν (cm^−1^): 3282 (OH), 1684 (CO), 1624 (CO); UV λ_max_ (nm): 302; UPLC/MS purity 98.91%, *t*_R_ = 6.73, C_24_H_33_N_5_O_4_, MW 455.54, [M + H]^+^ 456.46. Anal. for C_24_H_33_N_5_O_4_: Calcd: C, 64.58; H, 7.72; N, 14.48. Found: C, 64.98; H, 7.71; N, 14.39.

### 4.2. Biological Experiments

#### 4.2.1. Monoamine Oxidase Assays

Inhibition activity of compounds at MAO enzyme isoforms was measured via a fluorometric method using the Amplex Red Monoamine Oxidase Assay (ThermoFisher Scientific, Waltham, MA, USA #A12214) in a 96-well plates. Human recombinant MAO-B and MAO-A enzymes (Sigma Aldrich, St. Louis, MO, USA #M7441 and #M7316) were used. The assays were conducted as previously described [51].

#### 4.2.2. Phosphodiesterase Inhibition

Tested and reference compounds were dissolved in dimethyl sulfoxide (DMSO) at a concentration of 10 mM and further diluted in assay buffer (10 mM Tris-HCl, 10 mM magnesium chloride and 0.05% Tween-20; pH 7.4). Inhibition of human PDE10A and 4B1 was measured using PDElight HTS cAMP phosphodiesterase assay kit (Lonza, Basel, Switzerland) according to manufacturer’s recommendations. Then, 10 ng of PDE10A and 5 ng of PDE4B1 (BPS Biosciences, San Diego, CA, USA) in appropriate buffer was incubated with reference and tested compound for 20 min. After incubation, the cAMP substrate (final concentration 1.25 µM for PDE10A and 5 µM for PDE4B1) was added and incubated for 1 h. Then PDELight AMP Detection Reagent was added and incubated 10 min. All reactions were carried out at 37 °C in 96 white-walled, half-area-well plates (PerkinElmer). Luminescence was measured in a multifunction plate reader (POLARstar Omega, BMG Labtech, Ortenberg, Germany). The percentage of inhibition and *IC*_50_s were computed using GraphPad Prism Version 6.0 software.

#### 4.2.3. Radioligand Binding Assays at Adenosine Receptors

Adenosine receptor radioligand binding assays were performed as previously described [61] using rat brain cortical membrane preparations for A_1_ and rat brain striatal membrane preparations for A_2A_ AR assays. Frozen rat brains (unstripped) were obtained from Pel Freez, Rogers, AR, USA. For assays at human AR subtypes, cell membranes of CHO cells recombinantly expressing the respective receptor were used. The following compounds were employed as radioligands: A_1_: [^3^H]2-chloro-N^6^-cyclopentyladenosine ([^3^H]CCPA) [62]; A_2A_: [^3^H]3-(3-hydroxypropyl)-7-methyl-8-(*m*-methoxystyryl)-1-propargylxanthine ([^3^H]MSX-2) [63]; A_2B_: [^3^H]8-(4-(4-(4-chlorophenyl)piperazine-1-sulfonyl)phenyl)-1-propylxanthine ([^3^H]PSB-603) [64]; A_3_: [^3^H]phenyl-8-ethyl-4-methyl-(8*R*)-4,5,7,8-tetrahydro-1*H*-imidazo[2,1-*i*]purine-5-one ([^3^H]PSB-11) [65]. Initially, a single high concentration of compound was tested (1 µM). For potent compounds, full concentration–inhibition curves were determined using six to ten different concentrations of test compound spanning three orders of magnitude. At least three independent experiments were performed. Data were analyzed using GraphPad PRISM program version 4.0 or higher (Graph Pad, San Diego, CA, USA).

#### 4.2.4. Radioligand Binding Assays at Dopamine Receptors

**Dopamine D_1_ receptors**

Rat striatum tissue was thawed in 50 volumes of ice-cold 50 mM potassium phosphate buffer, pH 7.4, homogenized and centrifuged at 20,000× *g* for 20 min. The resulting pellet was resuspended in the same quantity of the buffer and centrifuged again for 20 min. Binding experiments were conducted in 96-well microplates in a total volume of 250 μL of appropriate buffers. Reaction mix included 50 μL solution of test compound, 50 μL of radioligand, and 150 μL of tissue suspension (3 mg/mL). [^3^H]SCH23390 (spec. act. 81.9 Ci/mmol, PerkinElmer, NET 930) was used for labeling the D_1_-receptor. For measuring unspecific binding, cis(Z)flupentixol—5 μM was applied. Samples were incubated at 30 °C for 60 min. The incubation was terminated by rapid filtration over glass fiber filters GF/B using a Harvester (PerkinElmer, USA). The radioactivity was measured in MicroBeta TriLux 1450—liquid scintillation counter (PerkinElmer). Each compound was tested in a screening assay in duplicate at final concentrations of 1 µM. Results were expressed as a percent of inhibition of specific binding.

**Dopamine D_2_ receptors**

HEK293 cells with stable expression of human D_2L_ receptor were maintained at 37 °C in a humidified atmosphere with 5% CO_2_ and grown in Dulbecco’s Modifier Eagle Medium containing 10% dialyzed fetal bovine serum and 500 µg/mL G418 sulfate. For membrane preparation, cells were subcultured in 150 cm^2^ flasks, grown to 90% confluence, washed twice with prewarmed to 37 °C phosphate buffered saline (PBS), and pelleted by centrifugation (200× *g*) in PBS containing 0.1 mM EDTA and 1 mM dithiothreitol. Prior to membrane preparation, pellets were stored at −80 °C.

Cell pellets were thawed and homogenized in 10 volumes of assay buffer using an Ultra Turrax tissue homogenizer and centrifuged twice at 35,000× *g* for 15 min at 4 °C. The incubation buffer consisted of 50 mM Tris-HCl, 1 mM EDTA, 4 mM MgCl_2_, 120 mM NaCl, 5 mM KCl, 1.5 mM CaCl_2_, and 0.1% ascorbate. Assay was incubated in a total volume of 200 µL in 96-well microtiter plate for 1 h at 37 °C. The process of equilibration was terminated by rapid filtration through Unifilter plate with a 96-well cell harvester and radioactivity retained on the filter was quantified on a Microbeta plate reader (PerkinElmer). For competition studies, the assay samples contained radioligand 2.5 nM [^3^H]-raclopride (PerkinElmer). Non-specific binding was defined with 10 µM of haloperidol. Each compound was tested in triplicate at 7 concentrations (10^−10^–10^−4^ M). The inhibition constants (*K*_i_) were calculated from the Cheng–Prusoff equation [66]. Results were expressed as means of at least two separate experiments.

#### 4.2.5. cAMP Accumulation Assay in Cells Expressing hD_2_R

Intrinsic activity of compound 13 and natural agonist—dopamine—was determined in cAMP accumulation assay. Briefly, HEK293 cells stably expressing human D_2L_R were incubated (30 min, room temperature) with forskolin (1 μM) and evaluated compounds in triplicates (7 appropriate concentrations in a range spanning over 6 log units) in presence of phosphodiesterase inhibitor (RO-201724, 100 μM). Subsequently, intracellular cAMP accumulation was measured with homogenous TR-FRET immunoassay, using LANCE Ultra cAMP kit (PerkinElmer) and following manufacturer’s instructions. TR-FRET signal, inversely proportional to cAMP concentration in sample, was measured using Infinite M1000 Pro microplate reader (Tecan, Männedorf, Switzerland). Sigmoidal dose–response curve fitting was performed, using GraphPad Prism™ software (version 5.01, San Diego, CA, USA).

#### 4.2.6. Determination of the Antioxidant Activity

Antioxidant properties of considered structures were evaluated in DPPH test. The assay utilizes a stable free radical 2,2-diphenyl-1-picrylhydrazyl (DPPH). DPPH has a deep violet color in solution and strong absorption band at 520 nm. The antioxidant activity of the tested compounds was defined by their ability to induce the formation of the reduced form of DPPH. Upon reduction, DPPH color turned pale yellow, which manifested by decreased sample absorption at 520 nm and was measured in conducted experiments using EnSpire microplate reader (PerkinElmer). Antioxidant activity of xanthine derivatives and reference compounds was tested in triplicate at 50 µM concentration and normalized to the activity of reference radical scavenger—quercetin.

#### 4.2.7. Neuroprotection Studies

Neuroblastoma SH-SY5Y cells were seeded at the density of 20,000 cells per well to 96-well plates one day before the experiment. Twelve hours prior to start of the assay, cell culture medium was replaced with the fresh portion of reduced serum (1%) medium. Tested compounds were added to the cells at the final concentration of 10 or 50 µM in 1% DMSO. In the neuroprotection studies, hydrogen peroxide, at a final concertation of 300 µM, was pipetted to the assay plate 1 h after tested compounds addition. Cell death, represented by the release of LDH through the perforated cell membrane, was measured following 14 h incubation of cells with the compounds and hydrogen peroxide. LDH levels in cell culture medium were determined using fluorometric CytoTox-ONE Homogeneous Membrane Integrity Assay (Promega Corporation, Madison, WI, USA), according to manufacturer’s instructions.

## Figures and Tables

**Table 1:** Inhibitory potencies of the xanthine–dopamine hybrid compounds in human MAO-B and MAO-A ^a^.

Compd	R^2^	MAO-B *IC*_50_ ± SEM (nM)	MAO-A *IC*_50_ ± SEM (nM) (or % Inhibition ± SEM at 1 µM)
Rasagiline	-	0.019	-
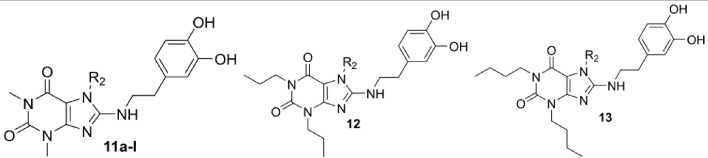			
**11a**	methyl	**50.7** ± 10.0	>1000 (44 ± 1)
**11b**	ethyl	**47.9** ± 8.4	**891** ± 63
**11c**	*n*-propyl	**165** ± 30	**>1000** (33 ± 10)
**11d**	*iso*-propyl	**126** ± 49	**≥1000** (47 ± 1)
**11e**	*n*-butyl	**43.8** ± 16.2	**>1000** (38 ± 1)
**11f**	*iso*-butyl	**205** ± 23	**>1000** (42 ± 1)
**11g**	*sec*-butyl	**197** ± 57	**>1000** (44 ± 1)
**11h**	*n*-pentyl	**58.3** ± 6.2	**692** ± 7
**11i**	*n*-hexyl	**76.4** ± 0.7	**246** ± 72
**11j**	benzyl	**45.6** ± 12.9	**576** ± 68
**11k**	phenethyl	**73.5** ± 7.5	**256** ± 93
**11l**	phenoxyethyl	**53.5** ± 18.2	**>1000** (41 ± 3)
**12**	*n*-propyl	**105** ± 23	**ca. 1000** (57 ± 0)
**13**	*n*-butyl	**81.8** ± 19.7	**>1000** (36 ± 1)
** 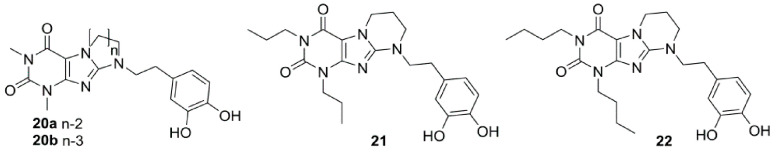 **			
**20a**	-	**121** ± 23	**>1000** (29 ± 1)
**20b**	-	**101** ± 21	**>1000** (44 ± 4)
**21**	-	**157** ± 39	**ca. 1000** (61 ± 1)
**22**	-	**170** ± 30	**>1000** (43 ± 1)

^a^ *IC*_50_ values are highlighted in bold.

**Table 2:** Affinity of hybrid compounds at adenosine and dopamine receptors.

Compd	*r*A_1_ [^3^H]CCPA	*r*A_2A_ [^3^H]MSX-2	*h*A_2B_ [^3^H]PSB-603	*h*A_3_ [^3^H]PSB-11	*r*D_1_ [^3^H]SCH23390	*h*D_2_ [^3^H]-Raclopride
	***K*_i_ ± SEM (µM) ^a^** **(or % inhibition ± SEM at 1 µM for A_2B_, A_3_ and D_1_ or at 10 µM for A_1_ and A_2A_)**					
**11a**	1.98 ± 0.29	**0.370** ± 0.063	>1.0 (20 ± 4)	>1.0 (−1 ± 3)	>1.0 (13 ± 2)	25.2 ± 5.97
**11b**	**0.479** ± 0.095	**0.672** ± 0.083	>1.0 (−2 ± 3)	>1.0 (−5 ± 3)	>1.0 (15 ± 2)	16.2 ± 1.23
**11c**	**0.730** ± 0.093	4.72 ± 0.93	>1.0 (6 ± 1)	>1.0 (5 ± 3)	>1.0 (9 ± 2)	9.37 ± 1.15
**11d**	**0.857** ± 0.043	1.15 ± 0.14	>1.0 (13 ± 8)	>1.0 (12 ± 2)	>1.0 (15 ± 4)	17.7 ± 3.99
**11e**	**0.304** ± 0.066	1.70 ± 0.26	>1.0 (14 ± 4)	>1.0 (0 ± 2)	>1.0 (13 ± 2)	12.7 ± 1.86
**11f**	1.13 ± 0.18	6.86 ± 0.77	>1.0 (1 ± 0)	>1.0 (−6 ± 2)	>1.0 (15 ± 6)	27.6 ± 4.0
**11g**	1.17 ± 0.38	8.98 ± 0.15	>1.0 (−5 ± 1)	>1.0 (−4 ± 12)	>1.0 (11 ± 3)	39.2 ± 8.43
**11h**	**0.191** ± 0.030	2.22 ± 0.26	>1.0 (12 ± 8)	>1.0 (0 ± 2)	>1.0 (12 ± 5)	8.26 ± 0.48
**11i**	>10.0 (43 ± 3)	>10.0 (14 ± 0)	>1.0 (31 ± 7)	>1.0 (3 ± 0)	>1.0 (9 ± 4)	6.68 ± 0.95
**11j**	**0.455** ± 0.077	3.60 ± 0.29	>1.0 (31 ± 1)	>1.0 (7 ± 1)	>1.0 (3 ± 2)	9.10 ± 1.65
**11k**	≥10.0 (46 ± 1)	≥10.0 (44 ± 1)	>1.0 (−9 ± 0)	>1.0 (−1 ± 2)	>1.0 (6 ± 2)	9.95 ± 1.95
**11l**	**0.124** ± 0.022	1.38 ± 0.12	>1.0 (22 ± 4)	>1.0 (−2 ± 1)	>1.0 (3 ± 2)	8.87 ± 0.94
**12**	**0.130** ± 0.031	**0.833** ± 0.081	>1.0 (34 ± 3)	>1.0 (25 ± 2)	>1.0 (9 ± 2)	13.3 ± 2.2
**13**	**0.100** ± 0.015	1.32 ± 0.25	>1.0 (31 ± 7)	>1.0 (17 ± 1)	>1.0 (2 ± 4)	4.39 ± 0.43
**20a**	≥10.0 (43 ± 5)	**0.507** ± 0.109	>1.0 (13 ± 1)	>1.0 (−6 ± 2)	>1.0 (15 ± 1)	6.68 ± 0.87
**20b**	1.05 ± 0.17	**0.234** ± 0.055	>1.0 (6 ± 2)	>1.0 (−2 ± 5)	>1.0 (15 ± 1)	10.7 ± 1.40
**21**	**0.070** ± 0.005	**0.274** ± 0.081	**0.371** ± 0.023	>1.0 (26 ± 3)	>1.0 (12 ± 2)	12.7 ± 2.8
**22**	**0.053** ± 0.008	**0.334** ± 0.052	**0.175** ± 0.021	**0.676** ± 0.143	>1.0 (11 ± 5)	16.6 ± 2.3

^a^ Submicromolar affinities are highlighted in bold.

**Table 3:** Inhibitory potencies of final structures at human PDE4B1 and PDE10A1 ^a^.

Compd	PDE 4B1 *IC*_50_ ± SEM (µM) (or % Inhibition ± SEM at 10 µM)	PDE 10A1 *IC*_50_ ± SEM (µM) (or % Inhibition ± SEM at 10 µM)
**rolipram**	0.17 [53]	
**papaverine**	-	0.1 [54]
**11a**	**7.85** ± 1.85	**15.1** ± 3.1
**11b**	**2.52** ± 0.14	**4.63** ± 1.25
**11c**	**3.32** ± 0.80	**3.45** ± 0.27
**11d**	**2.44** ± 0.80	**2.30** ± 0.64
**11e**	**3.16** ± 0.53	**3.77** ± 1.29
**11f**	**3.76** ± 0.16	**≥10.0** (59 ± 3)
**11g**	**4.41** ± 0.23	**10.2** ± 0.9
**11h**	**4.43** ± 0.25	**≥10.0** (51 ± 6)
**11i**	**>10.0** (42 ± 1)	**>10.0** (14 ± 6)
**11j**	**3.29** ± 0.54	**≥10.0** (36 ± 3)
**11k**	**4.40** ± 0.54	**2.41** ± 0.07
**11l**	**>10.0** (20 ± 1)	**>10.0** (17 ± 2)
**12**	**14.5** ± 0.48	**2.73** ± 0.03
**13**	**>10.0** (28 ± 1)	**>10.0** (26 ± 5)
**20a**	**>10.0** (44 ± 1)	**>10.0** (35 ± 3)
**20b**	**>10.0** (45 ± 1)	**30.7** ± 0.2
**21**	**16.5** ± 0.32	**31.0** ± 0.1
**22**	**>10.0** (24 ± 2)	**>10.0** (25 ± 4)

^a^ *IC*_50_ values are highlighted in bold.

**Table 4:** Antioxidative activity determined in a DPPH assay.

Studied Compound [50 µM]	Antioxidative Activity [% of Quercetin Response ^a^ at 50 µM ± SD]
**11a**	**79** ± 1
**11b**	**75** ± 1
**11c**	**69** ± 1
**11d**	**87** ± 1
**11e**	**75** ± 1
**11f**	**57** ± 1
**11g**	**71** ± 1
**11h**	**79** ± 1
**11i**	**26** ± 1
**11j**	**82** ± 1
**11k**	**73** ± 3
**11l**	**26** ± 3
**12**	**86** ± 3
**13**	**61** ± 1
**20a**	**68** ± 2
**20b**	**79** ± 1
**21**	**13** ± 1
**22**	**26** ± 2
**Ascorbic acid**	**78** ± 1
**Trolox**	**73** ± 1

^a^ highlighted in bold.

## Data Availability

Data sharing is not applicable to this article. New data created or analyzed in this study are available in the manuscript or supplementary materials.

## Supplementary Materials

The following supporting information can be downloaded at: https://www.mdpi.com/article/10.3390/biom13071079/s1.
