# Supplementary material for: Xanthine–Dopamine Hybrid Molecules as Multitarget Drugs with Potential for the Treatment of Neurodegenerative Diseases
Source: Biomolecules. 2023 Jul 5;13(7):1079. doi: 10.3390/biom13071079 (PMC10377586; doi:10.3390/biom13071079)

Supplementary material

Xanthine-dopamine hybrid molecules as multitarget drugs with potential for the treatment of neurodegenerative diseases

[1#]Michał Załuski, [1#]Tadeusz Karcz, [1]Anna Drabczyńska, [2]Christin Vielmuth, [1]Agnieszka Olejarz-Maciej, [3]Monika Głuch-Lutwin, [3]Barbara Mordyl, [3]Agata Siwek, [4]Grzegorz Satała, [2]Christa E. Müller, [1]Katarzyna Kieć-Kononowicz

[1]Department of Technology and Biotechnology of Drugs, Faculty of Pharmacy, Jagiellonian University Medical College, Kraków, Poland

[2]PharmaCenter Bonn, Pharmaceutical Institute, Pharmaceutical & Medicinal Chemistry, University of Bonn, Bonn, Germany

[3]Department of Pharmacobiology, Faculty of Pharmacy, Jagiellonian University Medical College, Kraków, Poland

[4]Department of Medicinal Chemistry, Maj Institute of Pharmacology, Polish Academy of Sciences, Kraków, Poland

# These authors contributed equally to this work
* Correspondence: mfkonono@cyf-kr.edu.pl; Tel./fax: +48-12-620-55-80/ +48-12-620-55-96 (K.K.); t.karcz@uj.edu.pl; Tel.: +48-12-620-55-92 (T.K.)

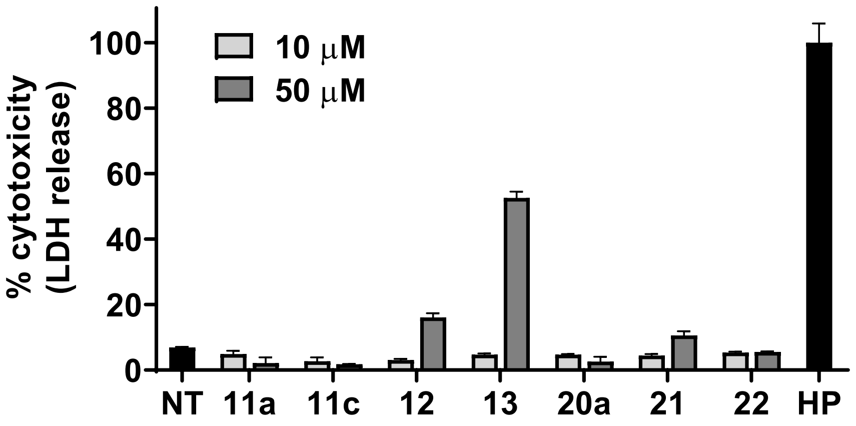

Supplementary Figure 1. Effects of selected xanthine derivatives on LDH release from SH-SY5Y cells. Experiments were conducted in six replicates. Results are normalized as percent of cytotoxicity induced by 300 μM hydrogen peroxide (HP) ± SEM. NT– vehicle control (1% DMSO).

Supplementary Table 1. Solvent, reaction time, crystal solvent and TLC properties for intermediate compounds.

Compd	Reaction medium	Time (h) reflux	Crystal. solvent	TLC benzene/acetone (7:3) R_f
8a	Me-Digol	4	methoxyethanol	0,43
8b	Me-Digol	4	butanol	0,51
8c	1-butanol	13	ethanol	0,65
8d	Me-Digol	4	ethanol	0,38
8e	Me-Digol	4	ethanol	0,50
8f	Me-Digol	7	ethanol	0,60
8g	Me-Digol	10	propanol	0,63
8h	Me-Digol	10	ethanol + H_2O	0,64
8i	Me-Digol	4	propanol + H_2O	0,69
8j	Me-Digol	3	ethanol	0,68
8k	Me-Digol	5	ethanol	0,65
8l	Me-Digol	4	ethanol	0,68
9	Me-Digol	10	ethanol + H_2O	0,73
10	Me-Digol	10	ethanol + H_2O	0,83
17a	Ref.1			
17b	DMF	10	ethanol	0.63
18	Ref.1			
19	DMF	10	ethanol + H_2O	0.77

General procedure for the synthesis of 1,3-dialkyl-8-3′,4′-dimethoxyphenylethyl-amino-7-alkyl- (or phenylalkyl- or phenoxyethyl-) xanthines.

A mixture of 2 mmol of appropriate 7-alkyl, 7-phenylalkyl,7-phenoxyethyl-1,3-dialkyl-8-bromoxanthine and 4 mmol of 3′4′-dimethoxyphenylethylamine was refluxed in 10 mL of Me-Digol or 10 mL of 1-butanol (comp **8b**) for 4-13h. The products were separated by cooling (comp **8b**, **8c**, **8e**) or by adding water to the reaction mixture. The resulting solids were filtered off and recrystallised.

8-((3,4-Dimethoxyphenethyl)amino)-1,3,7-trimethyl-3,7-dihydro-1*H*-purine-2,6-dione (8a)
Yield: 99 %; mp: 218–220 °C; Anal. for $C_{18}H_{23}N_5O_4$: Calcd: C, 57.90; H, 6.21; N, 18.76. Found: C, 58.05; H, 6.26; N, 18.74; UPLC/MS purity 100.00 %, t_R = 4.50, MW 373.41, $[M+H]^+$ 374.36; ^{1}H NMR (300 MHz, CHLOROFORM-*d*) δ ppm: 2.90 (t, *J* = 6.67 Hz, 2H, $NHCH_2C\underline{H}_2$), 3.37 (s, 3H, $N1CH_3$), 3.56 (d, *J* = 8.21 Hz, 6H, $N3CH_3$, $N7CH_3$), 3.74 (q, *J* = 6.54 Hz, 2H, $NHC\underline{H}_2CH_2$), 3.87 (s, 6H, 2 OCH_3), 4.32 (bs, 1H, $N\underline{H}$), 6.71-6.75 (m, 2H, C2H, C6H, phe), 6.82 (d, *J* = 8.72 Hz, 1H, C5H, phe). ^{13}C NMR (CHLOROFORM-*d*) δ ppm: 154.17 (C4Phe), 153.08 (C6), 151.69 (C2), 149.18 (C4), 148.35 (C8), 147.89 (C3Phe), 130.93 (C1Phe), 120.74 (C2Phe), 111.93 (C5Phe), 111.39 (C6Phe), 103.07 (C5), 55.94 ($C4PheO\underline{C}H_3$), 55.91 ($C3PheO\underline{C}H_3$), 44.94 ($NHCH_2$), 35.42 ($NHCH_2\underline{C}H_2$), 29.78 ($N1CH_3$), 29.78 ($N3CH_3$), 27.64 ($N7CH_3$). IR ν (cm^{-1}): 3332 (NH), 1698 (C=O), 1660 (C=O), 1276 (aryl OCH_3), 1081 (aryl OCH_3); UV λ_{max} (nm): 294.

8-((3,4-Dimethoxyphenethyl)amino)-7-ethyl-1,3-dimethyl-3,7-dihydro-1*H*-purine-2,6-dione (8b)
Yield: 90 %; mp: 211–213 °C; Anal. for $C_{19}H_{25}N_5O_4$: Calcd: C, 58.90; H, 6.50; N, 18.08. Found: C, 58.87; H, 6.48; N, 18.08. UPLC/MS purity 97.48 %, t_R = 4.88, MW 387.44, $[M+H]^+$ 388.39. ^{1}H NMR (300 MHz, CHLOROFORM-*d*) δ ppm: 1.24 (t, *J* = 7.31 Hz, 3H, $N7CH_2C\underline{H}_3$), 2.91 (t, *J* = 6.67 Hz, 2H, $NHCH_2C\underline{H}_2$), 3.38 (s, 3H, $N1C\underline{H}_3$), 3.55 (s, 3H, $N3C\underline{H}_3$), 3.74 (q, *J* = 6.41 Hz, 2H, $NHC\underline{H}_2CH_2$), 3.87 (s, 6H, 2 OCH_3), 3.99 (q, *J* = 7.31 Hz, 2H, $N7C\underline{H}_2$), 4.18 (bs, 1H, $N\underline{H}$), 6.72-6.74 (m, 2H, C2H, C6H, phe), 6.83 (d, *J* = 8.46 Hz, 1H, C5H, phe). ^{13}C NMR (CHLOROFORM-*d*) δ ppm: 153.90 (C4Phe), 152.22 (C6), 151.76 (C2), 149.19 (C4), 148.59 (C8), 147.89 (C3Phe), 131.02 (C1Phe), 120.77 (C2Phe), 111.94 (C5Phe), 111.40 (C6Phe), 102.43 (C5), 55.95 ($C4PheO\underline{C}H_3$), 55.89 ($C3PheO\underline{C}H_3$), 44.44 ($NH\underline{C}H_2$), 38.16 ($N7CH_2$), 35.37($NHCH_2\underline{C}H_2$), 29.75 ($N1CH_3$), 27.65 ($N3CH_3$), 14.68 ($N7CH_2\underline{C}H_3$). IR ν (cm^{-1}): 3344 (NH), 1694 (C=O), 1649 (C=O), 1233 (aryl OCH_3), 1097 (aryl OCH_3); UV λ_{max} (nm): 294.

8-((3,4-Dimethoxyphenethyl)amino)-1,3-dimethyl-7-propyl-3,7-dihydro-1*H*-purine-2,6-dione (8c)
Yield: 86 %; mp: 191–193 °C; Anal. for $C_{20}H_{27}N_5O_4$: Calcd: C, 59.84; H, 6.78; N, 17.44. Found: C, 59.68; H, 6.58; N, 17.39. UPLC/MS purity 96.40 %, t_R = 5.46, MW 401.47, $[M+H]^+$ 402.42. ^{1}H NMR (300 MHz, CHLOROFORM-*d*) δ ppm: 0.86 (t, *J* = 7.31 Hz, 3H, $N7CH_2CH_2C\underline{H}_3$), 1.62-1.69 (m, 2H, $N7CH_2C\underline{H}_2CH_3$), 2.91 (t, *J* = 6.67 Hz, 2H, $NHCH_2C\underline{H}_2$), 3.37 (s, 3H, $N1C\underline{H}_3$), 3.55 (s, 3H, $N3C\underline{H}_3$), 3.74 (q, *J* = 6.28 Hz, 2H, $NHC\underline{H}_2CH_2$), 3.89 (t, *J* = 6.70 Hz, 8H, $N7C\underline{H}_2$, 2 $OC\underline{H}_3$), 4.25 (bs, 1H, $N\underline{H}$), 6.71-6.75 (m, 2H, C2H, C6H, phe), 6.82 (d, *J* = 8.46 Hz, 1H, C5H, phe). ^{13}C NMR (CHLOROFORM-*d*) ⊚ ppm: 153.89 (C4Phe), 152.42 (C6), 151.74 (C2), 149.24 (C4), 148.31 (C8), 147.94 (C3Phe), 130.93 (C1Phe), 120.77 (C2Phe), 111.90 (C5Phe), 111.39 (C6Phe), 102.91 (C5), 55.97 ($C4PheO\underline{C}H_3$), 55.89 ($C3PheO\underline{C}H_3$), 44.78 ($NH\underline{C}H_2$), 35.33($NHCH_2\underline{C}H_2$), 29.81 ($N1\underline{C}H_3$),

27.68 (N3$\underline{C}H_3$), 22.75 (N7$CH_2\underline{C}H_2$) 10.94 (N7$CH_2CH_2\underline{C}H_3$). IR ν (cm^{-1}): 3353 (NH), 1694 (C=O), 1649 (C=O), 1261 (aryl OCH_3), 1025 (aryl OCH_3); UV λ_{max} (nm): 293.

8-((3,4-Dimethoxyphenethyl)amino)-7-*iso*propyl-1,3-dimethyl-3,7-dihydro-1*H*-purine-2,6-dione (8d)

Yield: 98 %; mp: 202–204 °C; Anal. for $C_{20}H_{27}N_5O_4$: Calcd: C, 59.84; H, 6.78; N, 17.44. Found: C, 59.93; H, 6.88; N, 17.13. UPLC/MS purity 96.40 %, t_R = 5.46, MW 401.47, $[M+H]^+$ 402.40. ^{1}H NMR (300 MHz, CHLOROFORM-*d*) δ ppm: 1.43 (d, *J* = 6.92 Hz, 6H, NHCH($C\underline{H}_3$)$_2$, 2.90 (t, *J* = 6.67 Hz, $NHCH_2C\underline{H}_2$), 3.37 (s, 3H, N1$C\underline{H}_3$), 3.54 (s, 3H, N3$C\underline{H}_3$), 3.73 (q, *J* = 5.90 Hz, 2H, NH$C\underline{H}_2$), 3.86 (s, 6H, 2xO$C\underline{H}_3$), 4.25 (bs, 1H, N$\underline{H}$), 4.64 (bs, 1H, N7C$\underline{H}$), 6.76 (d, *J* = 5.39 Hz, 2H, C2H, C6H, phe), 6.84 (d, *J* = 8.72 Hz, 1H, C5H, phe). ^{13}C NMR (CHLOROFORM-*d*) δ ppm: 153.69 (C4Phe), 152.13 (C6), 151.71 (C2), 149.25 (C4), 149.01 (C8), 147.94 (C3Phe), 131.05 (C1Phe), 120.82 (C2Phe), 111.93 (C5Phe), 111.41 (C6Phe), 102.68 (C5), 55.98 (C4PheO$\underline{C}H_3$), 55.90 (C3PheO$\underline{C}H_3$), 47.13 (N7$\underline{C}$H), 44.73 (NH$\underline{C}H_2$), 35.28 ($NHCH_2\underline{C}H_2$), 29.78 (N1$\underline{C}H_3$), 27.99 (N3$\underline{C}H_3$), 21.08 (N7CH($\underline{C}H_3$)$_2$). IR ν (cm^{-1}): 3356 (NH), 1690 (C=O), 1650 (C=O), 1281 (aryl OCH_3), 1067 (aryl OCH_3); UV λ_{max} (nm): 295.

7-Butyl-8-((3,4-dimethoxyphenethyl)amino)-1,3-dimethyl-3,7-dihydro-1*H*-purine-2,6-dione (8e)

Yield: 98 %; mp: 155-157 °C; Anal. for $C_{21}H_{29}N_5O_4$: Calcd: C, 60.71; H, 7.04; N, 16.86. Found: C, 60.30; H, 6.94; N, 17.05. UPLC/MS purity 97.45 %, t_R = 5.80, MW 415.49, $[M+H]^+$ 416.44. ^{1}H NMR (300 MHz, DMSO-d_6) δ ppm: 0.82 (t, *J* = 7.31 Hz, 3H, N7$CH_2CH_2CH_2C\underline{H}_3$), 1.15-1.22 (m, 2H, N7$CH_2CH_2C\underline{H}_2$), 1.46-1.53 (m, 2H, N7$CH_2C\underline{H}_2$), 2.80 (t, *J* = 7.44 Hz, 2H, $NHCH_2C\underline{H}_2$), 3.15 (s, 3H, N1CH_3), 3.35 (s, 3H, N3CH_3), 3.51 (q, *J* = 6.29 Hz, 2H, NH$C\underline{H}_2$), 3.70(d, *J* = 7.34 Hz, 6H, 2 O$C\underline{H}_3$), 3.96 (t, *J* = 7.31 Hz, 2H, N7$C\underline{H}_2$), 6.70 (d, *J* = 7.94 Hz, 1H, C5H, phe), 6.81-6.84 (m, 2H, C2H, C6H, phe),7.02 (t, *J* = 5.51 Hz, 1H, N$\underline{H}$). ^{13}C NMR (DMSO-d_6) δ ppm: 153.69 (C4Phe), 153.02 (C6), 151.42 (C2), 149.08 (C4), 148.98 (C8), 147.71 (C3Phe), 132.37 (C1Phe), 121.07 (C2Phe), 113.11 (C5Phe), 112.35 (C6Phe), 101.83 (C5), 55.96 (C4PheO$\underline{C}H_3$), 55.80 (C3PheO$\underline{C}H_3$), 44.59 (N7$\underline{C}H_2$), 42.59 (NH$\underline{C}H_2$), 35.94 ($NHCH_2\underline{C}H_2$), 31.63 (N1$\underline{C}H_3$), 29.66(N3$\underline{C}H_3$), 27.62 (N7$CH_2\underline{C}H_2$), 19.49 (N7$CH_2CH_2\underline{C}H_2$), 14.05 (N7$CH_2CH_2CH_2\underline{C}H_3$). IR ν ($cm^{-1}$): 3331 (NH), 1696 (C=O), 1654 (C=O), 1287 (aryl OCH_3), 1039 (aryl OCH_3); UV λ_{max} (nm): 296.

8-((3,4-Dimethoxyphenethyl)amino)-7-*iso*butyl-1,3-dimethyl-3,7-dihydro-1*H*-purine-2,6-dione (8f)

Yield: 98 %; mp: 172-174 °C; Anal. for $C_{21}H_{29}N_5O_4$: Calcd: C, 60.71; H, 7.04; N, 16.86. Found: C, 60.62; H, 6.95; N, 16.94. UPLC/MS purity 97.20 %, t_R = 5.73, MW 415.49, $[M+H]^+$ 416.44. ^{1}H NMR (300 MHz, CHLOROFORM-*d*) δ ppm: 0.85 (d, *J* = 6.67 Hz, 6H, N7$CH_2CH(C\underline{H}_3)_2$), 1.97-2.02 (m, 1H, N7$CH_2C\underline{H}$), 2.92 (t, *J* = 6.67 Hz, 2H, $NHCH_2C\underline{H}_2$), 3.36 (s, 3H, N1$C\underline{H}_3$), 3.60 (s, 3H, N3$C\underline{H}_3$), 3.72(d, *J* = 7.69 Hz, 2H, N7$C\underline{H}_2$) 3.78-3.88 (m, 8H, NH$C\underline{H}_2$, 2 O$C\underline{H}_3$), 4.40 (bs, 1H, N$\underline{H}$), 6.72-6.76 (m, 2H, C2H, C6H, phe), 6.82 (d, *J* = 7.55 Hz, 1H, C5H, phe). ^{13}C NMR (CHLOROFORM-*d*) δ ppm: 153.86 (C4Phe), 152.68 (C6), 151.74 (C2), 149.28 (C4), 148.31 (C8), 147.95 (C3Phe), 130.90 (C1Phe), 120.78 (C2Phe), 111.90 (C5Phe), 111.39 (C6Phe), 103.18 (C5), 55.98 (C4PheO$\underline{C}H_3$),

55.89 (C3PheO$\underline{C}H_3$), 50.49 (N7$\underline{C}H_2$), 44.40 (NH$\underline{C}H_2$), 35.29 (NHCH$_2\underline{C}H_2$), 29.78 (N1$\underline{C}H_3$), 28.92 (N3$\underline{C}H_3$), 27.68 (N7CH$_2\underline{C}$H), 19.70 (N7CH$_2$CH($\underline{C}H_3$)$_2$). IR ν (cm^{-1}): 3363 (NH), 1695 (C=O), 1652 (C=O), 1291 (aryl OCH$_3$), 1050 (aryl OCH$_3$); UV λ_{max} (nm): 296.

7-(*sec*-Butyl)-8-((3,4-dimethoxyphenethyl)amino)-1,3-dimethyl-3,7-dihydro-1*H*-purine-2,6-dione (8g)

Yield: 97 %; mp: 178-180 °C; Anal. for $C_{21}H_{29}N_5O_4$: Calcd: C, 60.71; H, 7.04; N, 16.86. Found: C, 60.75; H, 6.84; N, 16.55. UPLC/MS purity 98.43 %, t_R = 5.83, MW 415.49, [M+H]$^+$ 416.44. ^{1}H NMR (300 MHz, CHLOROFORM-*d*) δ ppm: 0.75 (t, *J* = 7.44 Hz, 3H, N7CH(CH$_3$)CH$_2$C$\underline{H}_3$), 1.42 (d, *J* = 6.92 Hz, 3H, N7CH(C$\underline{H}_3$)CH$_2$CH$_3$), 1.69-1.90 (m, 2H, N7CH(CH$_3$)C$\underline{H}_2$CH$_3$), 2.90 (t, *J* = 6.54 Hz, 2H, NHCH$_2$C$\underline{H}_2$), 3.37 (s, 3H, N1C$\underline{H}_3$), 3.55 (s, 3H, N3C$\underline{H}_3$), 3.74 (q, *J* = 6.15 Hz, NHC$\underline{H}_2$), 3.86 (s, 6H, 2 OC$\underline{H}_3$), 4.19 (bs, 1H, N7 C$\underline{H}$), 6.71-6.74 (m, 2H, C2H, C6H, phe), 6.80-6.83(m, 1H, C5H, phe). ^{13}C NMR (CHLOROFORM-*d*) δ ppm: 153.62 (C4Phe), 152.58 (C6), 151.69 (C2), 149.26 (C4), 148.53 (C8), 147.93 (C3Phe), 131.02 (C1Phe), 120.82 (C2Phe), 111.92 (C5Phe), 111.41 (C6Phe), 102.43 (C5), 55.98 (C4PheO$\underline{C}H_3$), 53.90 (C3PheO$\underline{C}H_3$), 53.34 (N7$\underline{C}$H), 44.68 (NH$\underline{C}H_2$), 35.27 (NHCH$_2\underline{C}H_2$), 29.81 (N1$\underline{C}H_3$), 28.10 (N3$\underline{C}H_3$), 27.99 (N7CH($\underline{C}H_3$)), 19.46 (N7CH(CH$_3$)$\underline{C}H_2$), 11.29 (N7CH(CH$_3$)CH$_2\underline{C}H_3$). IR ν (cm^{-1}): 3359 (NH), 1690 (C=O), 1652 (C=O), 1286 (aryl OCH$_3$), 1040 (aryl OCH$_3$); UV λ_{max} (nm): 293.

8-((3,4-Dimethoxyphenethyl)amino)-1,3-dimethyl-7-pentyl-3,7-dihydro-1*H*-purine-2,6-dione (8h)

Yield: 98 %; mp: 118-120 °C; Anal. for $C_{22}H_{31}N_5O_4$: Calcd: C, 61.52; H, 7.28; N, 16.31. Found: C, 61.48; H, 7.17; N, 16.21. UPLC/MS purity 95.49 %, t_R = 6.30, MW 429.52, [M+H]$^+$ 430.19. ^{1}H NMR (300 MHz, CHLOROFORM-*d*) δ ppm: 0.85 (t, *J* = 7.05 Hz, 3H, N7CH$_2$CH$_2$CH$_2$CH$_2$C$\underline{H}_3$) 1.18-1.29 (m, 4H, N7CH$_2$CH$_2$C$\underline{H}_2$C$\underline{H}_2$), 1.58-1.63 (m, 2H, N7CH$_2$C$\underline{H}_2$) 2.91 (t, *J* = 6.67 Hz, 2H, NHCH$_2$C$\underline{H}_2$), 3.37 (s, 3H, N1C$\underline{H}_3$), 3.55 (s, 3H, N3C$\underline{H}_3$), 3.75(q, *J* = 6.41 Hz, 2H, NHC$\underline{H}_2$) 3.86-3.93 (m, 8H, N7C$\underline{H}_2$, 2 OC$\underline{H}_3$), 4.12 (bs, 1H, N$\underline{H}$), 6.71-6.75 (m, 2H, C2H, C6H, phe), 6.82 (d, *J* = 8.72 Hz,1H, C5H, phe). ^{13}C NMR (CHLOROFORM-*d*) δ ppm: 153.89 (C4Phe), 152.38 (C6), 151.75 (C2), 149.28 (C4), 148.34 (C8), 147.94 (C3Phe), 130.89 (C1Phe), 120.76 (C2Phe), 111.85 (C5Phe), 111.33 (C6Phe), 102.84 (C5), 55.92 (C4PheO$\underline{C}H_3$), 55.89 (C3PheO$\underline{C}H_3$), 44.36 (N7$\underline{C}H_2$), 43.36 (NH$\underline{C}H_2$), 35.29 (NHCH$_2\underline{C}H_2$), 29.78 (N1$\underline{C}H_3$), 29.20 (N3$\underline{C}H_3$), 28.62 (N7CH$_2\underline{C}H_2$), 27.68 (N7CH$_2$CH$_2\underline{C}H_2$), 22.29 (N7CH$_2$CH$_2$CH$_2\underline{C}H_2$), 13.88 (N7CH$_2$CH$_2$CH$_2$CH$_2\underline{C}H_3$). IR ν (cm^{-1}): 3332 (NH), 1699 (C=O), 1646 (C=O), 1289 (aryl OCH$_3$), 1041 (aryl OCH$_3$); UV λ_{max} (nm): 294.

8-((3,4-Dimethoxyphenethyl)amino)-7-hexyl-1,3-dimethyl-3,7-dihydro-1*H*-purine-2,6-dione (8i)

Yield: 98 %; mp: 112-114 °C; Anal. for $C_{23}H_{33}N_5O_4$: Calcd: C, 62.28; H, 7.50; N, 15.79. Found: C, 62.39; H, 7.58; N, 15.77. UPLC/MS purity 97.19 %, t_R = 6.86, , MW 443.55, [M+H]$^+$ 444.21. ^{1}H NMR (300 MHz, CHLOROFORM-*d*) δ ppm: 0.85 (t, *J* = 6.67 Hz, 3H, N7CH$_2$CH$_2$CH$_2$CH$_2$CH$_2$C$\underline{H}_3$) 1.24 (s, 6H, N7CH$_2$CH$_2$C$\underline{H}_2$C$\underline{H}_2$C$\underline{H}_2$), 1.60 (s, 2H, N7CH$_2$C$\underline{H}_2$) 2.91 (t, *J* = 6.54 Hz, 2H, NHCH$_2$C$\underline{H}_2$), 3.37 (s, 3H, N1C$\underline{H}_3$), 3.56 (s, 3H, N3C$\underline{H}_3$), 3.76 (t, *J* = 6.03 Hz, 2H, NHC$\underline{H}_2$) 3.86-3.93 (m, 8H, N7C$\underline{H}_2$, 2 OC$\underline{H}_3$), 4.23 (bs, 1H, N$\underline{H}$), 6.71-6.74 (m, 2H, C2H, C6H, phe), 6.82 (d, *J* = 8.72 Hz, 1H, C5H, phe). ^{13}C NMR (CHLOROFORM-*d*) δ ppm: 153.89 (C4Phe),

152.27 (C6), 151.73 (C2), 149.28 (C4), 147.95 (C8/C3Phe), 130.86 (C1Phe), 120.75 (C2Phe), 111.86 (C5Phe), 111.32 (C6Phe), 102.83 (C5), 55.91 (C4PheOCH$_3$), 55.90 (C3PheOCH$_3$), 44.40 (N7CH$_2$), 43.44 (NHCH$_2$), 35.31 (NHCH$_2$CH$_2$), 31.35 (N7CH$_2$CH$_2$CH$_2$), 29.85 (N1CH$_3$), 29.47 (N3CH$_3$), 27.70 (N7CH$_2$CH$_2$), 26.24 (N7CH$_2$CH$_2$CH$_2$CH$_2$), 22.49 (N7CH$_2$CH$_2$CH$_2$CH$_2$CH$_2$), 13.96 (N7CH$_2$CH$_2$CH$_2$CH$_2$CH$_2$CH$_3$). IR ν (cm^{-1}): 3337 (NH), 1701 (C=O), 1658 (C=O), 1281 (aryl OCH$_3$), 1040 (aryl OCH$_3$); UV λ_{max} (nm): 297

7-Benzyl-8-((3,4-dimethoxyphenethyl)amino)-1,3-dimethyl-3,7-dihydro-1*H*-purine-2,6-dione (8j)

Yield: 75 %; mp: 167-170 °C; Anal. for $C_{24}H_{27}N_5O_4$: Calcd: C, 64.13; H, 6.06; N, 15.58. Found: C, 64.09; H, 6.06; N, 15.68. UPLC/MS purity 98.84 %, t_R = 5.95, MW 449,51, $[M+H]^+$ 450.31; ^{1}H NMR (300 MHz, CHLOROFORM-*d*) δ ppm: 2.79 (t, *J* = 6.54 Hz, 2H, NHCH$_2$CH$_2$), 3.38 (s, 3H, N1CH$_3$), 3.57 (s, 3H, N3CH$_3$) 3.67 (q, *J*=6.16 Hz, 2H, NHCH$_2$), 3.81 (s, 3H, 4'OCH$_3$), 3.87 (s, 3H, 3'OCH$_3$), 4.13-4.15 (m, 1H, NH), 5.22 (s, 2H, N7CH$_2$) 6.57 (d, *J* = 7.95 Hz, 1H, C5H, phe), 6.63 (s, 1H, C2H, phe) 6.73 (d, *J* = 7.95Hz, 1H, C6H, phe), 7.05-7.08 (m, 2H, C2H, C6H, benzyl), 7.24-7.28 (m, 3H, C3H, C4H, C5H, benzyl); 13C NMR (CHLOROFORM-*d*) ◎ ppm: 154.2 (C4Phe), 153.1 (C6), 151.7 (C2), 149.2 (C4), 148.5 (C8), 147.9 (C3Phe), 135.1 (C1benzyl), 130.6 (C1Phe), 129.1 (C3/C5benzyl), 128.1 (C4benzyl), 127.0 (C2/6benzyl), 120.6 (C2Phe), 111.7 (C5Phe), 111.3 (C6Phe), 102.9 (C5), 55.90 (C4PheOCH$_3$), 55.8 (C3PheOCH$_3$), 46.6 (N7CH$_2$), 44.1 (NHCH$_2$), 35.1 (NHCH$_2$CH$_2$), 29.8 (N1CH$_3$), 27.7 (N3CH$_3$); IR ν (cm^{-1}): 3331 (NH), 1696 (C=O), 1654 (C=O), 1289 (aryl OCH$_3$), 1039 (aryl OCH$_3$); UV λ_{max} (nm): 297

8-((3,4-Dimethoxyphenethyl)amino)-1,3-dimethyl-7-phenethyl-3,7-dihydro-1*H*-purine-2,6-dione (8k)

Yield: 95 %; mp: 168-171 °C; Anal. for $C_{25}H_{29}N_5O_4$: Calcd: C, 64.79; H, 6.31; N, 15.11. Found: C, 64.69; H, 6.21; N, 15.13. UPLC/MS purity 96.23 %, t_R = 5.95, MW 463,52, $[M+H]^+$ 464.15; ^{1}H NMR (300 MHz, CHLOROFORM-*d*) δ ppm: 2.61 (t, *J* = 6.92Hz, 2H, NHCH$_2$CH$_2$), 3.00 (t, *J* = 6.67 Hz, 2H, NHCH$_2$) 3.37-3.42 (m, 5H, N1CH$_3$ N7CH$_2$CH$_2$), 3.44 (bs, 1H, NH), 3.55 (s, 3H, N3CH$_3$) 3.87 (s, 6H, 2 OCH$_3$), 4.14 (t, *J* = 6.54 Hz, 2H, N7CH$_2$), 6.65-6.68 (m, 2H, C2H, C6H, phe), 6.81(d, *J* = 8.46 Hz, 1H, C5H, phe), 7.03-7.06 (m, 2H, C2H, C6H, phe-ethyl), 7.22-7.33 (m, 3H, C3H, C4H,C5H, phe-ethyl); 13C NMR (CHLOROFORM-*d*) ◎ ppm: 154.0 (C4Phe), 153.0 (C6), 151.8 (C2), 149.2 (C4), 148.6 (C8), 147.9 (C3Phe), 138.3 (C1, phe-ethyl), 131.0 (C1Phe), 129.0 (C3/C5, phe-ethyl), 128.8 (C4, phe-ethyl), 127.0 (C2/6, phe-ethyl), 120.7 (C2Phe), 111.9 (C5Phe), 111.3 (C6Phe), 102.6 (C5), 56.0 (C4PheOCH$_3$), 55.9 (C3PheOCH$_3$), 45.7 (N7CH$_2$), 44.5 (N7CH$_2$CH$_2$), 36.5 (NHCH$_2$), 35.3 (NHCH$_2$CH$_2$), 29.8 (N1CH$_3$), 27.7 (N3CH$_3$); IR ν (cm^{-1}): 3379 (NH), 1690 (C=O), 1647 (C=O), 1258 (aryl OCH$_3$), 1027 (aryl OCH$_3$); UV λ_{max} (nm): 296.

8-((3,4-Dimethoxyphenethyl)amino)-1,3-dimethyl-7-(2-phenoxyethyl)-3,7-dihydro-1*H*-purine-2,6-dione (8l)

Yield: 88 %; mp: 142-145 °C; Anal. for $C_{25}H_{29}N_5O_5$: Calcd: C, 62.62; H, 6.10; N, 14.60. Found: C, 62.63; H, 6.34; N, 14.51. UPLC/MS purity 96.48 %, t_R = 6.25, MW 479,52, $[M+H]^+$ 480.17; ^{1}H NMR (300 MHz, CHLOROFORM-*d*) δ ppm: 2.95 (t, *J* = 6.80 Hz, 2H, NHCH$_2$CH$_2$), 3.37 (s, 3H, N1CH$_3$), 3.57 (s, 3H, N3CH$_3$), 3.71-3.81 (m, 5H, 3'OCH$_3$, NHCH$_2$CH$_2$), 3.86 (s, 3H, 4'OCH$_3$), 4.27 (t, *J* = 4.41 Hz, 2H, N7CH$_2$CH$_2$O),4.42 (t, *J* = 4.36 Hz, 2H, N7CH$_2$CH$_2$O), 5.40 (bs, 1H, NH), 6.54-6.58 (m, 2H, C2H, C6H, phe), 6.75-6.82 (m, 3H, C3H, C4H,C5H, phenoxy), 6.93-6.99 (m, 1H, C5H, phe), 7.19-7.26 (m, 2H, C2H, C6H, phenoxy); 13C NMR (CHLOROFORM-*d*) ◎ ppm: 157.4 (C1,

phenoxy), 154.5 (C4Phe), 154.2 (C6), 151.7 (C2), 149.3 (C4), 148.5 (C8), 147.9 (C3Phe), 131.1 (C1Phe), 129.6 (C3/C5, phenoxy), 121.9 (C2Phe), 120.7 (C5Phe), 114.3 (C2/6, phenoxy), 111.9 (C6Phe), 111.4 (C4, phenoxy), 102.7 (C5), 68.5 ($N7CH_2\underline{C}H_2O$),55.9 ($C4PheO\underline{C}H_3$), 55.9 ($C3PheO\underline{C}H_3$), 44.7 ($N7\underline{C}H_2$), 43.9 ($NH\underline{C}H_2$), 35.6 ($NHCH_2\underline{C}H_2$), 29.9 ($N1\underline{C}H_3$), 27.7 ($N3\underline{C}H_3$); IR ν (cm^{-1}): 3395(NH), 1696 (C=O), 1654 (C=O), 1292 (aryl OCH_3), 1038 (aryl OCH_3); UV λ_{max} (nm): 296.

8-((3,4-Dimethoxyphenethyl)amino)-1,3,7-tripropyl-3,7-dihydro-1*H*-purine-2,6-dione (9)
Yield: 79 %; mp: 75-78 °C. UPLC/MS purity 98.61 %, t_R = 7.45, MW 457,58, $[M+H]^+$ 458.24; ^1H NMR (300 MHz, CHLOROFORM-*d*) δ ppm: 0.85-1.00 (m, 9H, 3 $CH_2CH_2C\underline{H}_3$) 1.62-1.83 (m, 6H, 3 $CH_2C\underline{H}_2CH_3$), 2.90 (t, *J* = 6.67 Hz, 2H, $NHCH_2C\underline{H}_2$), 3.73 (q, *J* = 5.77 Hz, 2H, $NHC\underline{H}_2$) 3.86-3.95 (m, 10H, $N1C\underline{H}_2$, $N3C\underline{H}_2$, 2 $OC\underline{H}_3$), 4.04 (t, *J* = 7.19 Hz, $N7C\underline{H}_2$), 4.25 (bs, 1H, $N\underline{H}$), 6.71-6.75 (m, 2H, C2H, C6H, phe), 6.82 (d, *J* = 8.46 Hz, 1H, C5H, phe). ^{13}C NMR (CHLOROFORM-*d*) δ ppm: 153.9 (C4Phe), 152.3 (C6), 151.2 (C2), 149.2 (C8), 147.9 (C3Phe), 131.1 (C1Phe), 120.8 (C2Phe), 112.0 (C5Phe), 111.4 (C6Phe), 103.0 (C5), 56.0 ($C4PheO\underline{C}H_3$), 55.9 ($C3PheO\underline{C}H_3$), 44.8 ($N7CH_2$), 44.6 ($N1CH_2$), 42.6 ($NHCH_2$), 35.4 ($NHCH_2CH_2$), 22.9 ($N7CH_2\underline{C}H_2$), 21.4 ($N3CH_2\underline{C}H_2/N1CH_2\underline{C}H_2$), 11.4 ($N3CH_2CH_2\underline{C}H_3$), 11.3 ($N1CH_2CH_2\underline{C}H_3$), 11.0 ($N7CH_2CH_2\underline{C}H_3$). IR ν ($cm^{-1}$): 3351 (NH), 1697 (C=O), 1639 (C=O), 1262 (aryl OCH_3), 1028 (aryl OCH_3); UV λ_{max} (nm): 297

1,3-dibutyl-8-((3,4-dimethoxyphenethyl)amino)-7-propyl-3,7-dihydro-1*H*-purine-2,6-dione (10)
Yield: 99 %; mp: 102-105 °C. UPLC/MS purity 96.60 %, t_R = 8.56, MW 485,61, $[M+H]^+$ 486.51; ^1H NMR (300 MHz, CHLOROFORM-*d*) δ ppm: 0.85-1.00 (m, 9H, $CH_2CH_2C\underline{H}_3$ + 2 x $CH_2CH_2CH_2C\underline{H}_3$), 1.31-1.46 (m, 4H, 2x $CH_2CH_2C\underline{H}_2CH_3$) 1.56-1.79 (m, 6H, 3 $CH_2C\underline{H}_2CH_3$, 2 x $CH_2C\underline{H}_2CH_2CH_3$), 2.90 (t, *J* = 6.67 Hz, 2H, $NHCH_2C\underline{H}_2$), 3.72 (q, *J* = 6.41 Hz, 2H, $NHC\underline{H}_2$) 3.83-3.90 (m, 8H, $N7C\underline{H}_2$, 2 $OC\underline{H}_3$), 3.96 (t, *J* = 7.56 Hz, $N3C\underline{H}_2$), 4.07 (t, *J* = 7.43 Hz, $N1C\underline{H}_2$), 4.25 (bs, 1H, $N\underline{H}$), 6.71-6.75 (m, 2H, C2H, C6H, phe), 6.82 (d, *J* = 8.69 Hz, 1H, C5H, phe). ^{13}C NMR (CHLOROFORM-*d*) δ ppm: 153.9 (C4Phe), 152.4 (C6), 151.2 (C2), 149.2 (C8), 148.2 (C4), 147.9 (C3Phe), 131.1 (C1Phe), 120.8 (C2Phe), 111.9 (C5Phe), 111.4 (C6Phe), 103.0 (C5), 56.0 ($C4PheO\underline{C}H_3$), 55.9 ($C3PheO\underline{C}H_3$), 44.8 ($N7CH_2$), 44.5 ($N1CH_2$), 43.05 ($NHCH_2$), 40.9 ($N3CH_2$), 35.2 ($NHCH_2CH_2$), 30.3 ($N1CH_2CH_2$), 30.2 ($N3CH_2CH_2$), 22.9 ($N7CH_2CH_2$), 20.3 ($N1CH_2CH_2CH_2$), 20.0 ($N3CH_2CH_2CH_2$), 13.9 ($N3CH_2CH_2CH_2CH_3$), 13.8 ($N1CH_2CH_2CH_2CH_3$), 11.0 ($N7CH_2CH_2CH_3$). IR ν (cm^{-1}): 3356 (NH), 1699 (C=O), 1645 (C=O), 1262 (aryl OCH_3), 1026 (aryl OCH_3); UV λ_{max} (nm): 297

General procedure for the synthesis of *N*-9,10-3′,4′-dimethoxyphenylethyl-1,3-dialkyl-6,7,8,9-tetrahydropyrimido- (or 7,8,9,10-tetrahydro-1*H*-[1,3]diazepino-) [2,1-*f*]purine-2,4(1*H*,3*H*)diones.

A mixture of 4 mmol of 1,3-dialkyl 7-Cl(Br) propyl, butyl-8-bromoxanthines and 8 mmol of 3,4-dimethoxyphenylethylamine was refluxed in 10 mL of DMF for 10-12h. After cooling water was added and precipitated products were filtered off, dried and recrystallised from ethanol or ethanol and water.

9-(3,4-Dimethoxyphenethyl)-1,3-dimethyl-6,7,8,9-tetrahydropyrimido[2,1-*f*]purine-2,4(1*H*,3*H*)-dione (17a)

The synthesis and physicochemical properties of the compound were reported previously.[1]

10-(3,4-Dimethoxyphenethyl)-1,3-dimethyl-7,8,9,10-tetrahydro-1*H*-[1,3]diazepino[2,1-*f*]purine-2,4(3*H*,6*H*)-dione (17b)

Yield: 98 %; mp: 132–134 °C; ^{1}H NMR (300 MHz, CHLOROFORM-*d*) δ ppm: 1.78–1.86 (m, 4H, $CH_2C\underline{H}_2C\underline{H}_2CH_2$); 2.92 (t, *J* = 7.44 Hz, 2H, $N10CH_2C\underline{H}_2$); 3.25 (t, *J* = 5.00 Hz, 2H, $N10C\underline{H}_2CH_2$); 3.37 (s, 3H, $N3CH_3$); 3.55 (s, 3H, $N1CH_3$); 3.75 (t, *J* = 7.44 Hz, 2H, $CH_2CH_2CH_2C\underline{H}_2$); 3.86 (d, *J* = 2,05 Hz, 6H, 2 OCH_3); 4.15 (t, *J* = 5.13 Hz, 2H, $C\underline{H}_2CH_2CH_2CH_2$); 6.73–6.81 (m, 3H, C2H, C5H, C6H, phe); ^{13}C NMR (CHLOROFORM-*d*) ◎ ppm: 158.7 (C4Phe); 154.4 (C4); 151.8 (C2); 148.9 (C9a); 148.0; (C10a); 147.6 (C3Phe); 131.8 (C1Phe); 120.8 (C2Phe); 112.0 (C5Phe); 111.3 (C6Phe); 55.9 ($C4PheOCH_3$); 55.9 ($C3PheOCH_3$); 55.2 (C9); 52.3 (C8); 45.8 ($N10CH_2$); 33.9 ($N10CH_2\underline{C}H_2$); 29.7 (C6); 29.0 ($N3CH_3$); 27.7 ($N1CH_3$); 26.4 (C7); IR ν (cm^{-1}): 1690 (C=O), 1656 (C=O), 1277 (aryl OCH_3), 1072 (aryl OCH_3); UV λ_{max} (nm): 299; UPLC/MS purity 98.50 %, t_R = 5.76, $C_{21}H_{27}N_5O_4$, MW 413,48, $[M+H]^+$ 414.10. Anal. for $C_{21}H_{27}N_5O_4$: Calcd: C, 61.00; H, 6.58; N, 16.94. Found: C, 60.85; H, 6.91; N, 16.91.

9-(3,4-Dimethoxyphenethyl)-1,3-dipropyl-6,7,8,9-tetrahydropyrimido[2,1-*f*]purine-2,4(1*H*,3*H*)-dione (18)

The synthesis and physicochemical properties of the compound were reported previously.[1]

1,3-Dibutyl-9-(3,4-dimethoxyphenethyl)-6,7,8,9-tetrahydropyrimido[2,1-*f*]purine-2,4(1*H*,3*H*)-dione (19)

Yield: 67 %; mp: 82–85 °C; ^{1}H NMR (300 MHz, CHLOROFORM-*d*) δ ppm: 0.84–0.93 (m, 6H, 2 x $CH_2CH_2CH_2C\underline{H}_3$); 1.25–1.40 (m, 4H, 2 x $CH_2CH_2C\underline{H}_2CH_3$); 1.51–1.73 (m, 4H, 2 x $CH_2C\underline{H}_2CH_2CH_3$); 1.93–2.02 (m, 2H, $CH_2C\underline{H}_2CH_2$); 2.82 (t, *J* = 7.29 Hz, 2H, $CH_2C\underline{H}_2$); 3.15 (t, *J* = 5.64 Hz, 2H, $C\underline{H}_2CH_2$); 3.64 (t, *J* = 7.33 Hz, 2H, $CH_2CH_2C\underline{H}_2$); 3.79 (s, 6H, 2 x OCH_3); 3.89 (t, *J* = 7.54 Hz, 2H, $N3C\underline{H}_2$); 3.98 (t, *J* = 7.27 Hz, 2H, $N1C\underline{H}_2$); 4.11 (t, *J* = 6.00 Hz, 2H, $C\underline{H}_2CH_2CH_2$); 6.66–6.75 (m, 3H, C2H, C5H, C6H, phe); ^{13}C NMR (CHLOROFORM-*d*) ◎ ppm: 153.8 (C4Phe); 151.4 (C4); 151.3 (C2); 149.0 (C9a); 148.90 (C10a); 147.7 (C3Phe); 131.5 (C1Phe); 120.8 (C2Phe); 112.0 (C5Phe); 111.3 (C6Phe); 102. 9 (C4a); 55.2 ($C4PheOCH_3$); 55.8 ($C3PheOCH_3$); 51.7 (C8); 45.4 ($N9CH_2$); 42.9 ($N1CH_2$); 41.5 ($N3CH_2$); 40.8 (C6); 33.5 ($N9CH_2CH_2$); 30.4 ($N1CH_2CH_2$); 30.2 ($N3CH_2CH_2$); 21.5 (C7); 20.3 ($N1CH_2CH_3CH_2$); 20.1 ($N3CH_2CH_2CH_2$); 13.9 ($N3CH_2CH_2CH_2CH_3$); 13.8 ($N1CH_2CH_2CH_2CH_3$); IR ν (cm^{-1}): 1687 (CO), 1655 (CO), 1234 (aryl OCH_3), 1070 (aryl OCH_3); UV λ_{max} (nm): 302; UPLC/MS purity 97.27 %, t_R = 8.16, $C_{26}H_{37}N_5O_4$, MW 483.60, $[M+H]^+$ 484.22. Anal. for $C_{26}H_{37}N_5O_4$: Calcd: C, 64.58; H, 7.72; N, 14.48. Found: C, 64.98; H, 7.71; N, 14.39.

References

1. Drabczyńska, A.; Müller, C.E.; Schiedel, A.; Schumacher, B.; Karolak-Wojciechowska, J.; Fruziński, A.; Zobnina, W.; Yuzlenko, O.; Kieć-Kononowicz, K. Phenylethyl-Substituted Pyrimido[2,1-f]Purinediones and Related Compounds: Structure–Activity Relationships as Adenosine A1 and A2A Receptor Ligands. *Bioorg. Med. Chem.* **2007**, *15*, 6956–6974, doi:10.1016/j.bmc.2007.07.051.

^{1}H and ^{13}C spectra for intermediates and final compounds

Compound 11a
^{1}H NMR

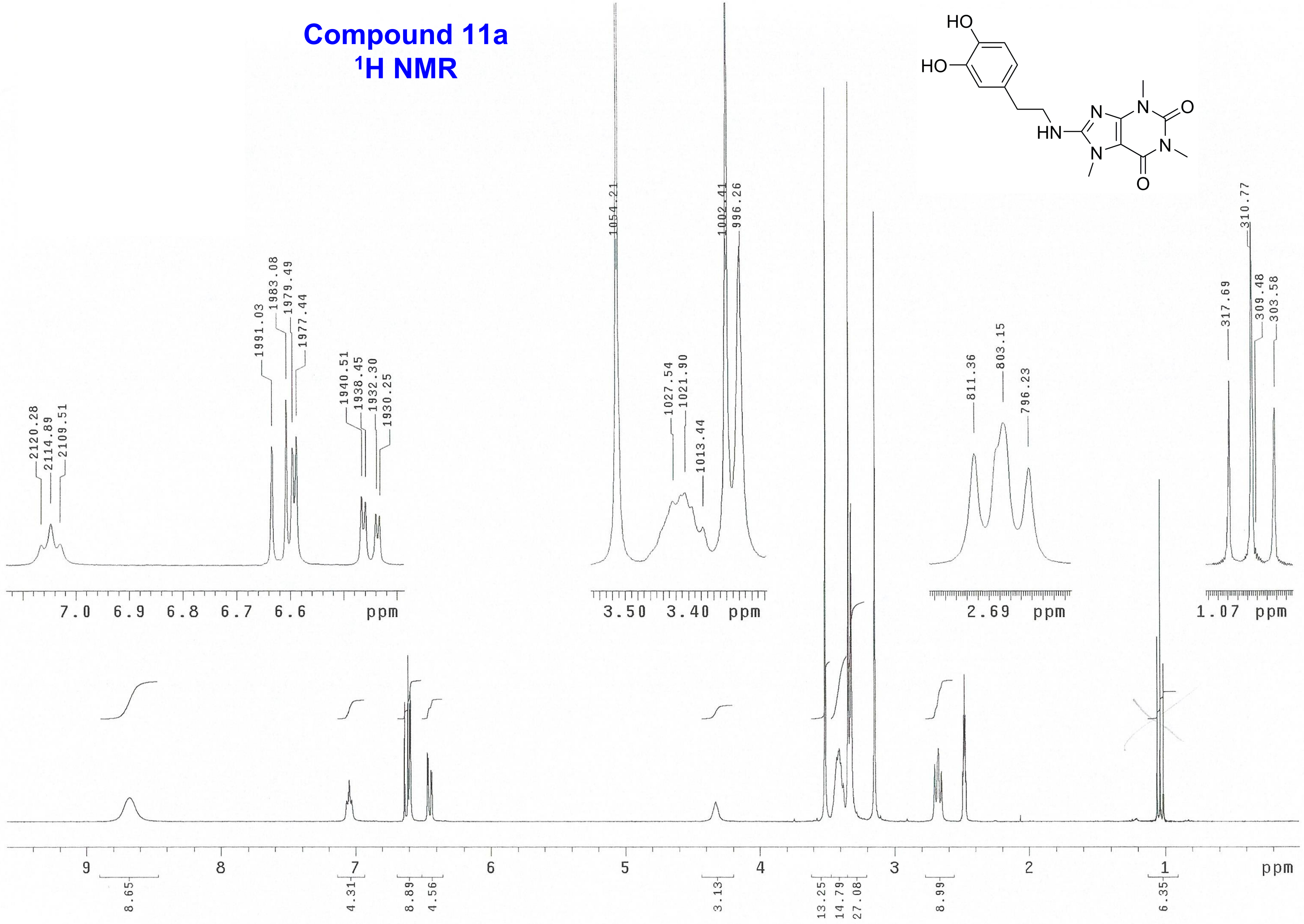

Table of peaks

INDEX	FREQUENCY	PPM	HEIGHT
1	2603.943	8.677	4.7
2	2120.278	7.066	4.0
3	2114.892	7.048	8.1
4	2109.507	7.030	4.3
5	1991.027	6.635	23.2
6	1983.077	6.608	32.3
7	1979.487	6.597	22.9
8	1977.435	6.590	25.1
9	1940.506	6.467	13.5
10	1938.455	6.460	12.4
11	1932.300	6.439	10.0
12	1930.248	6.432	9.7
13	1300.150	4.333	3.8
14	1054.214	3.513	142.8
15	1027.543	3.424	12.6
16	1023.953	3.412	13.8
17	1021.902	3.405	14.2
18	1018.568	3.394	11.5
19	1013.439	3.377	7.5
20	1002.411	3.340	144.0
21	996.257	3.320	62.1
22	943.941	3.146	118.7
23	811.356	2.704	11.2
24	803.150	2.676	14.3
25	796.225	2.653	9.8
26	748.526	2.494	9.7
27	746.730	2.488	20.8
28	744.935	2.482	28.7
29	742.884	2.476	20.7
30	741.089	2.470	9.8
31	317.689	1.059	36.0
32	310.765	1.036	66.7
33	309.483	1.031	3.4
34	303.584	1.012	30.7

Compound 11a
^{13}C NMR

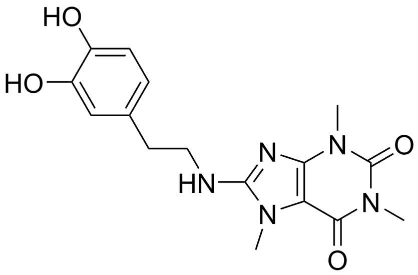

No.	(ppm)	(Hz)	Height
1	27.60	2777.1	0.1238
2	29.68	2986.9	0.1599
3	30.13	3032.4	0.1594
4	35.25	3547.4	0.0990
5	39.35	3959.7	0.1340
6	39.56	3980.9	0.4267
7	39.77	4002.2	0.8393
8	39.97	4022.8	1.0000
9	40.19	4044.0	0.8563
10	40.39	4064.6	0.4174
11	40.60	4085.8	0.1362
12	44.96	4524.5	0.1037
13	102.26	10290.6	0.0806
14	115.96	11669.8	0.1300
15	116.52	11725.5	0.1335
16	119.75	12051.2	0.1287
17	130.56	13138.4	0.1040
18	144.01	14491.9	0.1082
19	145.52	14644.5	0.1294
20	148.83	14977.5	0.0658
21	151.41	15236.5	0.0783
22	153.30	15427.2	0.0756
23	154.40	15537.3	0.0949

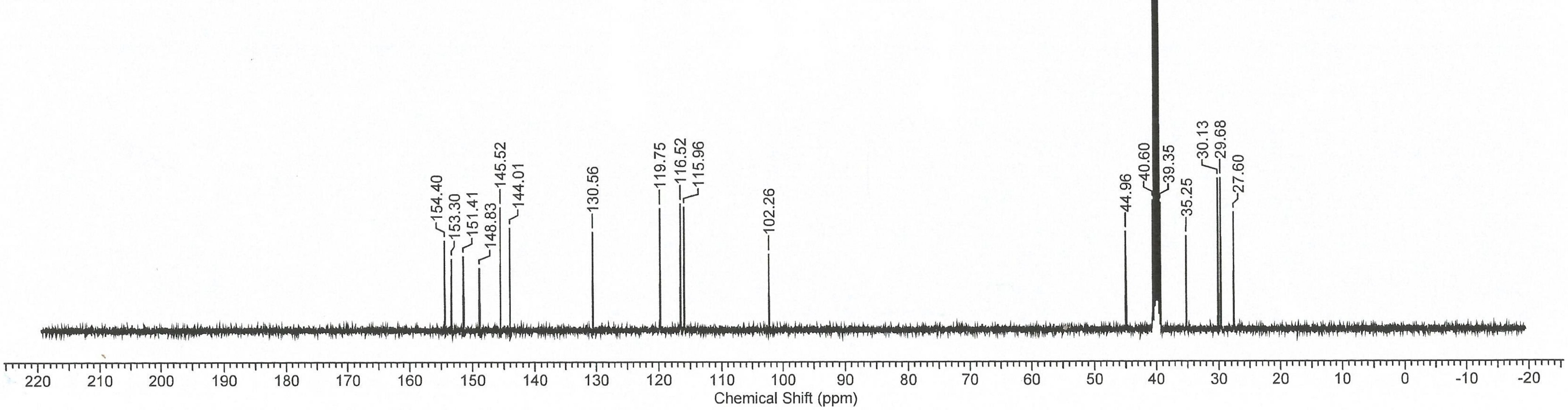

Compound 11b
^{1}H NMR - zoom of the aliphatic region

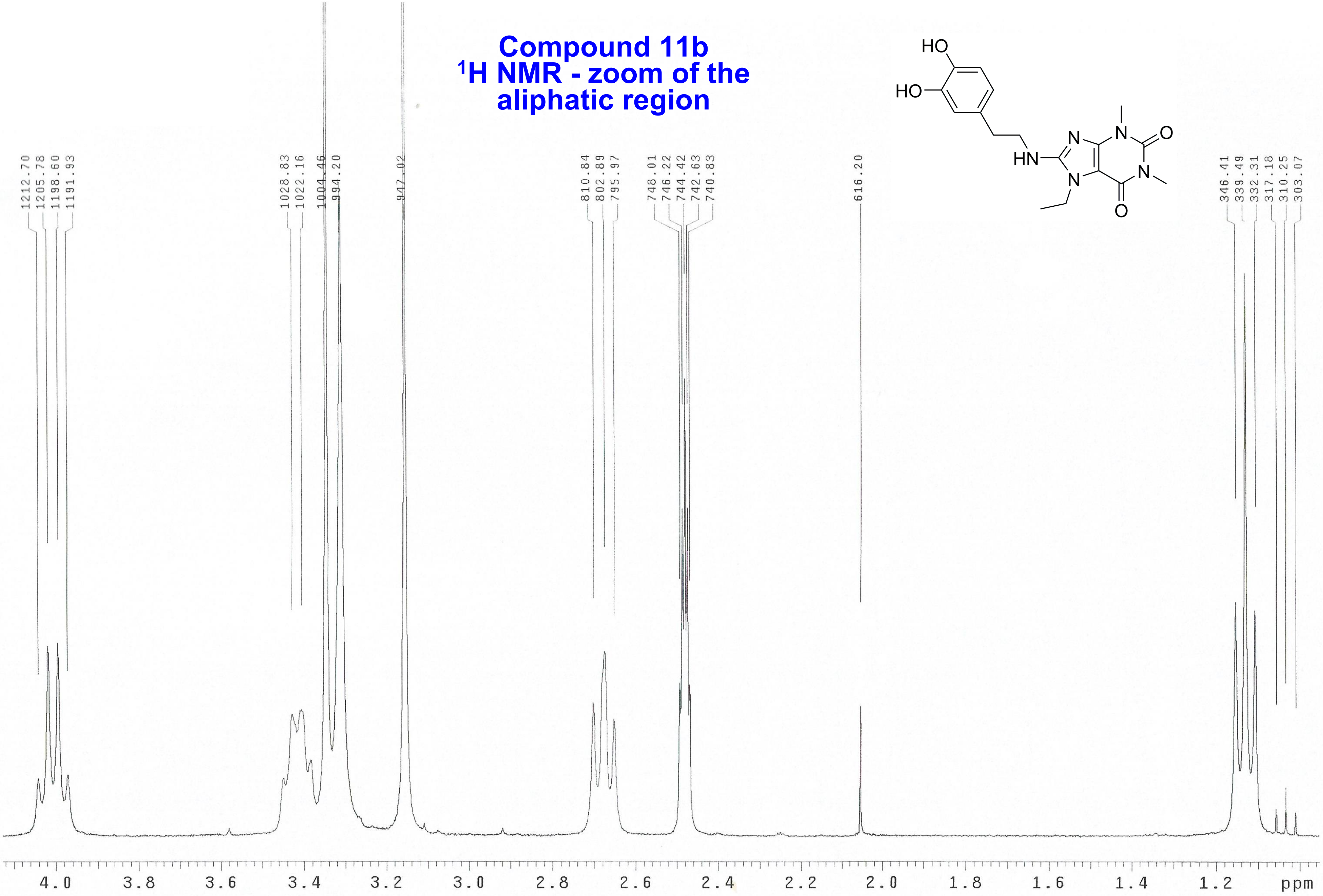

Compound 11b
¹H NMR - zoom of the aromatic region

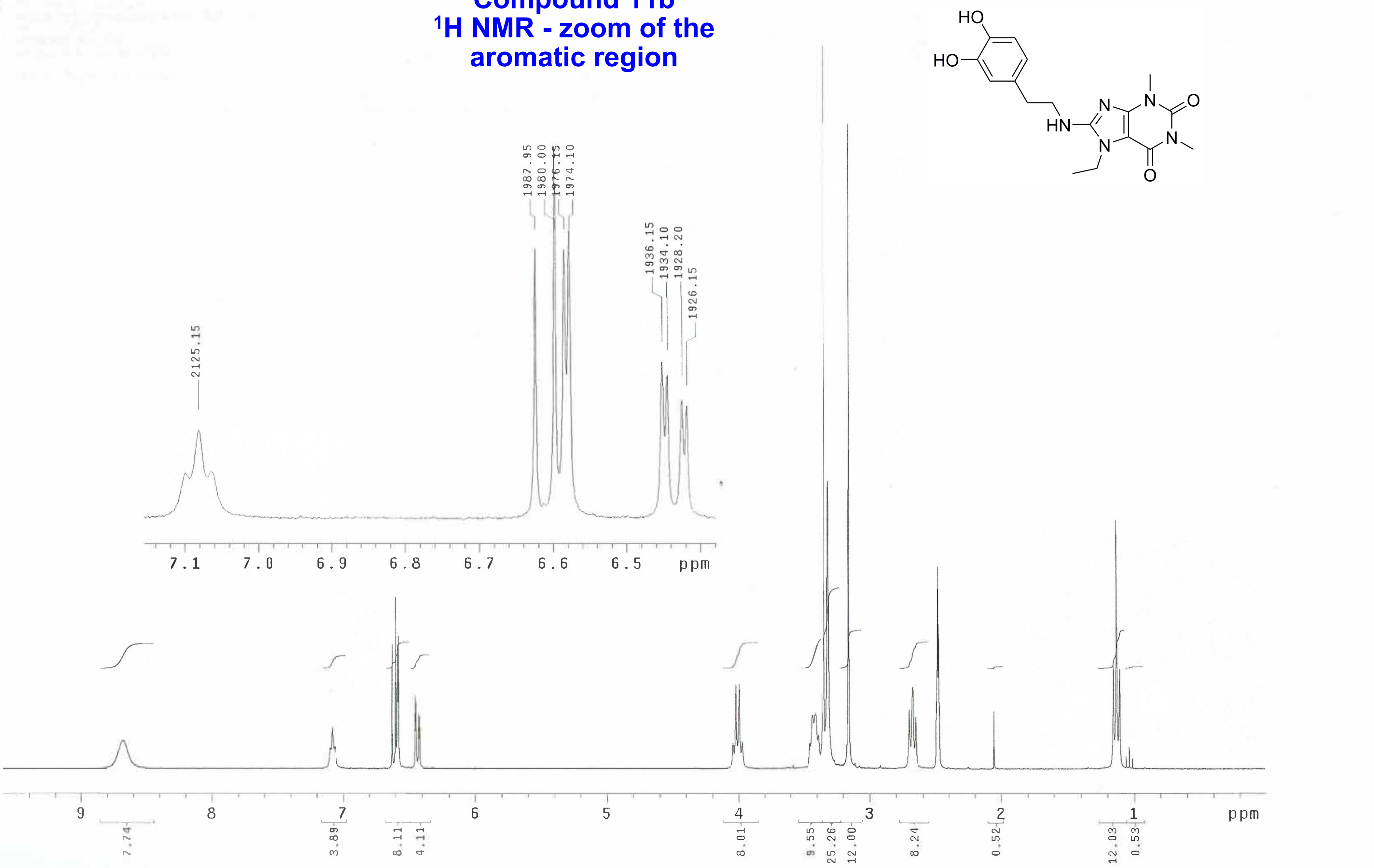

Table of peaks

INDEX	FREQUENCY	PPM	HEIGHT
1	2603.943	8.677	5.6
2	2130.536	7.100	4.2
3	2125.150	7.082	8.1
4	2120.021	7.065	4.3
5	1987.950	6.625	24.7
6	1980.000	6.598	34.1
7	1976.153	6.585	24.6
8	1974.101	6.579	26.4
9	1936.147	6.452	14.4
10	1934.095	6.445	13.2
11	1928.197	6.426	10.9
12	1926.145	6.419	10.3
13	1212.701	4.041	5.1
14	1205.776	4.018	16.7
15	1198.596	3.994	17.0
16	1191.928	3.972	5.4
17	1035.237	3.450	5.1
18	1028.826	3.428	10.7
19	1022.158	3.406	11.1
20	1015.490	3.384	6.7
21	1004.463	3.347	144.0
22	994.205	3.313	57.4
23	947.018	3.156	128.4
24	810.843	2.702	11.7
25	802.893	2.676	16.3
26	795.969	2.653	10.3
27	748.013	2.493	13.5
28	746.218	2.487	28.9
29	744.422	2.481	40.3
30	742.627	2.475	28.9
31	740.832	2.469	13.3
32	616.197	2.053	11.4
33	346.412	1.154	20.6
34	339.487	1.131	49.6
35	332.307	1.107	19.9
36	310.252	1.034	4.3

Compound 11b
^{13}C NMR

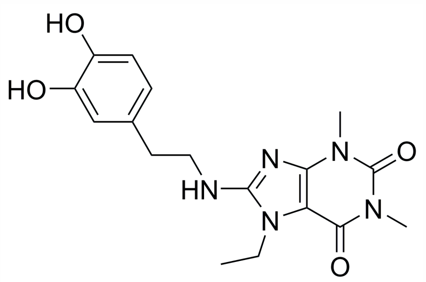

No.	(ppm)	(Hz)	Height
1	15.20	1530.0	0.1630
2	27.65	2782.2	0.1341
3	29.70	2989.1	0.1656
4	35.17	3539.3	0.1102
5	37.90	3814.4	0.0806
6	39.35	3959.7	0.1373
7	39.56	3980.9	0.4177
8	39.76	4001.5	0.8423
9	39.97	4022.8	1.0000
10	40.19	4044.0	0.8266
11	40.39	4064.6	0.4251
12	40.60	4085.8	0.1411
13	44.89	4517.2	0.1091
14	101.45	10209.2	0.0952
15	115.92	11665.3	0.1346
16	116.57	11730.6	0.1397
17	119.80	12055.6	0.1379
18	130.60	13142.8	0.1127
19	143.99	14489.7	0.1181
20	145.52	14643.7	0.1509
21	149.14	15008.3	0.0868
22	151.45	15240.9	0.0865
23	153.05	15401.6	0.0951
24	153.43	15440.4	0.1005

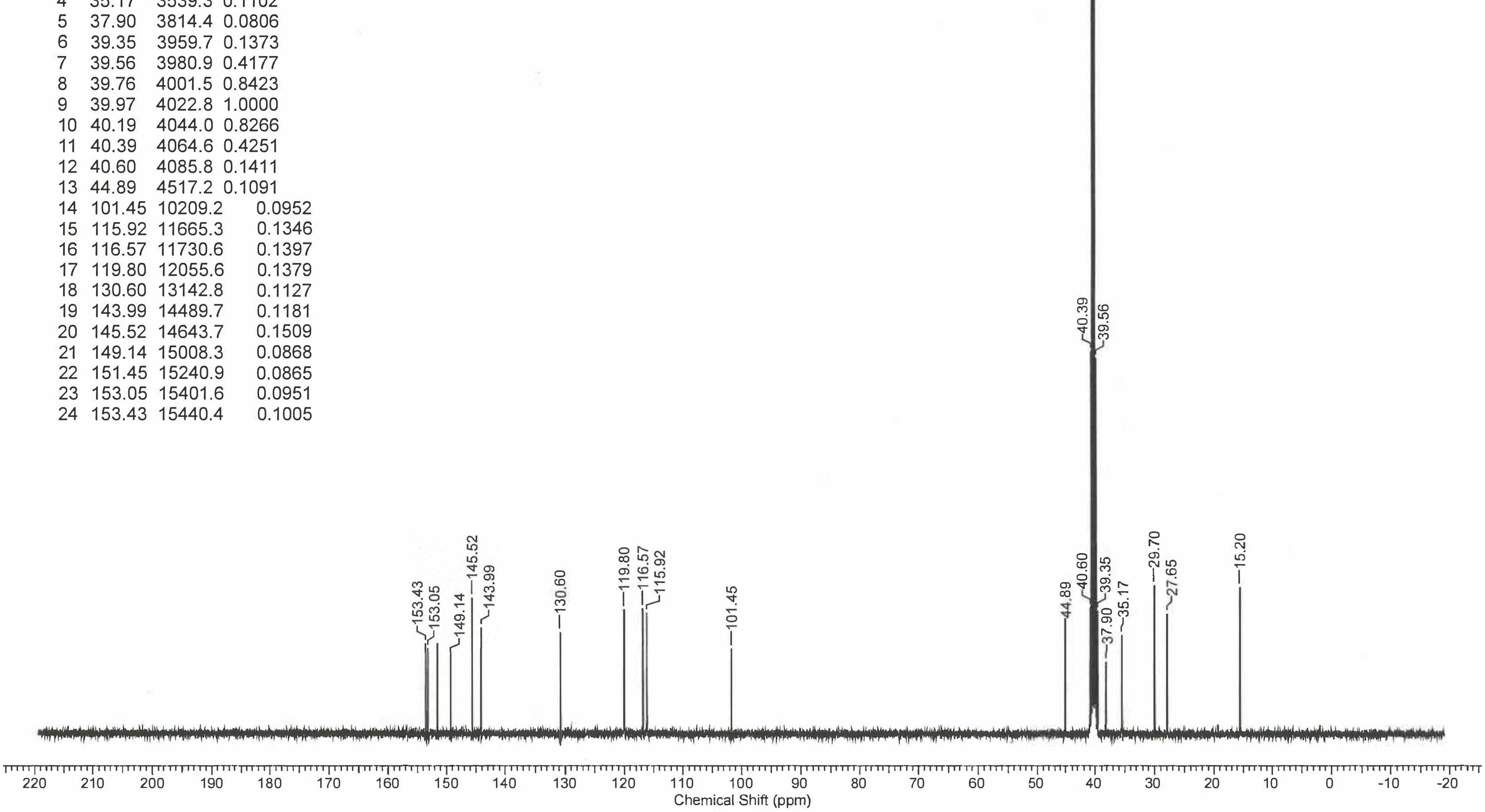

Compound 11c
^{1}H NMR

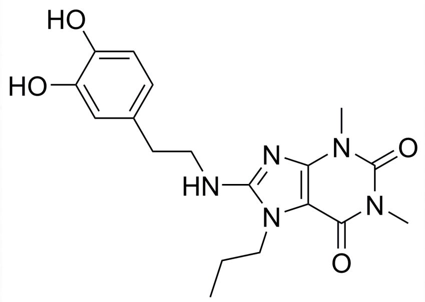

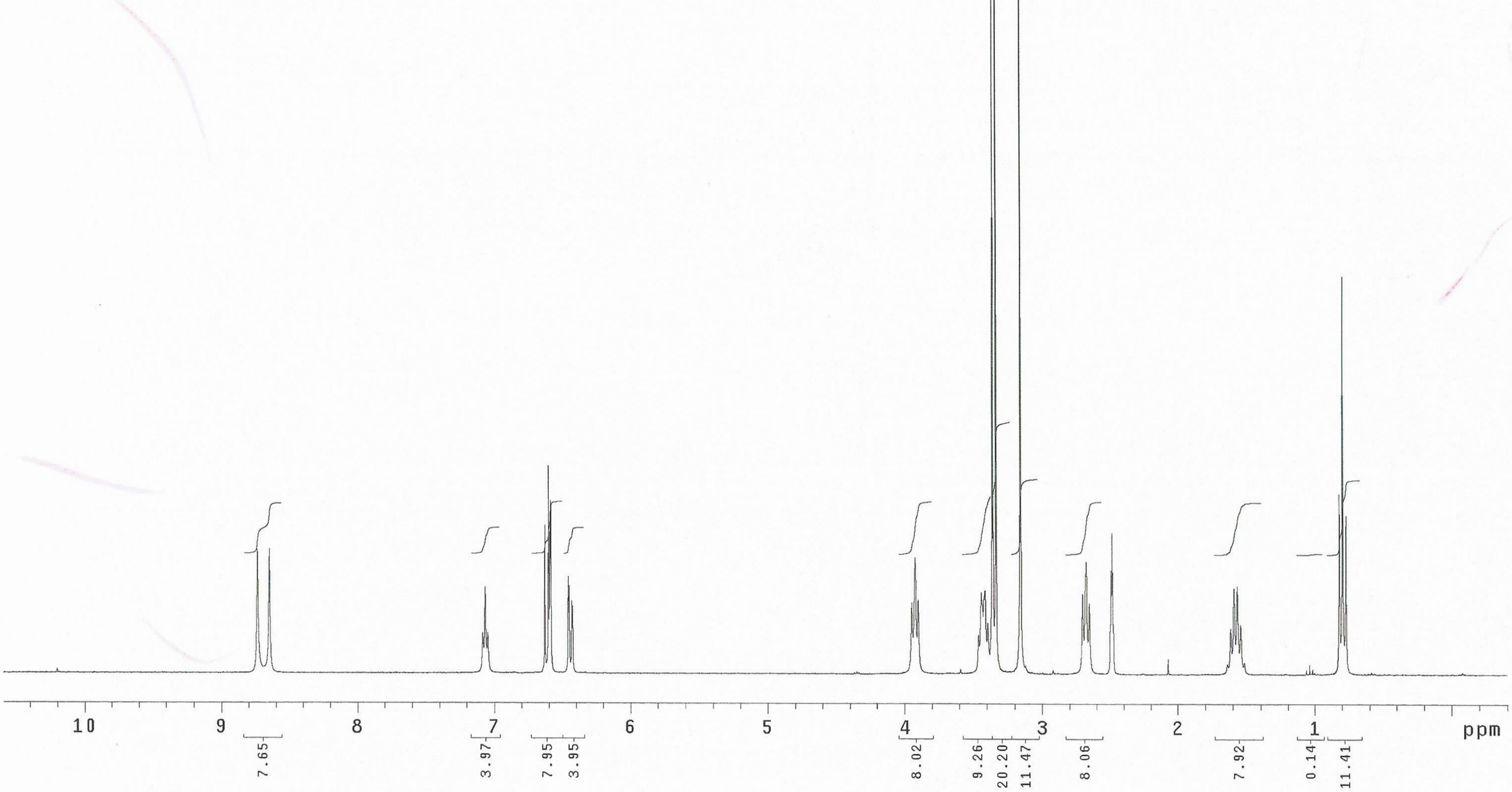

Compound 11c
^{1}H NMR - zoom of the aliphatic region

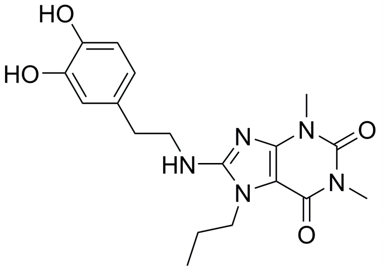

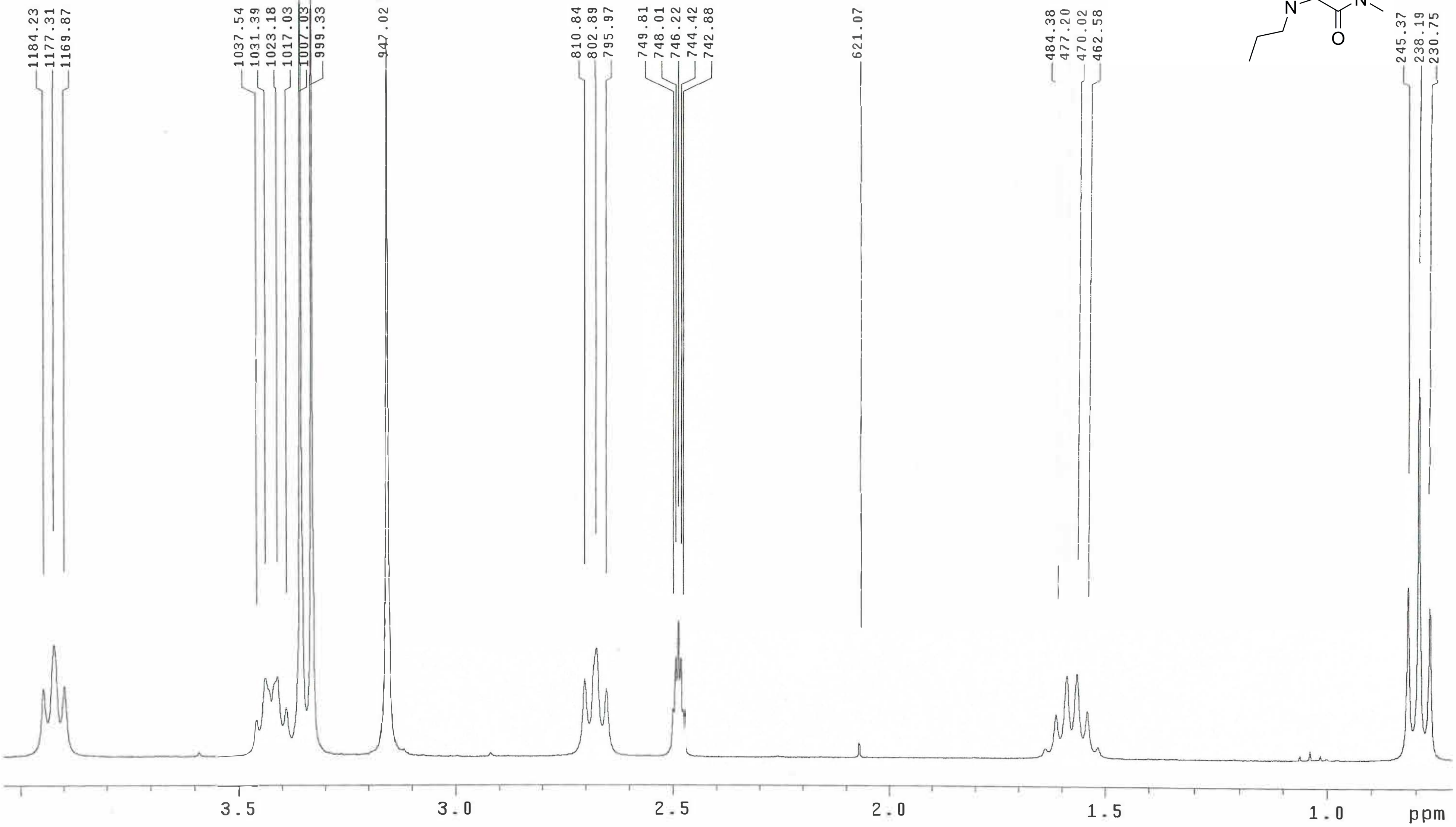

Compound 11c
^{1}H NMR - zoom of the aromatic region

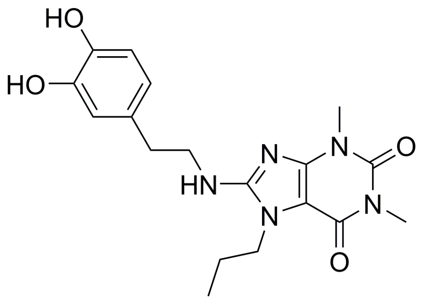

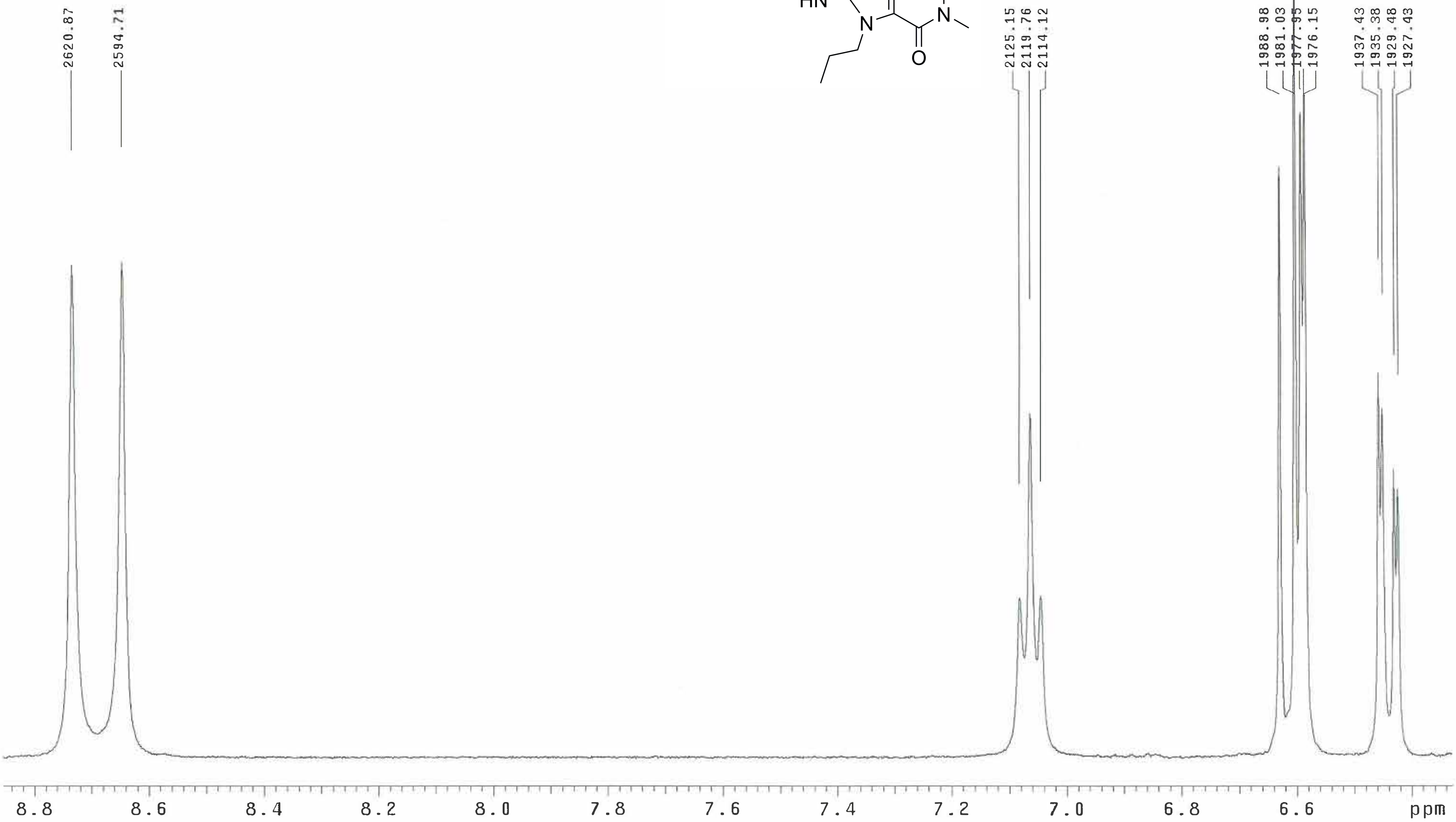

Table of peaks

INDEX	FREQUENCY	PPM	HEIGHT
1	2620.869	8.734	20.6
2	2594.711	8.647	20.8
3	2125.150	7.082	6.7
4	2119.765	7.064	14.4
5	2114.123	7.045	6.8
6	1988.975	6.628	24.8
7	1981.025	6.602	34.7
8	1977.948	6.591	27.0
9	1976.153	6.585	28.8
10	1937.429	6.456	16.1
11	1935.377	6.450	14.6
12	1929.479	6.430	12.1
13	1927.427	6.423	11.3
14	1184.235	3.946	12.0
15	1177.310	3.923	19.5
16	1169.873	3.899	12.4
17	1037.545	3.458	6.5
18	1031.390	3.437	13.6
19	1023.184	3.410	14.0
20	1017.029	3.389	8.5
21	1007.027	3.356	144.0
22	999.334	3.330	132.1
23	947.018	3.156	124.8
24	810.843	2.702	13.4
25	802.893	2.676	18.8
26	795.969	2.653	11.8
27	749.808	2.499	8.4
28	748.013	2.493	17.4
29	746.218	2.487	23.6
30	744.422	2.481	17.1
31	742.884	2.476	8.2
32	621.070	2.070	2.5
33	484.382	1.614	7.7
34	477.201	1.590	14.4
35	470.021	1.566	14.8
36	462.584	1.542	8.3
37	245.370	0.818	30.2
38	238.190	0.794	66.8
39	230.753	0.769	26.6

INDEX	FREQUENCY	PPM	HEIGHT

Compound 11c
^{13}C NMR

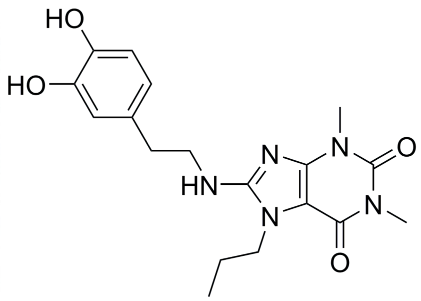

No.	(ppm)	(Hz)	Height
1	14.31	1439.8	0.1394
2	22.27	2241.6	0.1254
3	27.65	2782.2	0.1055
4	28.36	2854.1	0.1136
5	29.19	2937.8	0.0825
6	29.68	2986.9	0.1085
7	35.27	3548.8	0.0611
8	39.37	3961.9	0.1362
9	39.58	3983.1	0.4205
10	39.78	4003.7	0.8399
11	40.00	4025.0	1.0000
12	40.21	4046.2	0.8259
13	40.41	4066.8	0.4225
14	40.62	4088.0	0.1367
15	42.78	4305.2	0.0494
16	44.62	4490.1	0.0650
17	55.82	5616.9	0.0941
18	55.98	5633.0	0.0933
19	101.83	10247.3	0.0522
20	112.34	11305.2	0.0721
21	113.10	11381.4	0.0735
22	121.07	12183.3	0.0809
23	132.37	13321.1	0.0668
24	147.72	14865.3	0.0492
25	149.00	14994.4	0.0568
26	149.10	15003.9	0.0630
27	151.44	15239.4	0.0606
28	153.04	15400.8	0.0514
29	153.69	15466.1	0.0593

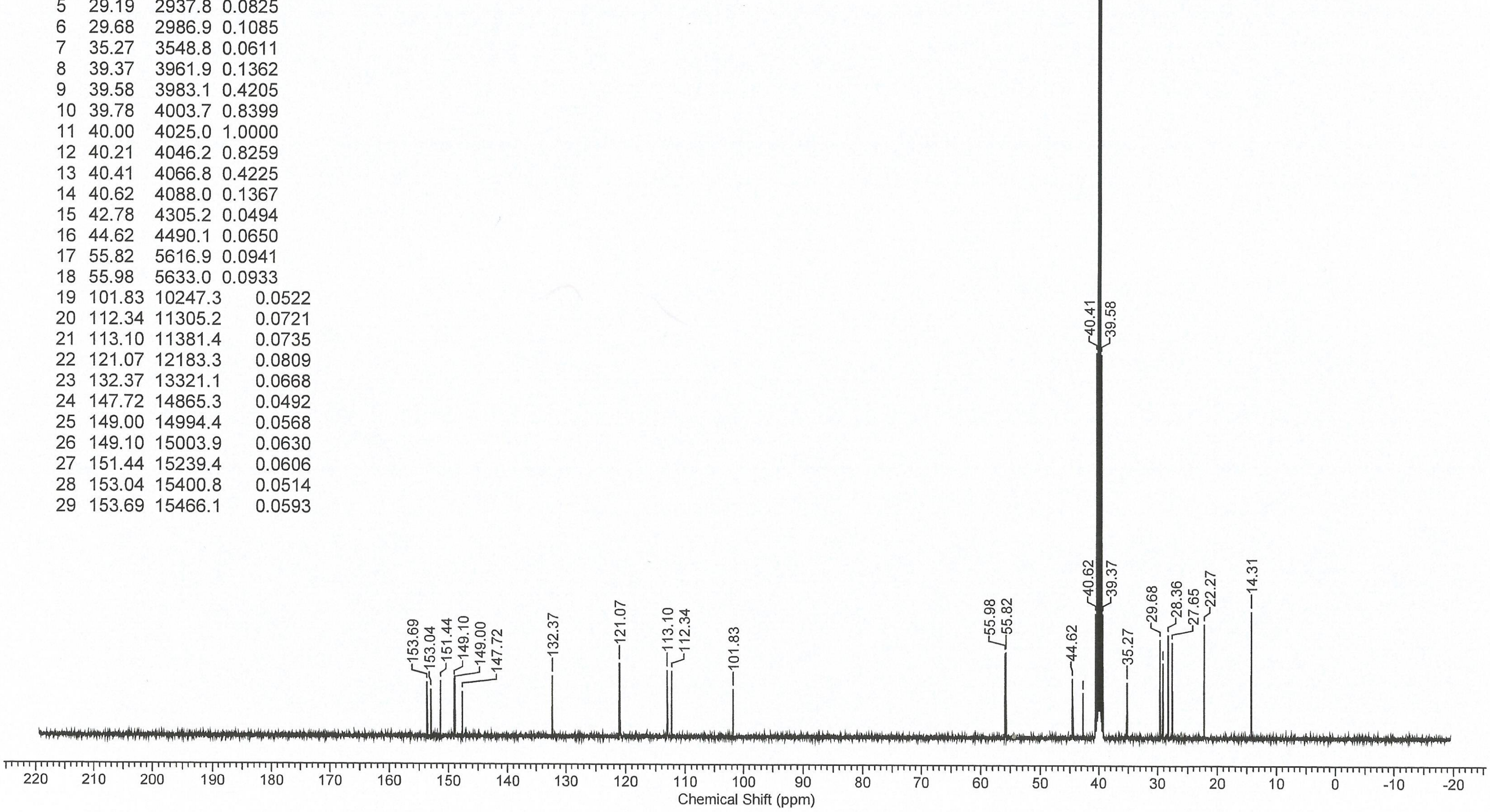

Compound 11d
^{1}H NMR

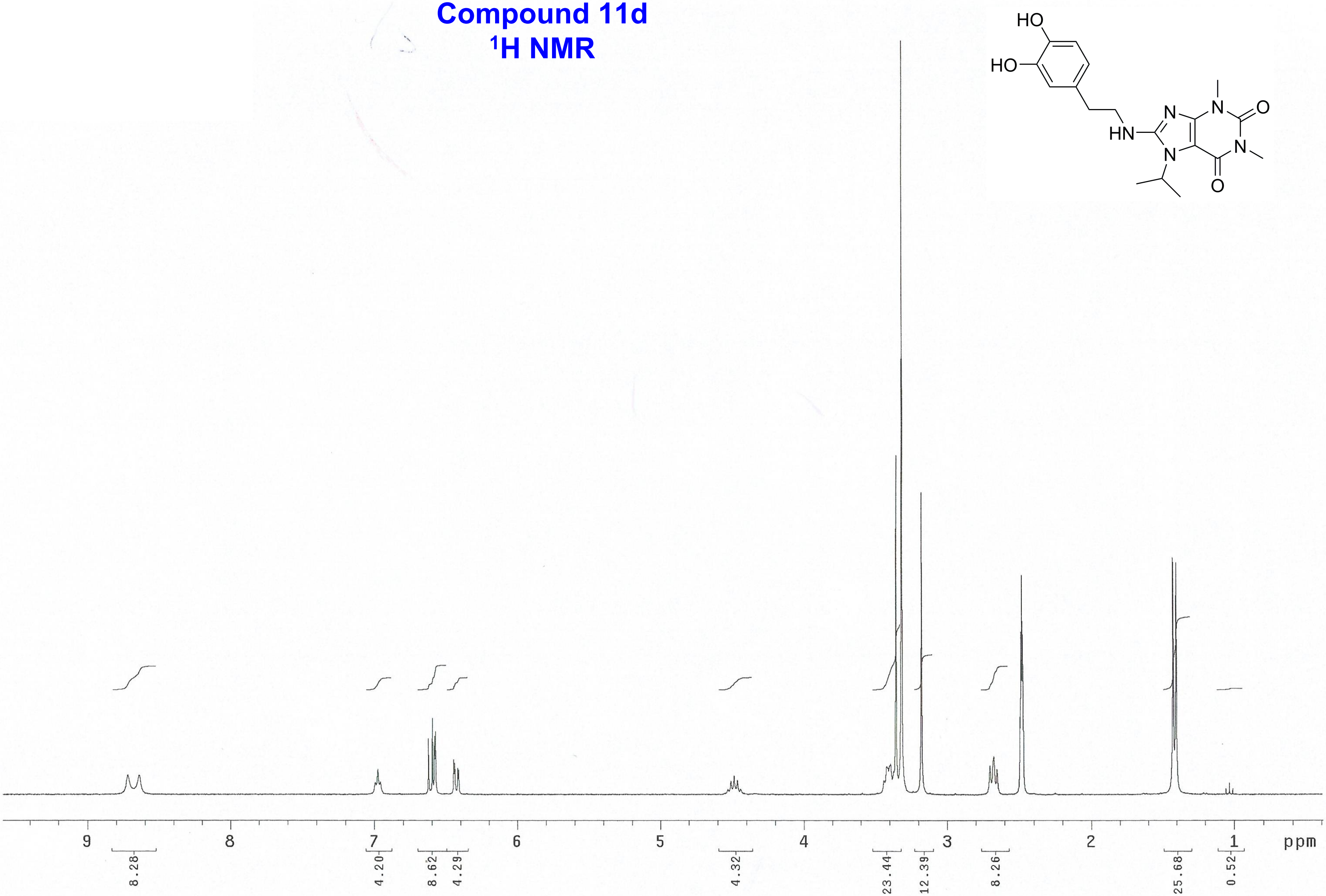

Compound 11d
^{1}H NMR - zoom of the aliphatic region

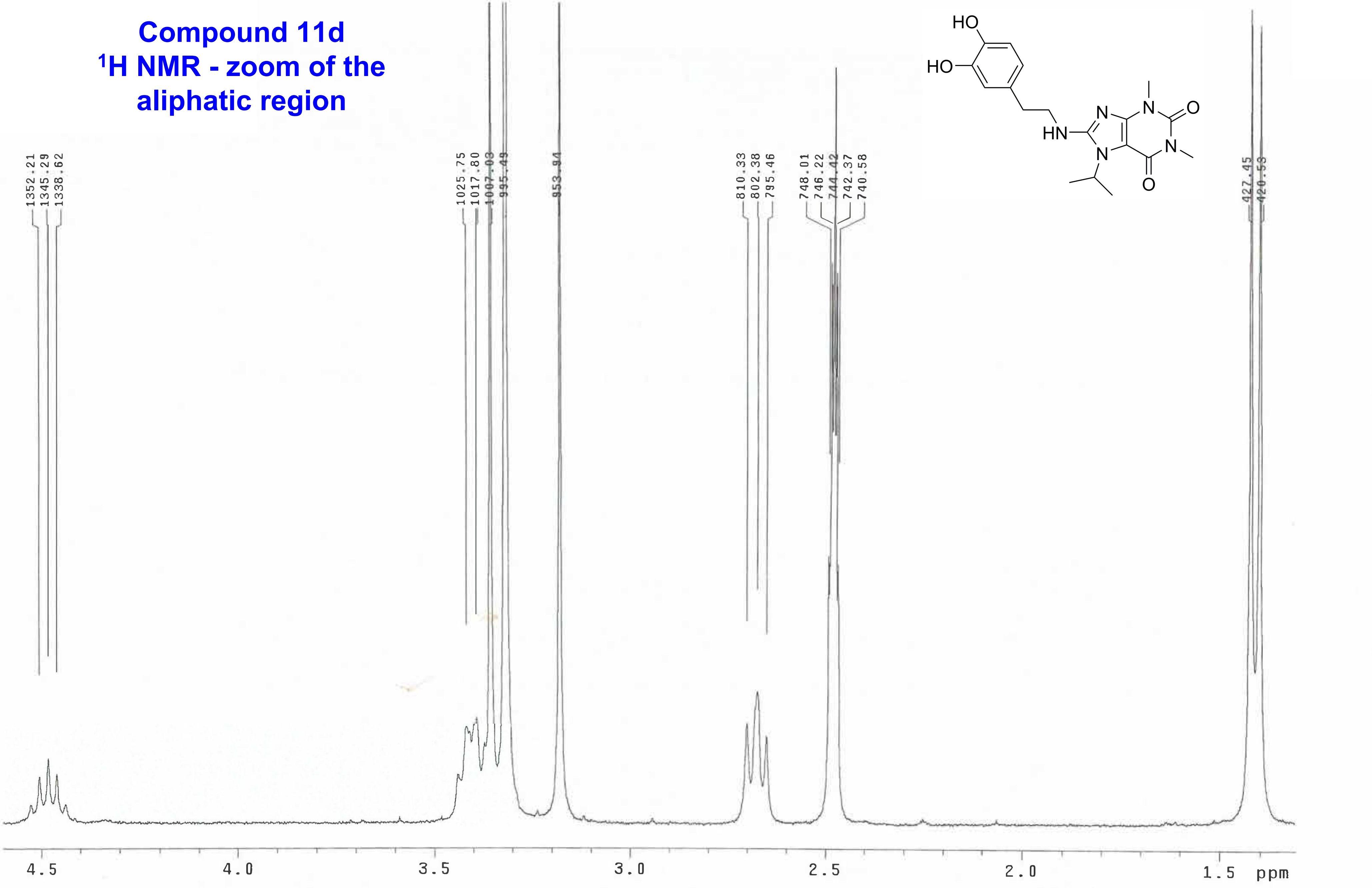

Compound 11d
^{1}H NMR - zoom of the aromatic region

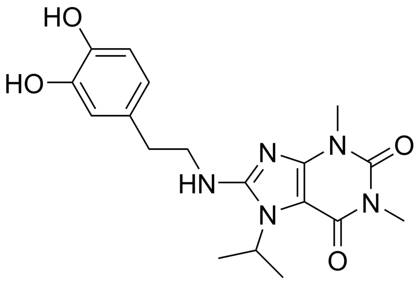

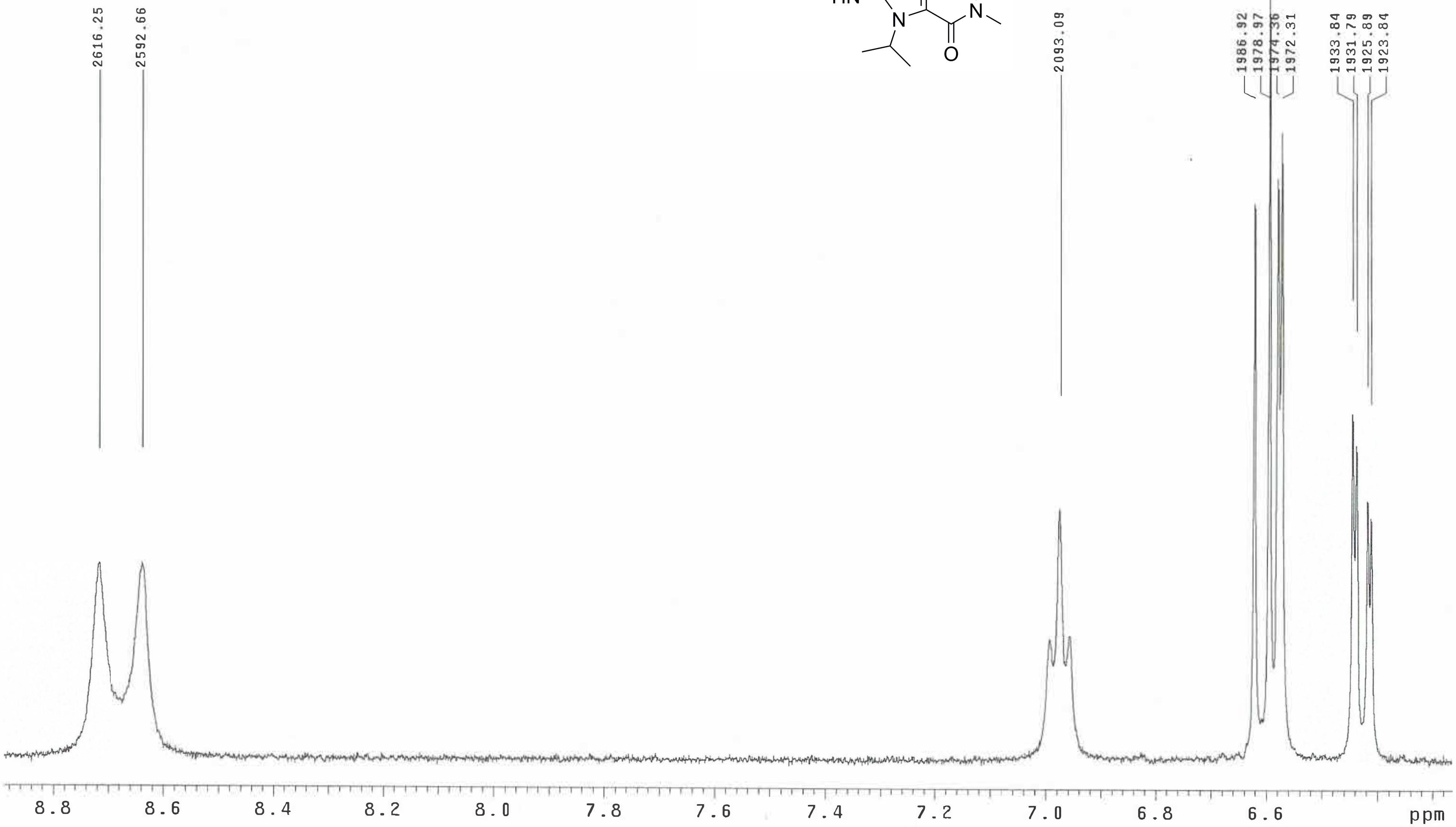

Table of peaks

INDEX	FREQUENCY	PPM	HEIGHT
1	2616.253	8.719	3.7
2	2592.659	8.640	3.7
3	2093.094	6.975	4.8
4	1986.924	6.621	10.7
5	1978.974	6.595	14.6
6	1974.358	6.579	11.1
7	1972.306	6.573	12.0
8	1933.839	6.444	6.7
9	1931.787	6.438	6.0
10	1925.889	6.418	5.0
11	1923.837	6.411	4.7
12	1352.209	4.506	2.6
13	1345.285	4.483	3.6
14	1338.618	4.461	2.7
15	1032.672	3.441	2.7
16	1025.748	3.418	5.3
17	1017.798	3.392	5.9
18	1011.900	3.372	4.4
19	1007.027	3.356	64.9
20	995.487	3.317	144.0
21	953.942	3.179	57.9
22	810.330	2.700	5.5
23	802.380	2.674	7.3
24	795.456	2.651	4.8
25	748.013	2.493	14.8
26	746.218	2.487	31.1
27	744.422	2.481	41.9
28	742.371	2.474	30.6
29	740.576	2.468	14.4
30	427.450	1.424	45.3
31	420.526	1.401	44.4

Compound 11d
^{13}C NMR

No.	(ppm)	(Hz)	Height
1	21.55	2168.2	0.2579
2	28.15	2832.9	0.1122
3	29.76	2995.0	0.1402
4	35.13	3535.6	0.0863
5	39.35	3960.4	0.1334
6	39.56	3980.9	0.4286
7	39.77	4002.2	0.8651
8	39.97	4022.8	1.0000
9	40.19	4044.0	0.8754
10	40.40	4065.3	0.4338
11	40.60	4085.8	0.1454
12	45.16	4544.3	0.0832
13	46.52	4681.5	0.0846
14	49.06	4936.8	0.0438
15	101.84	10248.8	0.0639
16	115.90	11663.1	0.1037
17	116.58	11731.4	0.1049
18	119.81	12056.4	0.1058
19	130.63	13145.7	0.0760
20	143.97	14488.2	0.0943
21	145.51	14643.0	0.1236
22	150.19	15114.0	0.0618
23	151.38	15234.3	0.0699
24	152.71	15367.8	0.0722
25	153.21	15418.4	0.0971

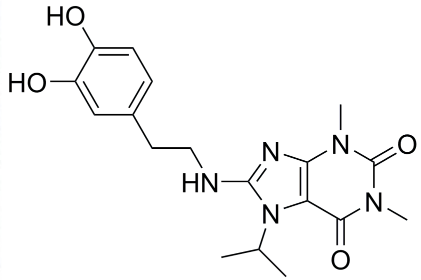

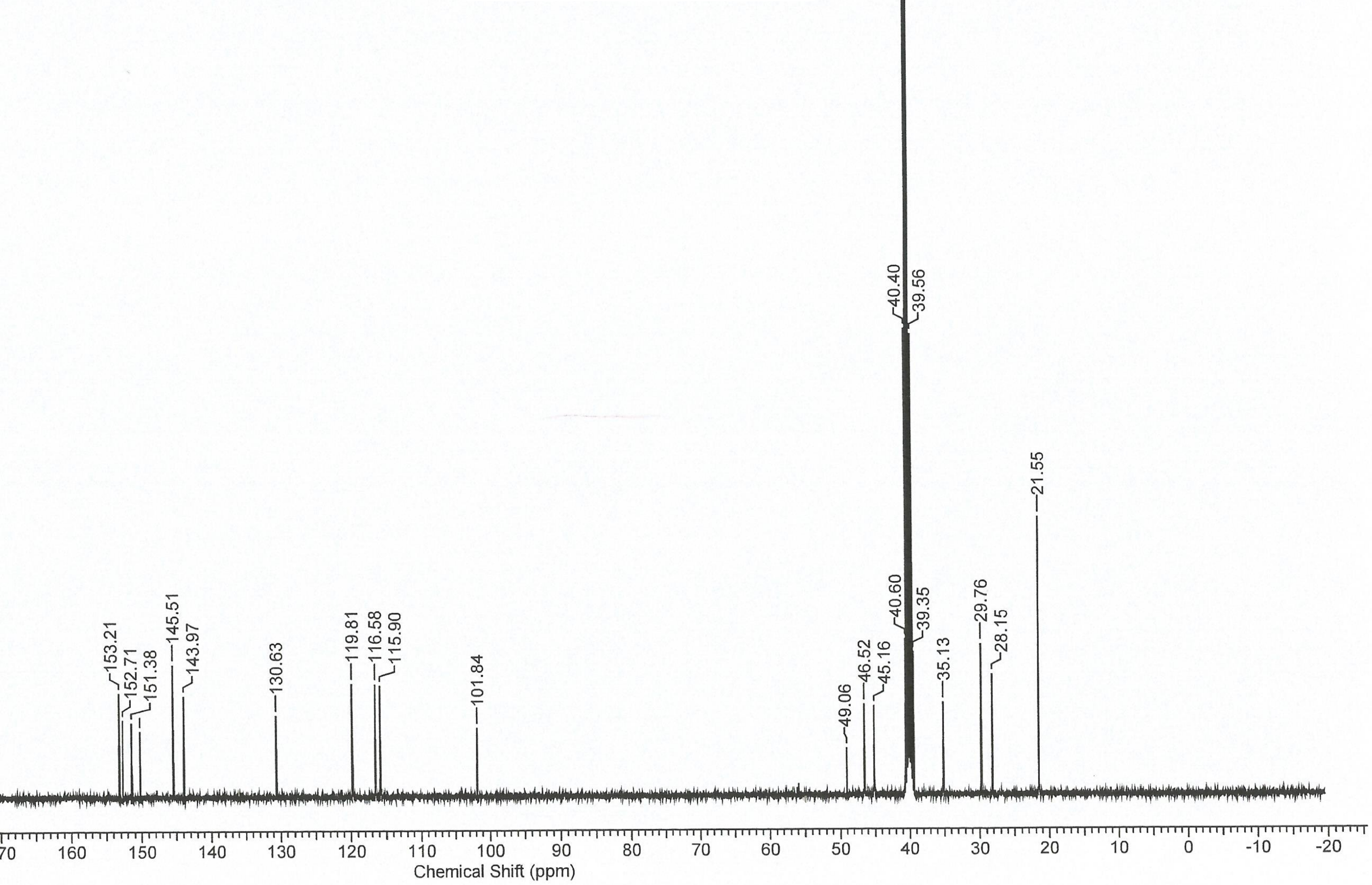

Compound 11e
^{1}H NMR - zoom of the aromatic region

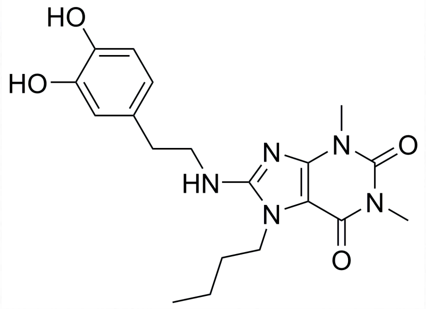

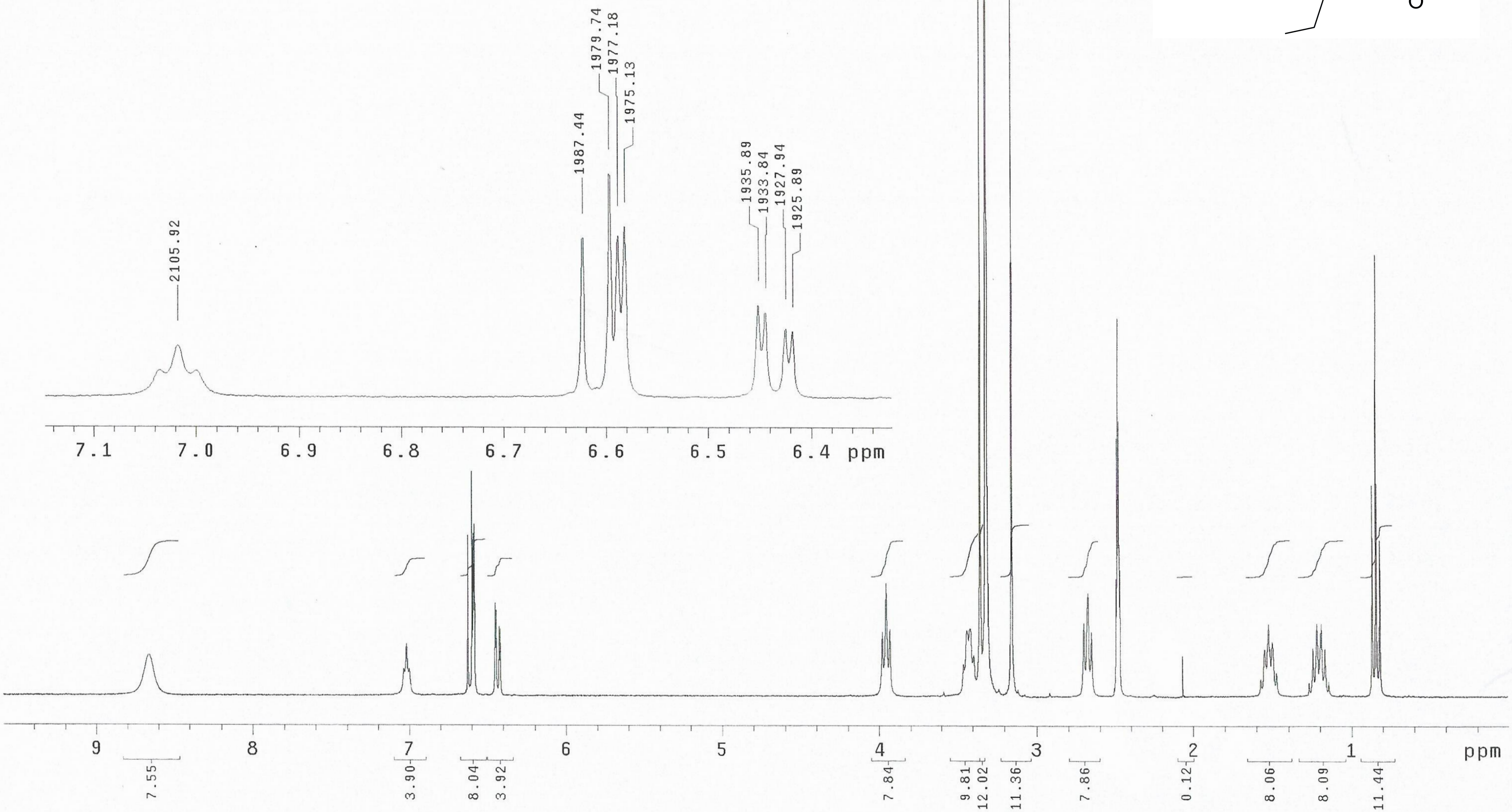

Compound 11e
^{1}H NMR - zoom of the aliphatic region

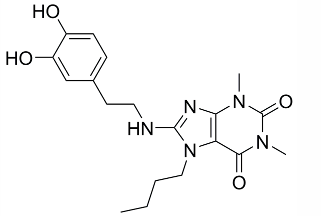

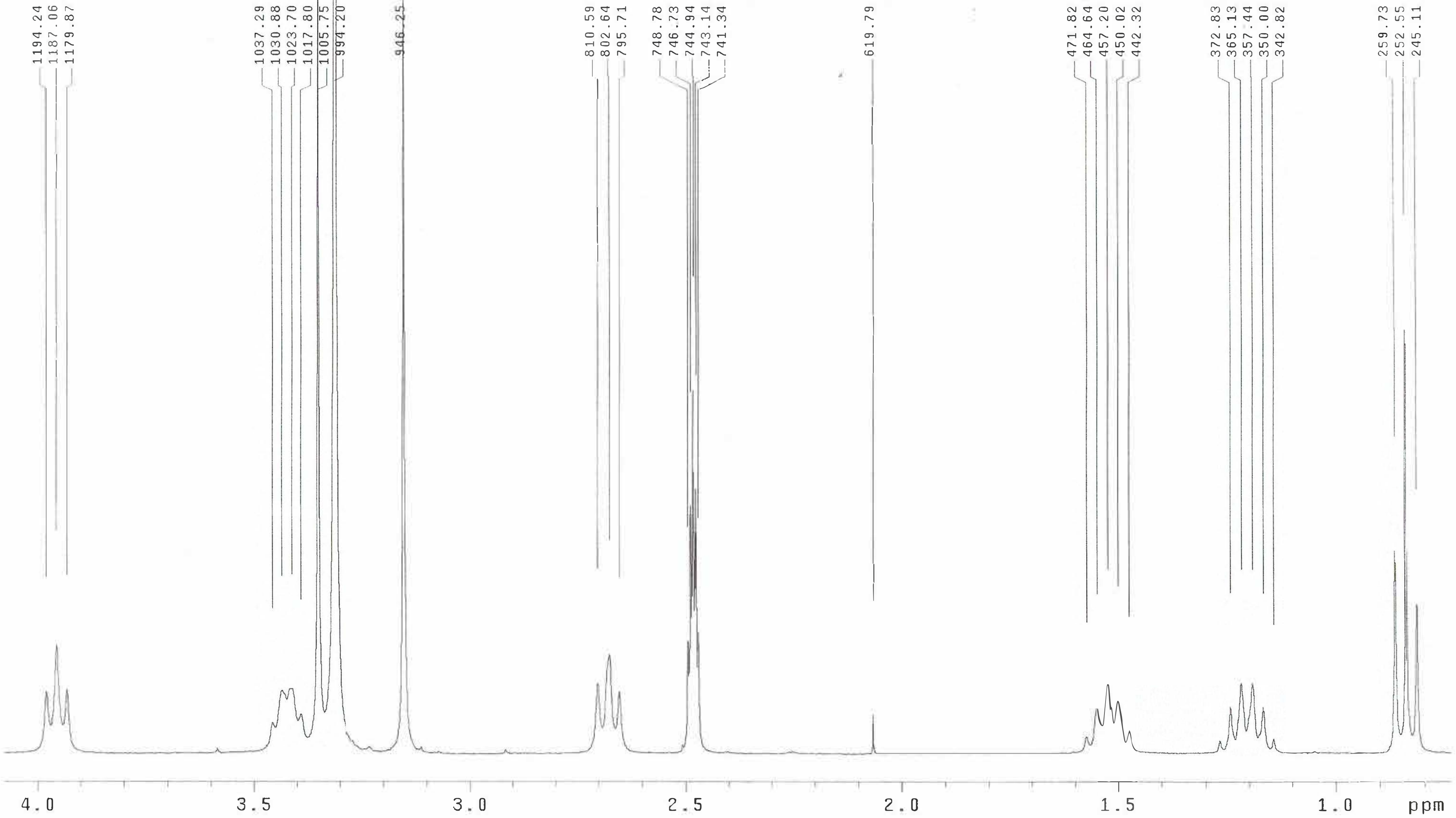

Table of peaks

INDEX	FREQUENCY	PPM	HEIGHT
1	2600.096	8.665	6.8
2	2111.046	7.035	4.5
3	2105.917	7.018	8.7
4	2100.531	7.000	4.5
5	1987.437	6.623	26.8
6	1979.743	6.597	37.5
7	1977.179	6.589	27.2
8	1975.127	6.582	28.7
9	1935.890	6.451	15.5
10	1933.839	6.444	14.3
11	1927.940	6.425	11.6
12	1925.889	6.418	11.1
13	1194.236	3.980	10.8
14	1187.056	3.956	18.9
15	1179.875	3.932	11.1
16	1037.289	3.457	5.3
17	1030.877	3.435	11.0
18	1023.697	3.411	11.3
19	1017.798	3.392	6.8
20	1005.745	3.352	154.7
21	994.205	3.313	269.8
22	946.249	3.153	147.3
23	810.587	2.701	12.3
24	802.637	2.675	17.2
25	795.712	2.652	10.7
26	748.782	2.495	19.6
27	746.730	2.488	43.3
28	744.935	2.482	63.4
29	743.140	2.476	46.1
30	741.345	2.470	21.1
31	619.788	2.065	6.7
32	471.816	1.572	2.9
33	464.635	1.548	7.9
34	457.198	1.524	12.1
35	454.634	1.515	8.0
36	450.018	1.500	9.1
37	442.324	1.474	3.9
38	372.826	1.242	8.2
39	365.132	1.217	12.2
40	357.439	1.191	12.2
41	350.002	1.166	8.0
42	342.821	1.142	2.6
43	259.731	0.866	35.4
44	252.551	0.842	74.0
45	245.114	0.817	26.1

Compound 11e
^{13}C NMR

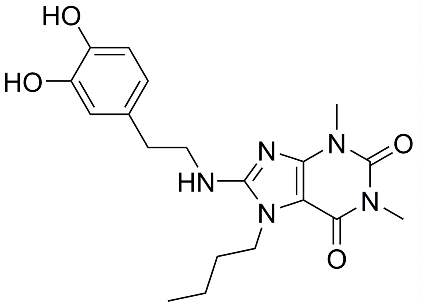

No.	(ppm)	(Hz)	Height
1	14.11	1420.0	0.1391
2	19.55	1967.2	0.1197
3	27.65	2783.0	0.0943
4	29.71	2989.8	0.1029
5	31.66	3185.7	0.0846
6	35.19	3540.8	0.0680
7	39.36	3961.1	0.1359
8	39.57	3982.4	0.4220
9	39.78	4002.9	0.8329
10	39.99	4024.2	1.0000
11	40.20	4045.5	0.8336
12	40.40	4066.0	0.4215
13	40.62	4087.3	0.1387
14	42.62	4289.0	0.0514
15	44.87	4515.7	0.0670
16	101.84	10248.8	0.0691
17	115.88	11660.9	0.0856
18	116.55	11729.2	0.0915
19	119.81	12056.4	0.0889
20	130.57	13139.9	0.0779
21	143.99	14490.4	0.0868
22	145.52	14644.5	0.0950
23	149.04	14998.8	0.0479
24	151.45	15240.9	0.0630
25	153.04	15400.8	0.0636
26	153.71	15468.3	0.0838

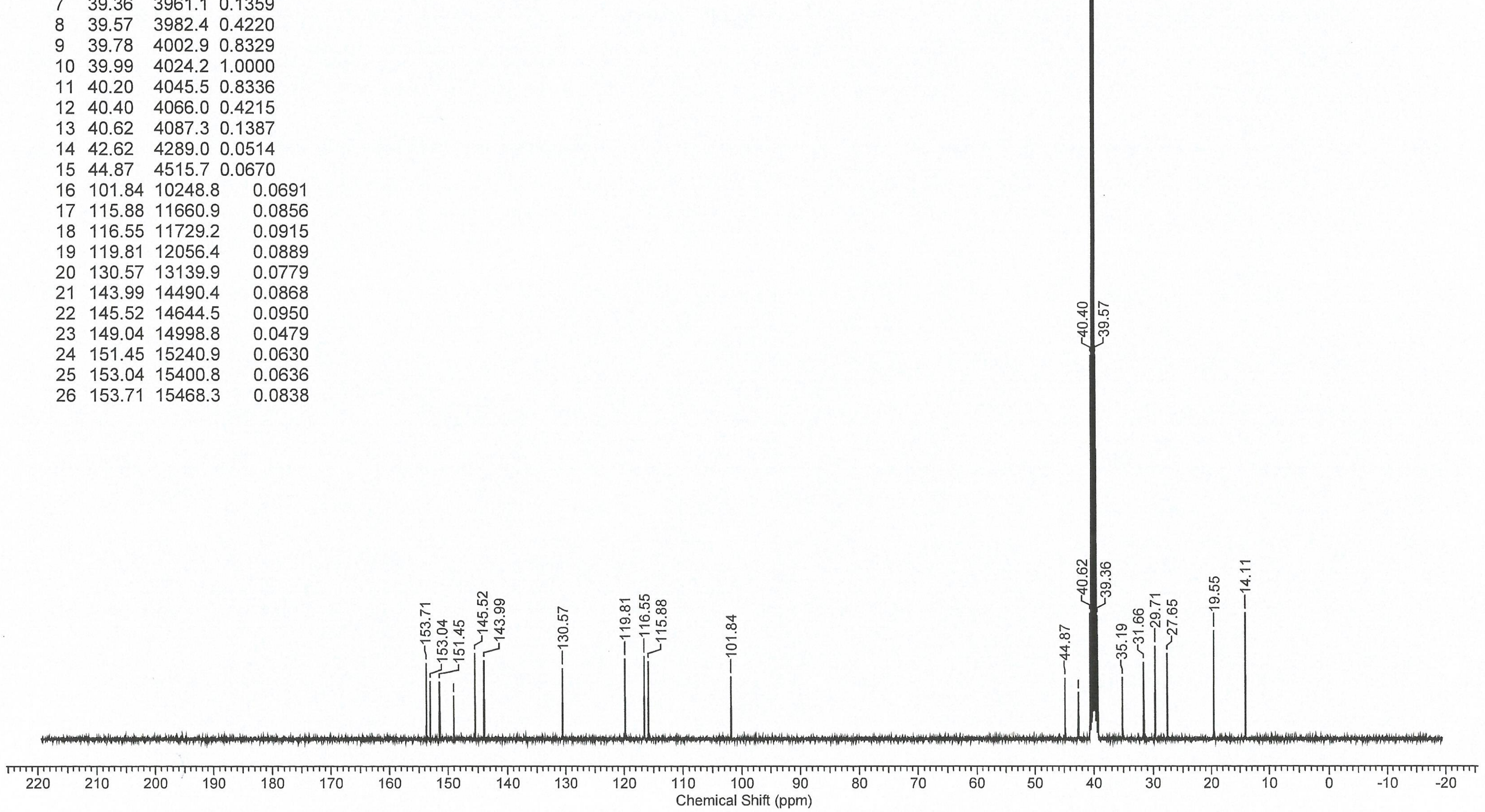

Compound 11f
^{1}H NMR - zoom of the aromatic region

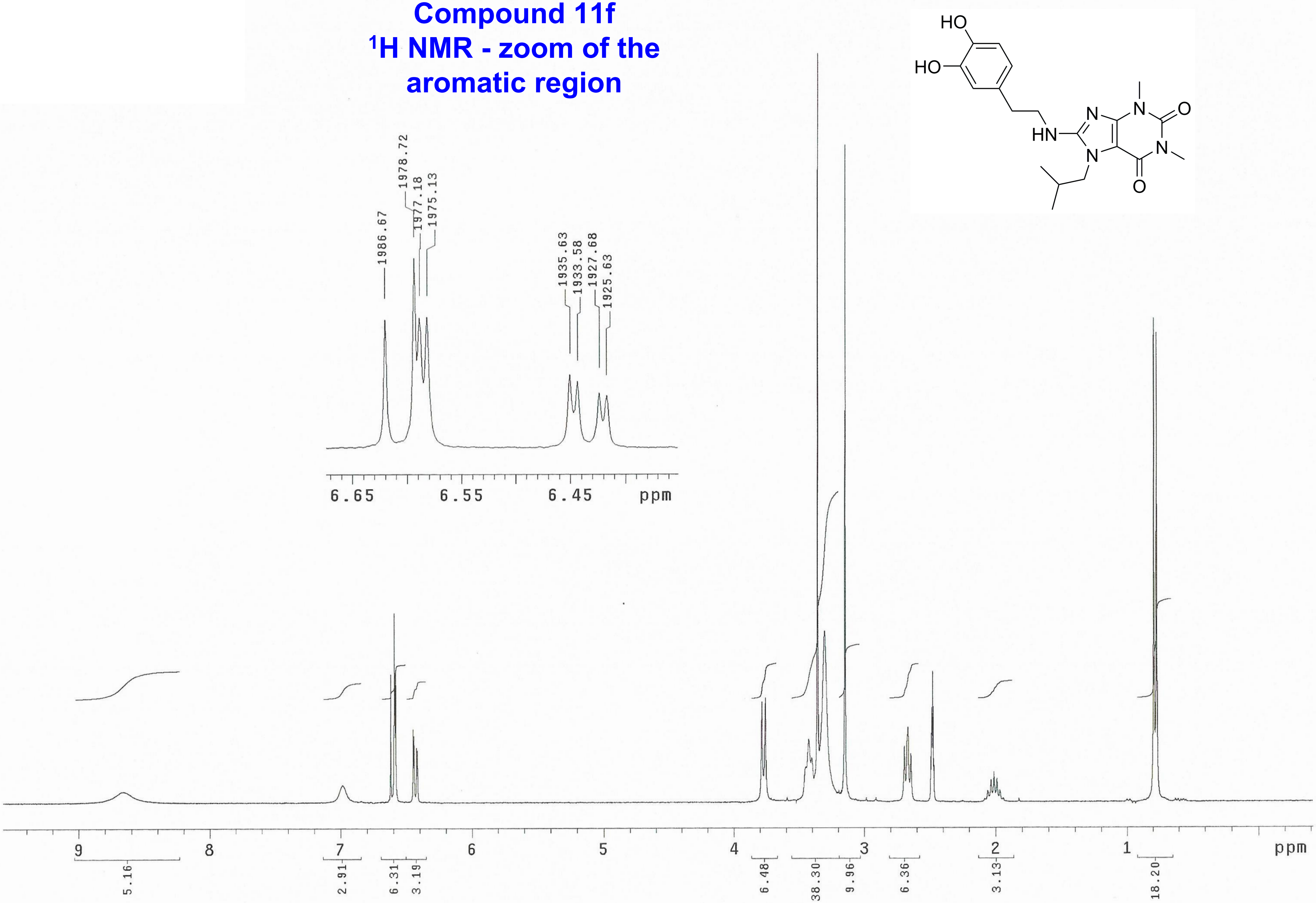

Compound 11f
^{1}H NMR - zoom of the aliphatic region

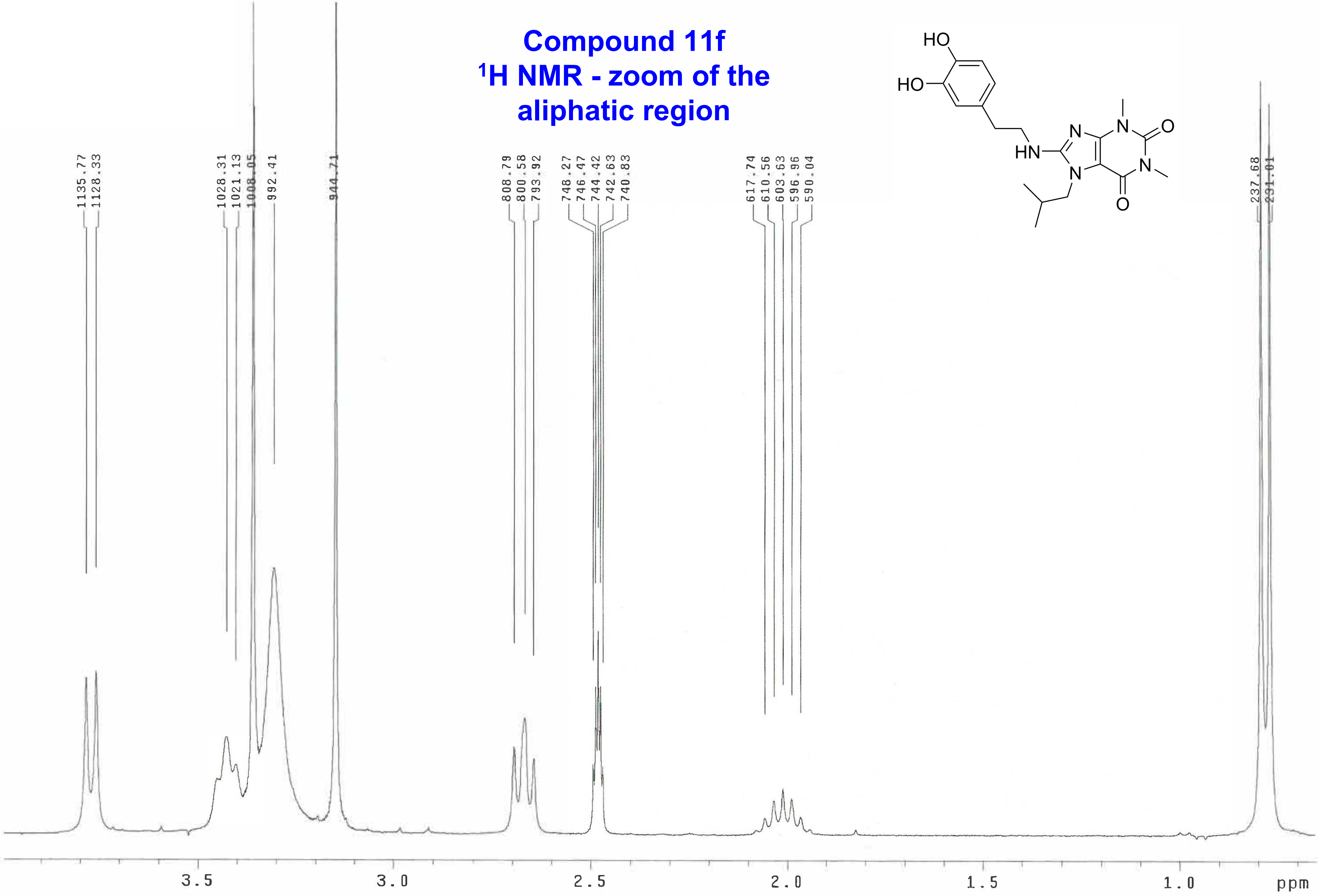

Table of peaks

INDEX	FREQUENCY	PPM	HEIGHT
1	2095.659	6.984	3.3
2	1986.667	6.620	24.7
3	1978.717	6.594	36.5
4	1977.179	6.589	25.1
5	1975.127	6.582	25.3
6	1935.634	6.450	14.2
7	1933.582	6.444	12.8
8	1927.684	6.424	10.6
9	1925.632	6.417	10.0
10	1135.765	3.785	19.3
11	1128.328	3.760	20.1
12	1035.750	3.452	6.9
13	1028.313	3.427	12.1
14	1021.132	3.403	8.5
15	1016.260	3.387	4.8
16	1015.747	3.385	4.8
17	1015.234	3.383	5.0
18	1014.208	3.380	5.4
19	1013.695	3.378	5.5
20	1008.053	3.359	144.0
21	1004.463	3.347	10.7
22	1003.950	3.346	10.4
23	1003.437	3.344	10.4
24	1002.924	3.342	10.7
25	1002.411	3.340	11.0
26	991.897	3.305	32.9
27	992.410	3.307	32.9
28	944.710	3.148	126.4
29	808.791	2.695	10.8
30	800.585	2.668	14.4
31	793.917	2.646	9.3
32	748.269	2.494	8.7
33	746.474	2.488	18.2
34	744.422	2.481	25.1
35	742.627	2.475	18.3
36	740.832	2.469	8.4
37	610.555	2.035	4.3
38	603.631	2.012	5.7
39	596.964	1.989	4.5
40	237.677	0.792	93.1
41	231.009	0.770	90.4

Compound 11f
^{13}C NMR

No.	(ppm)	(Hz)	Height
1	19.64	1976.0	0.2262
2	27.65	2783.0	0.1002
3	28.60	2877.6	0.0823
4	29.73	2991.3	0.1096
5	35.27	3549.6	0.0699
6	39.35	3959.7	0.1374
7	39.56	3980.9	0.4236
8	39.76	4001.5	0.8214
9	39.97	4022.8	1.0000
10	40.19	4044.0	0.8454
11	40.39	4064.6	0.4219
12	40.60	4085.8	0.1387
13	44.93	4521.6	0.0693
14	49.39	4969.8	0.0496
15	102.18	10282.5	0.0651
16	115.88	11661.7	0.0835
17	116.54	11727.7	0.0914
18	119.78	12054.2	0.0820
19	130.54	13136.2	0.0753
20	143.99	14490.4	0.0676
21	145.52	14644.5	0.0985
22	148.98	14992.2	0.0573
23	151.44	15240.2	0.0572
24	153.00	15397.1	0.0488
25	153.98	15495.5	0.0762

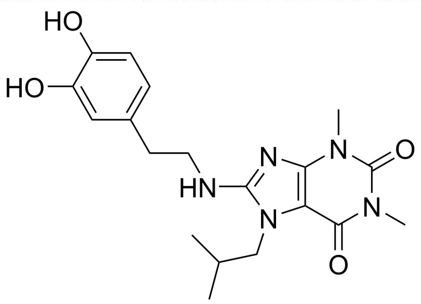

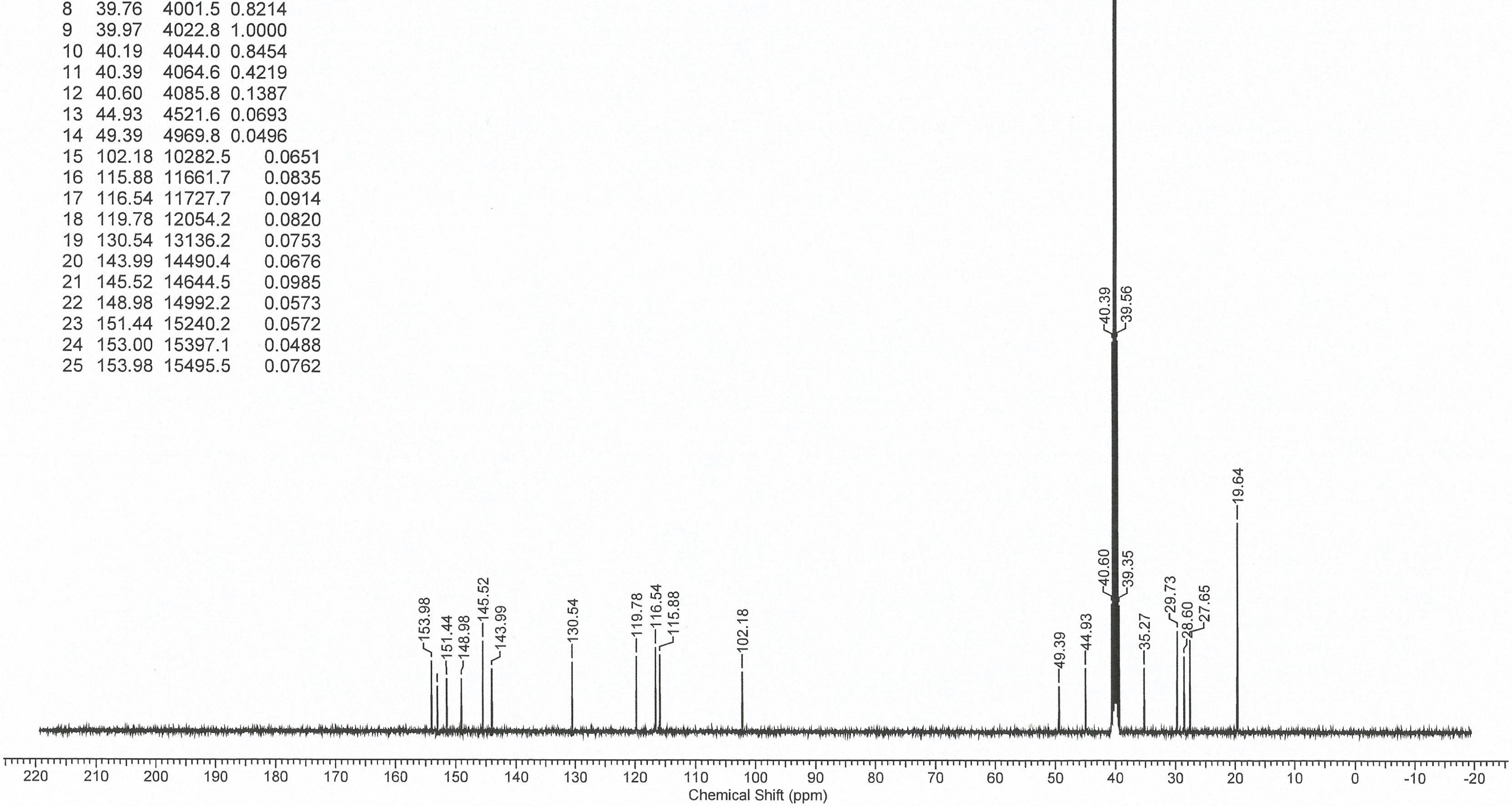

Compound 11g
^{1}H NMR - zoom of the aromatic region

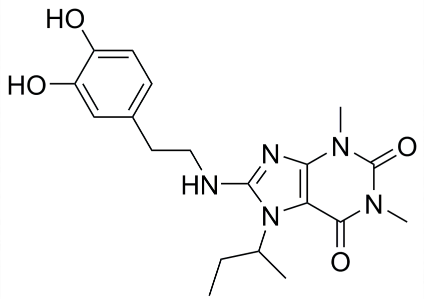

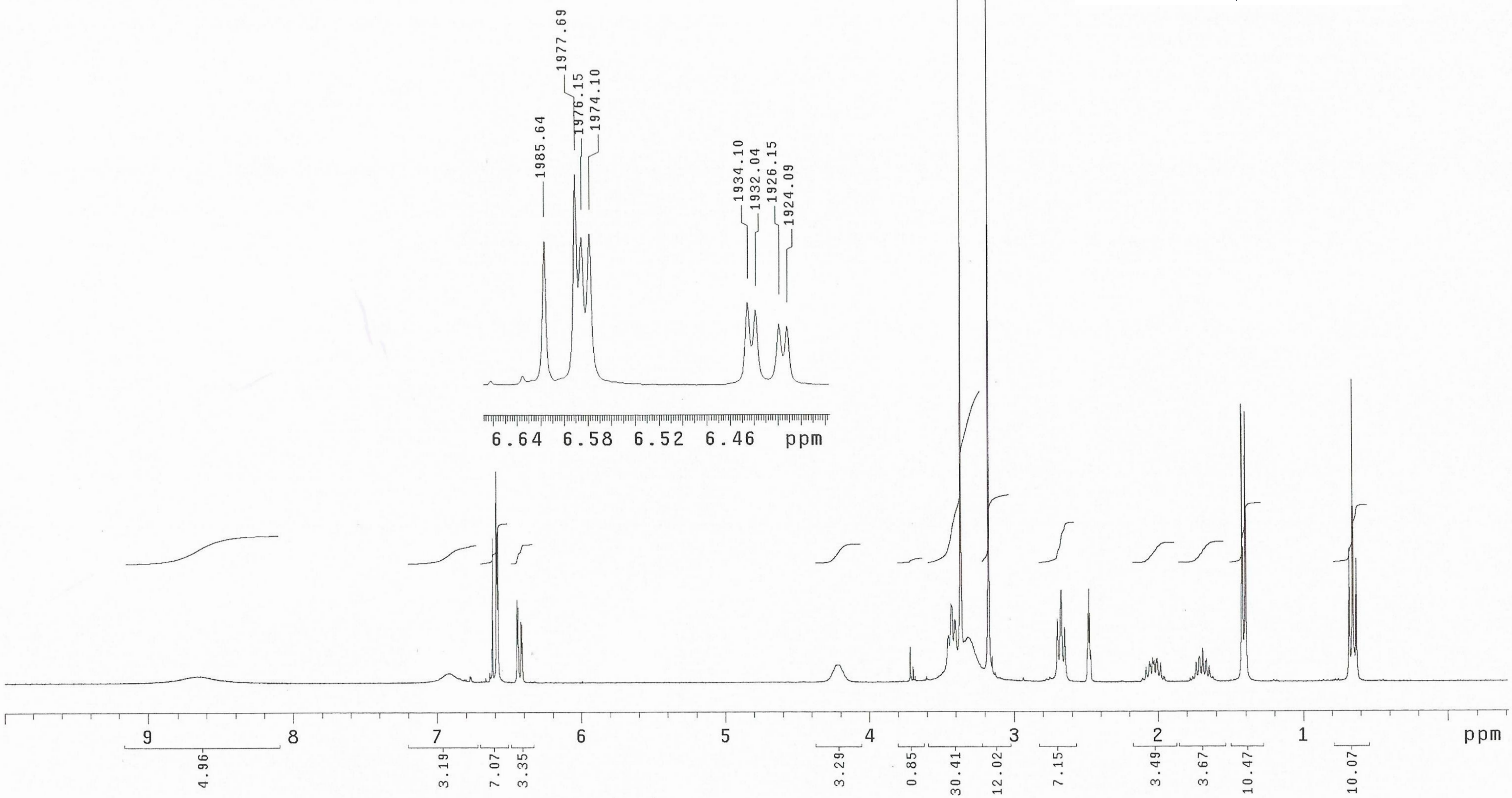

Compound 11g
^{1}H NMR - zoom of the aliphatic region

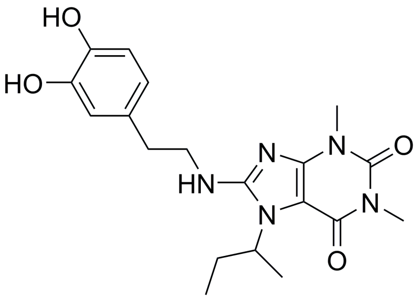

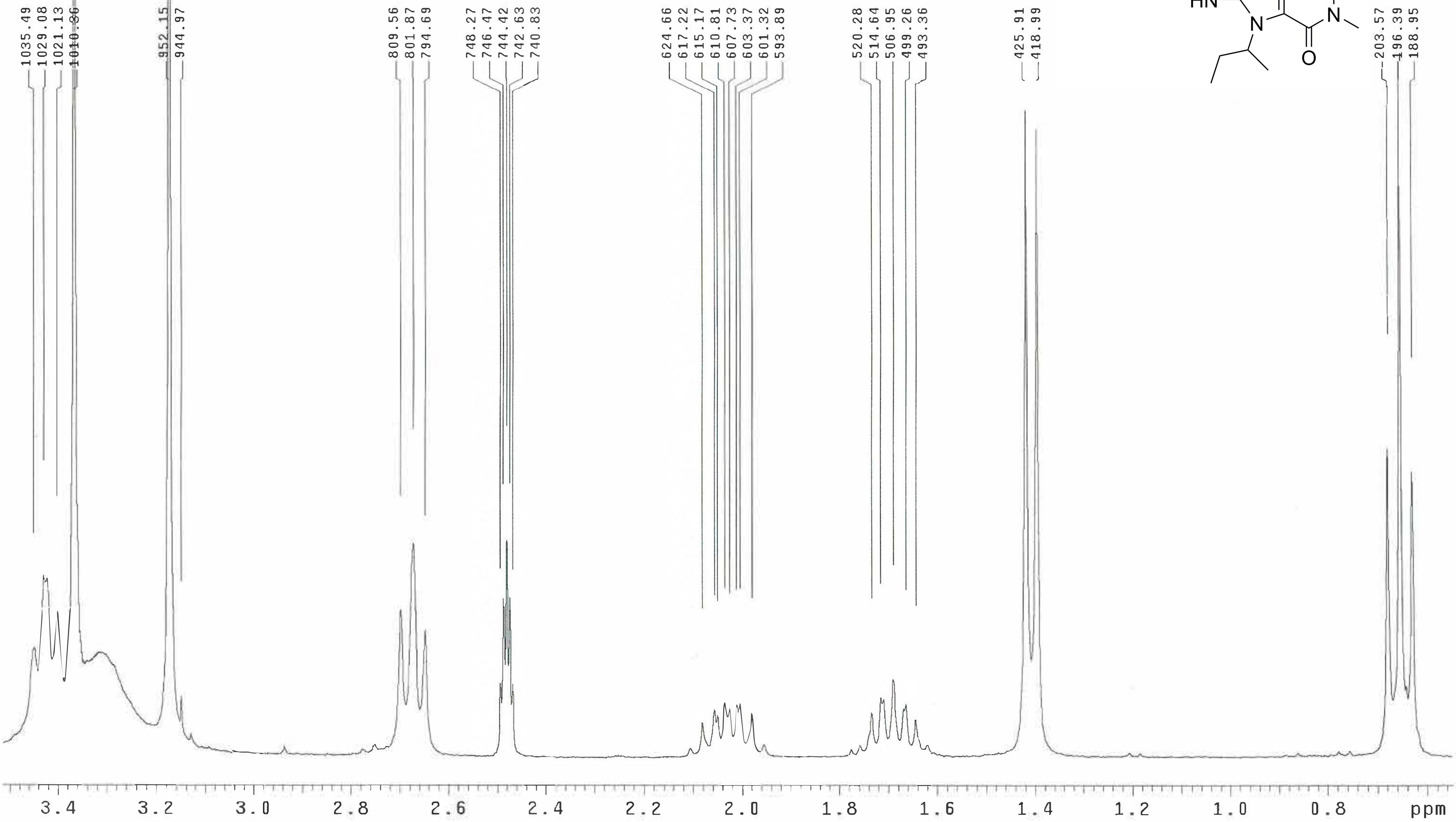

Table of peaks

INDEX	FREQUENCY	PPM	HEIGHT
1	1985.642	6.617	24.2
2	1977.692	6.591	35.4
3	1976.153	6.585	24.9
4	1974.101	6.579	25.5
5	1934.095	6.445	13.9
6	1932.043	6.438	12.6
7	1926.145	6.419	10.2
8	1924.093	6.412	9.8
9	1263.478	4.210	2.9
10	1114.737	3.715	5.9
11	1108.582	3.694	2.5
12	1035.493	3.451	7.8
13	1029.082	3.429	13.1
14	1027.031	3.423	12.8
15	1021.132	3.403	10.5
16	1017.029	3.389	5.6
17	1016.516	3.387	5.7
18	1016.003	3.386	5.7
19	1010.361	3.367	144.0
20	1005.232	3.350	7.2
21	1004.719	3.348	7.0
22	1004.206	3.346	6.8
23	994.718	3.315	7.6
24	952.147	3.173	118.3
25	944.966	3.149	4.3
26	809.561	2.698	10.6
27	801.867	2.672	15.4
28	794.687	2.648	9.1
29	748.269	2.494	5.3
30	746.474	2.488	11.4
31	744.422	2.481	15.6
32	742.627	2.475	11.5
33	740.832	2.469	5.2
34	617.223	2.057	3.4
35	615.171	2.050	3.0
36	610.812	2.035	3.9
37	607.734	2.025	3.5
38	603.375	2.011	3.8
39	601.323	2.004	3.9

INDEX	FREQUENCY	PPM	HEIGHT
40	593.886	1.979	3.2
41	520.285	1.734	3.2
42	514.643	1.715	4.3
43	512.848	1.709	4.1
44	506.949	1.689	5.6
45	499.256	1.664	3.8
46	493.358	1.644	2.7
47	425.911	1.419	46.6
48	418.987	1.396	45.3
49	203.569	0.678	22.3
50	196.388	0.654	50.7
51	192.541	0.642	5.1
52	188.951	0.630	20.6

Compound 11g
^{13}C NMR

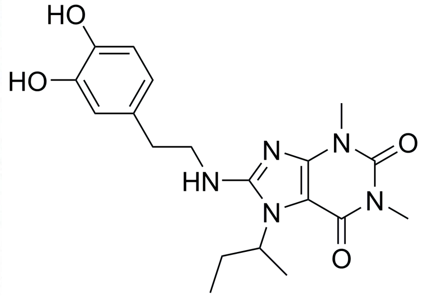

No.	(ppm)	(Hz)	Height
1	11.41	1148.5	0.1684
2	19.92	2004.6	0.0594
3	28.09	2827.0	0.0493
4	28.14	2831.4	0.1303
5	29.78	2996.5	0.1380
6	35.18	3540.0	0.0808
7	39.35	3960.4	0.1342
8	39.57	3981.7	0.4308
9	39.78	4002.9	0.8577
10	39.98	4023.5	1.0000
11	40.19	4044.8	0.8673
12	40.40	4066.0	0.4258
13	40.61	4086.6	0.1393
14	45.11	4539.9	0.0830
15	49.06	4936.8	0.0270
16	52.39	5272.1	0.0795
17	101.77	10241.4	0.0618
18	115.88	11660.9	0.1058
19	116.59	11732.8	0.0989
20	119.83	12058.6	0.0994
21	130.62	13144.3	0.0943
22	143.97	14488.2	0.1083
23	145.51	14643.0	0.1230
24	150.18	15113.2	0.0263
25	151.38	15233.6	0.0762
26	152.67	15363.4	0.0654
27	153.95	15492.5	0.0604

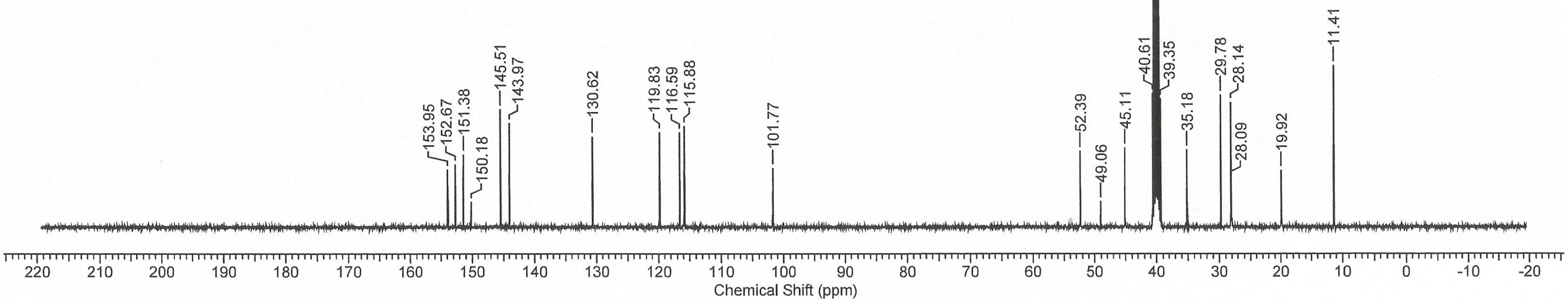

Compound 11h
^{1}H NMR

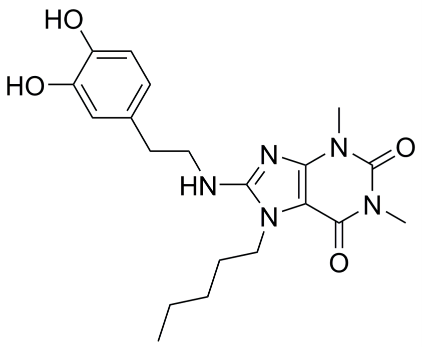

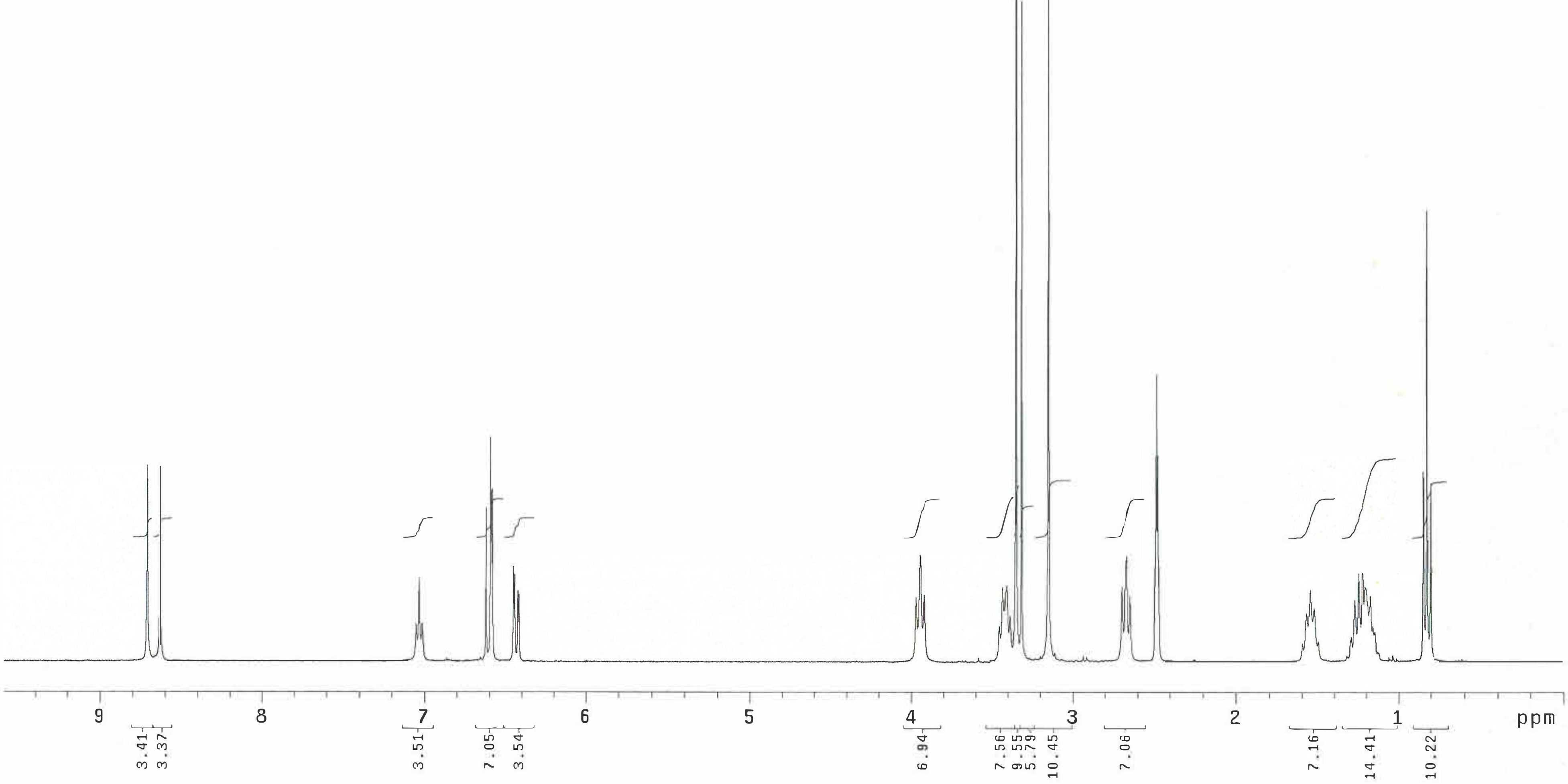

Compound 11h
^{1}H NMR - zoom of the aliphatic region

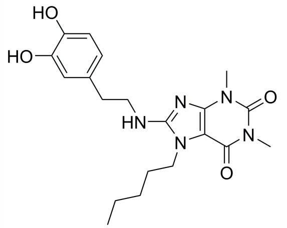

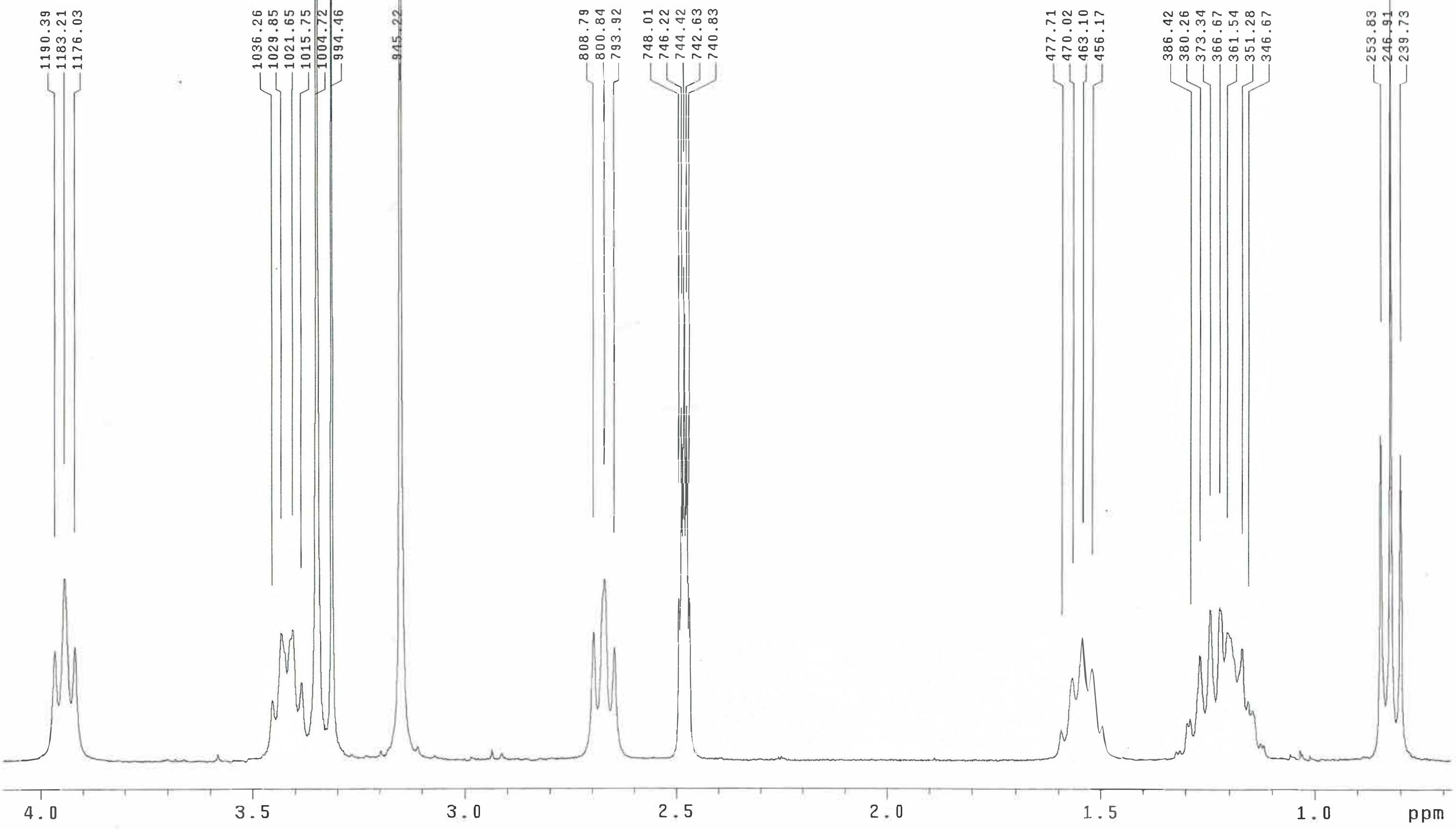

Compound 11h
^{1}H NMR - zoom of the aromatic region

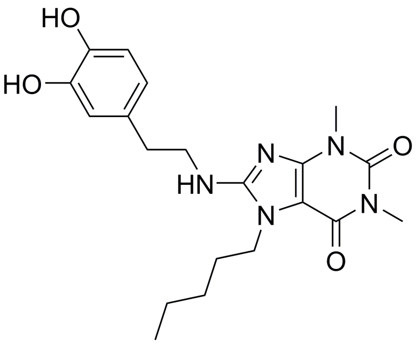

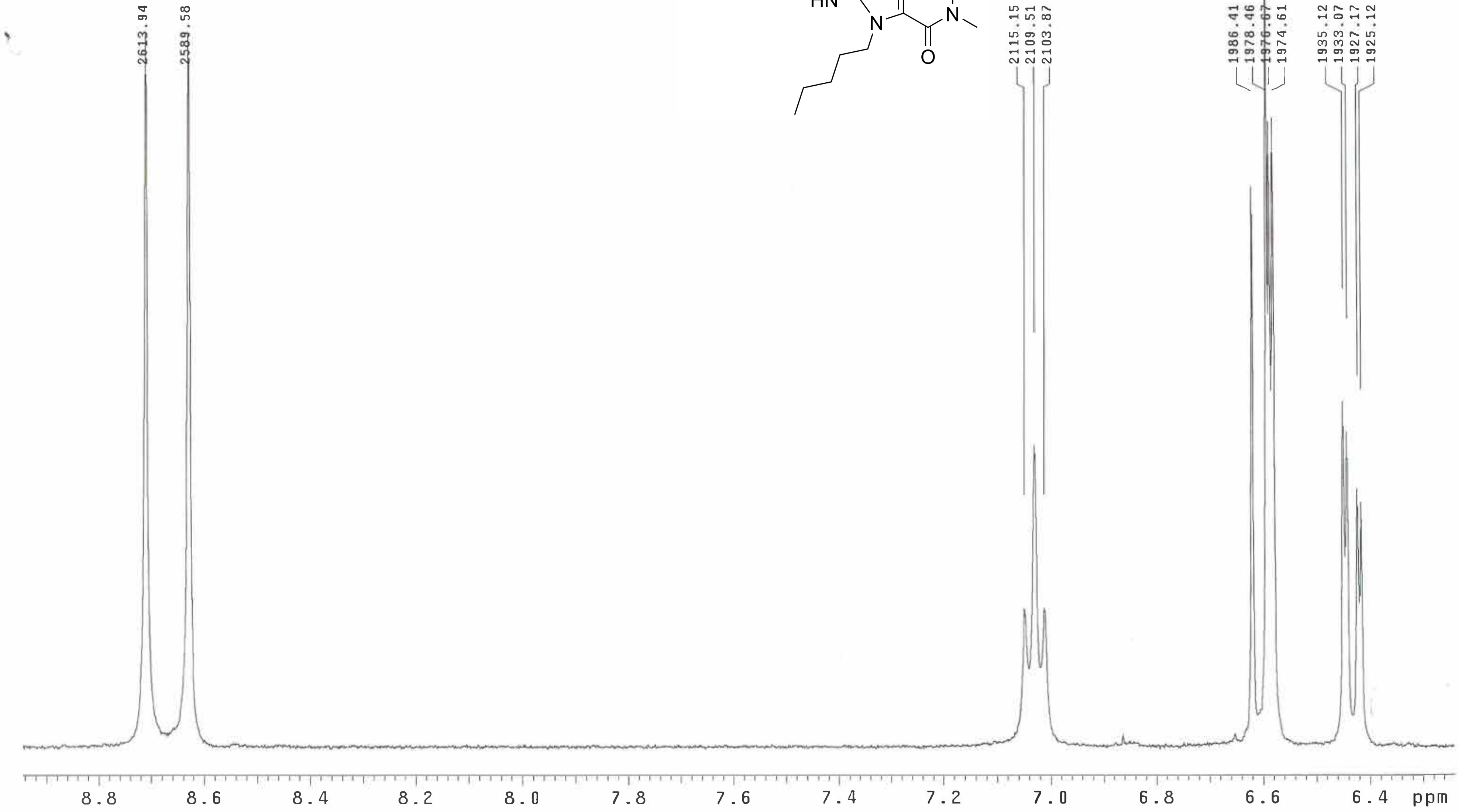

Table of peaks

INDEX	FREQUENCY	PPM	HEIGHT
1	2613.945	8.711	31.7
2	2589.582	8.630	31.5
3	2115.149	7.049	6.1
4	2109.507	7.030	13.4
5	2103.865	7.011	6.1
6	1986.411	6.620	24.8
7	1978.461	6.593	36.3
8	1976.666	6.587	27.7
9	1974.614	6.580	27.8
10	1935.121	6.449	15.3
11	1933.069	6.442	14.0
12	1927.171	6.422	11.4
13	1925.119	6.415	10.8
14	1190.389	3.967	10.4
15	1183.209	3.943	17.2
16	1176.028	3.919	10.8
17	1036.263	3.453	5.7
18	1029.852	3.432	12.0
19	1021.645	3.405	12.3
20	1015.747	3.385	7.3
21	1004.719	3.348	144.0
22	994.461	3.314	106.8
23	945.223	3.150	133.3
24	808.791	2.695	12.0
25	800.841	2.669	17.0
26	793.917	2.646	10.6
27	748.013	2.493	15.2
28	746.218	2.487	33.2
29	744.422	2.481	46.5
30	742.627	2.475	33.4
31	740.832	2.469	15.2
32	477.714	1.592	2.9
33	470.021	1.566	7.8
34	463.096	1.543	11.6
35	456.172	1.520	8.7
36	448.735	1.495	3.3
37	388.726	1.295	3.6
38	386.418	1.288	3.9
39	380.263	1.267	9.9

INDEX	FREQUENCY	PPM	HEIGHT
40	373.339	1.244	14.2
41	366.671	1.222	14.5
42	361.542	1.205	12.1
43	351.284	1.171	10.6
44	346.668	1.155	5.7
45	343.591	1.145	4.7
46	253.833	0.846	30.7
47	246.909	0.823	73.1
48	239.728	0.799	28.9

Compound 11h
^{13}C NMR

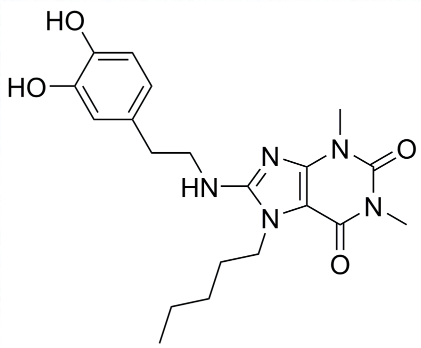

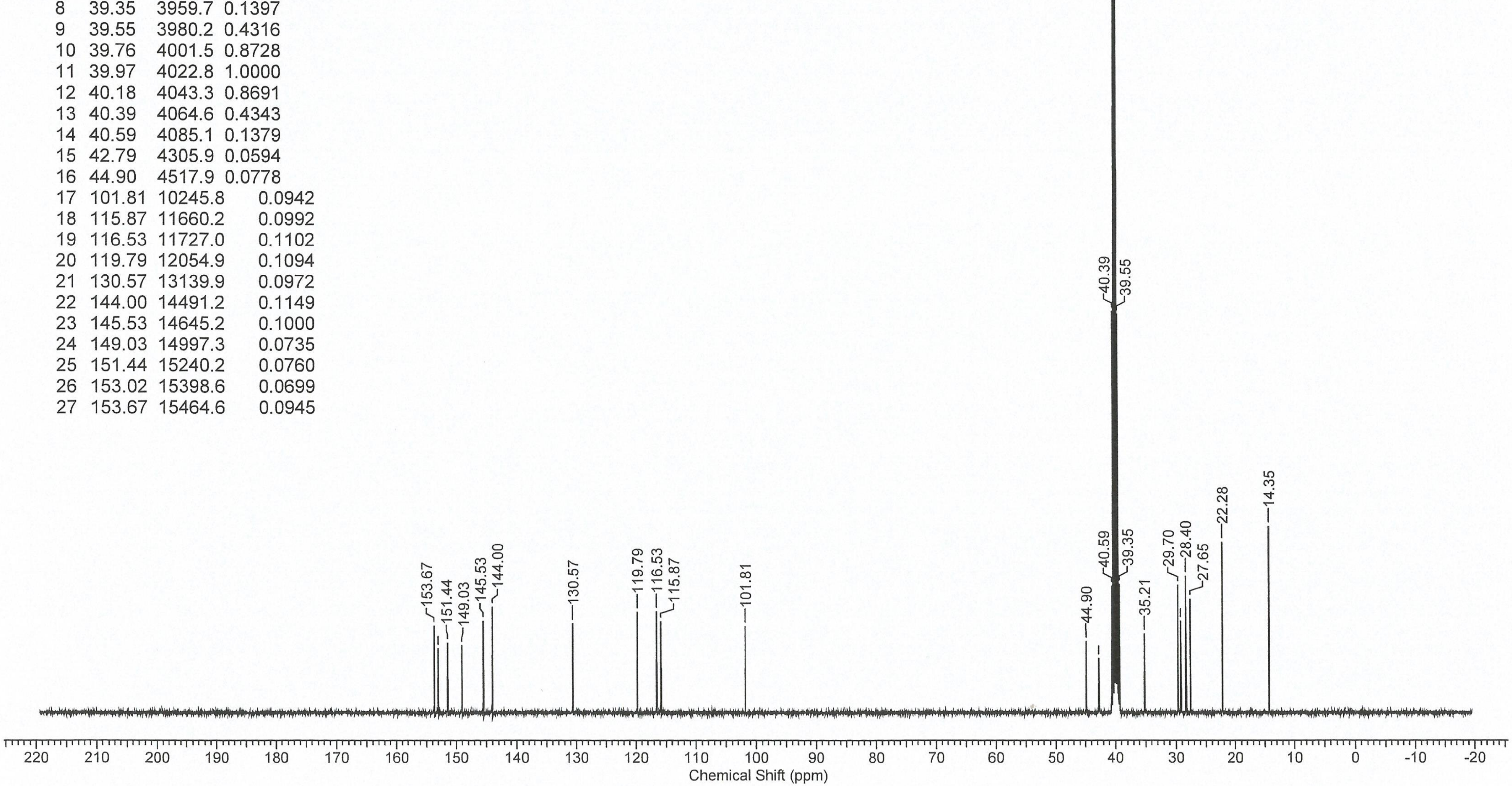

No.	(ppm)	(Hz)	Height
1	14.35	1444.2	0.2021
2	22.28	2242.3	0.1853
3	27.65	2782.2	0.1236
4	28.40	2857.8	0.1484
5	29.22	2940.7	0.0998
6	29.70	2989.1	0.1384
7	35.21	3543.7	0.0858
8	39.35	3959.7	0.1397
9	39.55	3980.2	0.4316
10	39.76	4001.5	0.8728
11	39.97	4022.8	1.0000
12	40.18	4043.3	0.8691
13	40.39	4064.6	0.4343
14	40.59	4085.1	0.1379
15	42.79	4305.9	0.0594
16	44.90	4517.9	0.0778
17	101.81	10245.8	0.0942
18	115.87	11660.2	0.0992
19	116.53	11727.0	0.1102
20	119.79	12054.9	0.1094
21	130.57	13139.9	0.0972
22	144.00	14491.2	0.1149
23	145.53	14645.2	0.1000
24	149.03	14997.3	0.0735
25	151.44	15240.2	0.0760
26	153.02	15398.6	0.0699
27	153.67	15464.6	0.0945

Compound 11i
^{1}H NMR

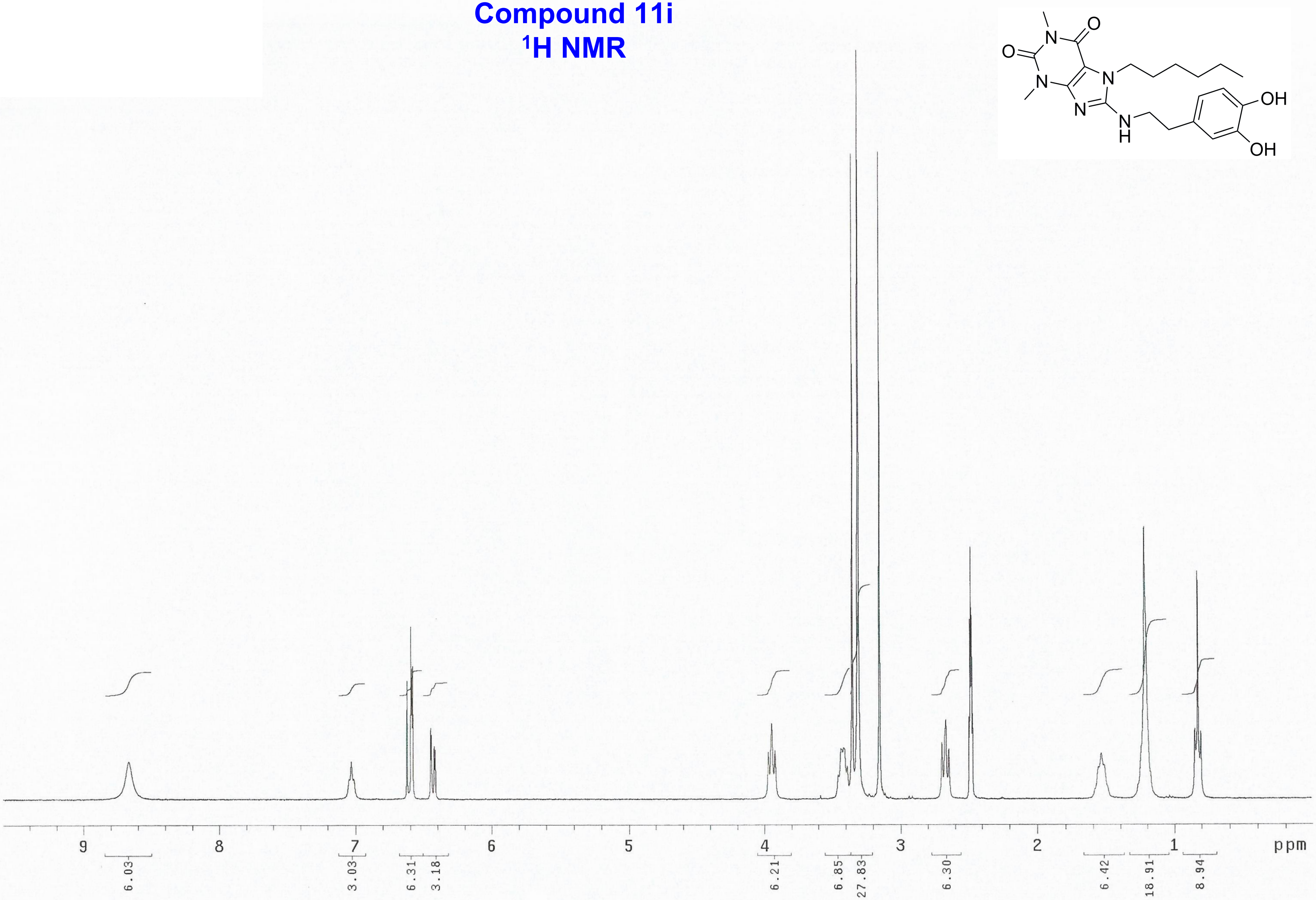

Compound 11i
^{1}H NMR - zoom of the aliphatic region

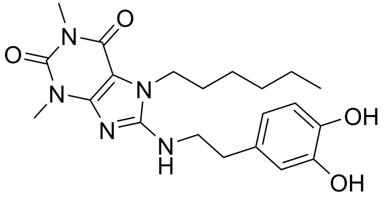

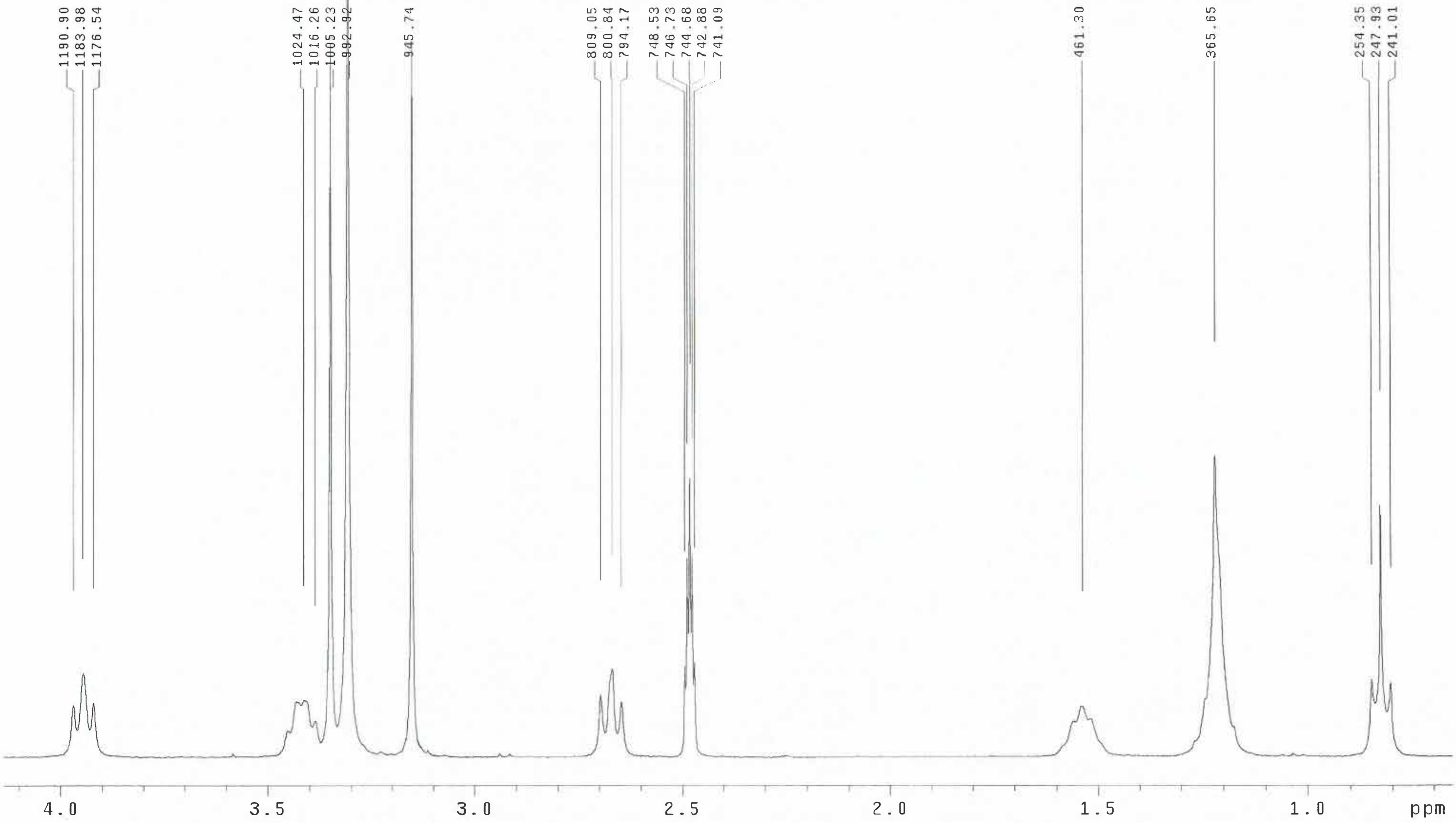

Compound 11i
^{1}H NMR - zoom of the aromatic region

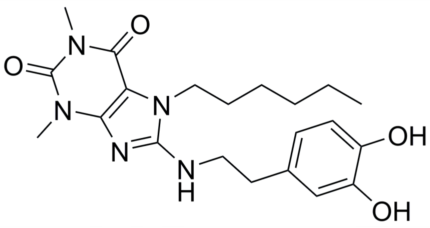

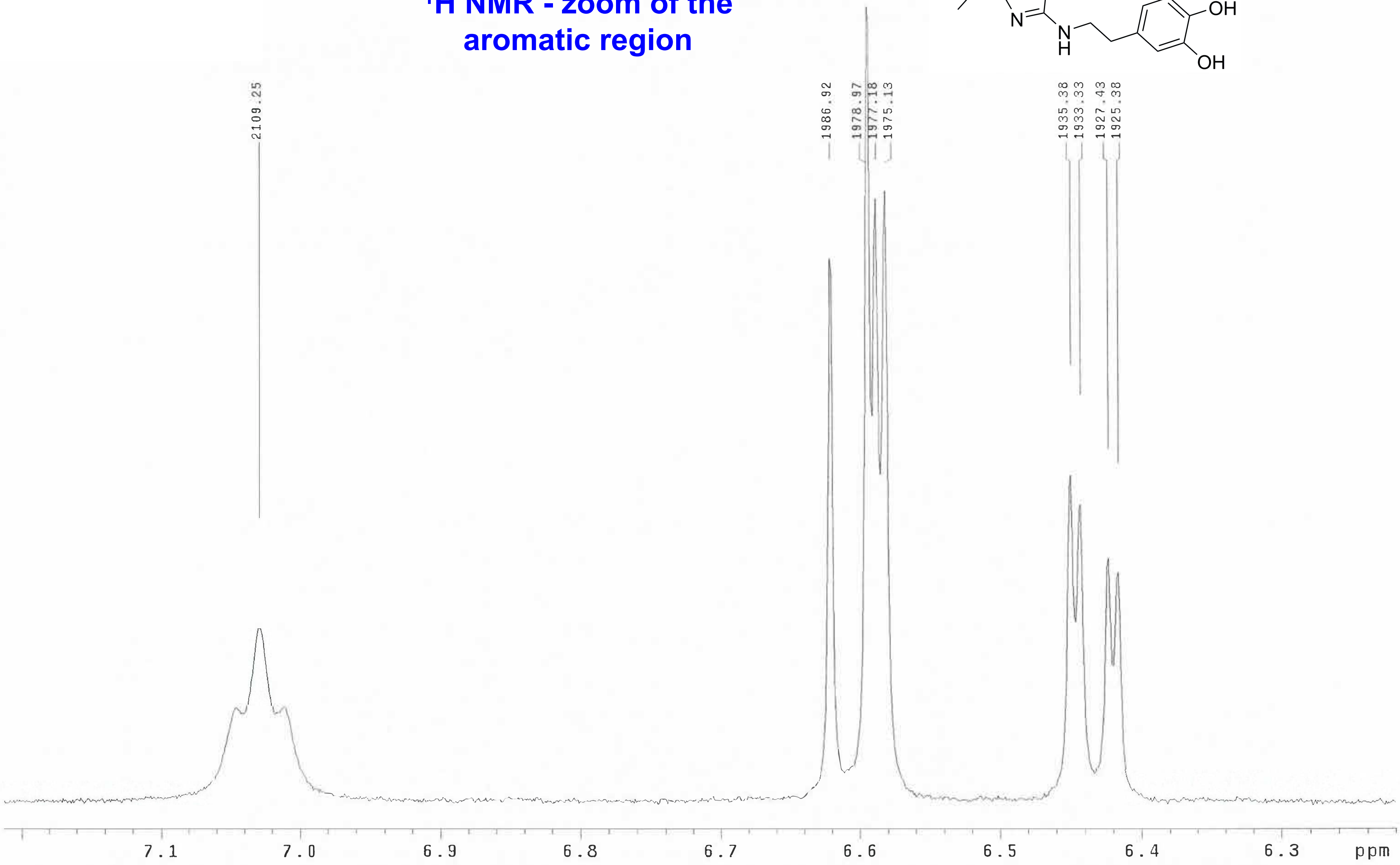

Table of peaks

INDEX	FREQUENCY	PPM	HEIGHT
1	2600.353	8.666	7.4
2	2109.251	7.029	7.3
3	2104.122	7.012	4.0
4	1986.924	6.621	22.8
5	1978.974	6.595	33.3
6	1977.179	6.589	25.3
7	1975.127	6.582	25.6
8	1935.377	6.450	13.7
9	1933.326	6.443	12.5
10	1927.427	6.423	10.2
11	1925.376	6.416	9.6
12	1190.902	3.969	9.1
13	1183.978	3.946	14.6
14	1176.541	3.921	9.4
15	1036.006	3.452	4.6
16	1029.595	3.431	9.7
17	1021.902	3.405	9.6
18	1016.260	3.387	6.3
19	1005.232	3.350	124.0
20	992.923	3.309	144.0
21	945.736	3.152	124.4
22	809.048	2.696	10.8
23	800.841	2.669	15.2
24	794.174	2.647	9.7
25	748.526	2.494	15.7
26	746.730	2.488	34.6
27	744.679	2.482	48.5
28	742.884	2.476	35.3
29	741.089	2.470	16.4
30	461.301	1.537	8.8
31	365.645	1.218	52.4
32	254.346	0.848	13.5
33	247.935	0.826	43.8
34	241.011	0.803	13.0

Compound 11i
^{13}C NMR

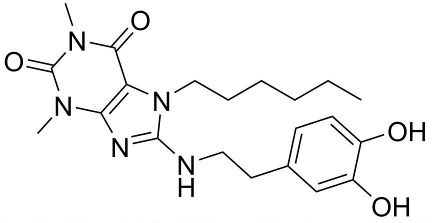

No.	(ppm)	(Hz)	Height
1	14.34	1442.7	0.1584
2	22.49	2263.6	0.1434
3	25.89	2605.4	0.1005
4	27.65	2783.0	0.0934
5	29.49	2967.8	0.0731
6	29.71	2989.8	0.1046
7	31.32	3152.0	0.1243
8	35.23	3545.2	0.0659
9	39.34	3958.9	0.1306
10	39.54	3979.5	0.4177
11	39.76	4000.7	0.8501
12	39.97	4022.0	1.0000
13	40.17	4042.6	0.8434
14	40.38	4063.8	0.4184
15	40.59	4084.4	0.1324
16	42.82	4308.9	0.0473
17	44.90	4517.9	0.0624
18	101.81	10245.1	0.0601
19	115.87	11660.2	0.0833
20	116.52	11725.5	0.0873
21	119.78	12053.4	0.0763
22	130.56	13138.4	0.0608
23	144.00	14491.2	0.0697
24	145.53	14645.2	0.0952
25	149.03	14997.3	0.0556
26	151.44	15240.2	0.0507
27	153.02	15398.6	0.0607
28	153.67	15464.6	0.0712

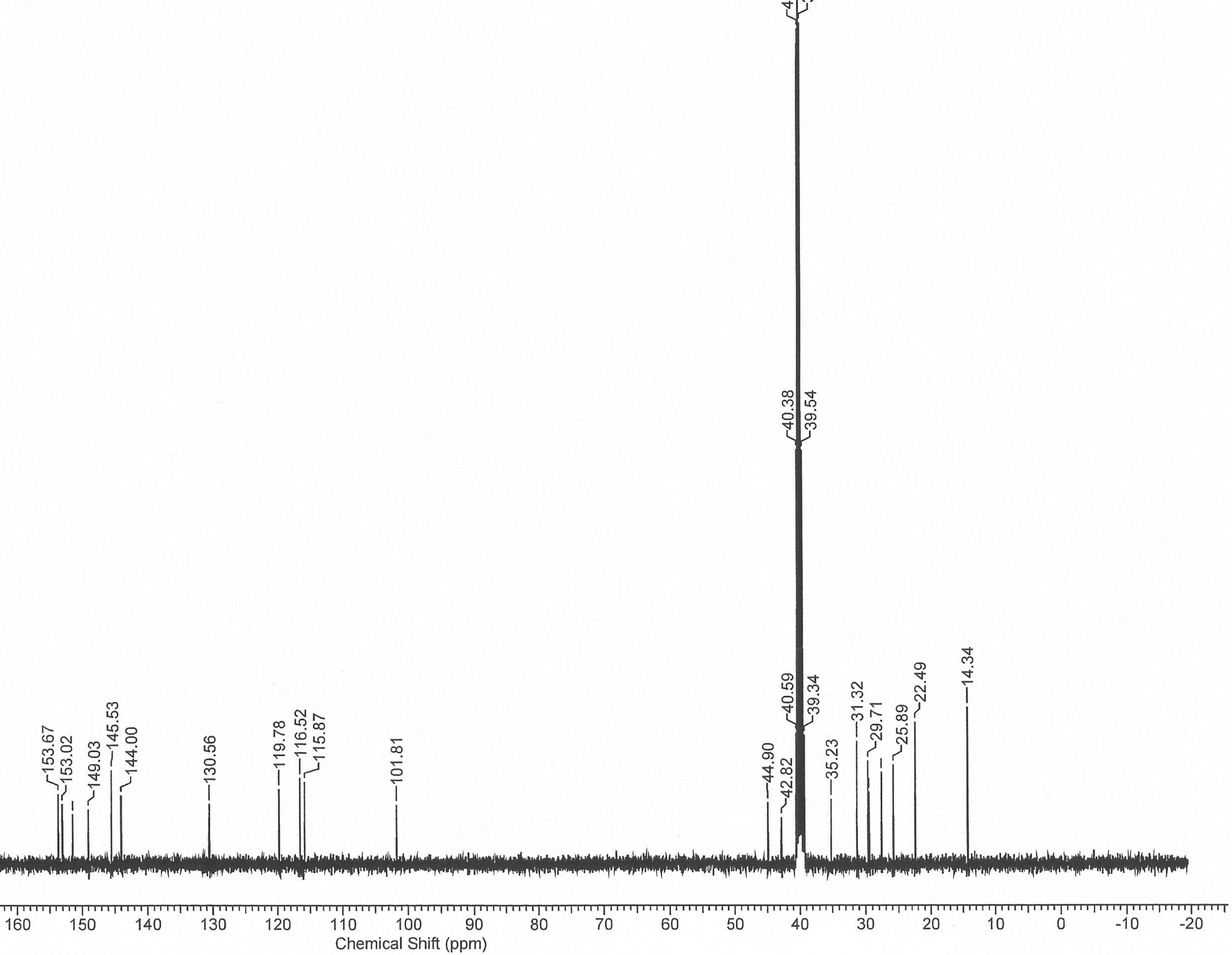

Compound 11j
^{1}H NMR

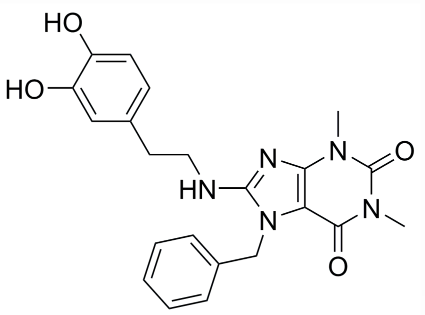

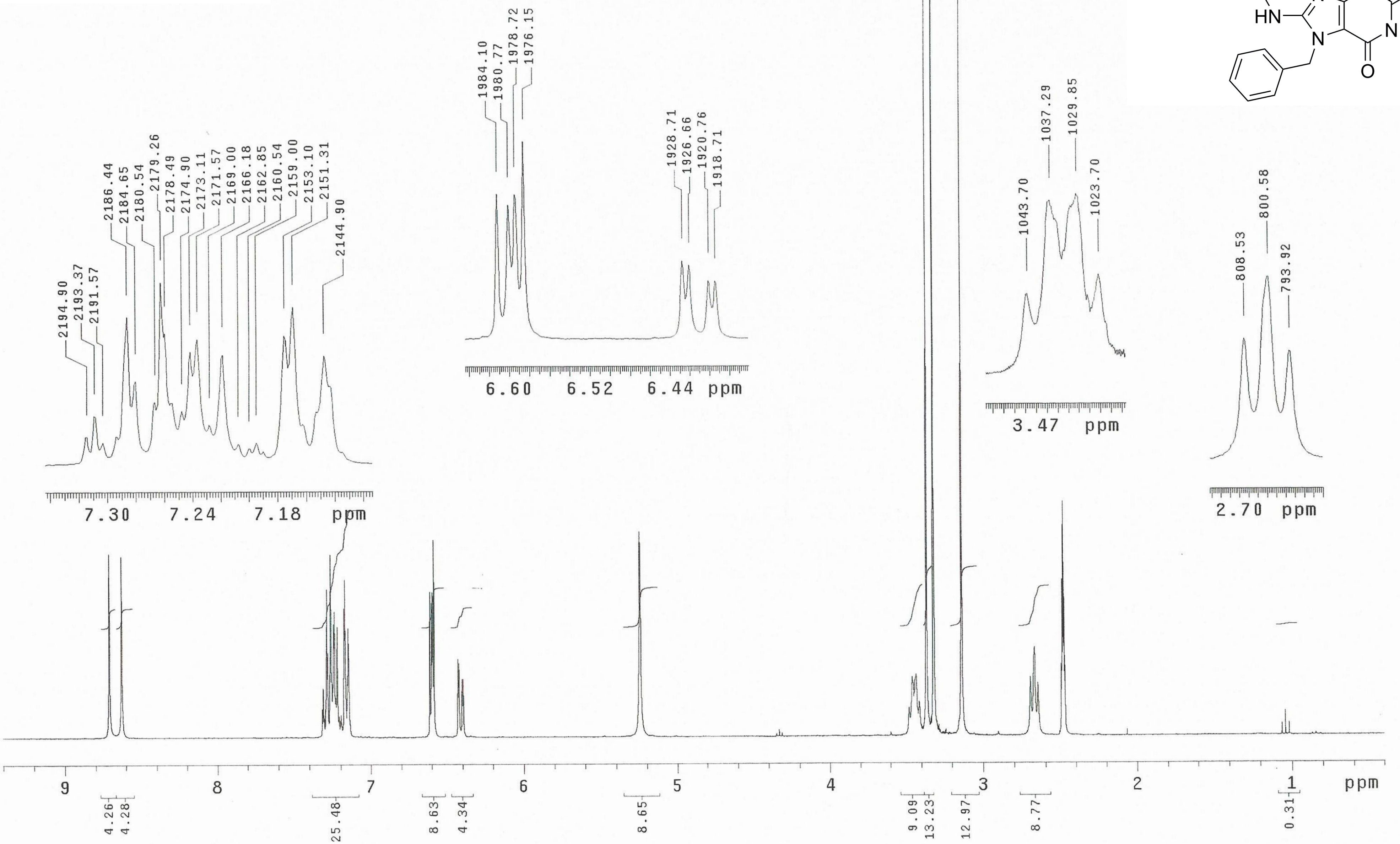

Table of peaks

INDEX	FREQUENCY	PPM	HEIGHT
1	2613.688	8.710	33.6
2	2589.582	8.630	33.1
3	2194.905	7.314	5.1
4	2193.366	7.309	9.0
5	2191.571	7.303	4.1
6	2188.494	7.293	5.1
7	2186.442	7.286	27.1
8	2184.647	7.280	15.4
9	2180.544	7.267	11.5
10	2179.261	7.262	33.4
11	2178.492	7.260	24.1
12	2176.953	7.255	11.4
13	2174.902	7.248	9.8
14	2173.107	7.242	20.7
15	2171.568	7.237	23.0
16	2169.003	7.228	7.3
17	2166.183	7.219	20.2
18	2162.849	7.208	3.8
19	2160.541	7.200	3.0
20	2159.002	7.195	4.0
21	2153.104	7.175	23.6
22	2151.308	7.169	28.8
23	2149.257	7.162	7.3
24	2146.436	7.153	9.7
25	2144.897	7.148	19.9
26	1984.103	6.612	26.5
27	1980.769	6.601	24.5
28	1978.717	6.594	26.4
29	1976.153	6.585	35.9
30	1928.710	6.427	14.2
31	1926.658	6.420	13.5
32	1920.760	6.401	10.6
33	1918.708	6.394	10.6
34	1574.039	5.245	37.5
35	1043.700	3.478	5.1
36	1037.289	3.457	10.8
37	1029.852	3.432	11.2
38	~~1026.774~~	~~3.422~~	~~5.0~~
39	1023.697	3.411	6.3
40	1014.208	3.380	2.8
41	1013.695	3.378	3.1
42	1009.592	3.364	152.9
43	1004.719	3.348	3.4
44	1001.898	3.339	2.5
45	1000.873	3.335	3.1
46	1000.360	3.334	3.4
47	999.847	3.332	3.4
48	999.334	3.330	3.8
49	998.821	3.329	5.4
50	998.308	3.327	7.6
51	996.257	3.320	438.3
52	990.102	3.299	2.8
53	989.589	3.298	2.7
54	989.076	3.296	2.6
55	946.762	3.155	2.7
56	941.633	3.138	143.0
57	808.535	2.694	10.8
58	800.585	2.668	16.2
59	793.917	2.646	9.7
60	748.782	2.495	13.1
61	746.730	2.488	28.6
62	744.935	2.482	42.7
63	743.140	2.476	31.6
64	741.345	2.470	14.5
65	311.534	1.038	4.4

Compound 11j
^{13}C NMR

No.	(ppm)	(Hz)	Height
1	27.65	2783.0	0.0902
2	29.76	2995.0	0.1076
3	35.09	3531.2	0.0666
4	39.35	3960.4	0.1371
5	39.56	3980.9	0.4337
6	39.77	4002.2	0.8719
7	39.97	4022.8	1.0000
8	40.19	4044.0	0.8765
9	40.40	4065.3	0.4349
10	40.60	4085.8	0.1423
11	44.90	4518.7	0.0612
12	45.71	4600.1	0.0488
13	101.68	10231.9	0.0656
14	115.91	11664.6	0.0826
15	116.53	11727.0	0.0887
16	119.83	12058.6	0.0848
17	127.54	12834.7	0.2572
18	127.74	12854.5	0.0691
19	128.92	12973.4	0.2653
20	130.46	13128.9	0.0748
21	137.53	13840.5	0.0809
22	144.01	14491.9	0.0738
23	145.53	14645.2	0.1022
24	149.30	15024.5	0.0607
25	151.44	15239.4	0.0564
26	153.19	15416.2	0.0592
27	154.13	15510.1	0.0825

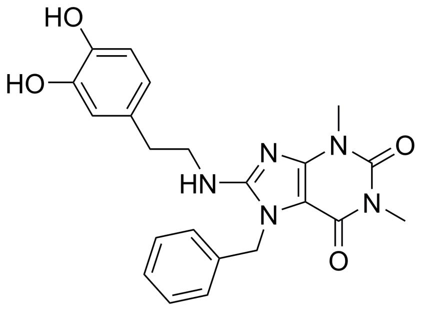

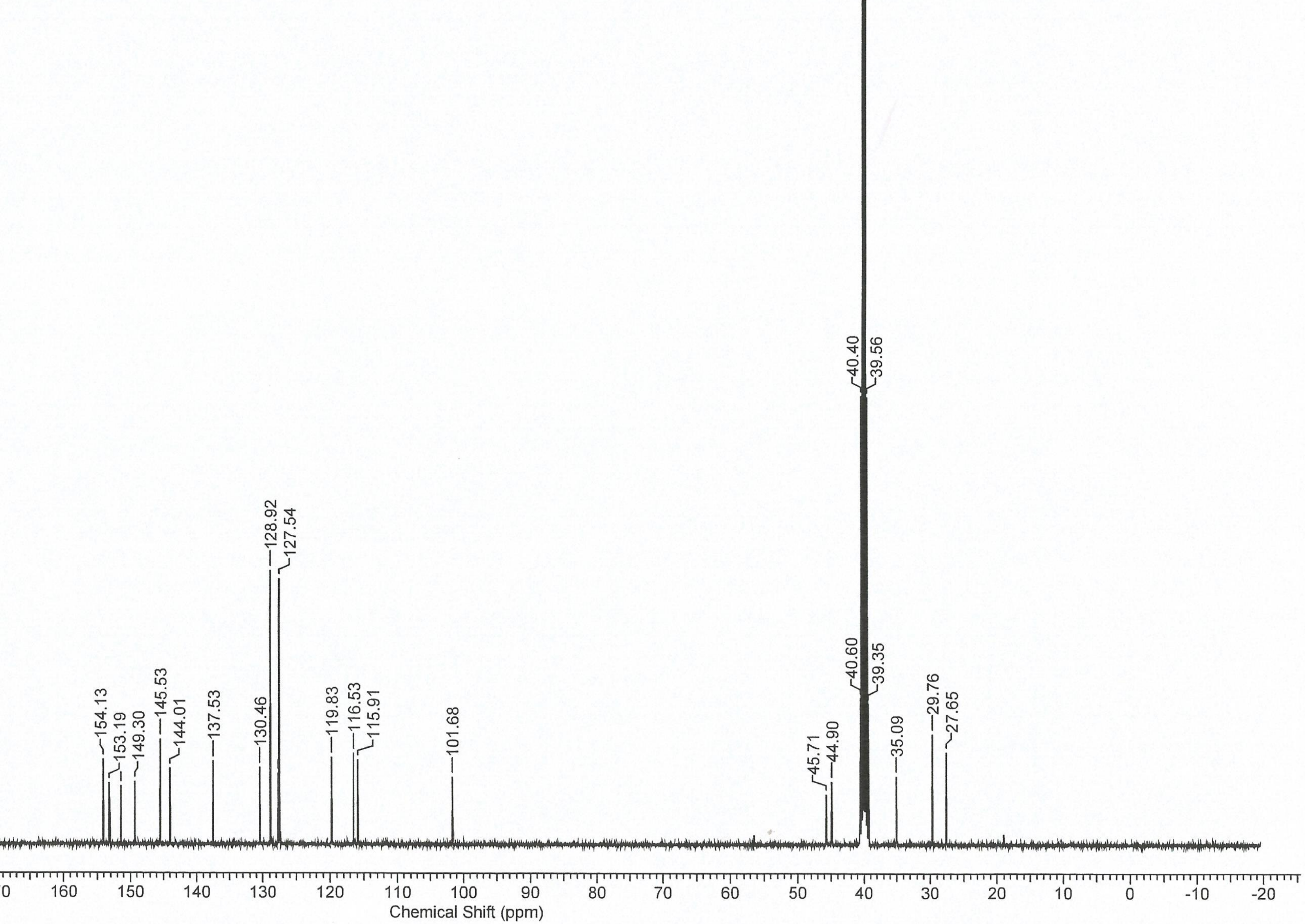

Compound 11k
^{1}H NMR

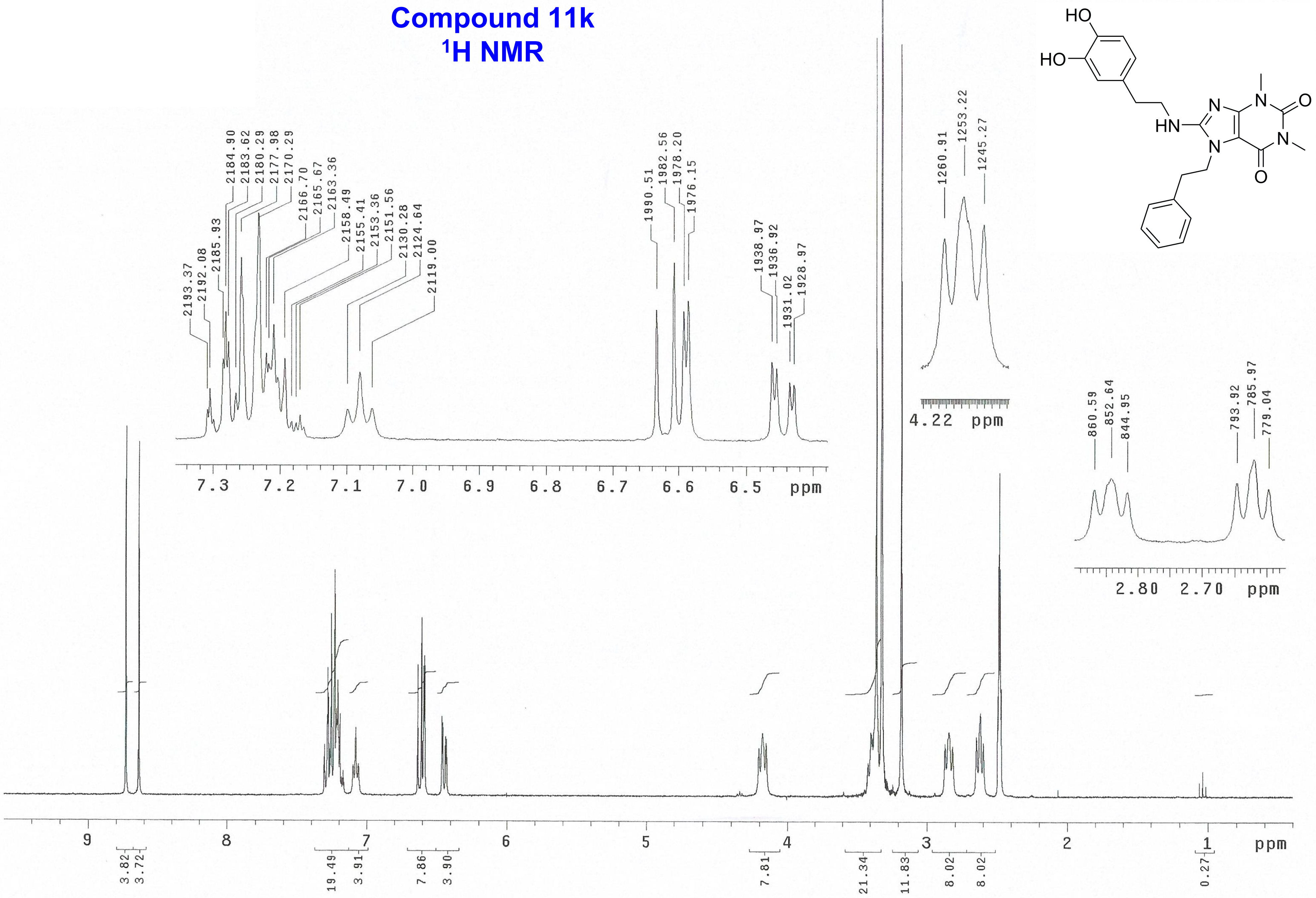

Table of peaks

INDEX	FREQUENCY	PPM	HEIGHT	INDEX	FREQUENCY	PPM	HEIGHT	INDEX	FREQUENCY	PPM	HEIGHT
1	2619.843	8.730	72.3	40	1016.516	3.387	11.0	79	742.884	2.476	44.6
2	2592.146	8.638	69.3	41	1016.003	3.386	11.1	80	741.089	2.470	21.1
3	2193.366	7.309	5.9	42	1015.490	3.384	11.4	81	317.689	1.059	2.7
4	2192.084	7.305	10.0	43	1014.977	3.382	10.6	82	310.765	1.036	4.9
5	2190.545	7.300	3.9	44	1014.464	3.381	10.1				
6	2185.929	7.284	15.8	45	1007.284	3.357	148.5				
7	2184.903	7.281	25.0	46	1001.129	3.336	6.9				
8	2183.621	7.277	19.2	47	1000.616	3.334	7.6				
9	2180.287	7.266	9.1	48	1000.103	3.333	8.3				
10	2177.979	7.258	35.5	49	999.590	3.331	9.6				
11	2170.286	7.232	44.2	50	999.077	3.329	12.0				
12	2166.695	7.220	16.9	51	998.565	3.328	15.8				
13	2165.670	7.217	14.9	52	998.052	3.326	21.4				
14	2163.362	7.209	22.5	53	996.000	3.319	1106.3				
15	2161.566	7.203	12.2	54	993.179	3.310	22.5				
16	2158.489	7.193	15.9	55	992.666	3.308	16.8				
17	2155.412	7.183	3.6	56	989.845	3.299	6.5				
18	2153.360	7.176	3.3	57	989.332	3.297	5.1				
19	2151.565	7.170	4.8	58	988.819	3.295	4.6				
20	2130.279	7.099	5.9	59	988.307	3.293	3.8				
21	2124.638	7.080	13.3	60	987.794	3.292	3.3				
22	2118.996	7.061	6.2	61	987.281	3.290	3.3				
23	1990.514	6.633	25.6	62	986.768	3.288	3.4				
24	1982.564	6.607	34.8	63	986.255	3.287	3.5				
25	1978.204	6.592	25.3	64	985.742	3.285	3.2				
26	1976.153	6.585	27.4	65	984.716	3.282	2.9				
27	1938.968	6.461	15.5	66	984.203	3.280	3.1				
28	1936.916	6.455	14.2	67	983.690	3.278	2.7				
29	1931.018	6.435	11.6	68	953.942	3.179	147.3				
30	1928.966	6.428	11.0	69	947.787	3.158	2.7				
31	1260.913	4.202	9.4	70	860.594	2.868	10.3				
32	1253.220	4.176	12.4	71	852.644	2.841	12.6				
33	1245.270	4.150	10.4	72	844.951	2.816	9.8				
34	1027.031	3.423	2.7	73	793.917	2.646	11.7				
35	1024.466	3.414	6.6	74	785.967	2.619	16.4				
36	1018.568	3.394	12.5	75	779.043	2.596	10.5				
37	1018.055	3.393	12.3	76	748.526	2.494	19.6				
38	1017.542	3.391	12.1	77	746.730	2.488	45.1				
39	1017.029	3.389	11.7	78	744.935	2.482	63.5				

Compound 11k
^{13}C NMR

No.	(ppm)	(Hz)	Height
1	27.70	2787.4	0.0801
2	29.75	2994.2	0.0755
3	35.18	3540.0	0.0597
4	35.62	3584.8	0.0504
5	39.35	3960.4	0.1400
6	39.56	3980.9	0.4183
7	39.77	4002.2	0.8594
8	39.98	4023.5	1.0000
9	40.19	4044.0	0.8470
10	40.40	4065.3	0.4294
11	40.61	4086.6	0.1364
12	44.14	4441.6	0.0411
13	44.86	4514.3	0.0560
14	101.62	10226.0	0.0499
15	115.93	11666.1	0.0717
16	116.54	11727.7	0.0794
17	119.78	12054.2	0.0722
18	126.83	12763.5	0.0646
19	128.72	12953.5	0.1909
20	129.35	13016.6	0.1823
21	130.55	13137.7	0.0654
22	138.42	13930.0	0.0642
23	144.02	14493.4	0.0774
24	145.55	14646.7	0.0712
25	149.20	15014.2	0.0435
26	151.48	15243.8	0.0500
27	153.15	15411.8	0.0547
28	153.67	15463.9	0.0673

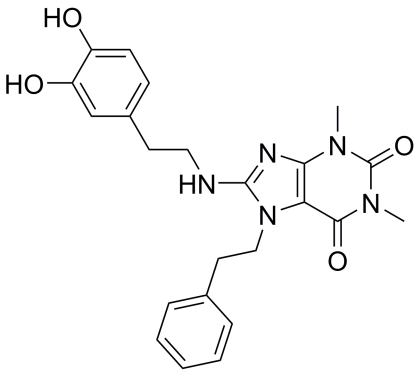

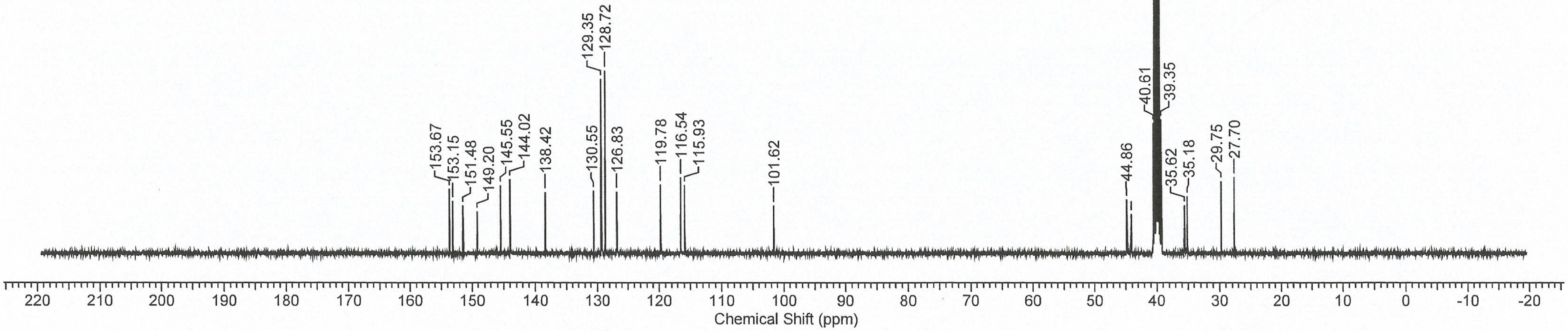

Compound 11l
^{1}H NMR

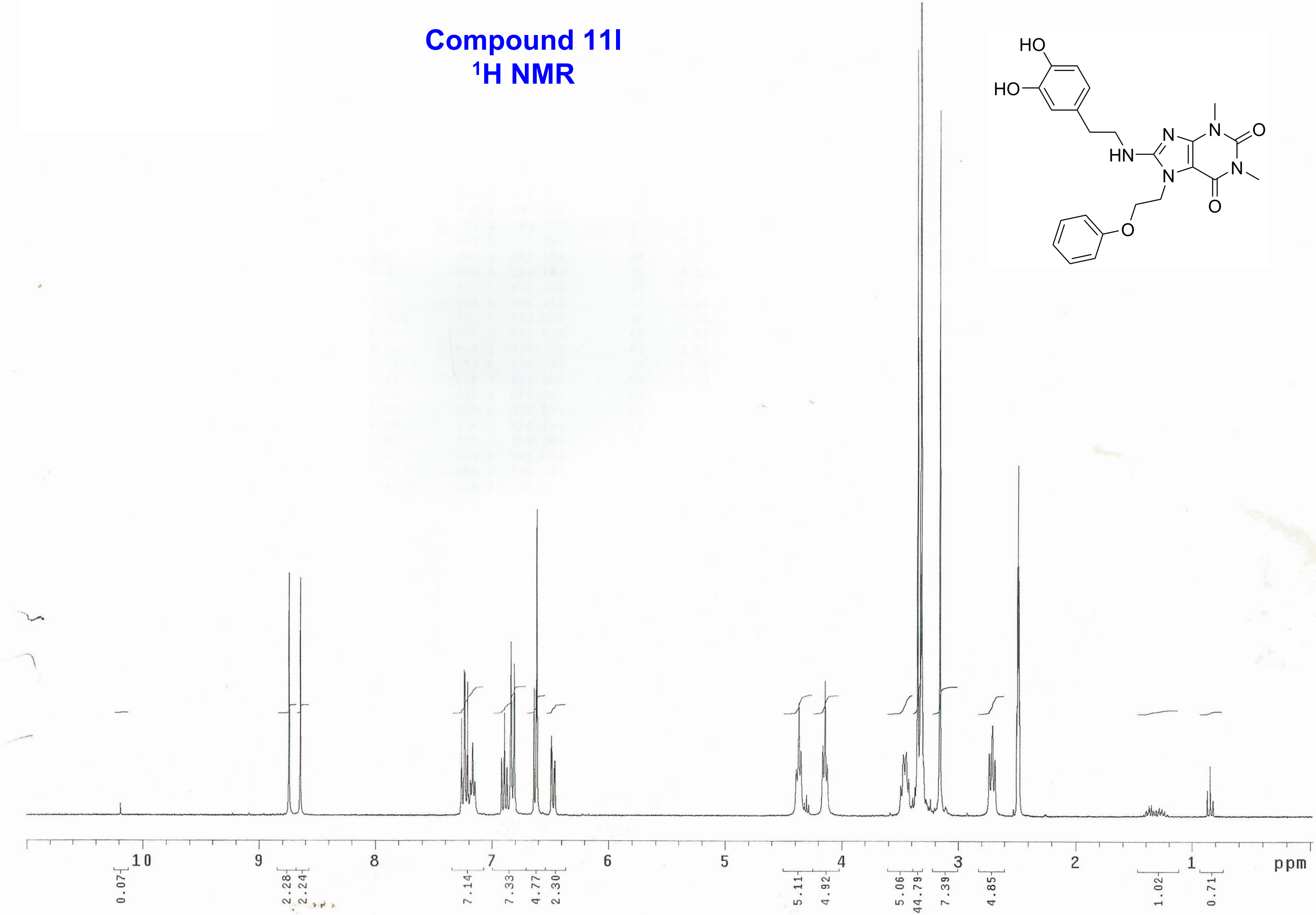

Compound 11l
^{1}H NMR - zoom of the aliphatic region

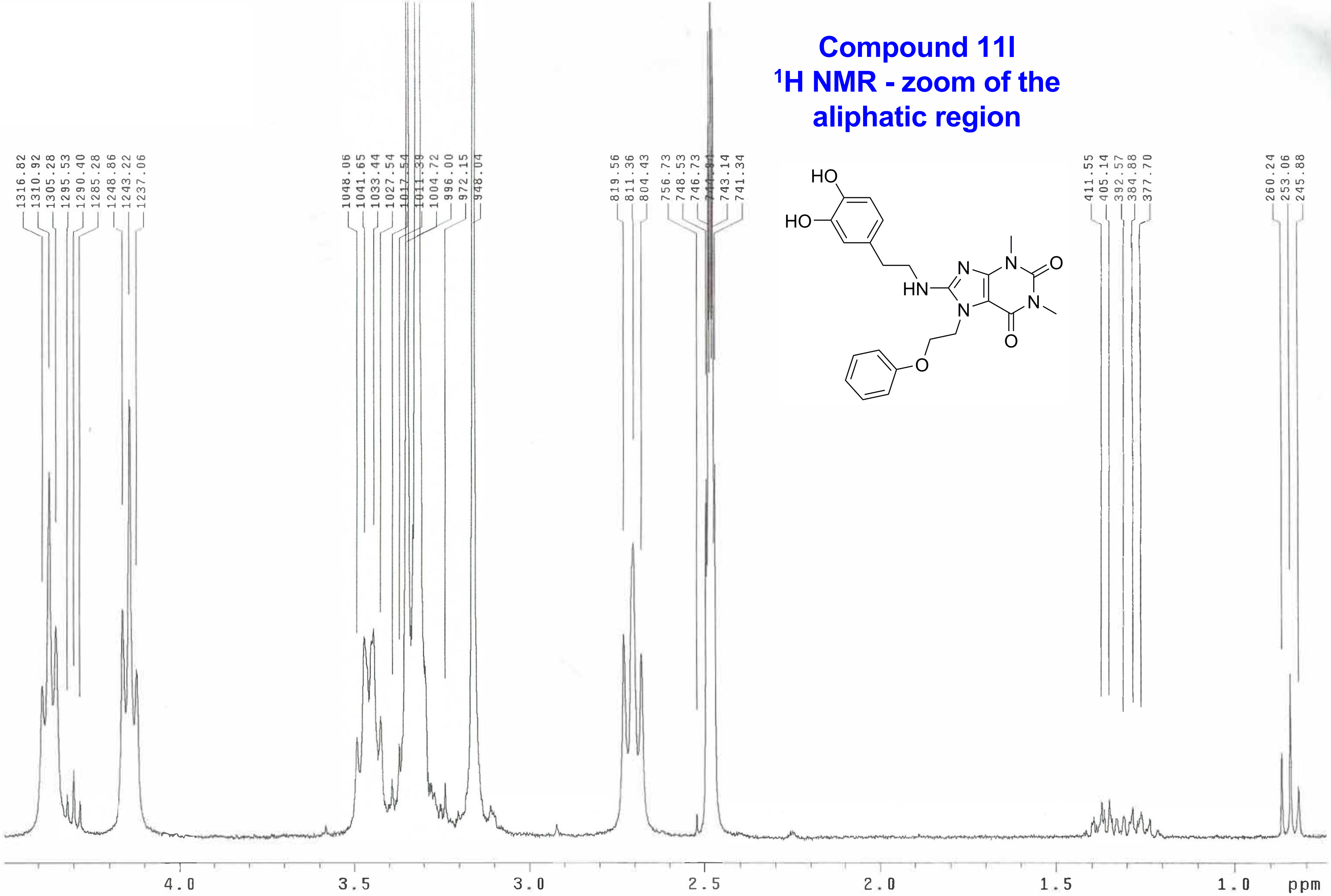

Compound 11l
^{1}H NMR - zoom of the aromatic region

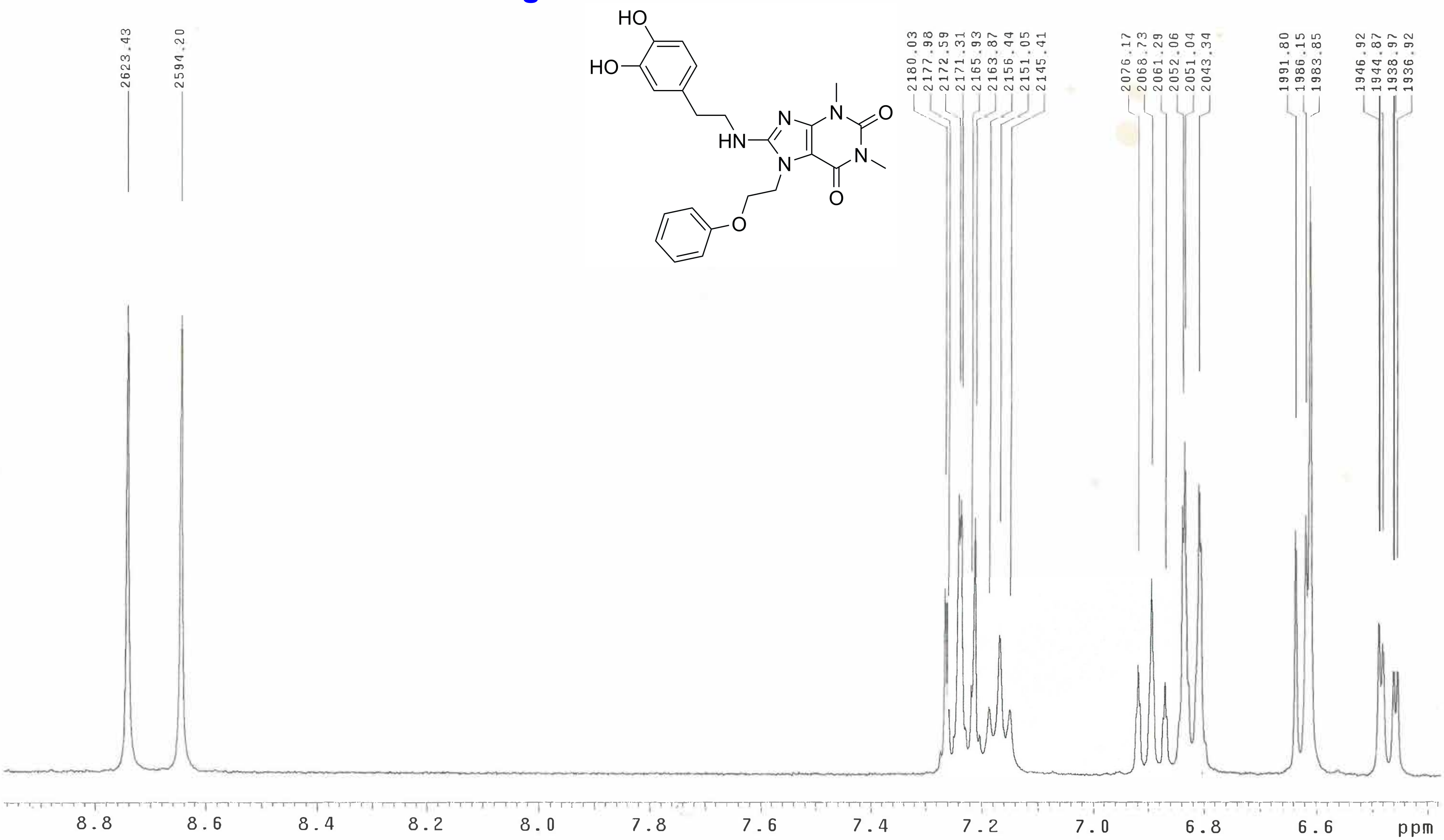

Table of peaks

INDEX	FREQUENCY	PPM	HEIGHT	INDEX	FREQUENCY	PPM	HEIGHT
1	2623.433	8.742	47.5	40	1027.543	3.424	7.3
2	2594.198	8.645	46.6	41	1017.542	3.391	3.5
3	2182.339	7.273	2.5	42	1011.387	3.370	5.6
4	2180.031	7.265	19.0	43	1004.719	3.348	150.2
5	2177.979	7.258	6.6	44	1001.642	3.338	12.8
6	2172.594	7.240	28.5	45	1001.129	3.336	12.3
7	2171.312	7.236	28.0	46	1000.616	3.334	13.9
8	2165.926	7.218	9.1	47	999.590	3.331	18.9
9	2163.874	7.211	26.1	48	996.000	3.319	1145.0
10	2161.566	7.203	4.1	49	989.845	3.299	10.7
11	2156.437	7.186	7.0	50	988.819	3.295	10.3
12	2151.052	7.168	14.3	51	986.768	3.288	3.9
13	2145.410	7.149	6.7	52	985.742	3.285	3.2
14	2076.168	6.919	11.3	53	985.229	3.283	3.1
15	2075.143	6.915	7.3	54	984.716	3.282	3.2
16	2068.731	6.894	20.1	55	984.203	3.280	3.3
17	2061.294	6.869	9.5	56	983.178	3.276	3.1
18	2052.062	6.838	27.5	57	972.150	3.240	3.3
19	2051.036	6.835	34.0	58	948.044	3.159	138.3
20	2043.343	6.809	29.7	59	819.562	2.731	12.3
21	2042.317	6.806	23.8	60	811.356	2.704	17.8
22	1991.796	6.638	25.0	61	804.432	2.681	11.1
23	1986.154	6.619	26.6	62	748.526	2.494	21.7
24	1983.846	6.611	60.0	63	746.730	2.488	48.8
25	1946.918	6.488	15.6	64	744.935	2.482	68.8
26	1944.866	6.481	13.5	65	743.140	2.476	49.0
27	1938.968	6.461	10.6	66	741.345	2.470	22.6
28	1936.916	6.455	10.7	67	260.244	0.867	5.1
29	1316.819	4.388	9.1	68	253.064	0.843	9.9
30	1310.921	4.369	22.1	69	245.883	0.819	3.1
31	1305.279	4.350	12.8				
32	1295.534	4.317	2.6				
33	1290.405	4.300	4.1				
34	1248.860	4.162	13.8				
35	1243.218	4.143	26.5				
36	1237.063	4.122	10.2				
37	1048.059	3.493	6.0				
38	1041.648	3.471	12.1				
39	1033.442	3.444	12.6				

Compound 11l
^{13}C NMR

No.	(ppm)	(Hz)	Height
1	27.68	2785.9	0.0937
2	29.73	2992.0	0.1041
3	35.15	3537.1	0.0795
4	39.35	3959.7	0.1367
5	39.55	3980.2	0.4247
6	39.76	4001.5	0.8660
7	39.97	4022.8	1.0000
8	40.18	4043.3	0.8636
9	40.39	4064.6	0.4373
10	40.59	4085.1	0.1407
11	42.35	4261.9	0.0473
12	44.92	4520.9	0.0674
13	66.15	6657.1	0.0668
14	101.81	10245.1	0.0729
15	114.76	11548.7	0.2115
16	115.95	11668.3	0.0888
17	116.50	11723.3	0.0867
18	119.76	12052.0	0.0888
19	121.23	12200.1	0.0885
20	129.95	13077.5	0.1950
21	130.53	13135.5	0.0810
22	144.05	14496.3	0.0981
23	145.57	14648.9	0.1032
24	149.24	15018.6	0.0565
25	151.42	15238.0	0.0684
26	153.15	15411.8	0.0663
27	154.49	15546.8	0.0697
28	158.43	15943.7	0.0840

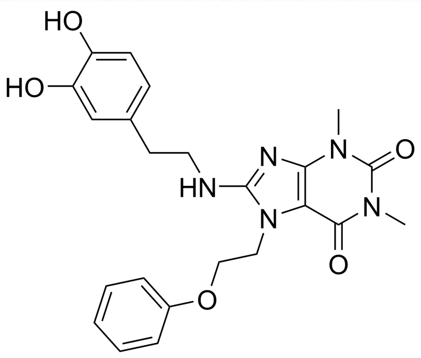

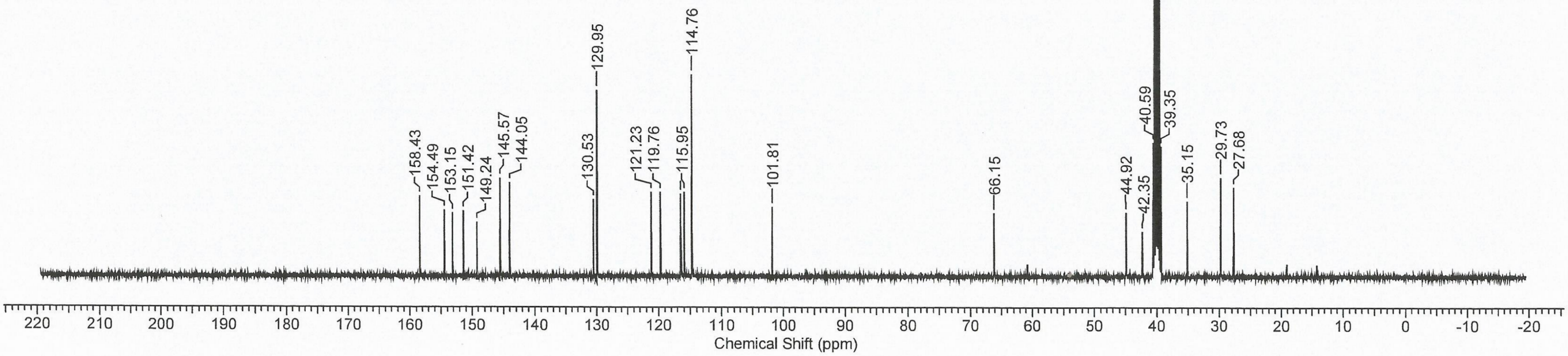

Compound 12
^{1}H NMR - zoom of the aromatic region

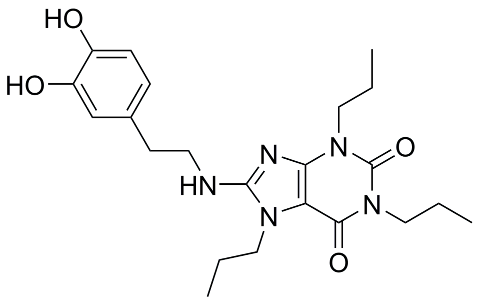

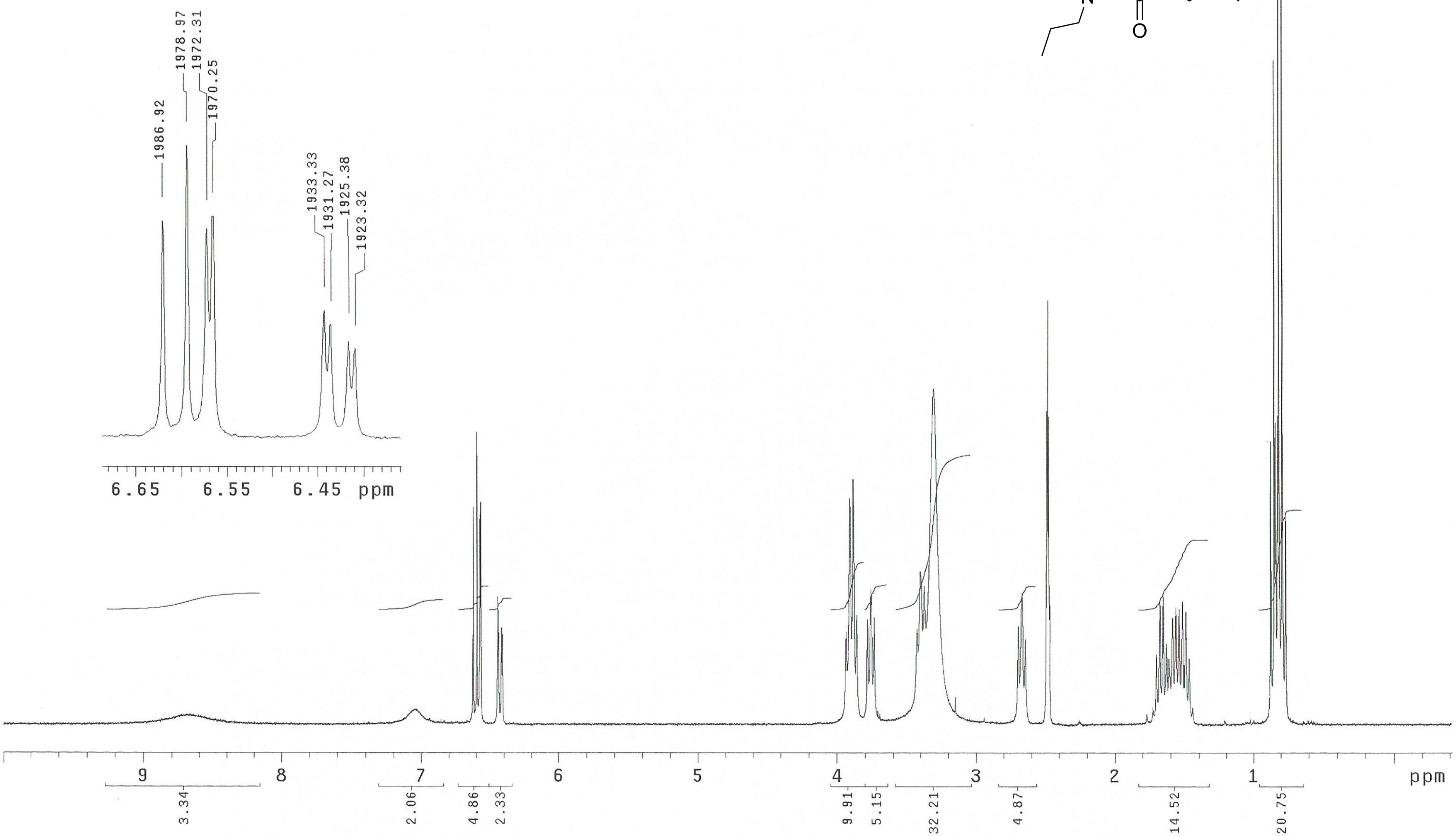

Compound 12
^{1}H NMR - zoom of the aliphatic region

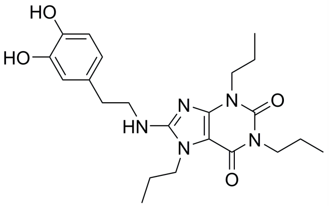

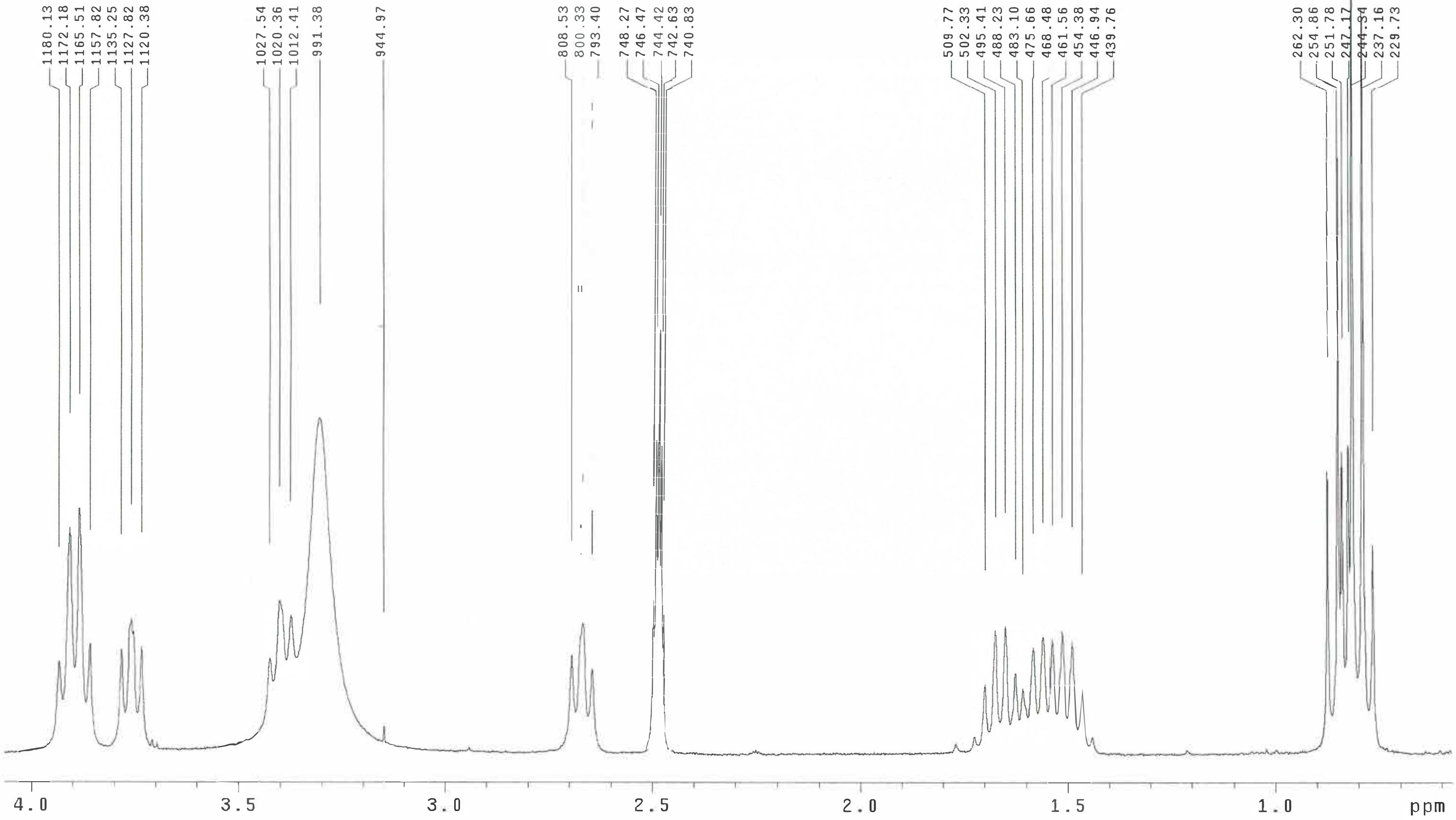

Table of peaks

INDEX	FREQUENCY	PPM	HEIGHT
1	2111.302	7.036	2.7
2	1986.924	6.621	38.0
3	1978.974	6.595	51.1
4	1972.306	6.573	36.6
5	1970.255	6.566	38.8
6	1933.326	6.443	22.3
7	1931.274	6.436	19.9
8	1925.376	6.416	16.8
9	1923.324	6.409	15.6
10	1180.131	3.933	16.1
11	1172.181	3.906	39.5
12	1165.514	3.884	42.8
13	1157.820	3.858	19.1
14	1135.253	3.783	18.3
15	1127.815	3.758	23.5
16	1120.378	3.734	18.6
17	1027.543	3.424	16.7
18	1020.363	3.400	26.7
19	1012.413	3.374	24.1
20	991.384	3.304	58.6
21	944.966	3.149	4.7
22	808.535	2.694	17.1
23	800.329	2.667	22.8
24	793.404	2.644	14.8
25	748.269	2.494	26.7
26	746.474	2.488	54.7
27	744.422	2.481	74.1
28	742.627	2.475	53.8
29	740.832	2.469	24.2
30	517.207	1.724	3.0
31	509.770	1.699	12.1
32	502.333	1.674	21.5
33	495.409	1.651	22.2
34	488.229	1.627	14.0
35	483.100	1.610	11.4
36	475.663	1.585	18.6
37	468.482	1.561	20.4
38	461.558	1.538	20.1
39	454.377	1.514	21.4
40	446.940	1.489	19.7
41	439.760	1.465	11.5
42	432.322	1.441	2.9
43	262.296	0.874	49.4
44	254.859	0.849	115.8
45	251.781	0.839	52.8
46	247.165	0.824	54.0
47	244.344	0.814	144.0
48	237.164	0.790	130.0
49	229.727	0.766	36.5

Compound 12
^{13}C NMR

No.	(ppm)	(Hz)	Height
1	11.00	1106.7	0.0989
2	11.55	1162.5	0.0923
3	11.64	1171.3	0.0788
4	21.36	2149.1	0.0634
5	21.45	2158.7	0.0616
6	22.87	2301.0	0.0570
7	35.18	3540.0	0.0423
8	39.35	3960.4	0.1396
9	39.56	3980.9	0.4161
10	39.77	4002.2	0.8570
11	39.98	4023.5	1.0000
12	40.19	4044.0	0.8434
13	40.40	4065.3	0.4290
14	40.61	4086.6	0.1355
15	41.92	4218.6	0.0337
16	44.14	4442.4	0.0315
17	44.28	4456.3	0.0345
18	44.95	4523.8	0.0410
19	101.93	10257.6	0.0475
20	115.90	11663.1	0.0541
21	116.58	11731.4	0.0545
22	119.74	12049.8	0.0571
23	130.61	13143.5	0.0420
24	143.99	14490.4	0.0466
25	145.52	14644.5	0.0652
26	148.87	14981.2	0.0372
27	150.95	15190.3	0.0415
28	152.93	15389.8	0.0365
29	153.77	15474.2	0.0539

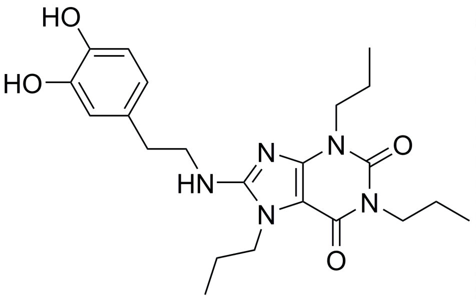

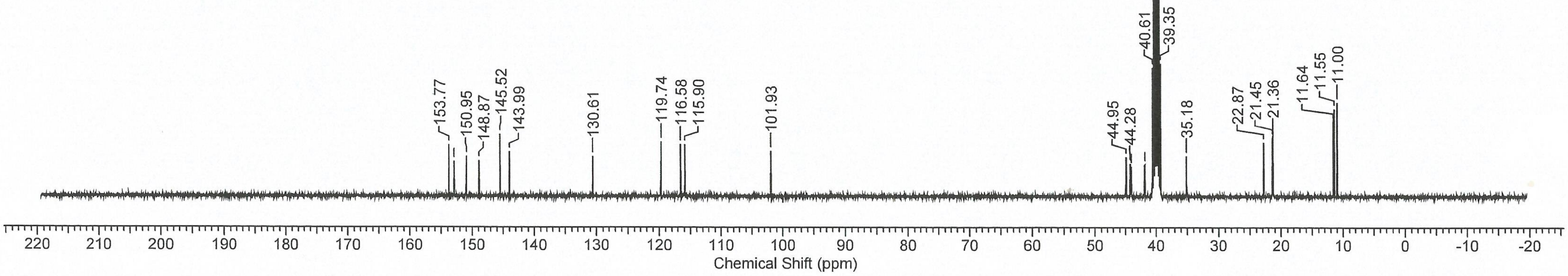

Compound 13
^{1}H NMR - zoom of the aromatic region

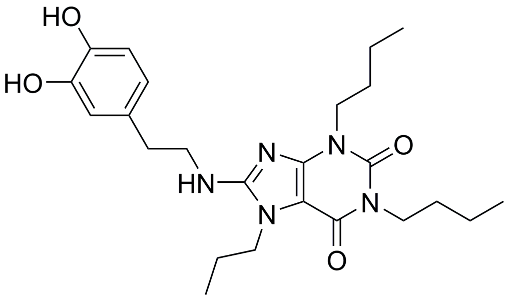

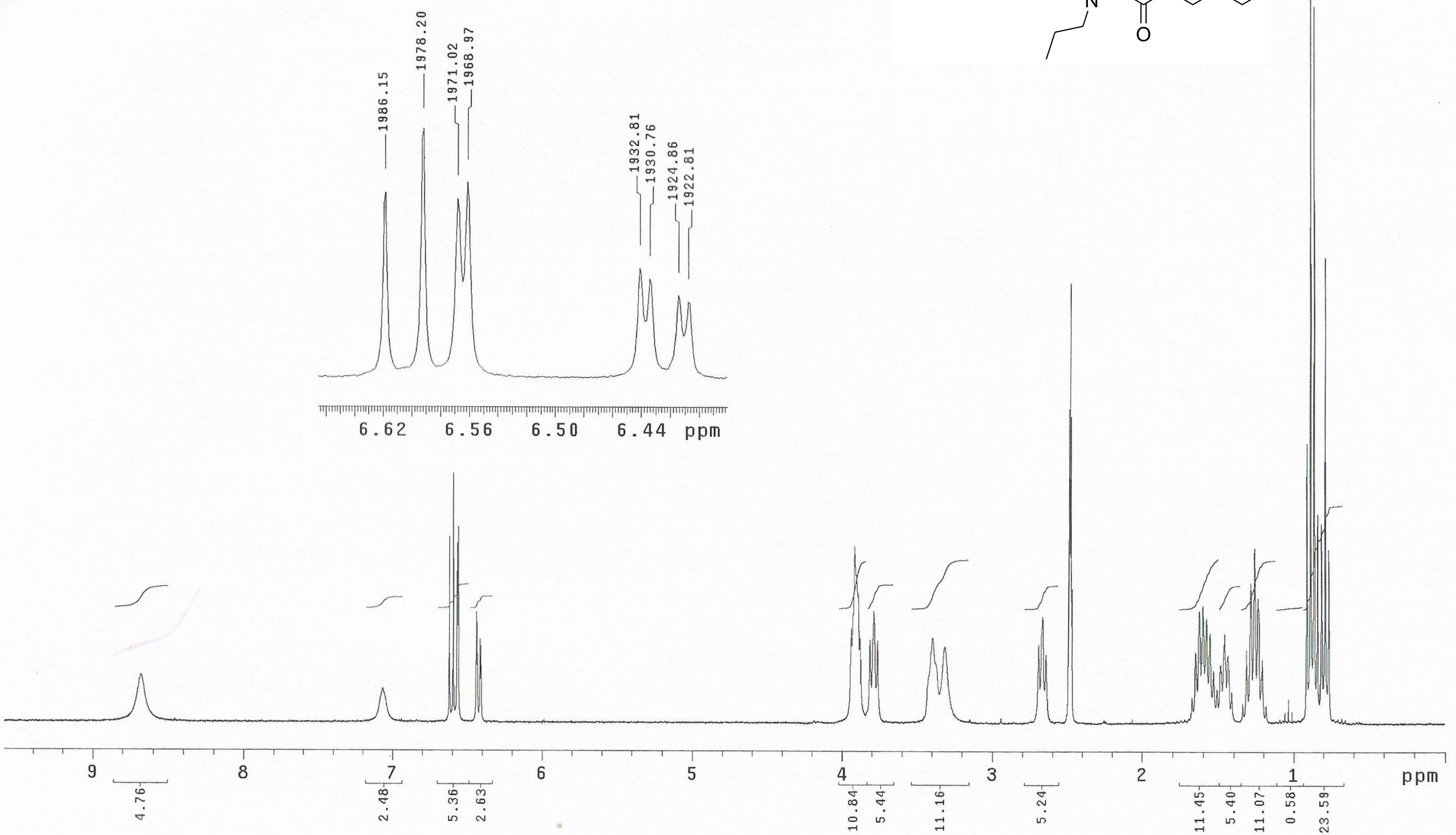

Compound 13

^{1}H NMR - zoom of the aliphatic region

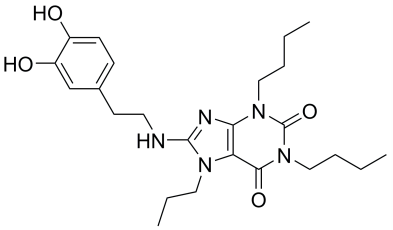

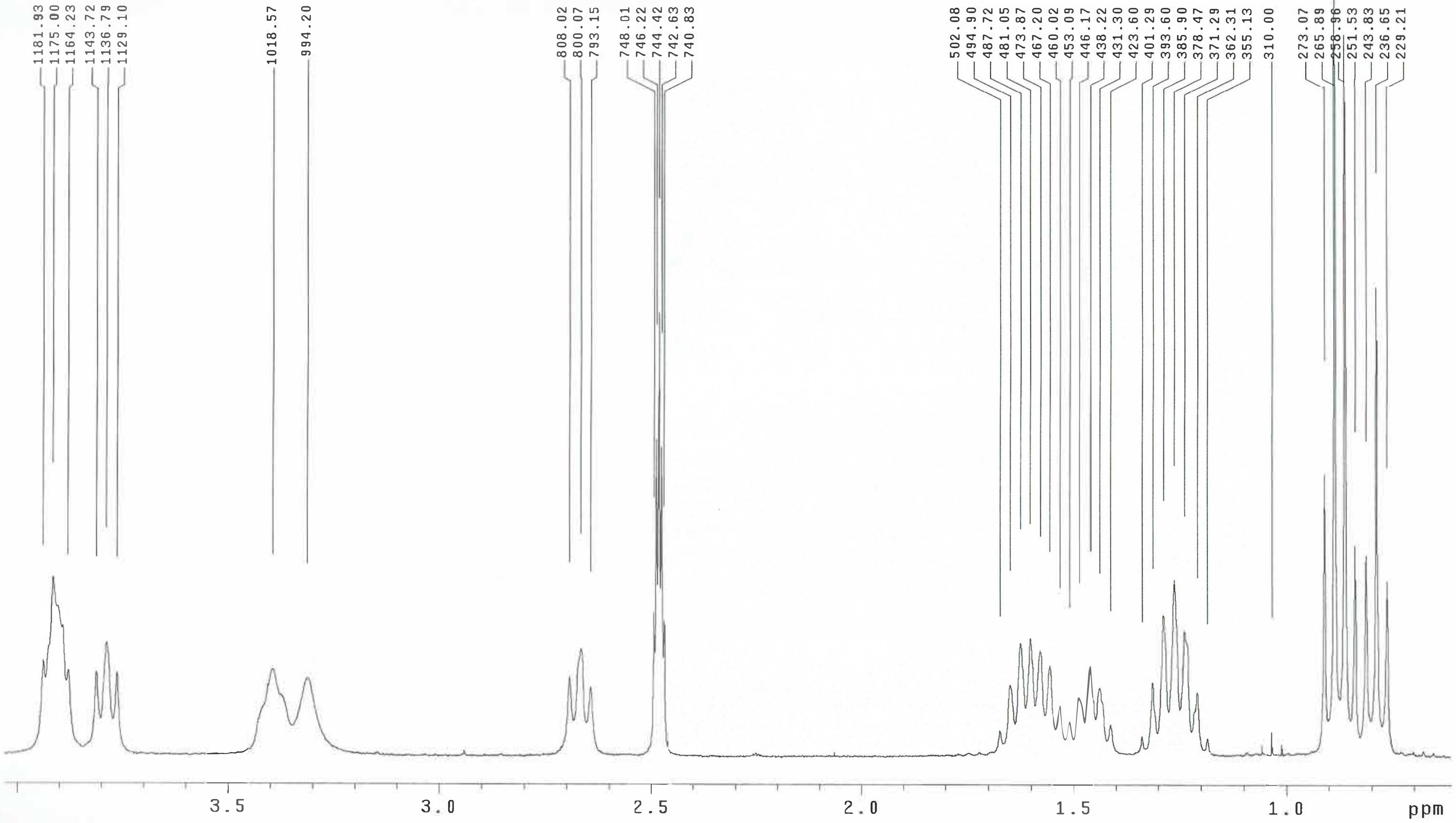

Table of peaks

INDEX	FREQUENCY	PPM	HEIGHT
1	2604.969	8.681	8.3
2	2120.278	7.066	5.9
3	1986.154	6.619	32.5
4	1978.204	6.592	43.7
5	1971.024	6.568	31.4
6	1968.972	6.561	34.3
7	1932.813	6.441	19.3
8	1930.761	6.434	17.5
9	1924.863	6.414	14.5
10	1922.811	6.408	13.4
11	1181.926	3.939	16.5
12	1175.002	3.916	30.9
13	1168.078	3.893	22.6
14	1164.231	3.880	14.8
15	1143.715	3.811	14.5
16	1136.791	3.788	19.6
17	1129.098	3.763	14.4
18	1018.568	3.394	15.0
19	994.205	3.313	13.4
20	808.022	2.693	13.7
21	800.072	2.666	18.6
22	793.148	2.643	12.1
23	748.013	2.493	25.0
24	746.218	2.487	55.1
25	744.422	2.481	77.2
26	742.627	2.475	53.8
27	740.832	2.469	23.5
28	502.077	1.673	4.5
29	494.896	1.649	12.5
30	487.716	1.625	19.6
31	481.048	1.603	20.6
32	473.867	1.579	18.4
33	467.200	1.557	15.8
34	460.019	1.533	9.3
35	453.095	1.510	6.0
36	446.171	1.487	10.2
37	438.221	1.460	15.8
38	431.297	1.437	12.0
39	423.603	1.412	5.5

INDEX	FREQUENCY	PPM	HEIGHT
40	401.292	1.337	3.5
41	393.598	1.312	12.8
42	385.905	1.286	24.6
43	378.468	1.261	30.7
44	371.287	1.237	21.9
45	362.312	1.207	11.2
46	355.131	1.183	3.1
47	309.996	1.033	4.3
48	273.067	0.910	49.1
49	265.886	0.886	144.0
50	258.962	0.863	125.6
51	251.525	0.838	36.7
52	243.832	0.813	35.0
53	236.651	0.789	81.8
54	229.214	0.764	30.5

Compound 13
^{13}C NMR

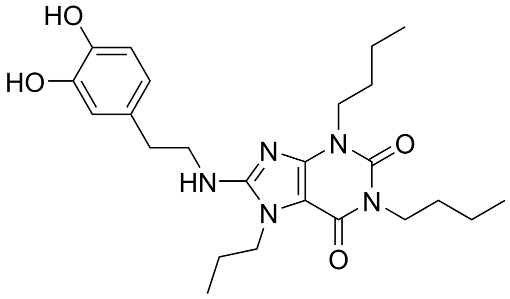

No.	(ppm)	(Hz)	Height
1	11.00	1106.7	0.1214
2	14.08	1417.0	0.1237
3	14.19	1428.0	0.1147
4	19.82	1994.4	0.1183
5	20.14	2026.6	0.0932
6	22.87	2301.7	0.0688
7	30.08	3027.3	0.0749
8	30.32	3051.5	0.0640
9	35.20	3542.2	0.0456
10	39.35	3959.7	0.1338
11	39.56	3980.9	0.4279
12	39.77	4002.2	0.8441
13	39.97	4022.8	1.0000
14	40.19	4044.0	0.8771
15	40.40	4065.3	0.4165
16	40.60	4085.8	0.1394
17	42.41	4267.8	0.0361
18	44.14	4442.4	0.0332
19	44.94	4522.3	0.0485
20	101.95	10259.0	0.0519
21	115.88	11661.7	0.0620
22	116.58	11731.4	0.0624
23	119.70	12046.1	0.0634
24	130.60	13142.8	0.0554
25	143.98	14489.0	0.0616
26	145.52	14644.5	0.0638
27	148.84	14978.3	0.0474
28	150.87	15182.2	0.0428
29	152.89	15385.4	0.0475
30	153.75	15472.0	0.0581

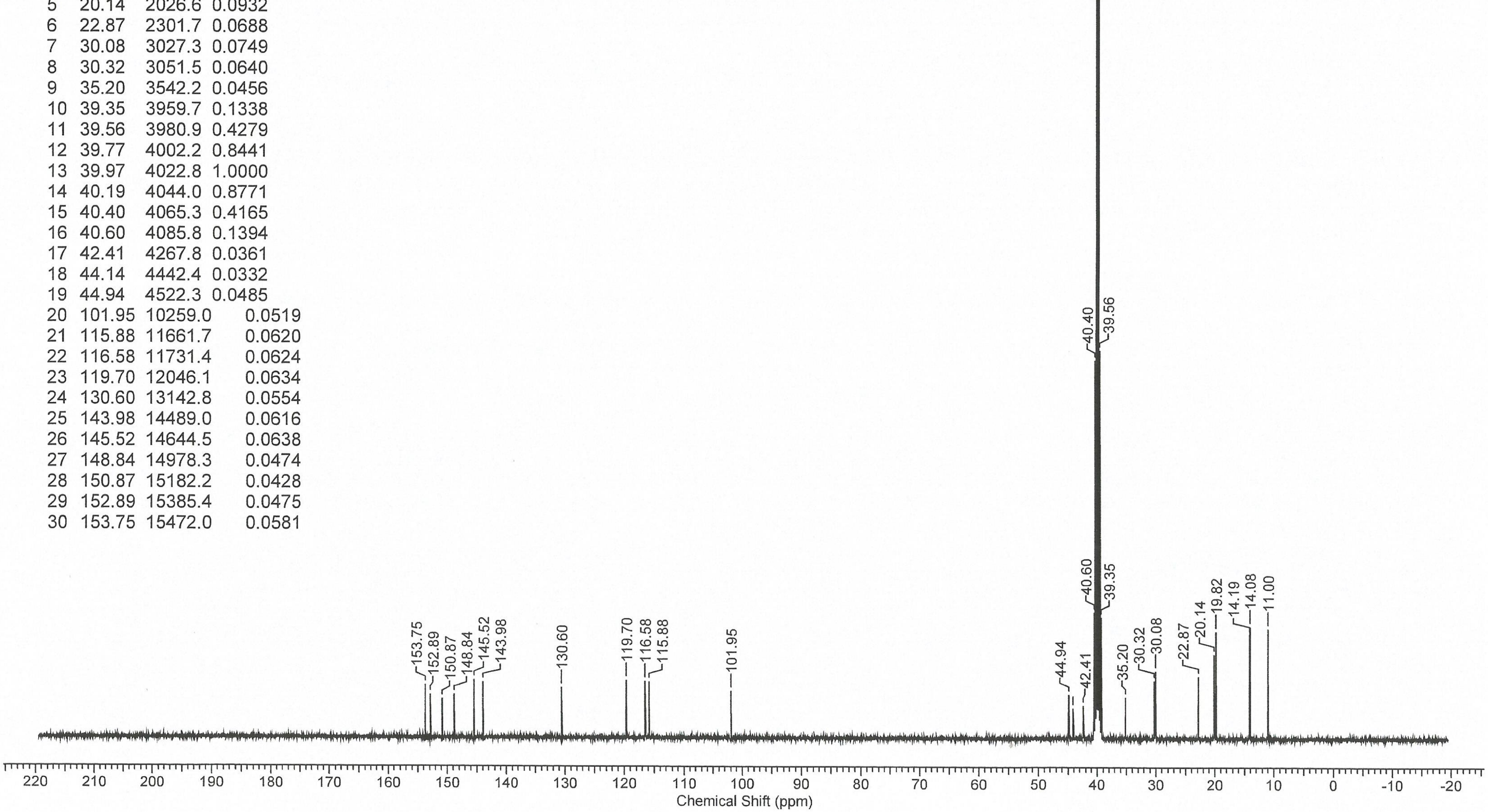

Compound 20a
^{1}H NMR

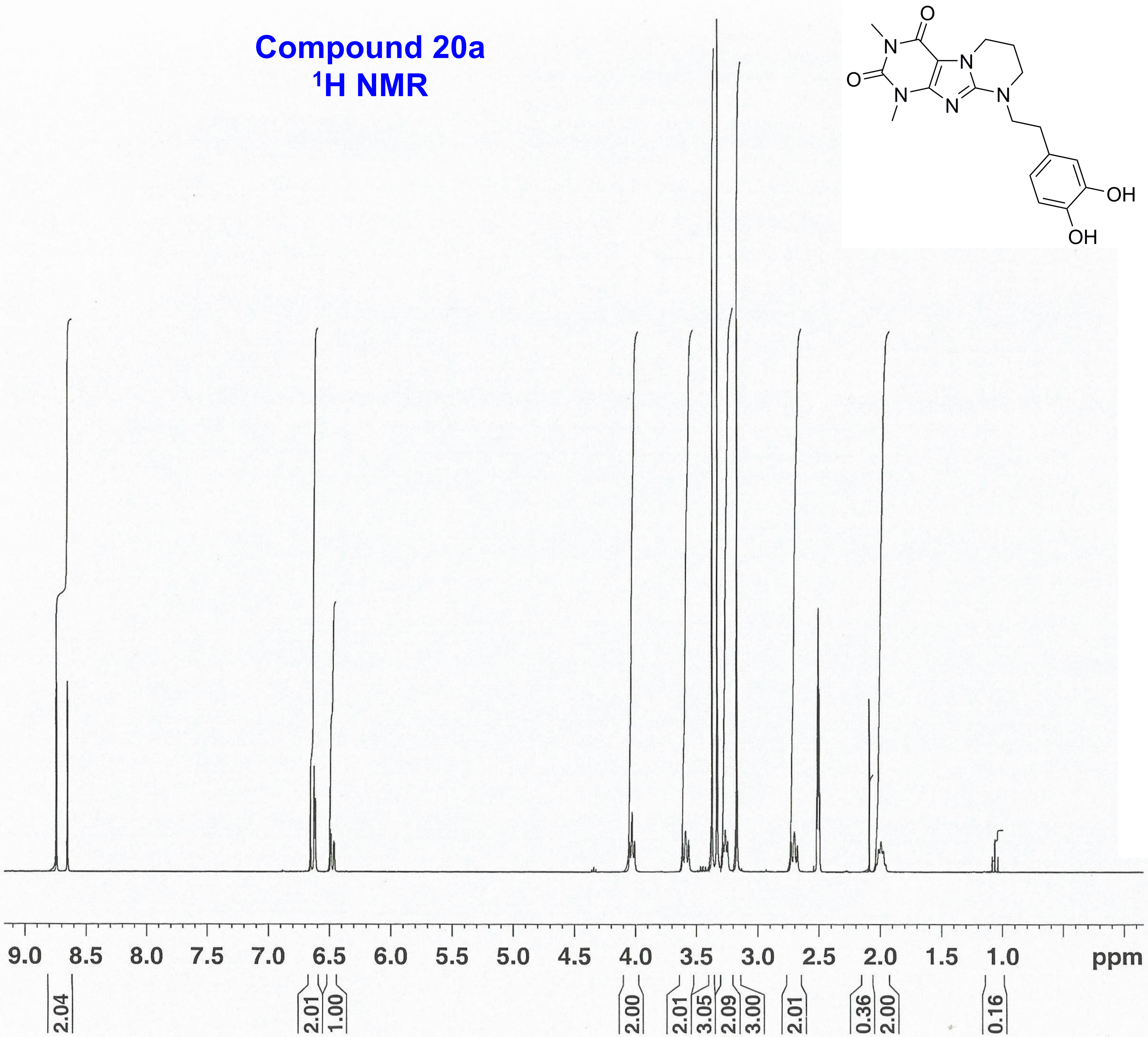

Compound 20a
1H NMR - zoom of the aliphatic region

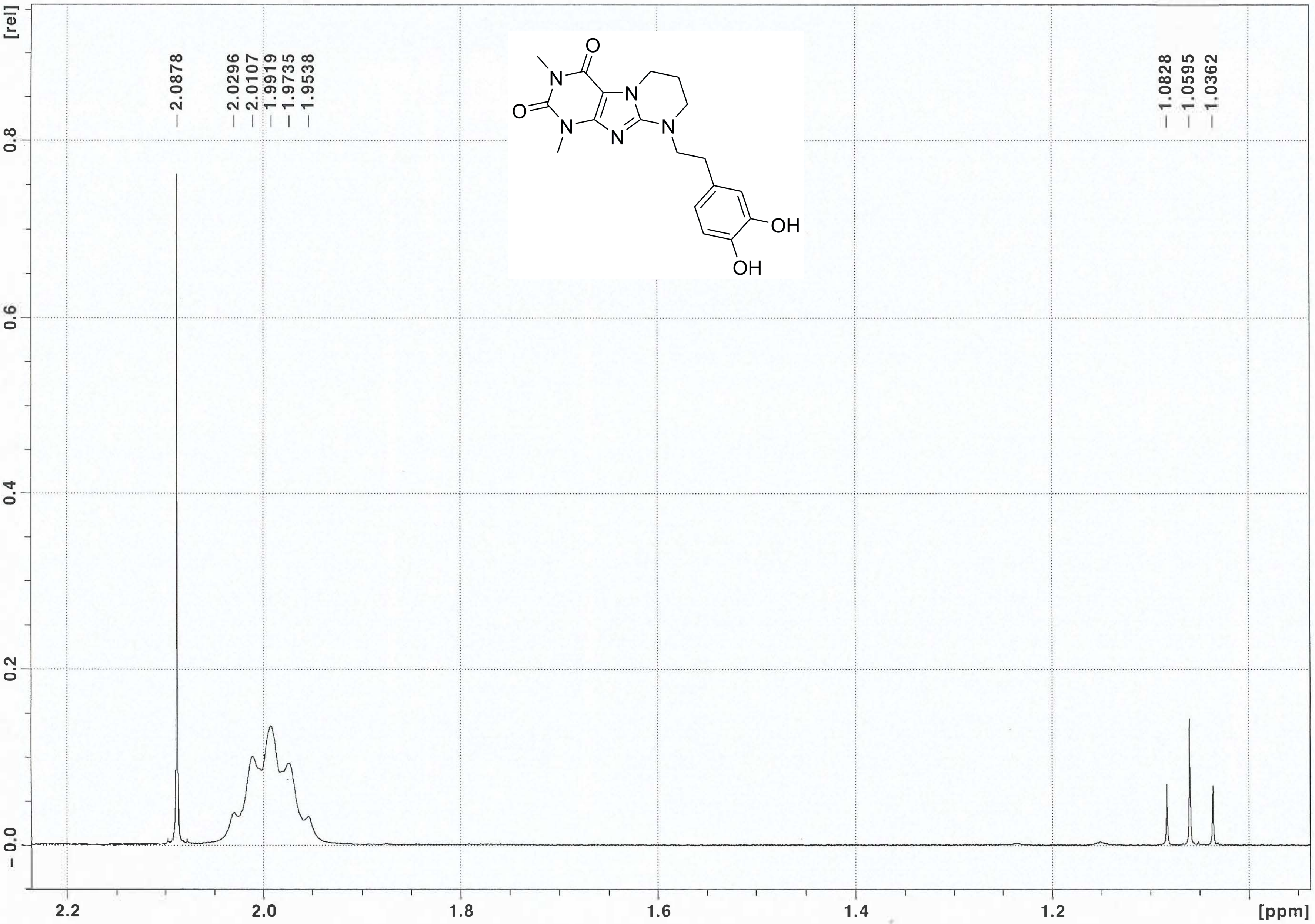

Compound 20a
^{1}H NMR - zoom of the aliphatic region

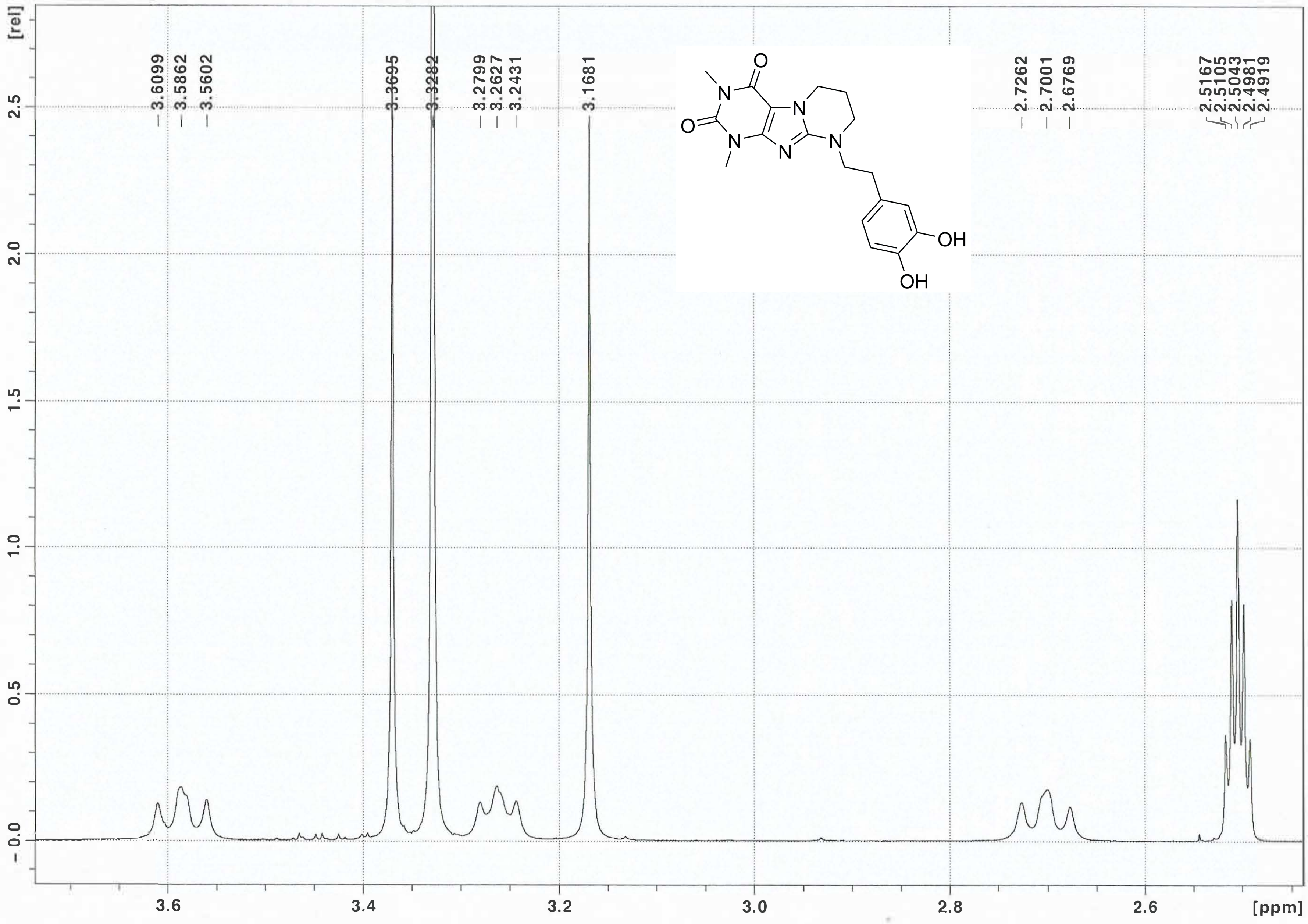

Compound 20a
^{1}H NMR - zoom of the aliphatic region

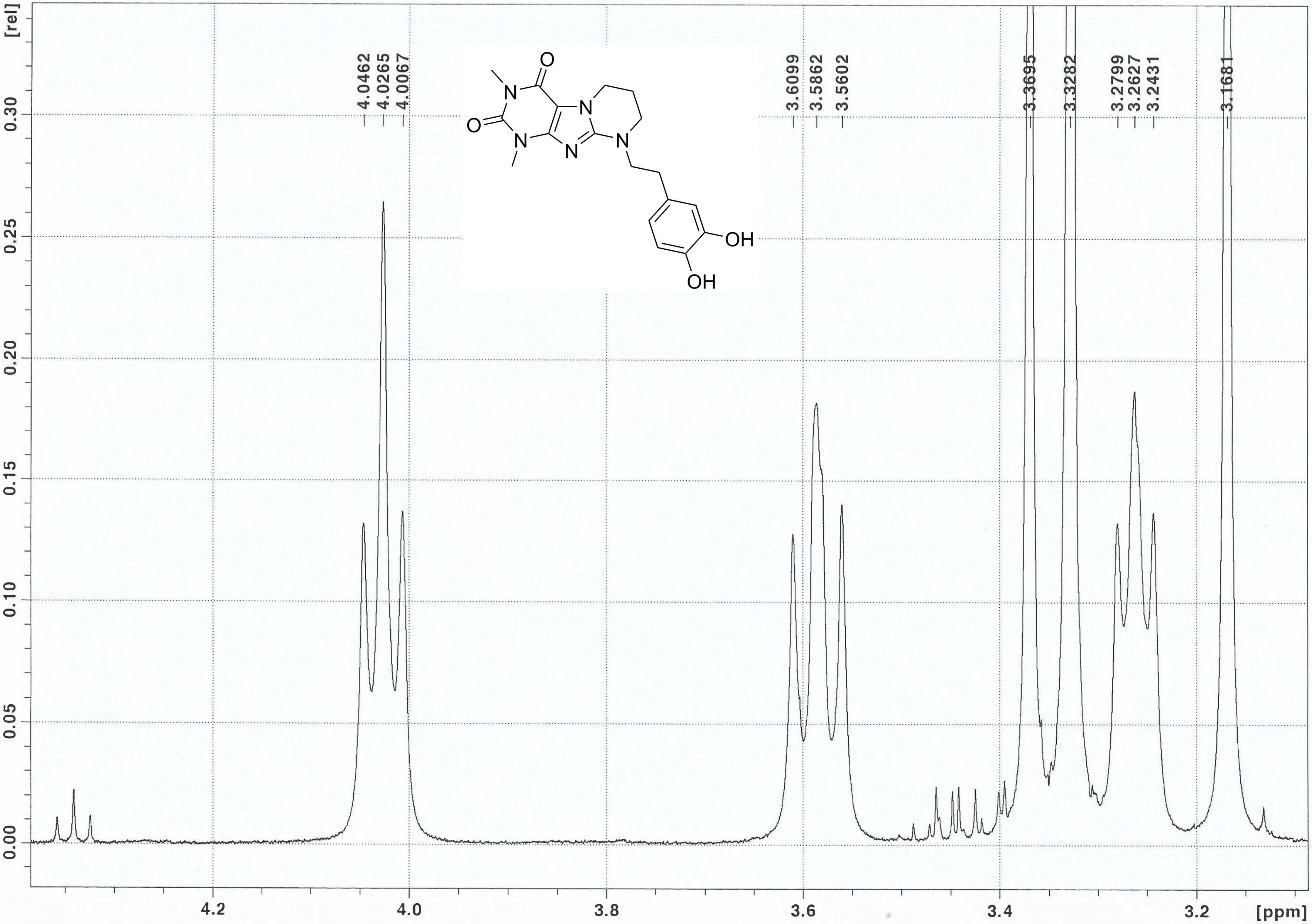

Compound 20a
¹H NMR - zoom of the aromatic region

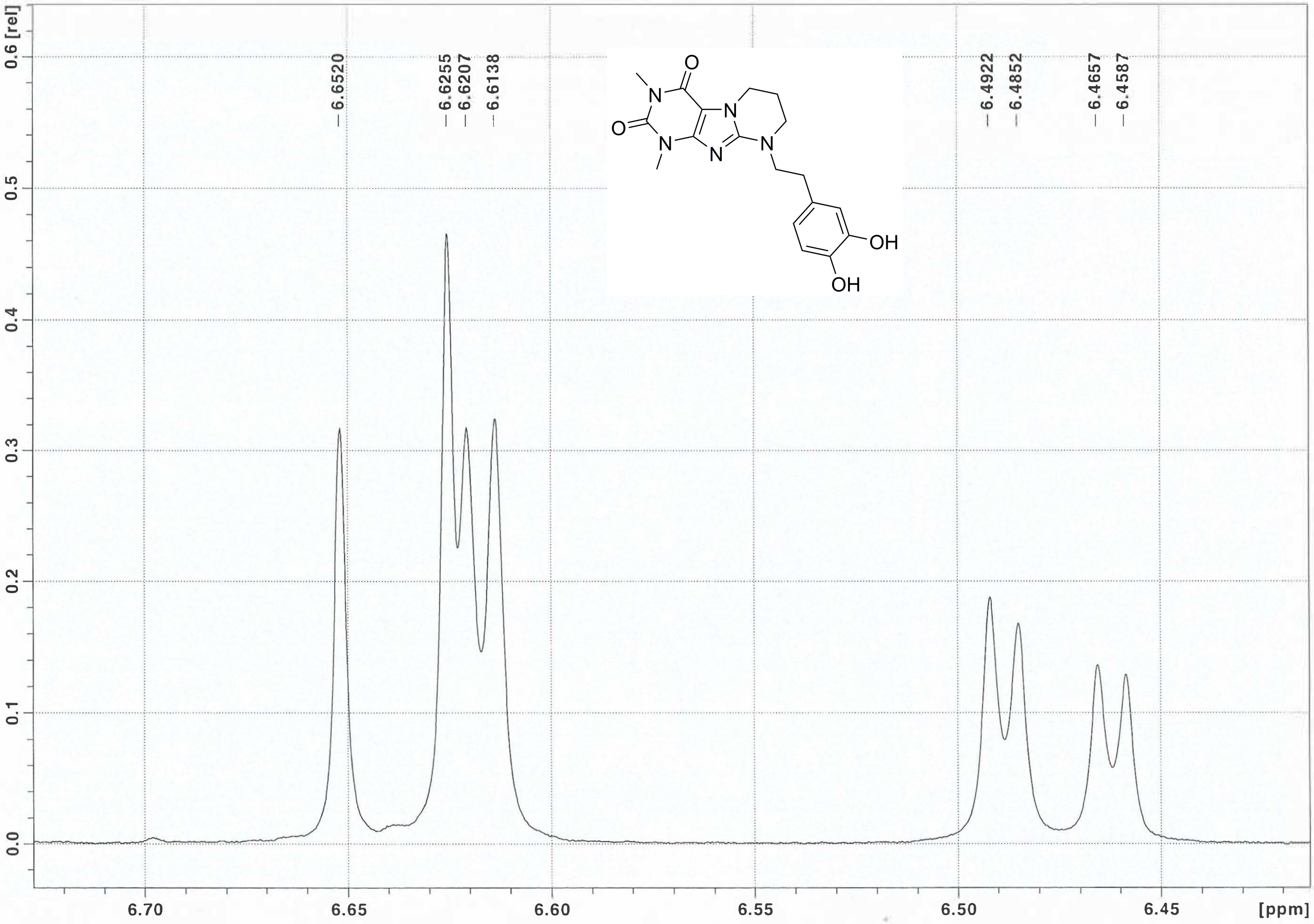

Compound 20a
^{1}H NMR - zoom of the aromatic region

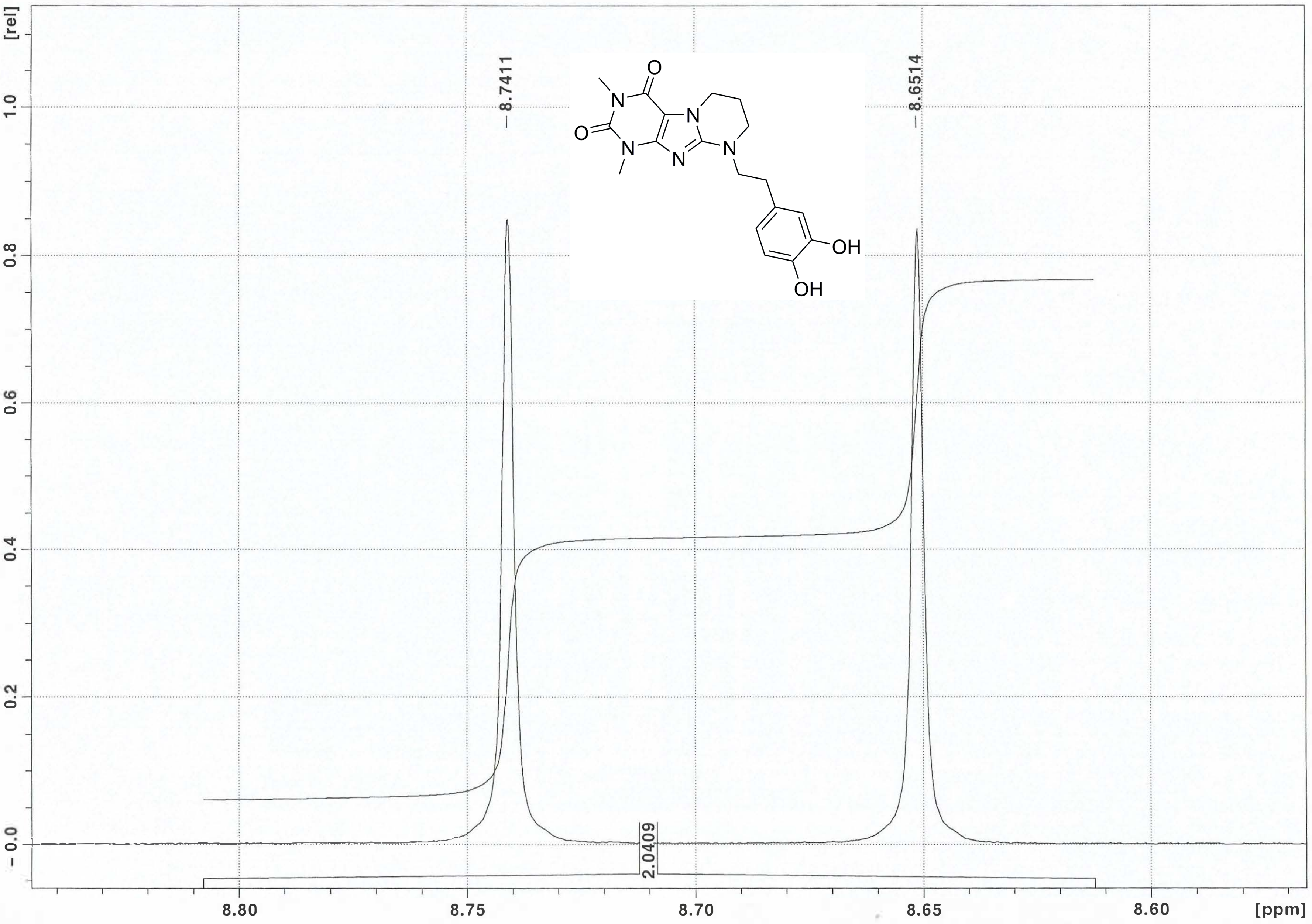

Table of peaks

Peak	v(F1) [ppm]	v(F1) [Hz]	Intensity
1	8.7411	2623.4663	0.84
2	8.6514	2596.5447	0.82
3	6.6520	1996.4648	0.31
4	6.6255	1988.5113	0.46
5	6.6207	1987.0707	0.31
6	6.6138	1984.9998	0.32
7	6.4922	1948.5040	0.19
8	6.4852	1946.4031	0.17
9	6.4657	1940.5505	0.13
10	6.4587	1938.4496	0.13
11	4.0462	1214.3860	0.13
12	4.0265	1208.4734	0.26
13	4.0067	1202.5309	0.14
14	3.6099	1083.4393	0.13
15	3.5862	1076.3262	0.18
16	3.5602	1068.5228	0.14
17	3.3695	1011.2880	2.68
18	3.3282	998.8927	15.00
19	3.2799	984.3964	0.13
20	3.2627	979.2342	0.18
21	3.2431	973.3516	0.13
22	3.1681	950.8419	2.45
23	2.7262	818.2144	0.13
24	2.7001	810.3810	0.18
25	2.6769	803.4180	0.12
26	2.5167	755.3372	0.36
27	2.5105	753.4764	0.81
28	2.5043	751.6156	1.15
29	2.4981	749.7548	0.80
30	2.4919	747.8939	0.34
31	2.0878	626.6114	0.76
32	2.0296	609.1438	0.04
33	2.0107	603.4714	0.10
34	1.9919	597.8289	0.13
35	1.9735	592.3066	0.09
36	1.9538	586.3940	0.03
37	1.0828	324.9808	0.07
38	1.0595	317.9877	0.14
39	1.0362	310.9947	0.00

Compound 20a
^{13}C NMR

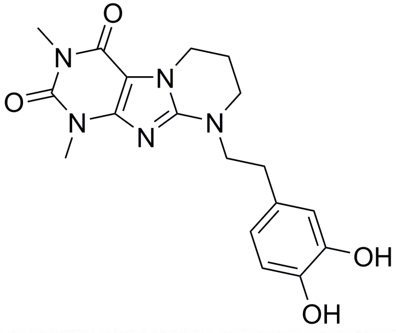

No.	(ppm)	(Hz)	Height
1	21.15	2128.6	0.0830
2	27.60	2777.8	0.1235
3	29.75	2994.2	0.1513
4	32.93	3313.4	0.0859
5	39.35	3960.4	0.1422
6	39.56	3980.9	0.4364
7	39.77	4002.2	0.8793
8	39.98	4023.5	1.0000
9	40.19	4044.0	0.8772
10	40.40	4065.3	0.4377
11	40.60	4085.8	0.1402
12	41.75	4201.7	0.0752
13	44.78	4506.2	0.0786
14	51.38	5170.8	0.0819
15	102.20	10284.7	0.0735
16	116.00	11673.4	0.1017
17	116.55	11729.2	0.1105
18	119.81	12056.4	0.1006
19	130.21	13103.2	0.0956
20	144.05	14496.3	0.1031
21	145.57	14648.9	0.1004
22	148.83	14976.8	0.0719
23	151.45	15240.9	0.0732
24	151.67	15262.9	0.0816
25	152.99	15395.7	0.0836

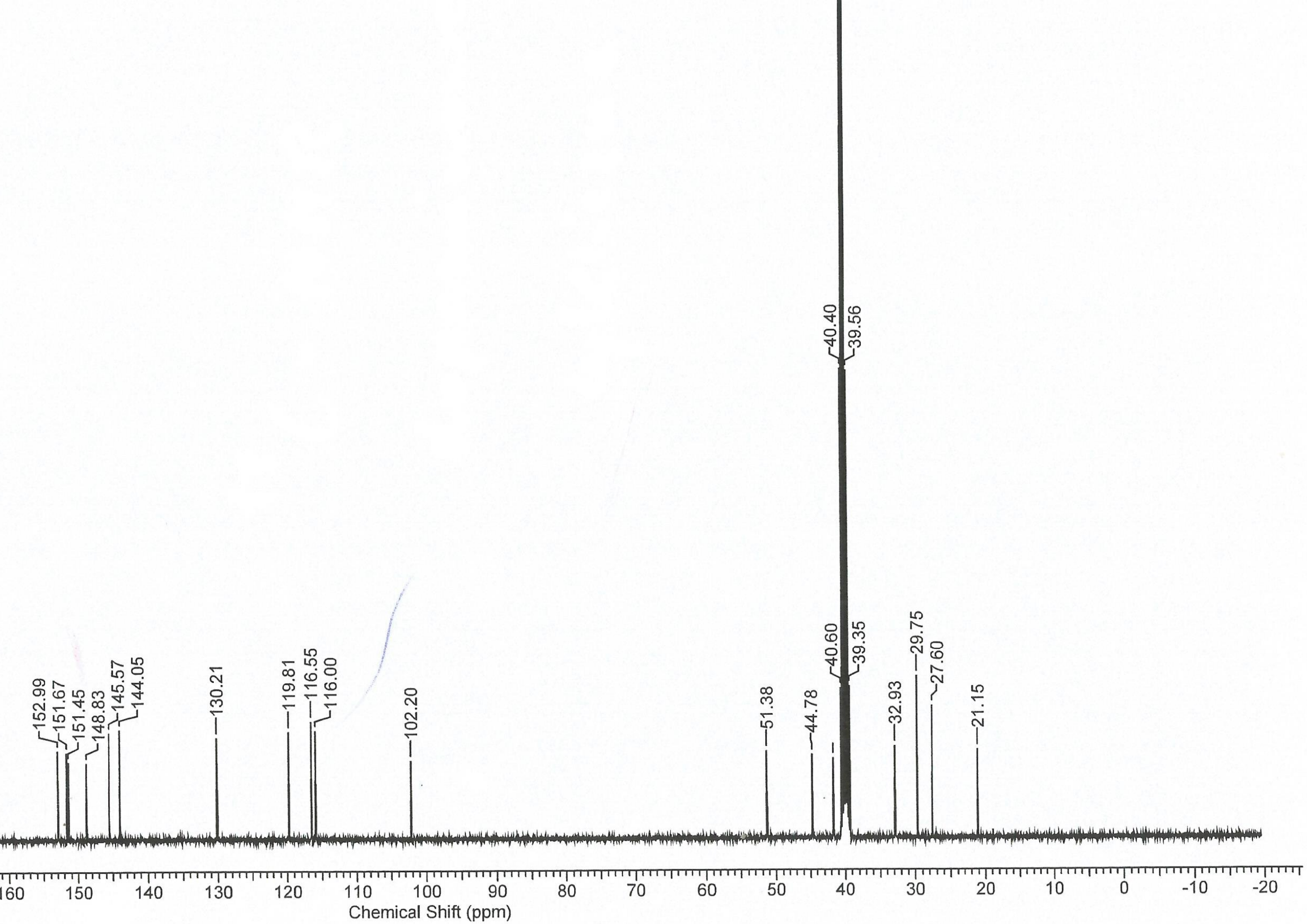

Compound 20b
^{1}H NMR - zoom of the aromatic region

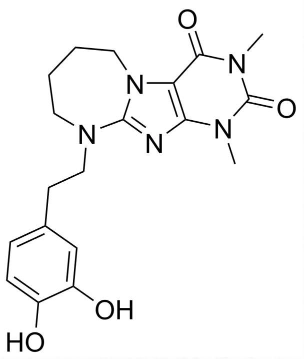

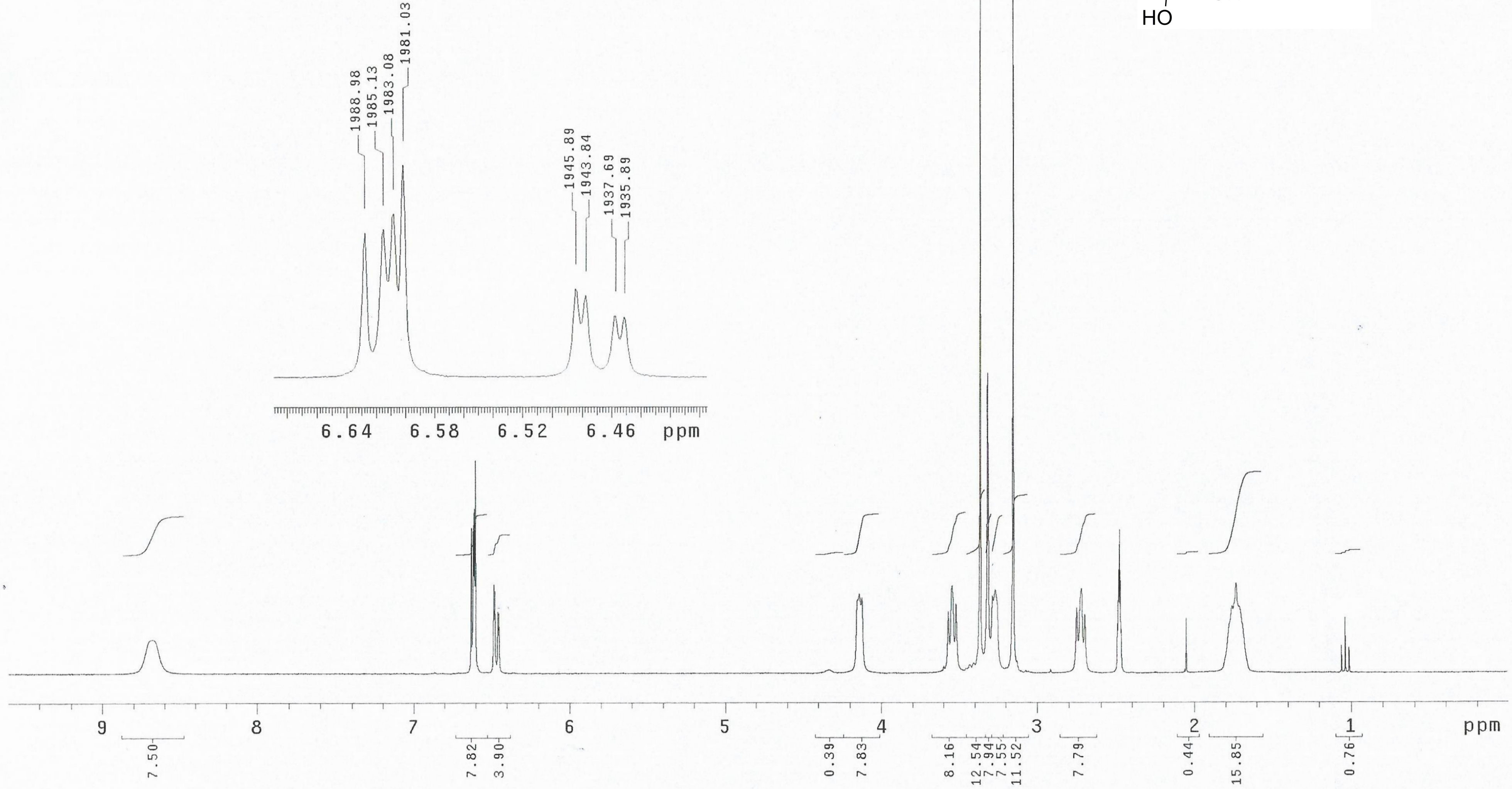

Compound 20b
^{1}H NMR - zoom of the aliphatic region

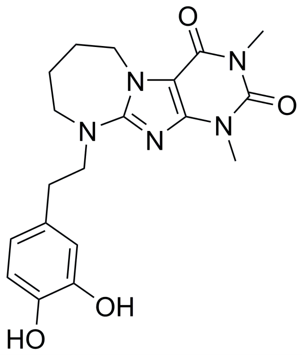

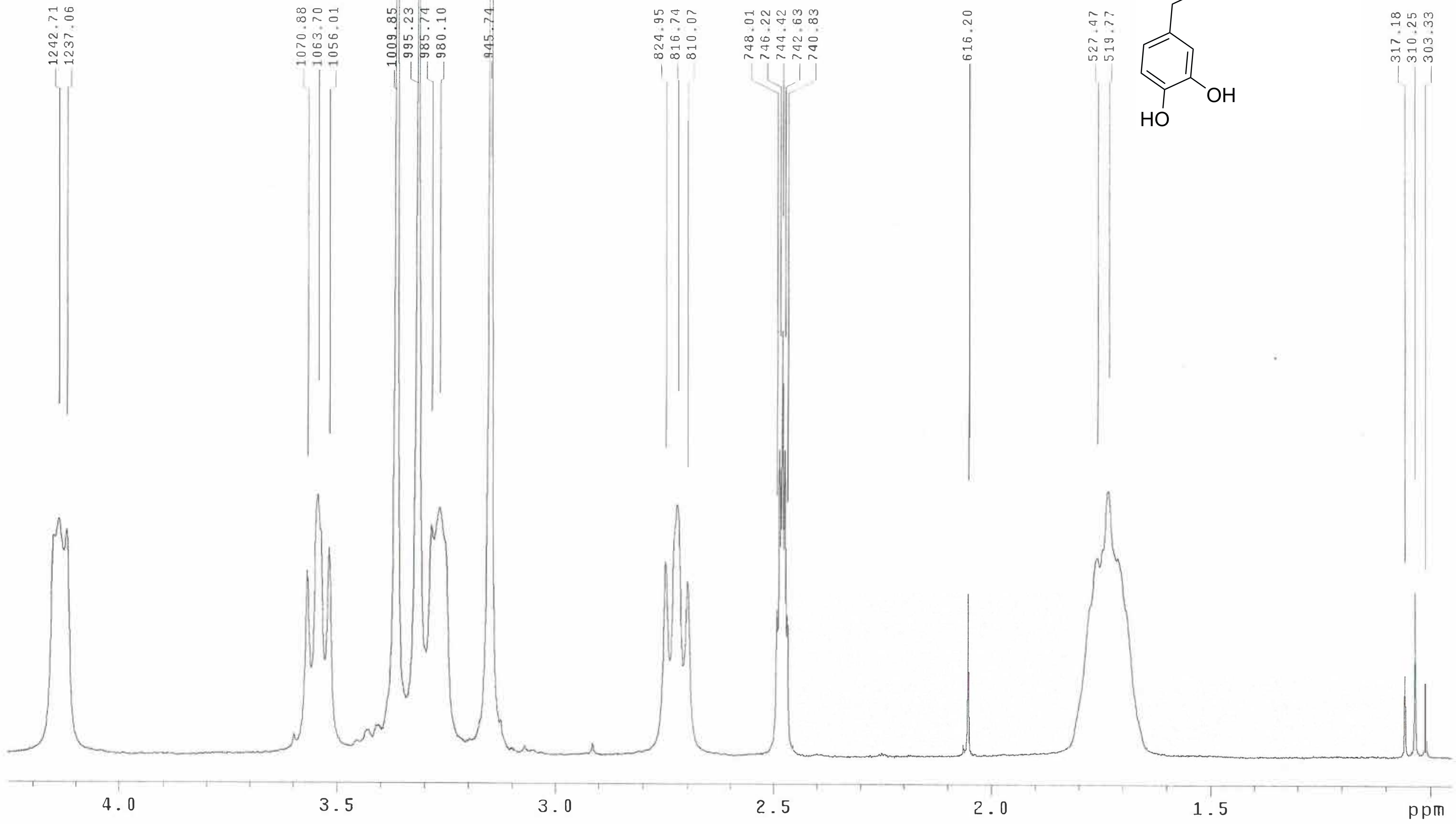

Table of peaks

INDEX	FREQUENCY	PPM	HEIGHT
1	2603.687	8.677	5.7
2	1988.975	6.628	24.5
3	1985.129	6.615	25.0
4	1983.077	6.608	27.8
5	1981.025	6.602	35.9
6	1945.892	6.485	15.0
7	1943.840	6.478	13.9
8	1937.685	6.457	10.5
9	1935.890	6.451	10.2
10	1242.705	4.141	13.3
11	1237.063	4.122	12.7
12	1070.884	3.569	10.4
13	1063.703	3.545	14.7
14	1056.009	3.519	11.7
15	1009.848	3.365	144.0
16	995.231	3.317	50.7
17	985.742	3.285	13.0
18	980.100	3.266	14.0
19	945.736	3.152	132.7
20	824.948	2.749	11.0
21	816.741	2.722	14.2
22	810.074	2.700	9.9
23	748.013	2.493	8.3
24	746.218	2.487	17.4
25	744.422	2.481	24.1
26	742.627	2.475	17.3
27	740.832	2.469	7.9
28	616.197	2.053	9.2
29	527.465	1.758	11.3
30	519.772	1.732	15.1
31	317.176	1.057	4.6
32	310.252	1.034	9.3
33	303.328	1.011	4.2

Compound 20b
^{13}C NMR

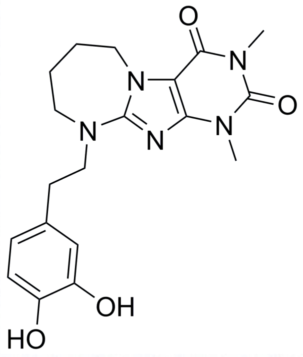

No.	(ppm)	(Hz)	Height
1	26.25	2641.4	0.1047
2	27.71	2788.1	0.1541
3	28.74	2892.3	0.1037
4	29.72	2990.6	0.1667
5	33.18	3339.0	0.0958
6	39.35	3960.4	0.1362
7	39.57	3981.7	0.4146
8	39.77	4002.2	0.8515
9	39.98	4023.5	1.0000
10	40.19	4044.0	0.8359
11	40.40	4065.3	0.4266
12	40.61	4086.6	0.1376
13	45.75	4603.8	0.0863
14	51.59	5191.4	0.0898
15	55.11	5545.7	0.0962
16	103.49	10414.6	0.0940
17	115.95	11668.3	0.1383
18	116.64	11738.0	0.1447
19	119.86	12062.2	0.1350
20	130.53	13135.5	0.1127
21	143.96	14487.5	0.1493
22	145.51	14643.0	0.1440
23	148.18	14911.5	0.1028
24	151.38	15233.6	0.0935
25	153.70	15466.8	0.0904
26	158.95	15995.8	0.1352

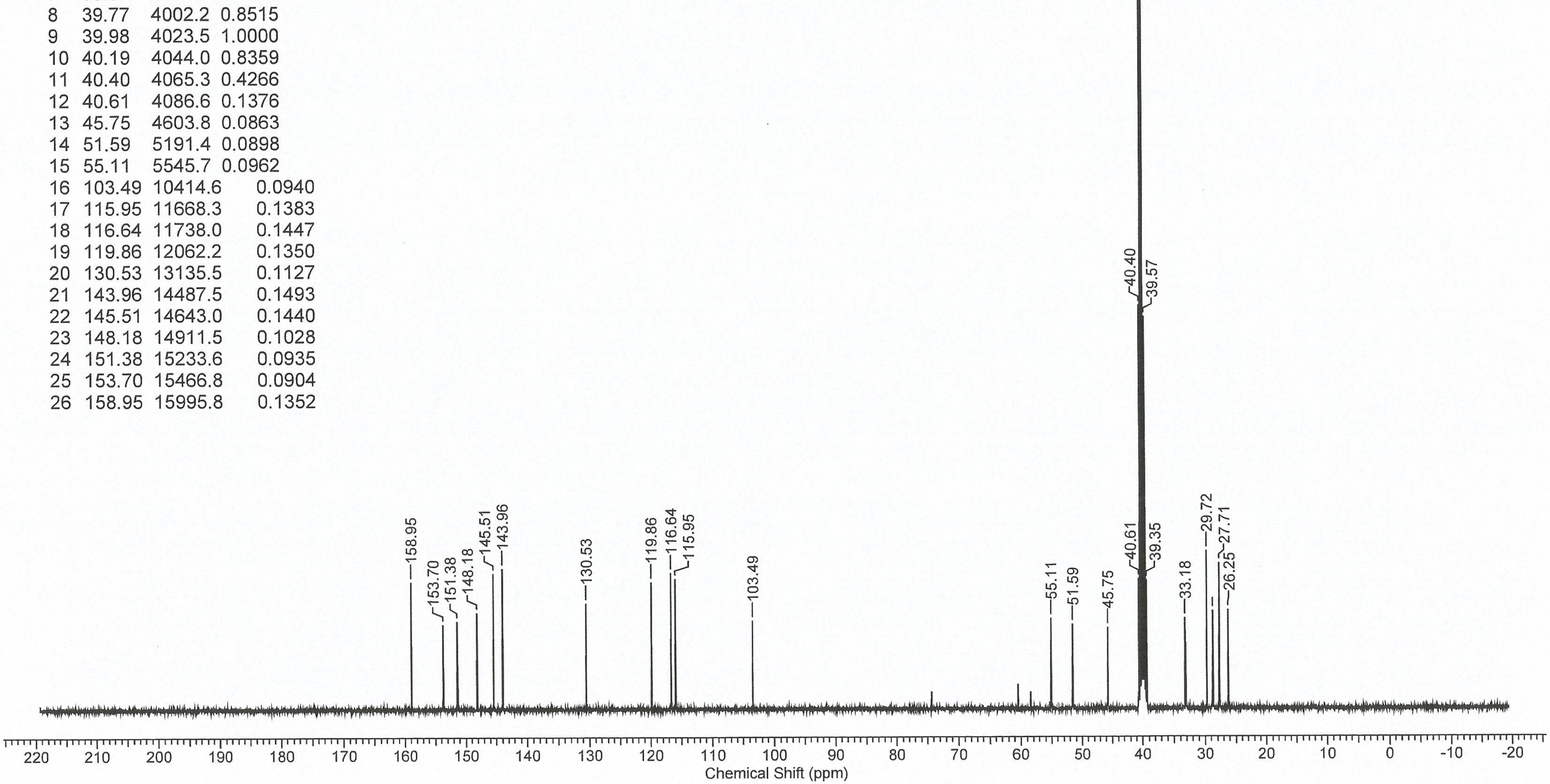

Compound 21
^{1}H NMR - zoom of the aromatic region

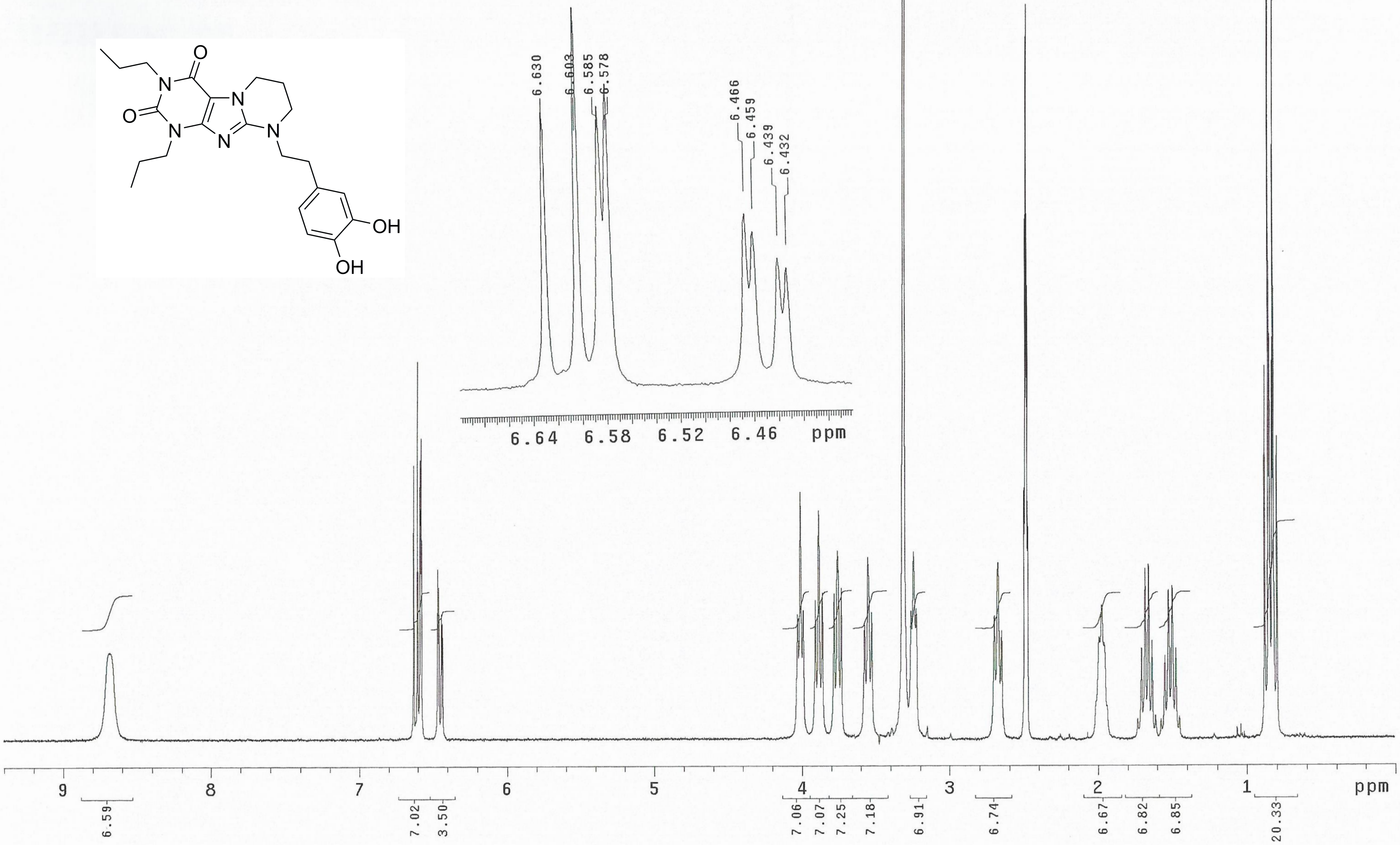

Compound 21
^{1}H NMR - zoom of the aliphatic region

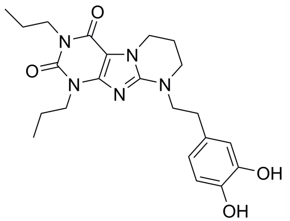

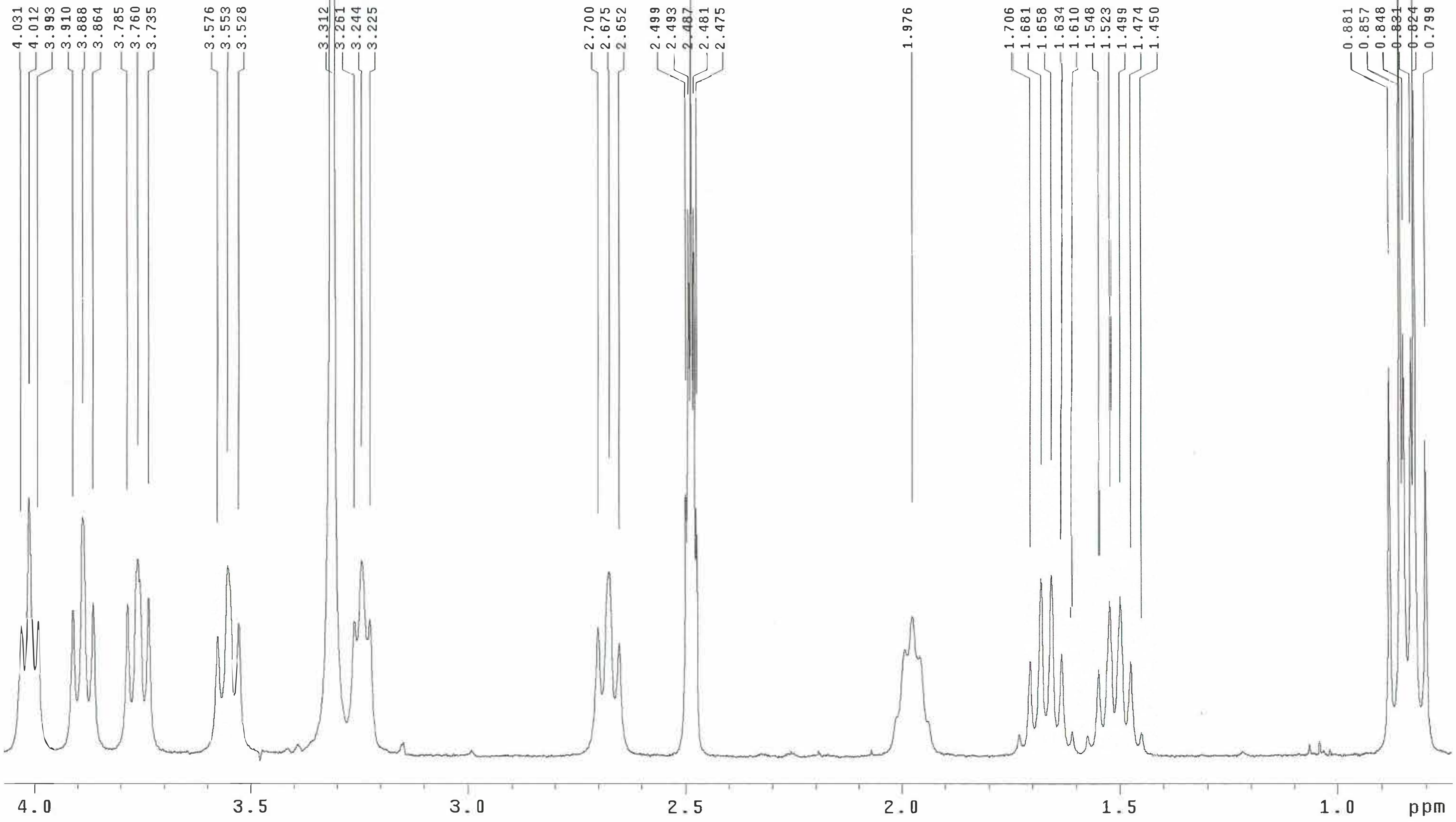

Table of peaks

INDEX	FREQUENCY	PPM	HEIGHT
1	2605.995	8.684	15.9
2	1989.488	6.630	49.9
3	1981.538	6.603	68.8
4	1975.896	6.585	50.8
5	1973.845	6.578	54.8
6	1940.250	6.466	30.9
7	1938.198	6.459	27.6
8	1932.300	6.439	22.7
	1930.248	6.432	21.0
10	1209.623	4.031	22.6
11	1203.981	4.012	44.9
12	1198.083	3.993	23.3
13	1173.464	3.910	25.1
14	1166.796	3.888	41.5
15	1159.615	3.864	26.5
16	1135.765	3.785	26.3
17	1128.328	3.760	34.2
18	1120.891	3.735	27.4
19	1073.192	3.576	20.6
20	1066.267	3.553	32.9
21	1058.574	3.528	22.9
22	993.948	3.312	930.2
23	978.561	3 261	23.2
24	973.432	3.244	34.0
25	967.791	3.225	23.6
26	810.330	2.700	22.2
27	802.637	2.675	31.9
28	795.712	2.652	19.5
29	749.808	2.499	45.6
30	748.013	2.493	95.2
31	746.218	2.487	133.3
32	744.422	2.481	95.4
33	742.627	2.475	43.1
34	598.246	1.994	18.4
35	592.860	1.976	24.2
36	587.731	1.959	17.3
37	519.259	1.730	3.6
38	511.822	1.706	16.3
39	504.385	1.681	30.8
40	497.461	1.658	31.6
41	490.280	1.634	17.7
42	483.100	1.610	4.1
43	471.816	1.572	3.5
44	464.379	1.548	14.9
45	456.942	1.523	26.9
46	449.761	1.499	27.7
47	442.324	1.474	16.3
48	435.143	1.450	4.0
49	264.348	0.881	67.6
50	257.167	0.857	153.1
51	254.602	0.848	73.5
52	249.473	0.831	72.8
53	247.165	0.824	140.1
54	239.728	0.799	54.8

Compound 21
^{13}C NMR

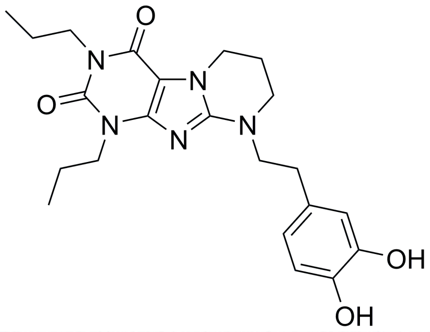

No.	(ppm)	(Hz)	Height
1	11.52	1159.5	0.1475
2	11.64	1171.3	0.1370
3	21.20	2133.7	0.0655
4	21.32	2145.5	0.1022
5	21.44	2158.0	0.1036
6	32.84	3304.6	0.0617
7	39.37	3961.9	0.1391
8	39.57	3982.4	0.4352
9	39.78	4003.7	0.8725
10	39.99	4024.2	1.0000
11	40.20	4045.5	0.8743
12	40.41	4066.8	0.4317
13	40.62	4087.3	0.1393
14	41.72	4198.8	0.0564
15	41.88	4215.0	0.0592
16	44.30	4458.5	0.0570
17	44.82	4510.6	0.0578
18	51.49	5181.1	0.0572
19	102.24	10289.1	0.0700
20	115.99	11672.0	0.0831
21	116.58	11732.1	0.0855
22	119.74	12049.8	0.0785
23	130.26	13108.3	0.0798
24	144.04	14494.8	0.0784
25	145.56	14648.1	0.1041
26	148.72	14965.8	0.0665
27	150.99	15194.7	0.0707
28	151.72	15268.0	0.0661
29	152.92	15389.1	0.0667

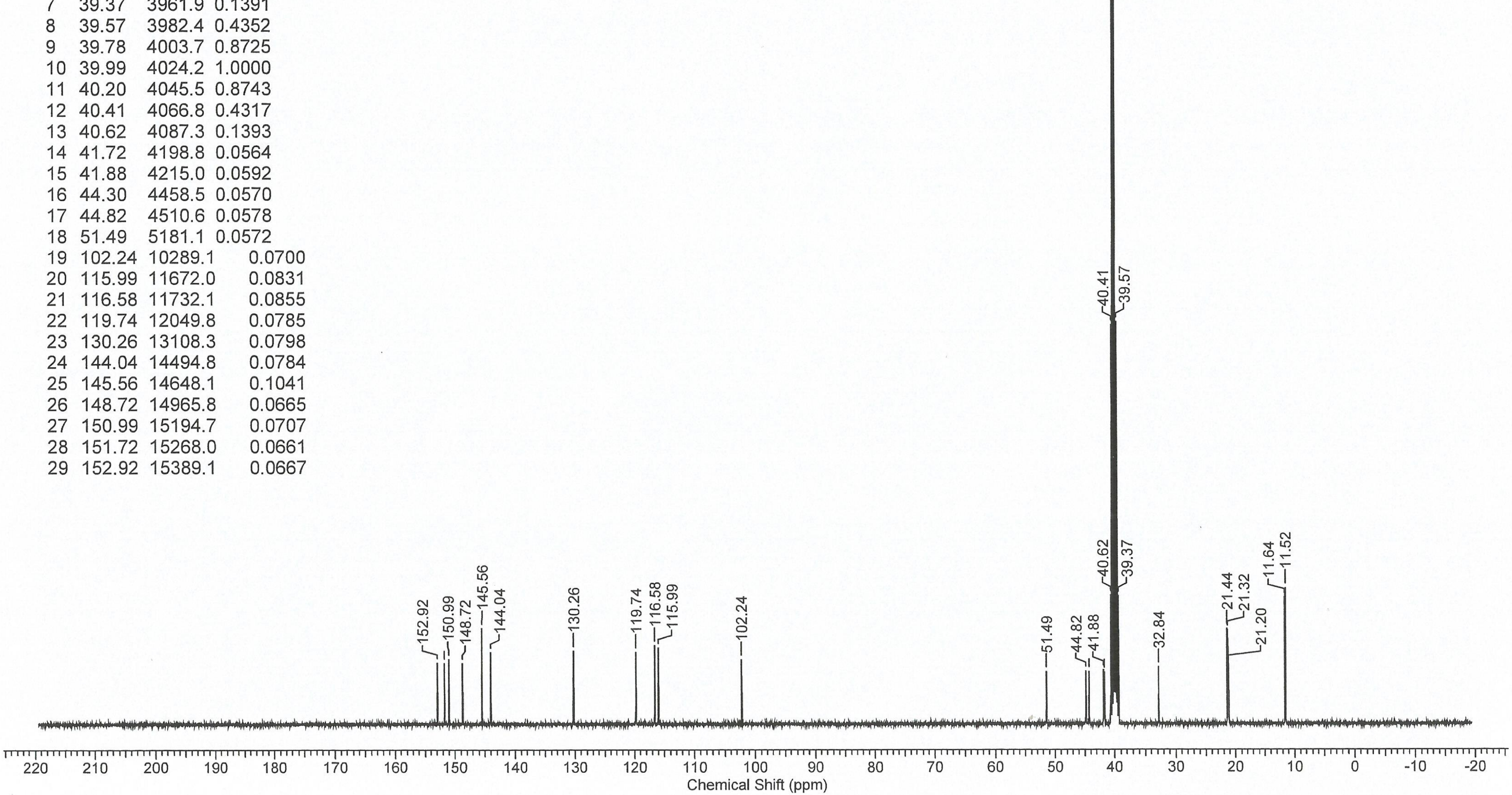

Compound 22
^{1}H NMR

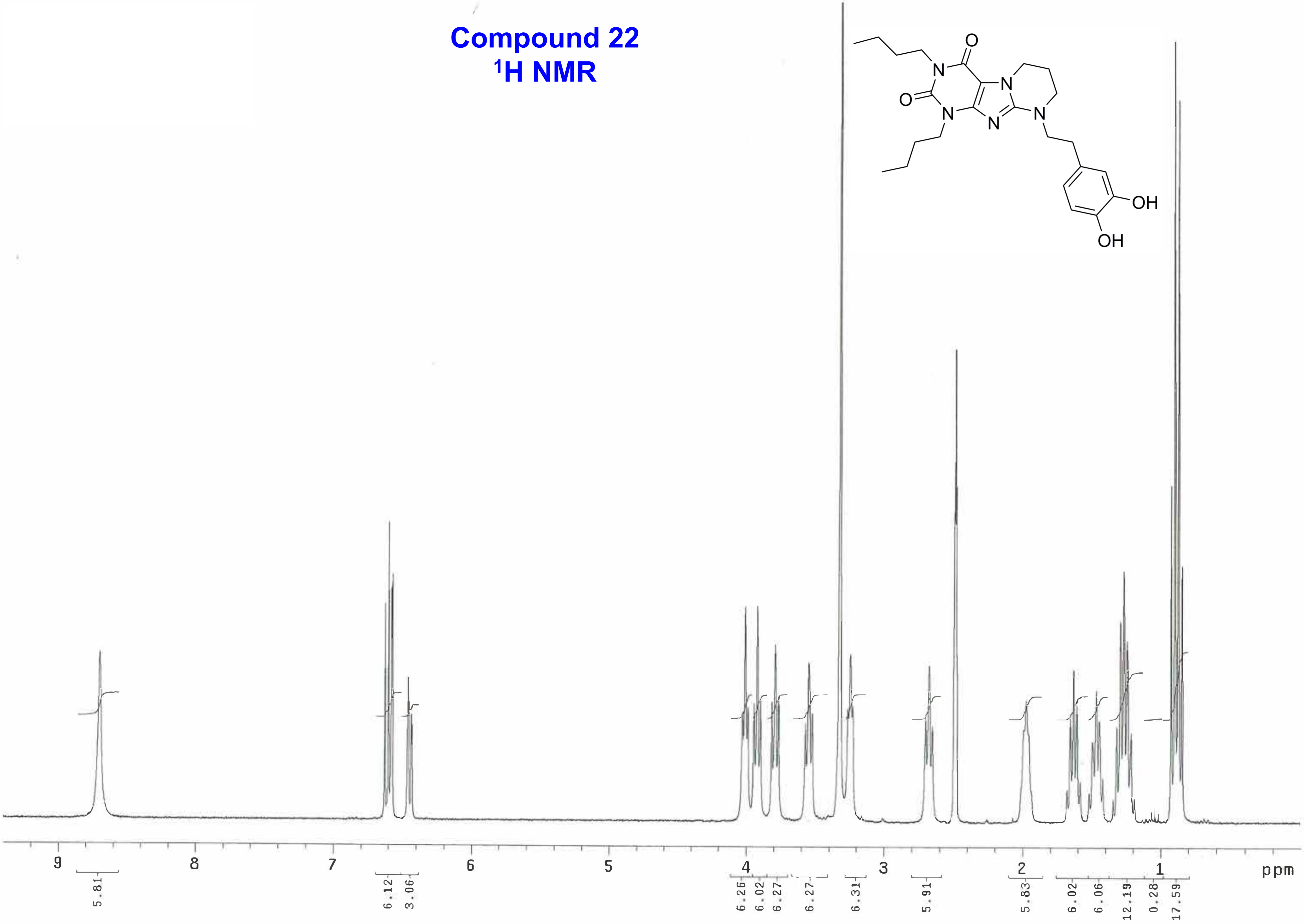

Compound 22
^{1}H NMR - zoom of the aliphatic region

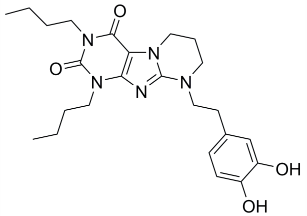

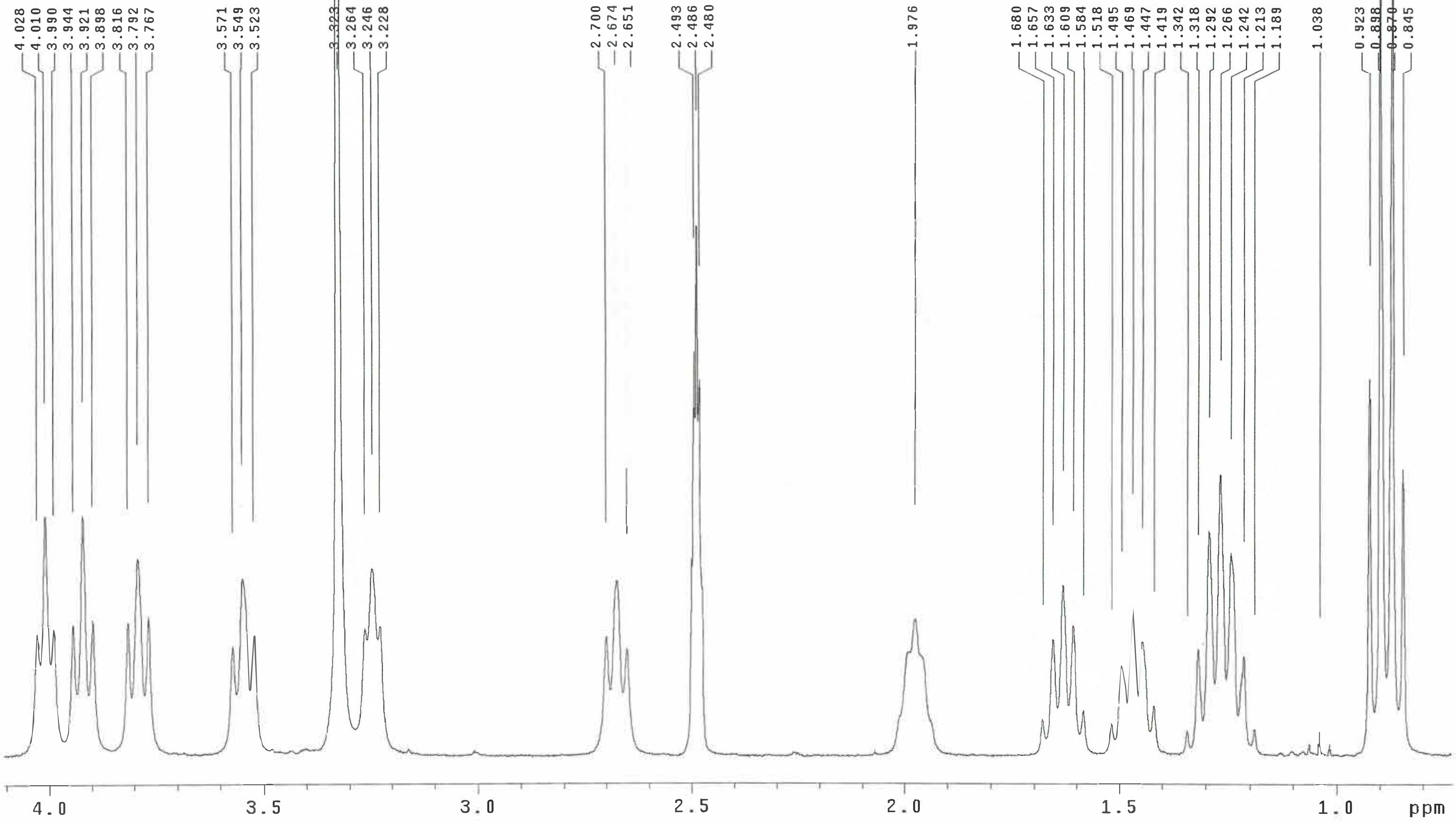

Compound 22
^{1}H NMR - zoom of the aromatic region

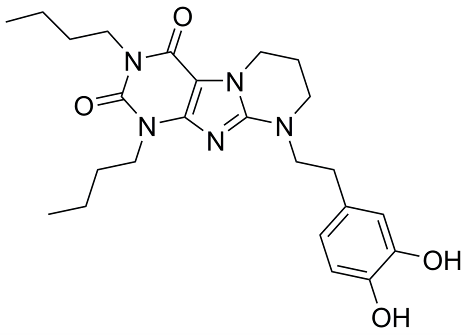

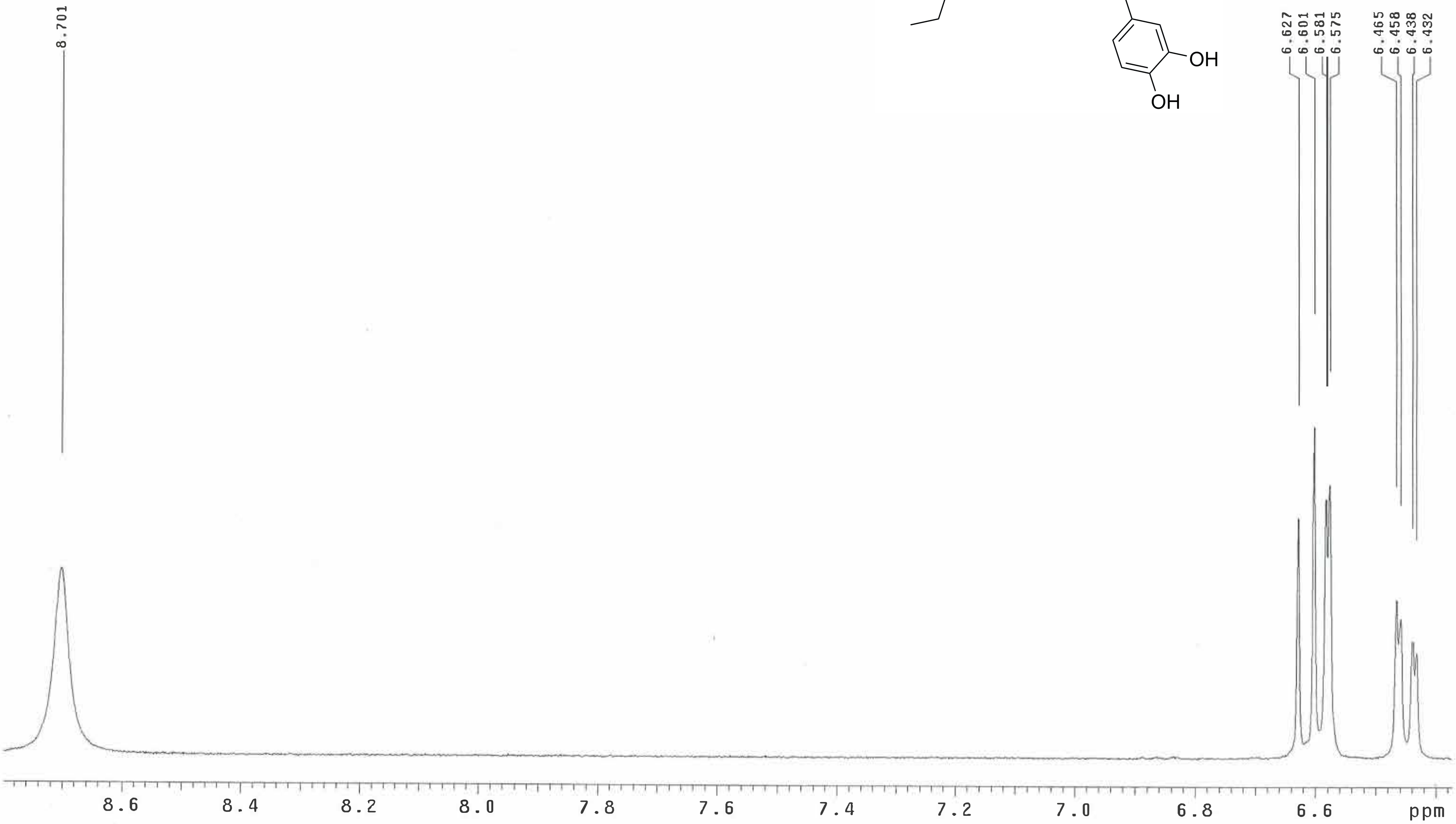

Table of peaks

INDEX	FREQUENCY	PPM	HEIGHT
1	2611.124	8.701	32.5
2	1988.719	6.627	42.0
3	1980.769	6.601	58.0
4	1974.871	6.581	45.4
5	1973.075	6.575	47.8
6	1939.993	6.465	27.7
7	1937.942	6.458	24.4
8	1931.787	6.438	20.4
9	1929.992	6.432	18.3
10	1208.854	4.028	21.3
11	1203.212	4.010	41.7
12	1197.313	3.990	22.1
13	1183.465	3.944	22.8
14	1176.541	3.921	41.9
15	1169.617	3.898	23.6
16	1144.998	3.816	23.2
17	1137.817	3.792	34.4
18	1130.380	3.767	24.3
19	1071.653	3.571	19.1
20	1064.985	3.549	30.8
21	1057.035	3.523	21.0
22	997.282	3.323	379.4
23	979.331	3.264	22.1
24	973.945	3.246	32.6
25	968.560	3.228	22.5
26	810.074	2.700	20.5
27	802.380	2.674	30.4
28	795.456	2.651	18.5
29	749.808	2.499	34.1
30	748.013	2.493	70.2
31	745.961	2.486	92.1
32	744.166	2.480	65.3
33	592.860	1.976	23.6
34	504.128	1.680	6.3
35	497.204	1.657	20.1
36	490.024	1.633	29.6
37	482.843	1.609	22.6
38	475.406	1.584	7.9
39	455.659	1.518	5.5

INDEX	FREQUENCY	PPM	HEIGHT
40	448.479	1.495	15.6
41	440.785	1.469	25.6
42	434.118	1.447	19.7
43	425.911	1.419	8.6
44	402.831	1.342	4.4
45	395.394	1.318	18.6
46	387.700	1.292	39.1
47	380.007	1.266	49.0
48	372.826	1.242	35.3
49	364.107	1.213	17.4
50	356.926	1.189	4.7
51	311.534	1.038	4.1
52	276.914	0.923	65.7
53	269.477	0.898	152.2
54	261.014	0.870	140.5
55	253.577	0.845	50.0

Compound 22
^{13}C NMR

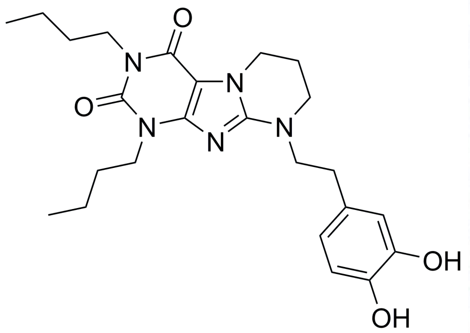

No.	(ppm)	(Hz)	Height
1	14.07	1416.3	0.1367
2	14.20	1428.8	0.1231
3	19.78	1990.7	0.1170
4	20.12	2024.4	0.1220
5	21.21	2134.5	0.0484
6	30.05	3023.6	0.0838
7	30.33	3052.2	0.0765
8	32.84	3304.6	0.0473
9	39.35	3960.4	0.1378
10	39.57	3981.7	0.4290
11	39.78	4002.9	0.8398
12	39.98	4023.5	1.0000
13	40.19	4044.8	0.8527
14	40.40	4065.3	0.4140
15	40.61	4086.6	0.1369
16	41.72	4198.1	0.0471
17	42.42	4268.5	0.0449
18	44.81	4509.1	0.0460
19	51.51	5183.3	0.0482
20	102.26	10290.6	0.0491
21	115.97	11670.5	0.0655
22	116.59	11732.8	0.0658
23	119.71	12046.8	0.0698
24	130.25	13107.6	0.0566
25	144.03	14494.1	0.0742
26	145.56	14648.1	0.0814
27	148.68	14962.1	0.0444
28	150.90	15185.9	0.0495
29	151.69	15265.1	0.0612
30	152.89	15386.1	0.0536

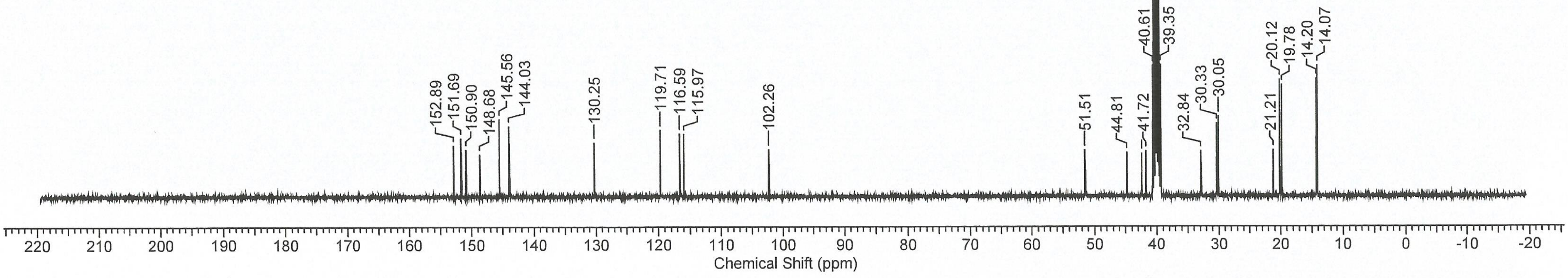

Compound 8a
^{1}H NMR

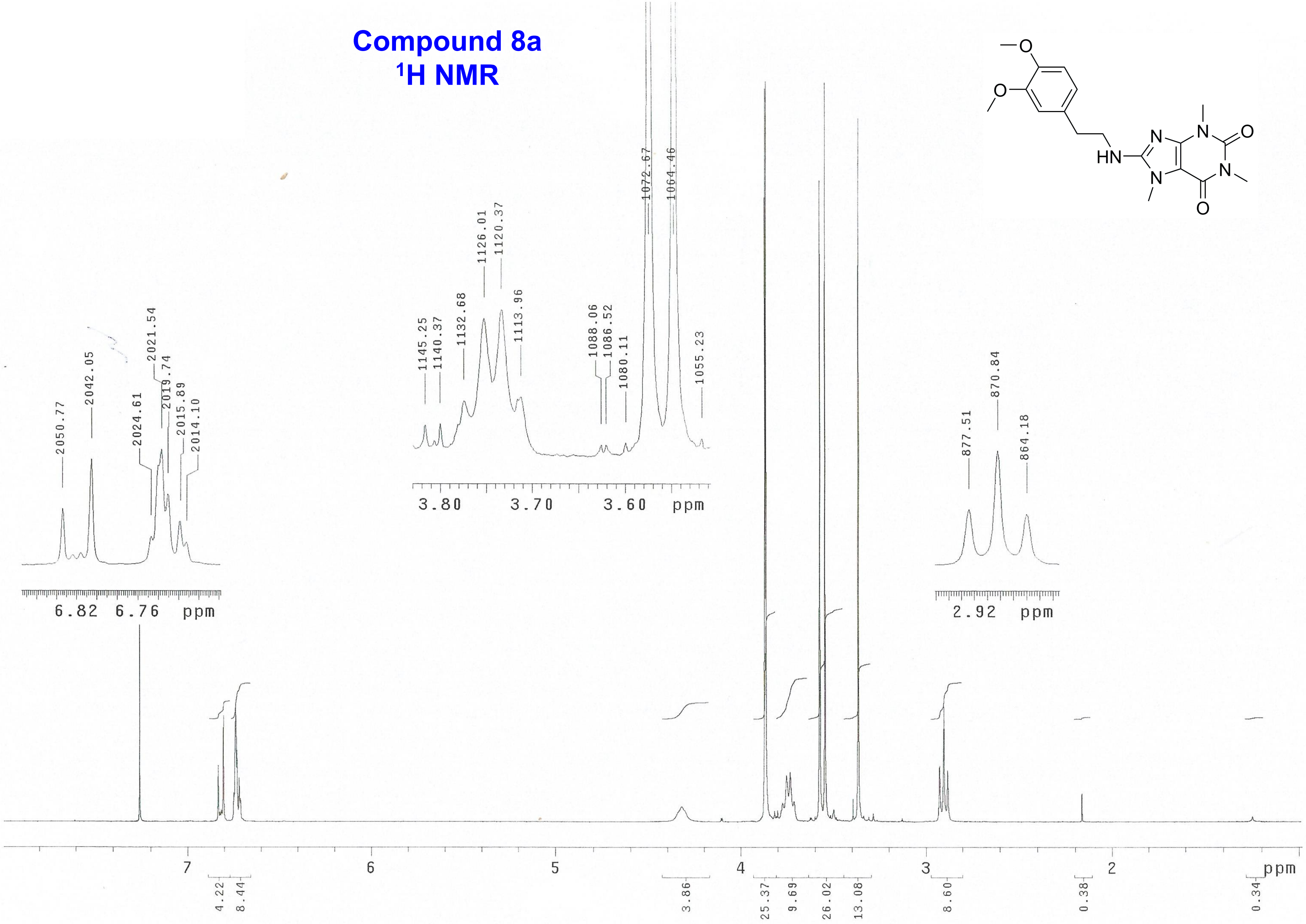

Table of peaks

INDEX	FREQUENCY	PPM	HEIGHT
1	2178.227	7.259	38.3
2	2050.771	6.834	11.0
3	2042.052	6.805	20.7
4	2024.614	6.747	5.6
5	2022.562	6.740	19.5
6	2021.536	6.737	22.6
7	2019.741	6.731	13.9
8	2015.894	6.718	8.5
9	2014.099	6.712	4.5
10	1296.808	4.322	2.9
11	1160.889	3.869	142.6
12	1159.350	3.863	144.0
13	1132.680	3.775	3.7
14	1126.012	3.752	9.1
15	1120.370	3.734	9.6
16	1113.959	3.712	4.0
17	1072.670	3.575	124.7
18	1064.464	3.547	143.8
19	1018.559	3.394	4.4
20	1010.096	3.366	136.9
21	877.512	2.924	10.9
22	870.844	2.902	22.3
23	864.176	2.880	10.0
24	649.014	2.163	5.5

Compound 8a
^{13}C NMR

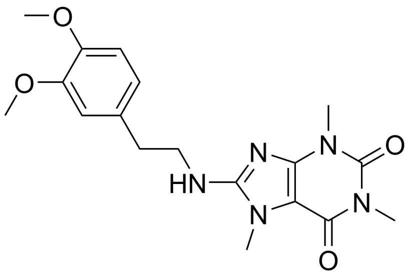

No.	(ppm)	(Hz)	Height
1	27.64	2781.5	0.3734
2	29.52	2970.8	0.2557
3	29.78	2996.5	0.3052
4	35.42	3564.3	0.3002
5	44.47	4474.6	0.2995
6	55.91	5626.4	0.4358
7	55.94	5629.3	0.4577
8	76.76	7724.5	0.9323
9	77.07	7756.0	1.0000
10	77.39	7788.3	0.9661
11	103.07	10372.0	0.1289
12	111.39	11209.8	0.3370
13	111.93	11263.3	0.3056
14	120.74	12150.3	0.3930
15	130.93	13175.8	0.1848
16	147.89	14882.2	0.1723
17	148.35	14929.1	0.0768
18	149.18	15012.7	0.1816
19	151.69	15265.1	0.1378
20	153.08	15404.5	0.1191
21	154.17	15514.5	0.1182

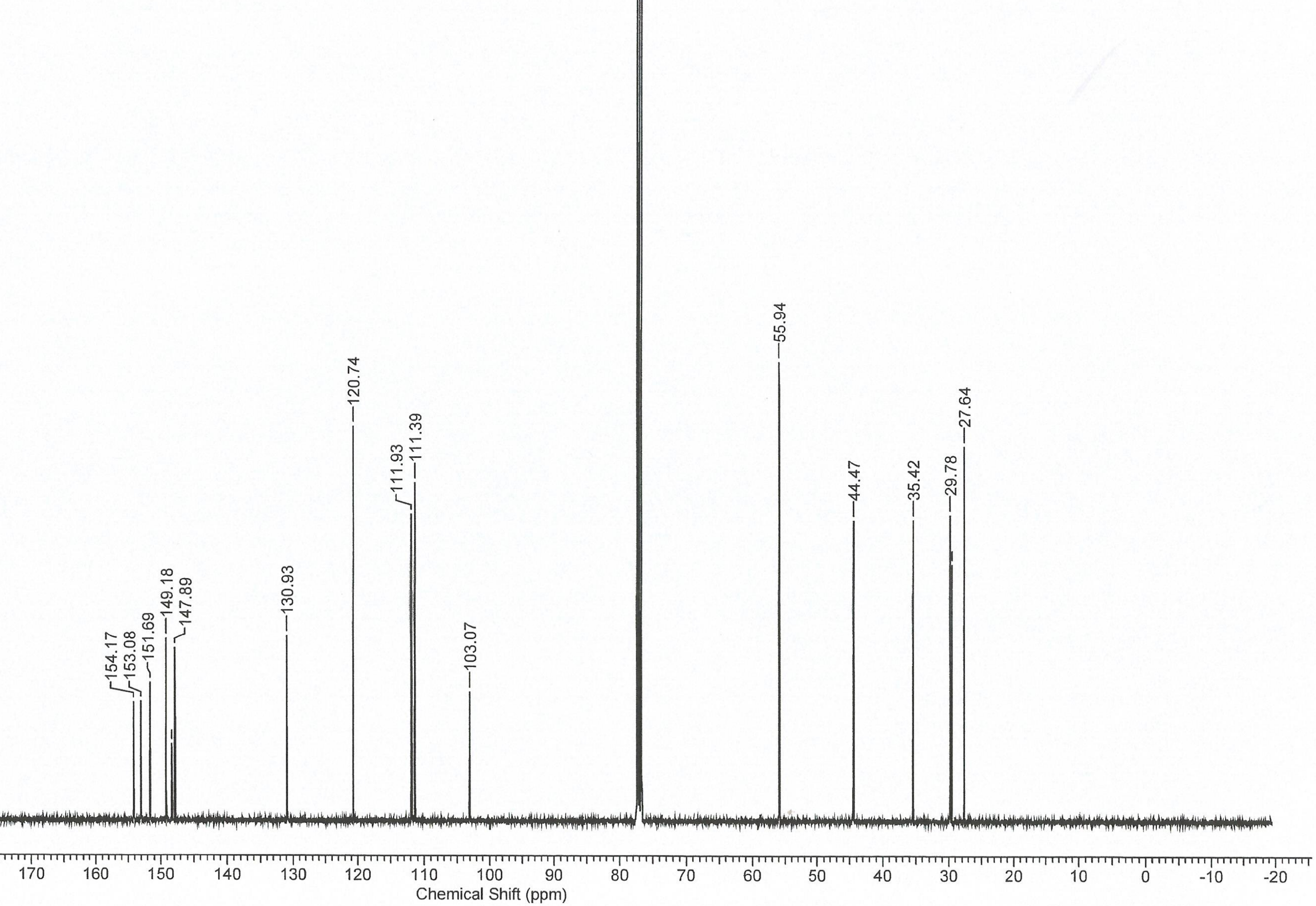

Compound 8b
^{1}H NMR

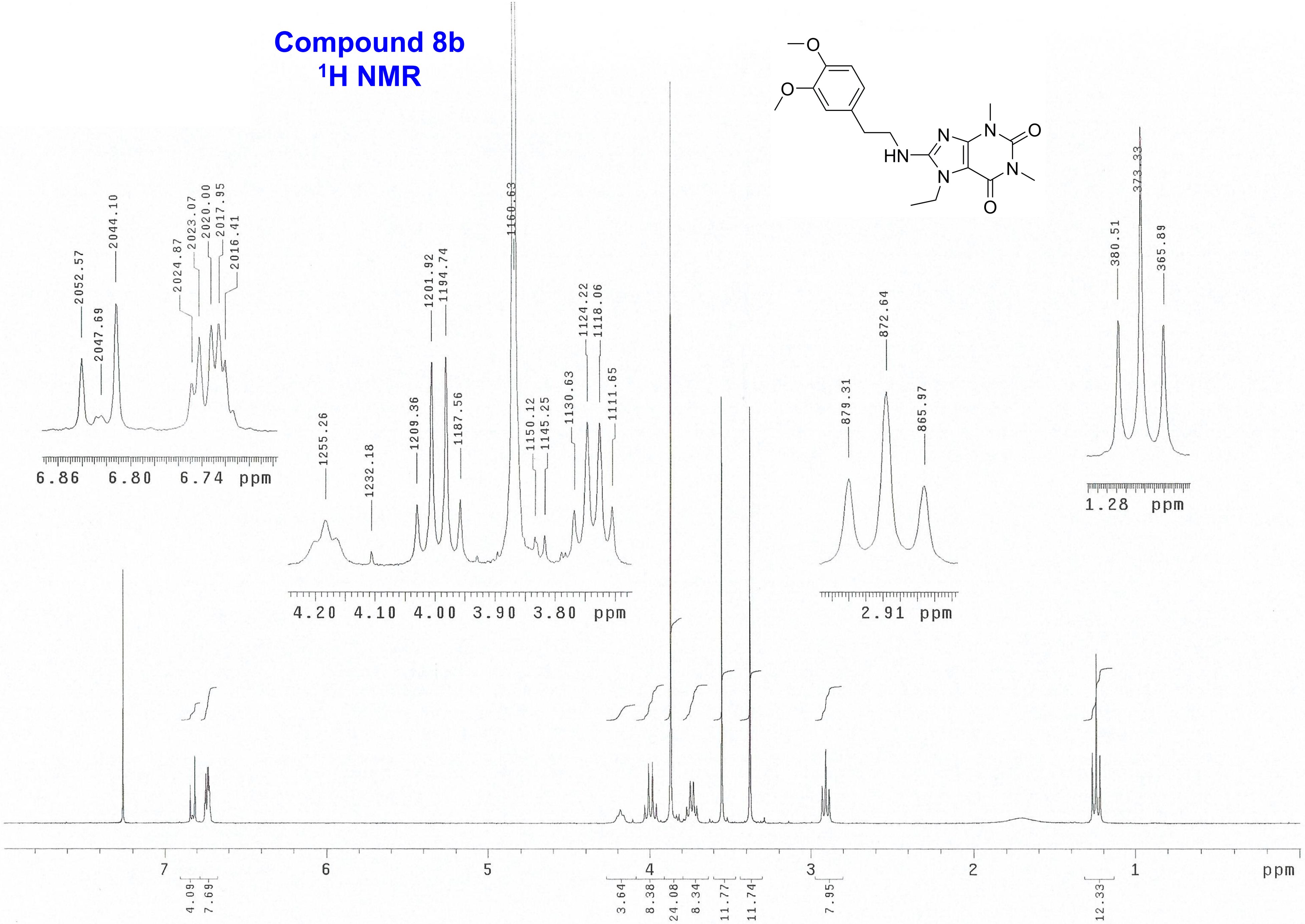

Table of peaks

INDEX	FREQUENCY	PPM	HEIGHT
1	2178.227	7.259	49.3
2	2052.567	6.840	7.4
3	2044.104	6.812	13.1
4	2024.870	6.748	4.9
5	2023.075	6.742	9.6
6	2019.997	6.732	10.8
7	2017.946	6.725	11.0
8	2016.407	6.720	7.3
9	1255.263	4.183	2.6
10	1209.358	4.030	3.5
11	1201.921	4.005	11.6
12	1194.741	3.981	11.9
13	1187.560	3.957	3.8
14	1160.633	3.868	144.0
15	1130.628	3.768	3.2
16	1124.217	3.746	8.2
17	1118.062	3.726	8.2
18	1111.651	3.705	3.4
19	1064.977	3.549	83.0
20	1012.917	3.376	81.0
21	879.307	2.930	7.3
22	872.639	2.908	14.4
23	865.971	2.886	6.7
24	380.511	1.268	13.7
25	373.330	1.244	32.9
26	365.893	1.219	13.2

Compound 8b
^{13}C NMR

No.	(ppm)	(Hz)	Height
1	14.68	1477.2	0.4127
2	27.65	2783.0	0.3992
3	29.75	2993.5	0.3359
4	35.37	3559.9	0.3447
5	38.16	3840.1	0.2556
6	44.44	4472.4	0.3151
7	55.89	5624.2	0.4608
8	55.95	5630.8	0.4700
9	76.75	7723.7	0.9464
10	77.07	7756.0	1.0000
11	77.39	7788.3	0.9381
12	102.43	10308.2	0.1377
13	111.40	11210.5	0.3414
14	111.94	11264.8	0.2951
15	120.77	12153.9	0.4298
16	131.02	13185.4	0.1988
17	147.89	14882.2	0.2007
18	148.59	14952.6	0.0929
19	149.19	15013.5	0.2200
20	151.76	15272.4	0.1585
21	152.22	15317.9	0.1510
22	153.90	15487.4	0.1493

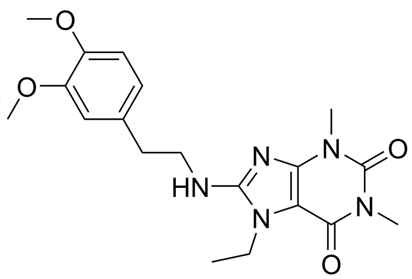

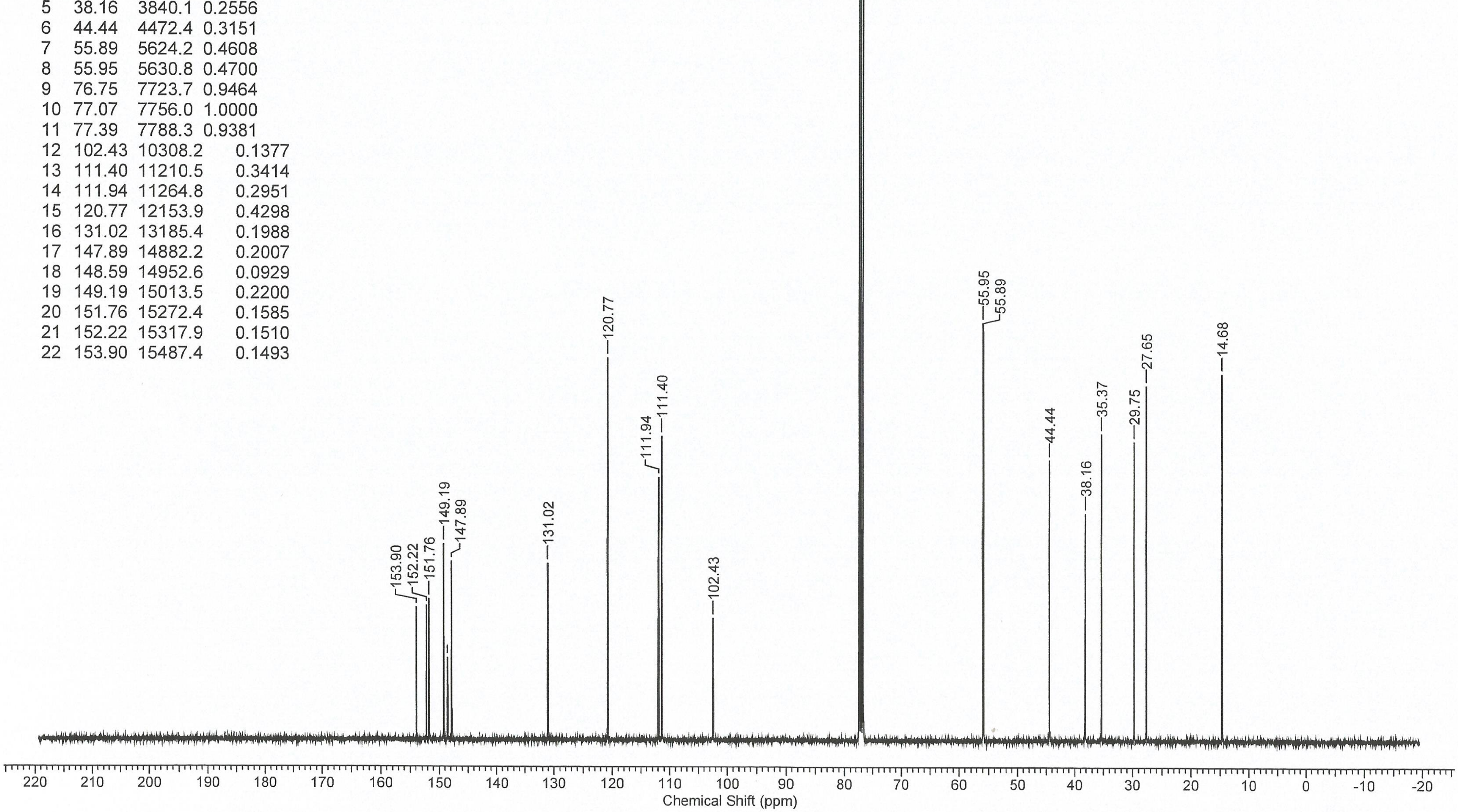

Compound 8c
^{1}H NMR - zoom of the aromatic region

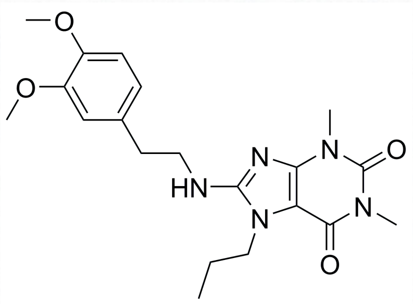

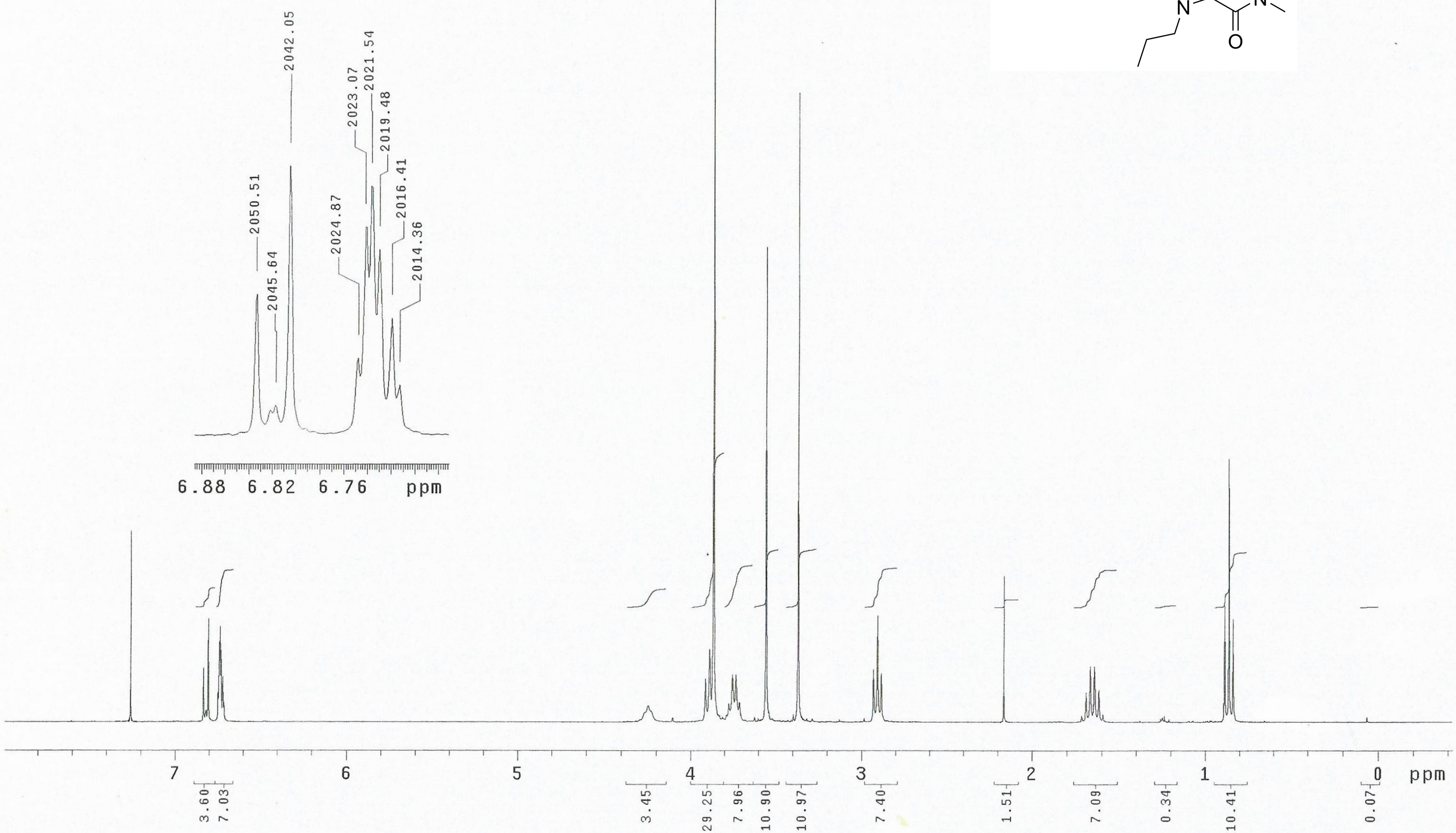

Compound 8c
^{1}H NMR - zoom of the aliphatic region

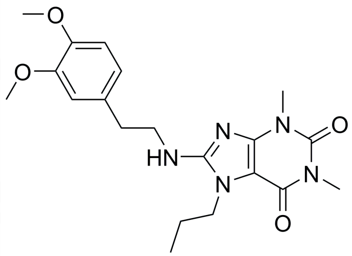

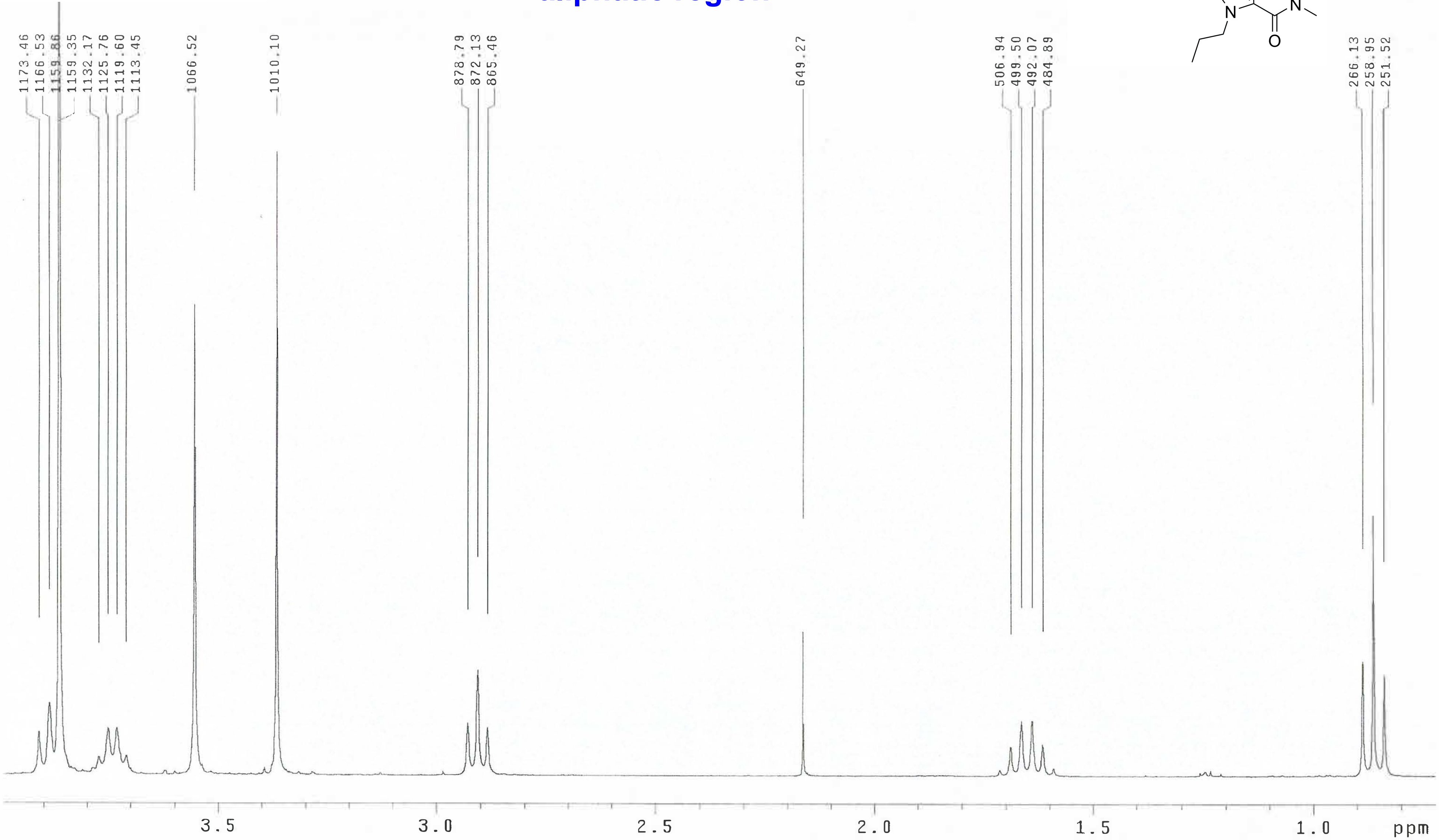

Table of peaks

INDEX	FREQUENCY	PPM	HEIGHT
1	2178.227	7.259	33.3
2	2050.515	6.833	9.4
3	2042.052	6.805	18.0
4	2024.870	6.748	5.2
5	2023.075	6.742	13.9
6	2021.536	6.737	16.6
7	2019.485	6.730	12.4
8	2016.407	6.720	7.8
9	2014.356	6.713	3.4
10	1274.240	4.246	2.8
11	1173.455	3.910	7.6
12	1166.531	3.887	12.7
13	1159.863	3.865	139.0
14	1159.350	3.863	144.0
15	1132.167	3.773	3.2
16	1125.755	3.752	8.3
17	1119.601	3.731	8.3
18	1113.446	3.711	3.4
19	1066.515	3.554	83.0
20	1010.096	3.366	109.7
21	878.794	2.929	9.2
22	872.126	2.906	18.5
23	865.458	2.884	8.4
24	649.271	2.164	25.4
25	506.941	1.689	5.1
26	499.504	1.665	9.7
27	492.067	1.640	9.8
28	484.886	1.616	5.6
29	266.134	0.887	20.2
30	258.954	0.863	45.9
31	251.517	0.838	17.9

Compound 8c
^{13}C NMR

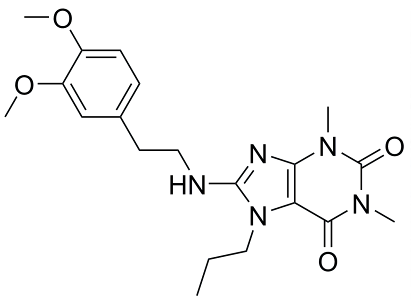

No.	(ppm)	(Hz)	Height
1	10.94	1100.8	0.2496
2	22.75	2289.3	0.1854
3	27.68	2785.2	0.1984
4	29.81	2999.4	0.1848
5	35.33	3555.5	0.1794
6	44.43	4471.0	0.1762
7	44.78	4506.2	0.1383
8	55.89	5624.2	0.1960
9	55.97	5632.3	0.2336
10	76.73	7721.5	0.9990
11	77.04	7753.1	0.9540
12	77.36	7785.4	1.0000
13	102.91	10356.6	0.0893
14	111.39	11209.1	0.1798
15	111.90	11261.1	0.1730
16	120.77	12153.9	0.1823
17	130.93	13175.8	0.1278
18	147.94	14887.3	0.1138
19	148.31	14924.7	0.0376
20	149.24	15018.6	0.1230
21	151.74	15270.2	0.0885
22	152.42	15338.5	0.0962
23	153.89	15486.7	0.1070

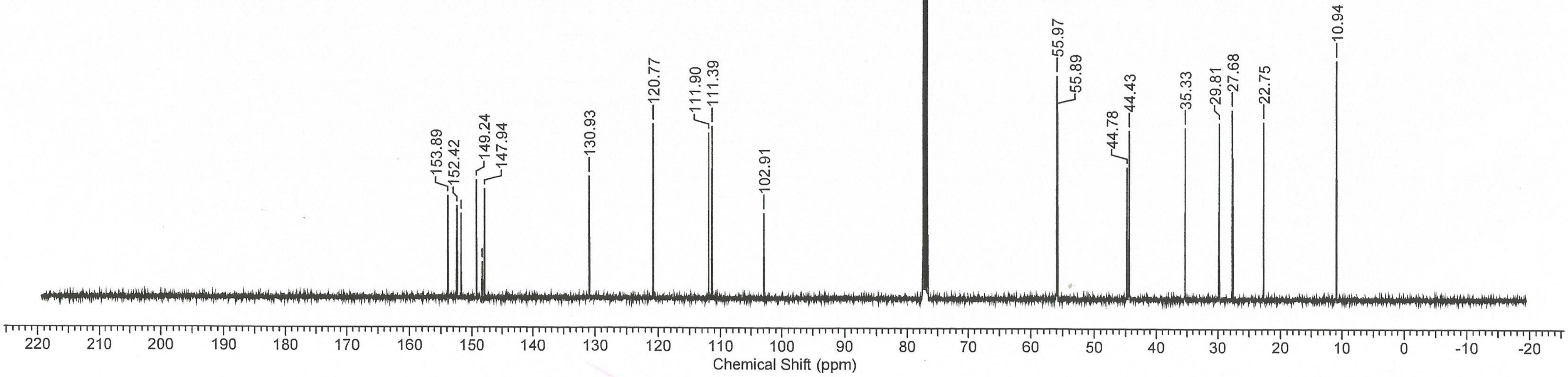

Compound 8d
^{1}H NMR

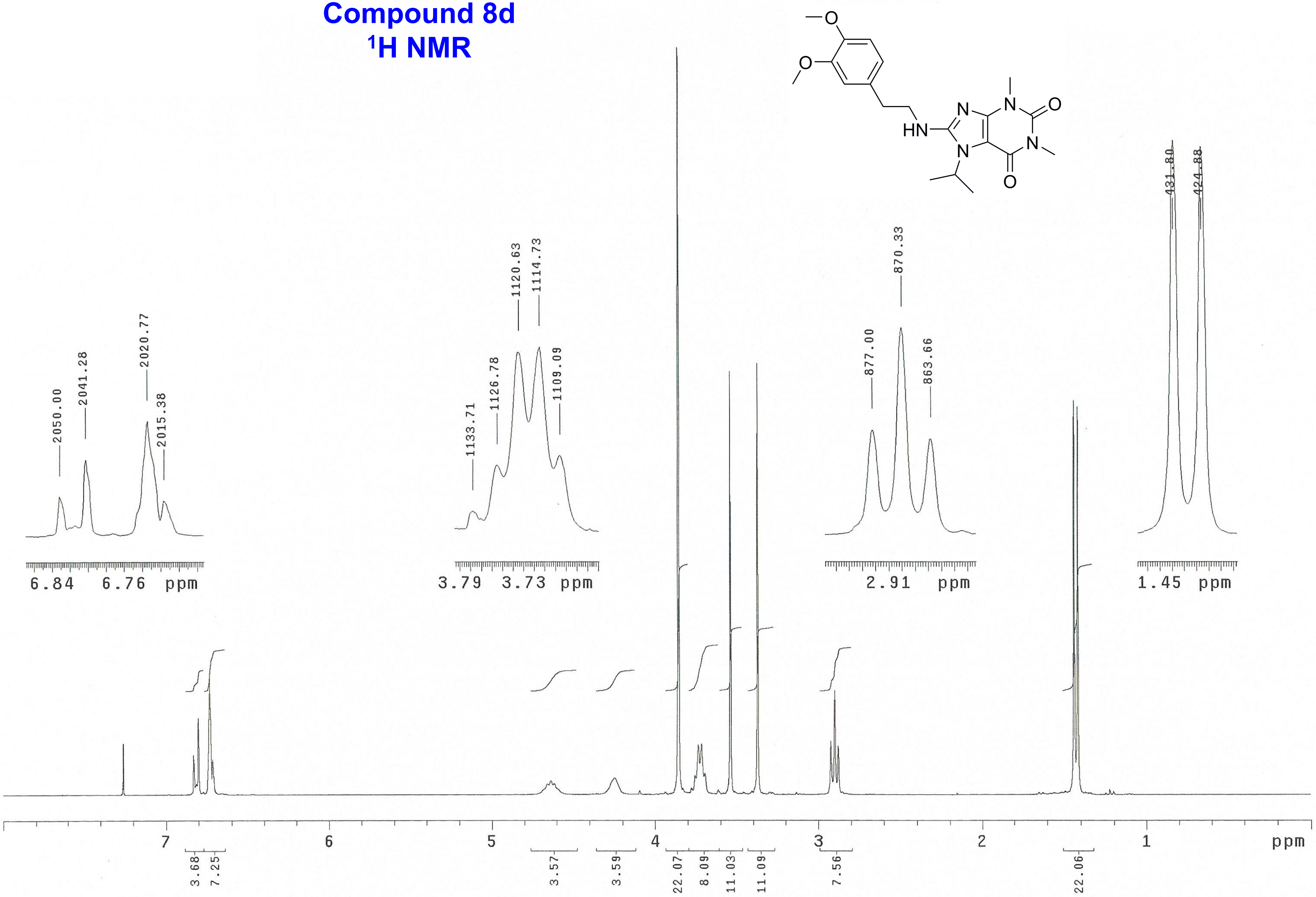

Table of peaks

INDEX	FREQUENCY	PPM	HEIGHT
1	2177.458	7.256	9.9
2	2050.002	6.832	7.7
3	2041.283	6.802	14.8
4	2020.767	6.734	22.3
5	2015.381	6.716	6.9
6	1391.181	4.636	2.7
7	1273.984	4.245	3.3
8	1157.555	3.858	144.0
9	1126.781	3.755	3.7
10	1120.626	3.734	9.5
11	1114.728	3.715	9.8
12	1109.086	3.696	4.2
13	1062.412	3.540	81.8
14	1061.386	3.537	57.8
15	1012.404	3.374	83.3
16	876.999	2.923	10.3
17	870.331	2.900	20.1
18	863.663	2.878	9.4
19	431.801	1.439	76.1
20	424.877	1.416	74.9

Compound 8d
^{13}C NMR

No.	(ppm)	(Hz)	Height
1	21.08	2121.3	0.4448
2	27.99	2816.7	0.2303
3	29.78	2996.5	0.1862
4	35.28	3550.3	0.1955
5	44.73	4501.1	0.2072
6	47.13	4742.4	0.1559
7	55.90	5624.9	0.2649
8	55.98	5633.0	0.2926
9	76.74	7722.3	0.9200
10	77.05	7753.8	1.0000
11	77.37	7786.1	0.9558
12	102.68	10333.1	0.0838
13	111.41	11211.3	0.2255
14	111.93	11263.3	0.2266
15	120.82	12158.3	0.2623
16	131.05	13187.6	0.1561
17	147.94	14887.3	0.1359
18	149.01	14995.1	0.0450
19	149.25	15019.3	0.1363
20	151.71	15267.3	0.0940
21	152.13	15309.1	0.1146
22	153.69	15466.1	0.0959

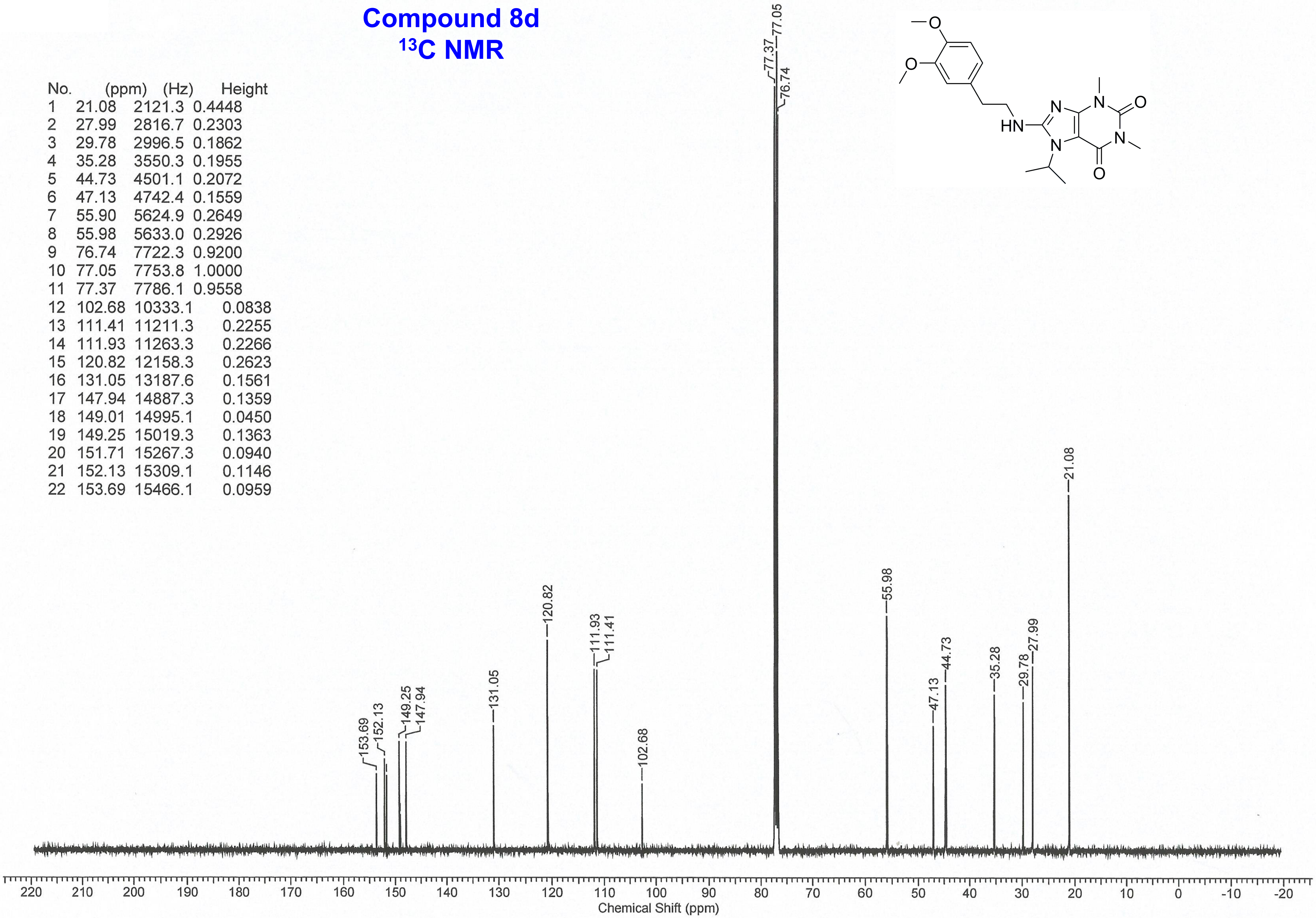

Compound 8e
1H NMR - zoom of the aromatic region

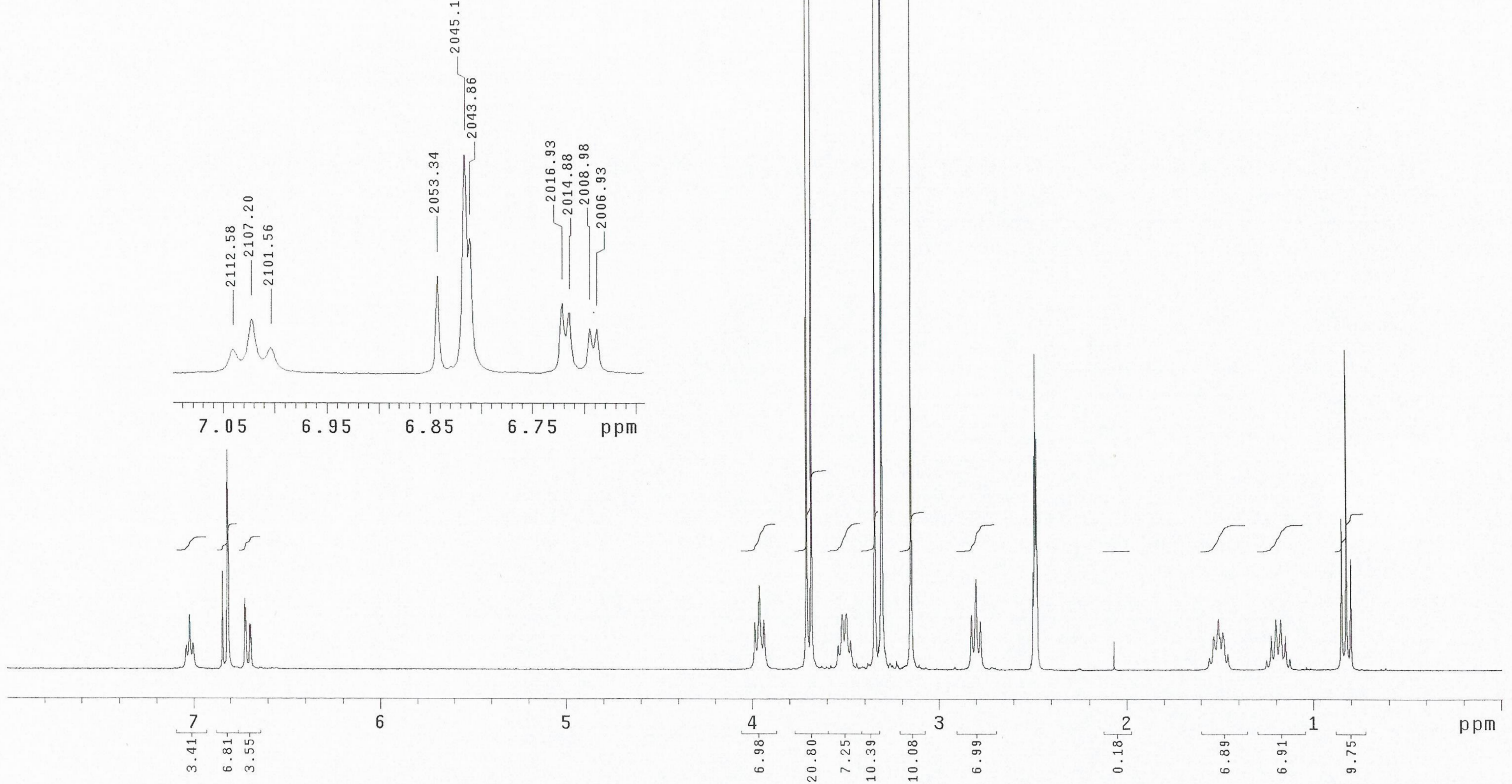

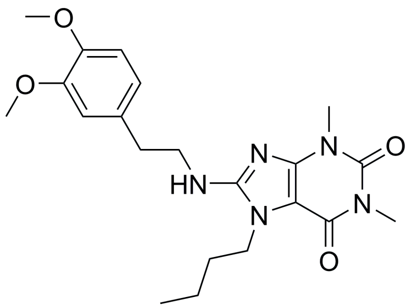

Compound 8e
^{1}H NMR - zoom of the aliphatic region

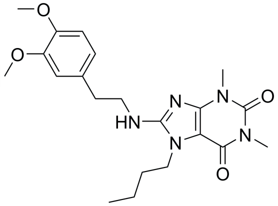

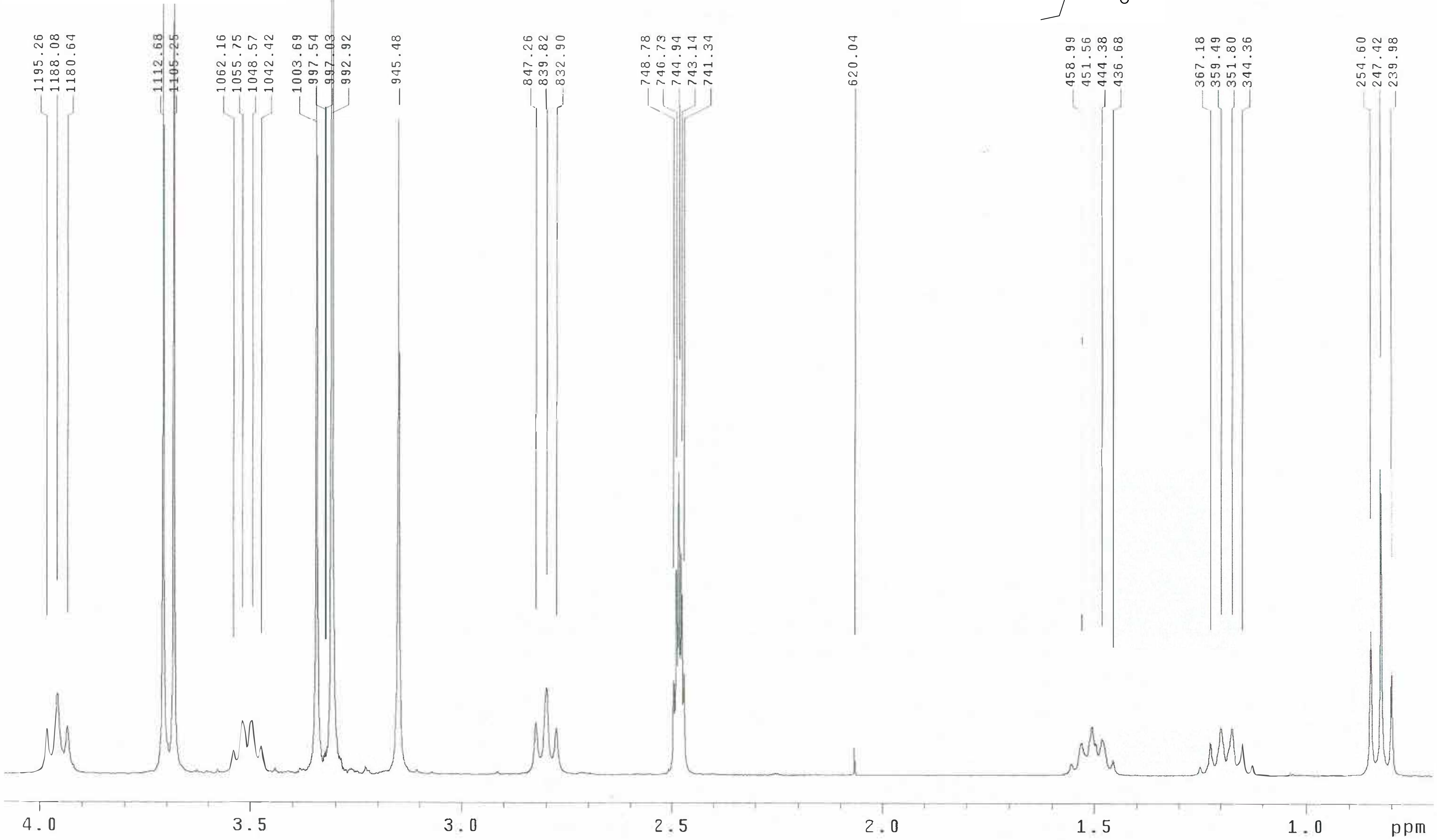

Table of peaks

INDEX	FREQUENCY	PPM	HEIGHT
1	2112.584	7.040	4.0
2	2107.199	7.022	9.1
3	2101.557	7.003	4.3
4	2053.344	6.843	16.5
5	2045.138	6.815	37.0
6	2043.856	6.811	22.9
7	2016.928	6.721	11.9
8	2014.877	6.714	10.3
9	2008.979	6.695	7.6
10	2006.927	6.688	7.5
11	1195.262	3.983	7.9
12	1188.081	3.959	14.3
13	1180.644	3.934	8.4
14	1112.685	3.708	147.7
15	1105.248	3.683	144.3
16	1062.164	3.540	4.2
17	1055.753	3.518	9.4
18	1048.572	3.494	9.5
19	1042.418	3.474	4.9
20	1003.694	3.345	121.2
21	999.077	3.329	2.6
22	998.565	3.328	2.8
23	998.052	3.326	3.3
24	997.539	3.324	3.8
25	997.026	3.323	3.9
26	996.513	3.321	4.3
27	996.000	3.319	4.9
28	992.923	3.309	565.4
29	988.307	3.293	3.8
30	987.794	3.292	3.3
31	987.281	3.290	3.2
32	986.768	3.288	3.0
33	986.255	3.287	2.8
34	945.479	3.151	115.3
35	847.259	2.823	9.2
36	839.822	2.799	15.4
37	832.898	2.776	8.2
38	748.782	2.495	16.5
39	746.730	2.488	36.2
40	744.935	2.482	53.4
41	743.140	2.476	39.0
42	741.345	2.470	17.8
43	620.044	2.066	4.8
44	458.993	1.530	5.7
45	451.556	1.505	8.6
46	448.735	1.495	5.6
47	444.376	1.481	6.5
48	436.682	1.455	2.7
49	367.184	1.224	5.8
50	359.491	1.198	8.6
51	351.797	1.172	8.6
52	344.360	1.148	5.7
53	254.602	0.848	25.6
54	247.422	0.825	54.1
55	239.985	0.800	18.7

Compound 8e
^{13}C NMR

No.	(ppm)	(Hz)	Height
1	14.05	1060.3	0.2041
2	19.49	1471.1	0.2037
3	27.61	2083.6	0.0808
4	27.63	2084.8	0.0777
5	29.64	2236.8	0.0568
6	29.66	2238.5	0.0582
7	31.63	2386.6	0.1785
8	35.24	2659.2	0.1295
9	39.15	2954.4	0.1349
10	39.43	2975.4	0.4208
11	39.71	2996.4	0.8468
12	39.99	3017.4	1.0000
13	40.26	3038.5	0.8577
14	40.54	3059.5	0.4316
15	40.81	3079.9	0.1436
16	42.59	3214.3	0.0874
17	44.59	3365.2	0.0893
18	55.80	4210.5	0.0767
19	55.82	4212.2	0.0763
20	55.96	4223.3	0.0739
21	55.99	4225.5	0.0687
22	101.83	7684.2	0.1134
23	112.35	8478.2	0.1745
24	113.11	8535.7	0.1743
25	121.07	9136.1	0.1815
26	132.37	9989.2	0.1754
27	147.71	11146.3	0.1301
28	148.98	11242.5	0.1009
29	149.08	11250.3	0.1501
30	151.42	11426.6	0.1153
31	153.02	11547.7	0.1298
32	153.69	11598.0	0.1854

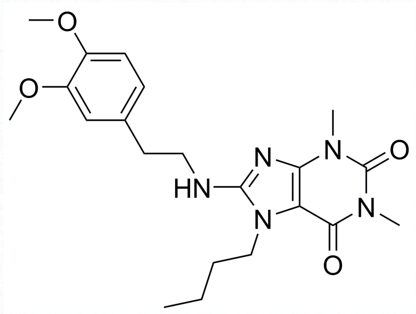

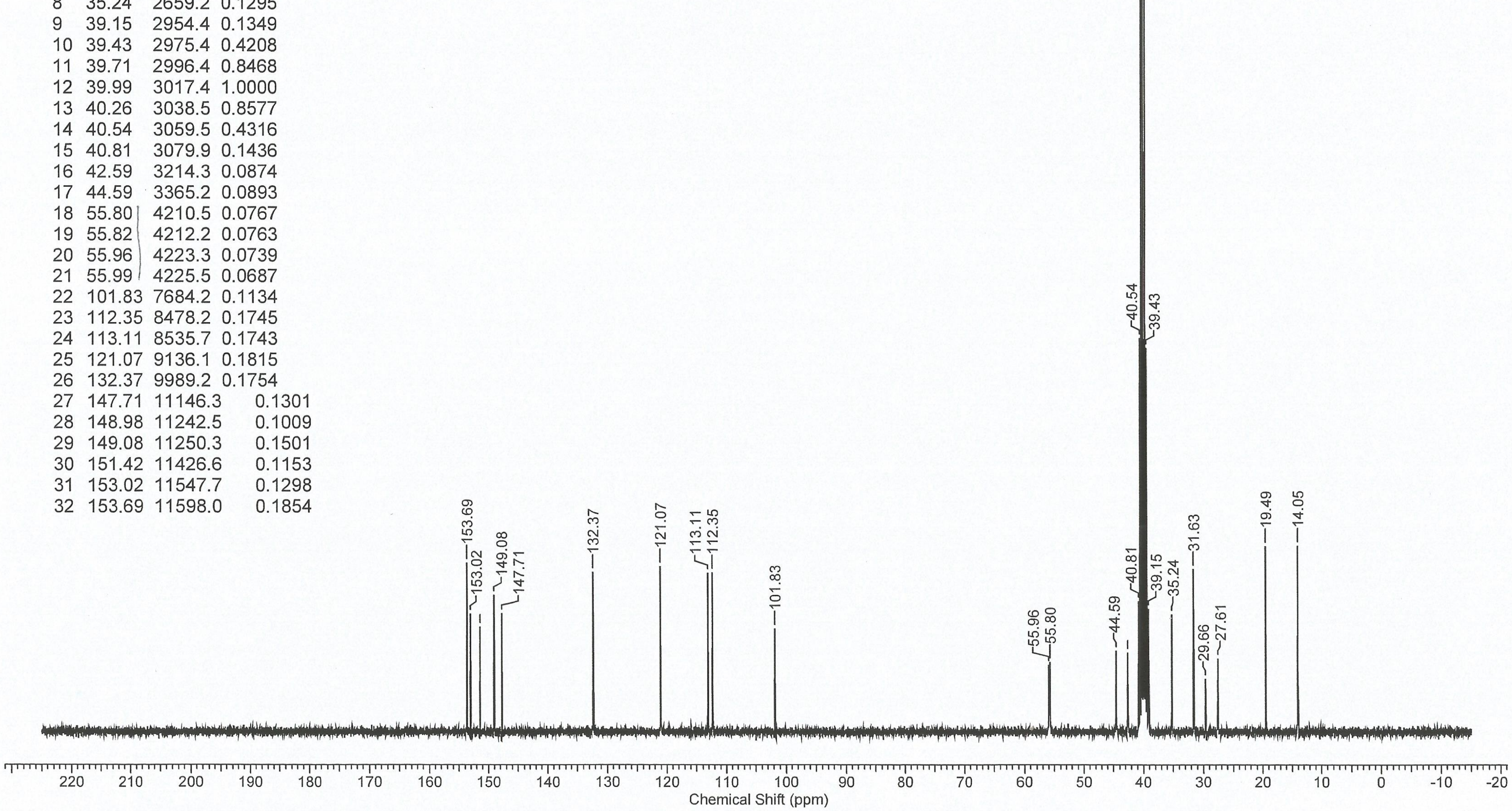

[illegible]

Compound 8f

^{1}H NMR - zoom of the aromatic region

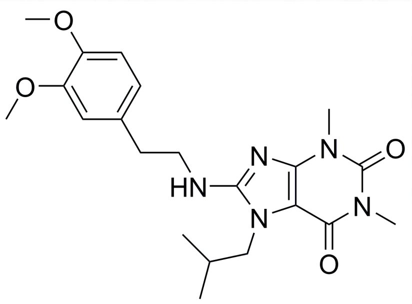

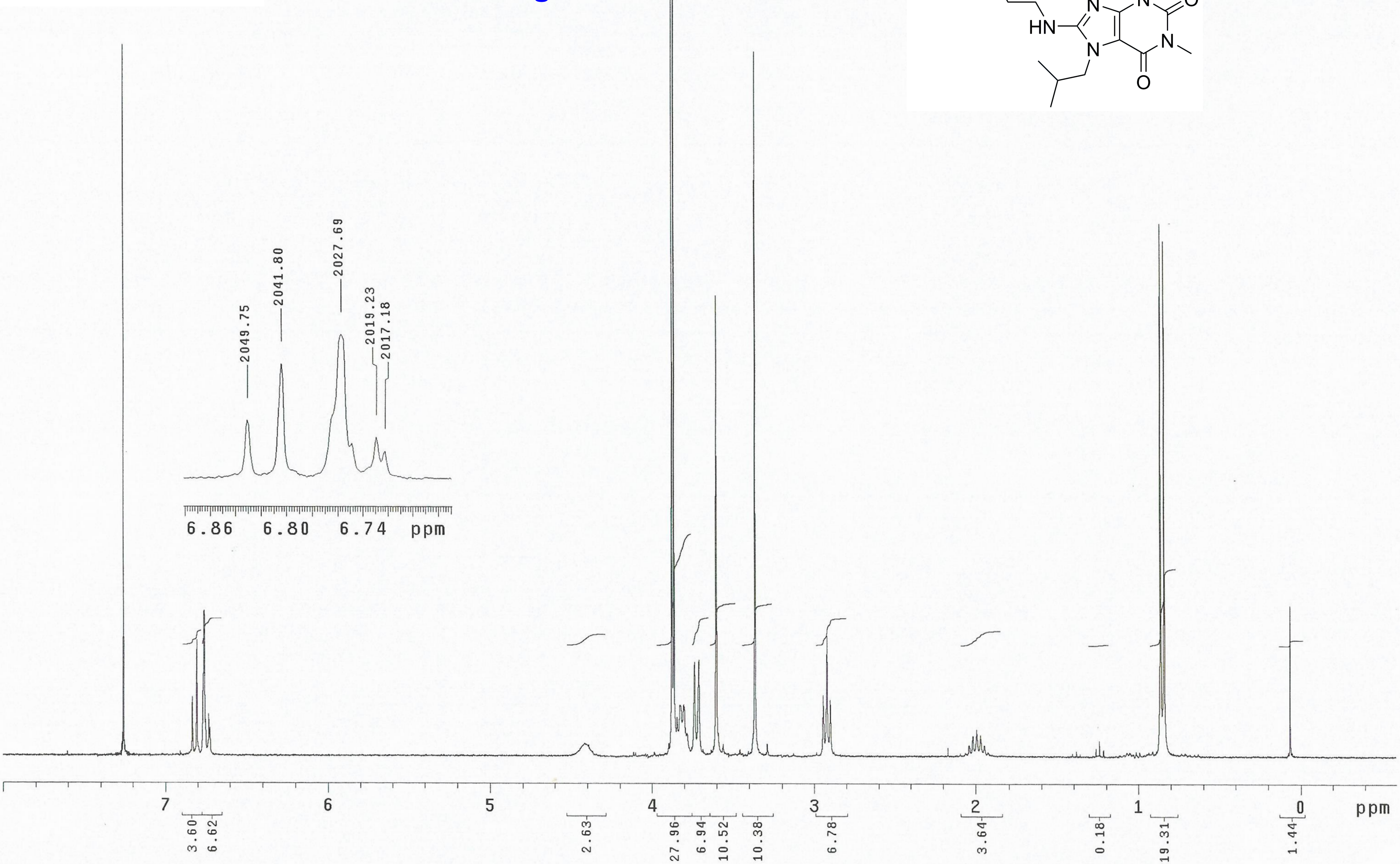

Compound 8f
1H NMR - zoom of the aliphatic region

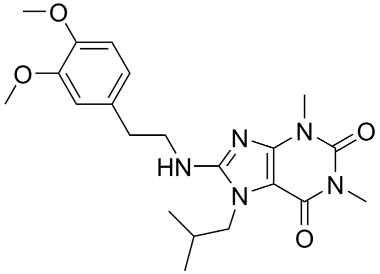

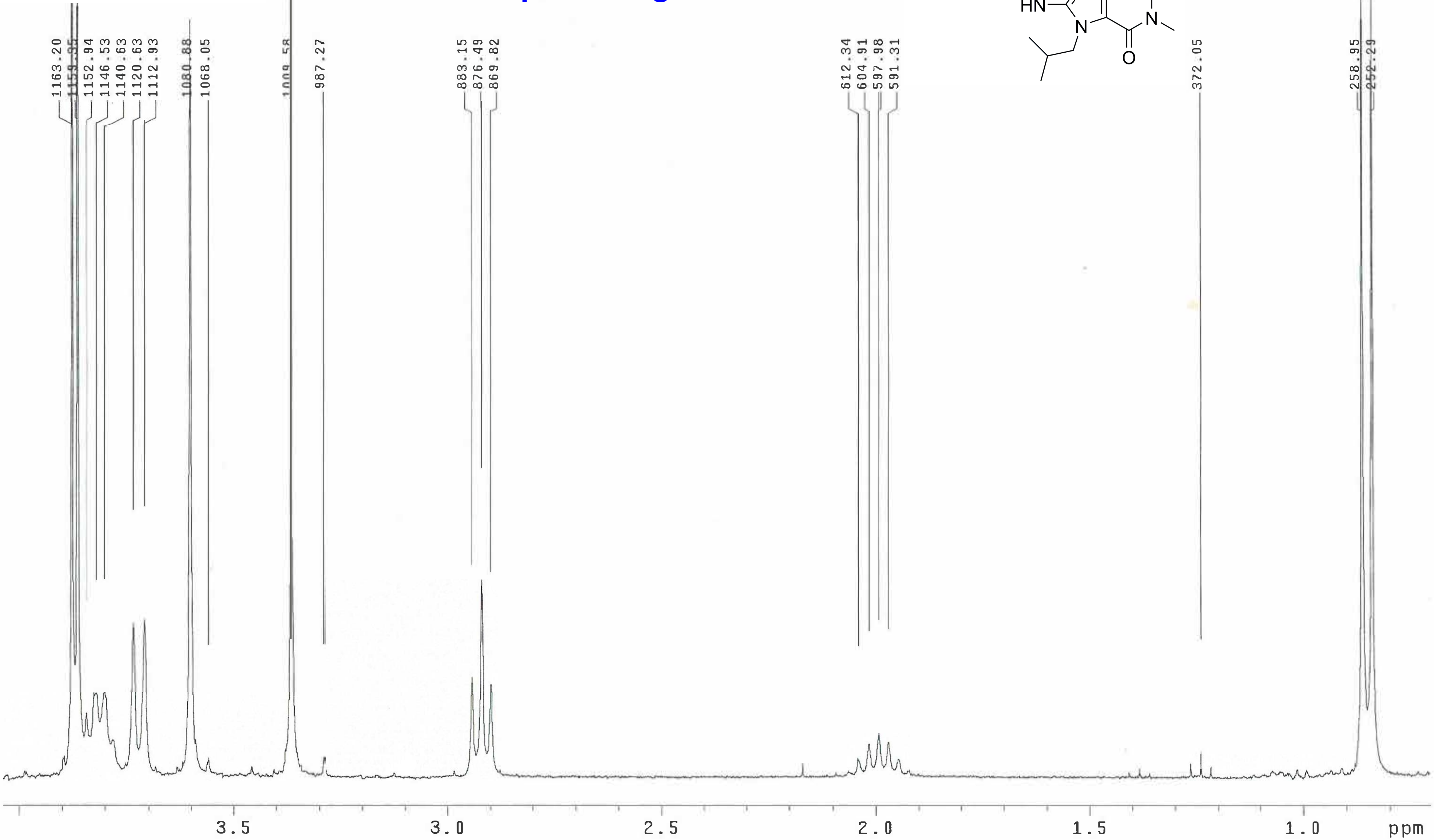

Table of peaks

INDEX	FREQUENCY	PPM	HEIGHT
1	2180.022	7.265	2.8
2	2179.509	7.263	4.1
3	2178.997	7.261	7.1
4	2177.971	7.258	128.2
5	2177.201	7.255	10.8
6	2176.689	7.254	4.9
7	2176.176	7.252	2.7
8	2049.746	6.831	10.6
9	2041.796	6.804	20.8
10	2027.691	6.757	26.2
11	2025.126	6.749	6.5
12	2019.228	6.729	7.5
13	2017.176	6.722	5.0
14	1163.197	3.876	144.0
15	1159.350	3.863	143.8
16	1152.939	3.842	7.0
17	1146.528	3.821	9.3
18	1140.630	3.801	9.3
19	1134.988	3.782	4.2
20	1120.626	3.734	17.0
21	1112.933	3.709	17.4
22	1080.877	3.602	83.3
23	1077.030	3.589	4.2
24	1009.583	3.364	127.1
25	883.154	2.943	11.0
26	876.486	2.921	21.7
27	869.818	2.899	10.2
28	604.905	2.016	3.7
29	597.981	1.993	4.8
30	591.313	1.971	3.8
31	372.048	1.240	2.8
32	258.954	0.863	96.3
33	252.286	0.841	93.2
34	19.173	0.064	27.3

Compound 8f
^{13}C NMR

No.	(ppm)	(Hz)	Height
1	19.70	1982.6	0.5730
2	27.68	2785.9	0.3126
3	28.92	2910.6	0.3000
4	29.78	2997.2	0.2791
5	35.29	3551.0	0.2445
6	44.40	4468.0	0.2490
7	50.49	5080.6	0.2154
8	55.89	5624.2	0.3337
9	55.98	5633.0	0.3469
10	76.74	7722.3	0.9942
11	77.06	7754.6	1.0000
12	77.37	7786.1	0.9669
13	103.18	10383.8	0.1249
14	111.39	11209.8	0.2350
15	111.90	11260.4	0.2279
16	120.78	12154.7	0.2878
17	130.90	13172.9	0.1777
18	147.95	14888.8	0.1467
19	148.31	14924.7	0.0657
20	149.26	15020.8	0.1378
21	151.74	15270.2	0.1126
22	152.68	15364.1	0.1408
23	153.86	15483.0	0.1181

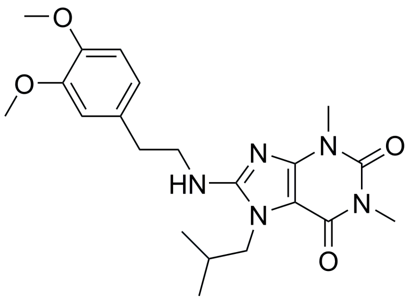

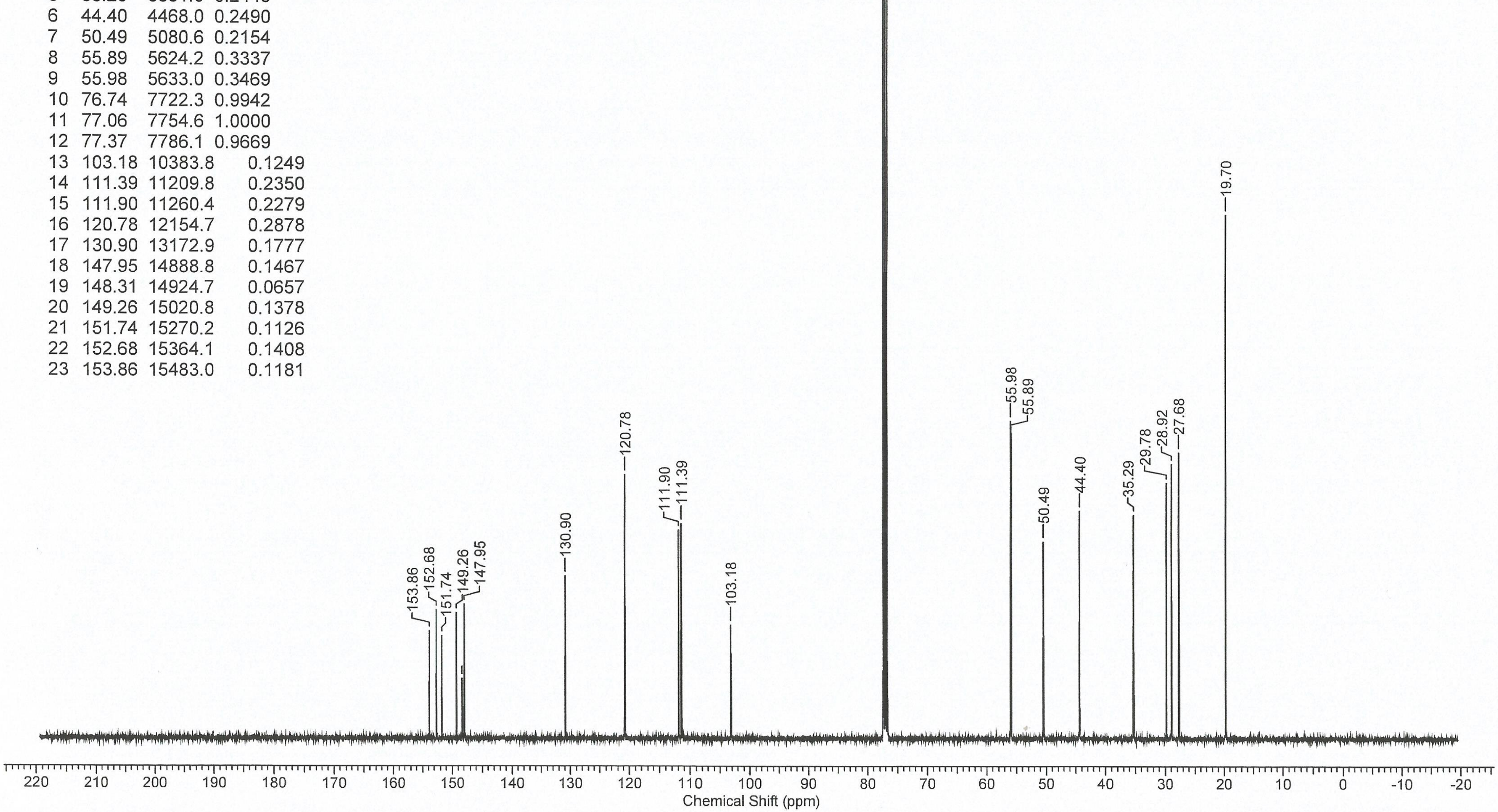

Compound 8g
^{1}H NMR - zoom of the aromatic region

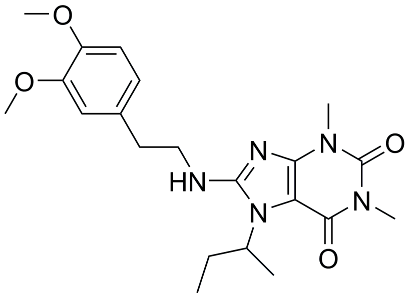

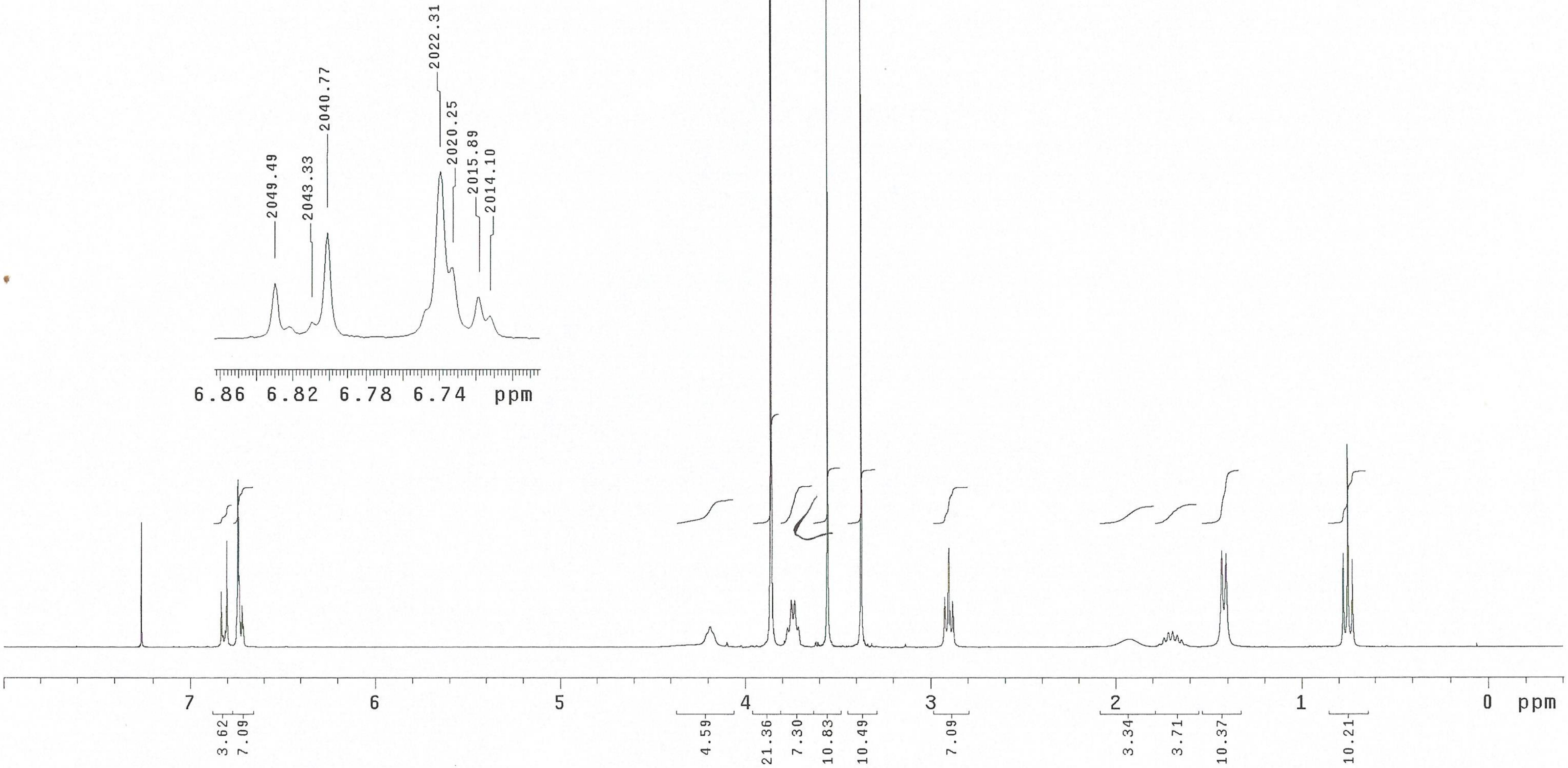

Compound 8g
^{1}H NMR - zoom of the aliphatic region

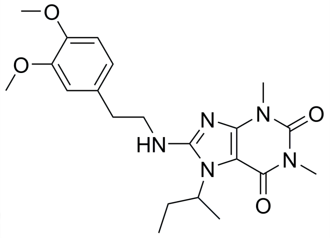

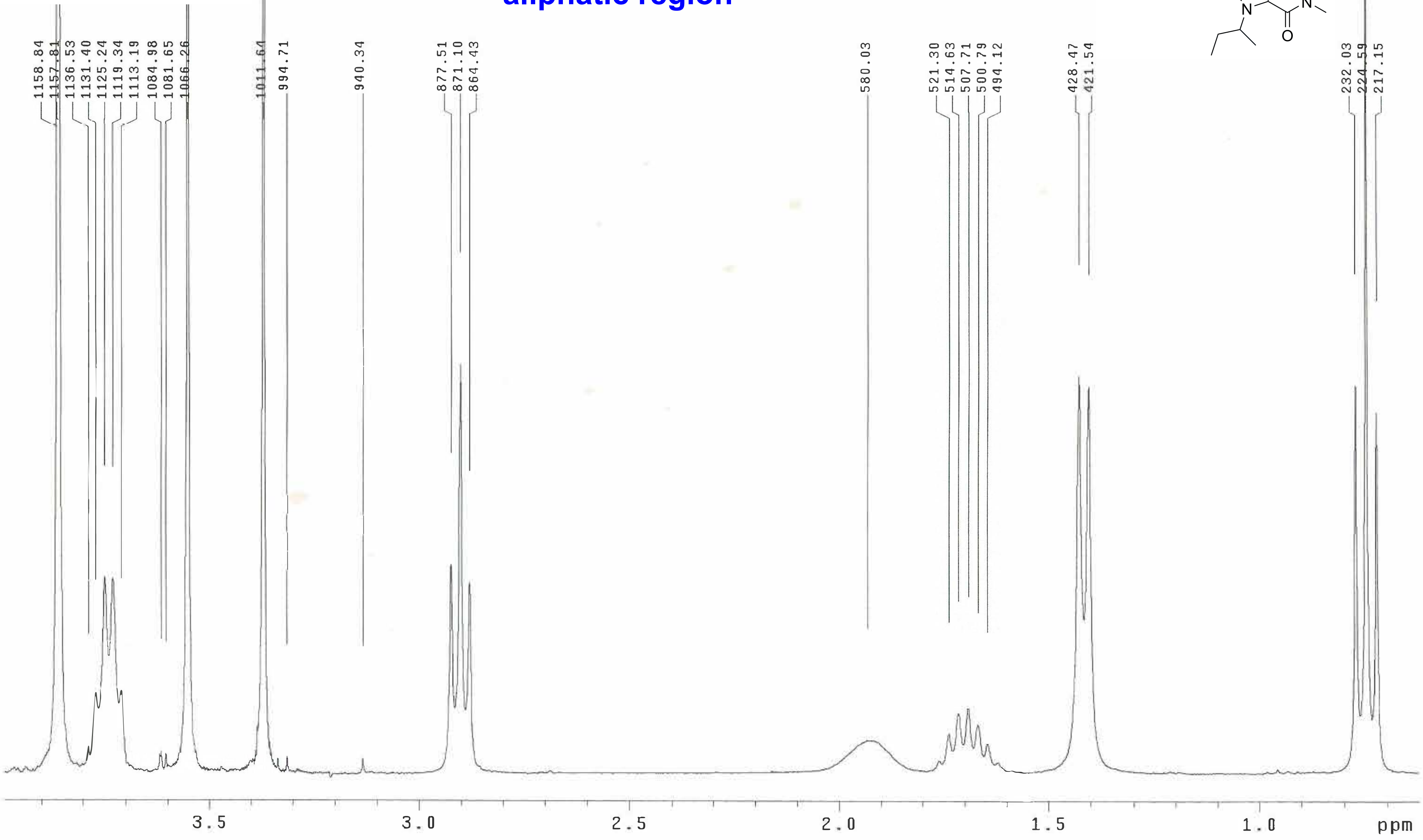

Table of peaks

INDEX	FREQUENCY	PPM	HEIGHT
1	2177.971	7.258	20.1
2	2049.489	6.830	9.0
3	2043.334	6.809	2.7
4	2040.770	6.801	17.2
5	2022.305	6.739	27.1
6	2020.254	6.732	11.6
7	2015.894	6.718	6.7
8	2014.099	6.712	3.7
9	1257.058	4.189	3.3
10	1158.838	3.862	137.2
11	1157.812	3.858	144.0
12	1131.397	3.770	3.1
13	1125.243	3.750	7.6
14	1119.344	3.730	7.6
15	1113.189	3.710	3.2
16	1066.259	3.553	120.0
17	1011.635	3.371	123.7
18	877.512	2.924	8.1
19	871.100	2.903	16.0
20	864.433	2.881	7.4
21	507.710	1.692	2.5
22	428.467	1.428	15.6
23	421.543	1.405	15.1
24	232.026	0.773	15.2
25	224.589	0.748	32.7
26	217.152	0.724	14.1

Compound 8g
^{13}C NMR

No.	(ppm)	(Hz)	Height
1	11.29	1136.1	0.2907
2	19.46	1958.4	0.0927
3	27.99	2816.7	0.2555
4	28.10	2827.7	0.1751
5	29.81	2999.4	0.2927
6	35.27	3549.6	0.2798
7	44.68	4496.7	0.2979
8	53.34	5368.2	0.0419
9	55.90	5624.9	0.3800
10	55.98	5633.0	0.4598
11	76.75	7723.7	0.9732
12	77.07	7755.3	1.0000
13	77.39	7787.6	0.9911
14	102.90	10355.1	0.1194
15	111.41	11211.3	0.3085
16	111.92	11262.6	0.2753
17	120.82	12158.3	0.3501
18	131.02	13184.6	0.1926
19	147.93	14886.6	0.1697
20	149.26	15020.1	0.2016
21	151.69	15265.1	0.1427
22	152.58	15354.6	0.1545
23	153.62	15459.5	0.0758

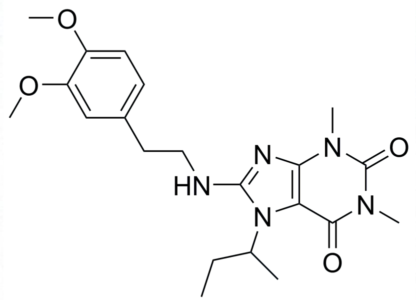

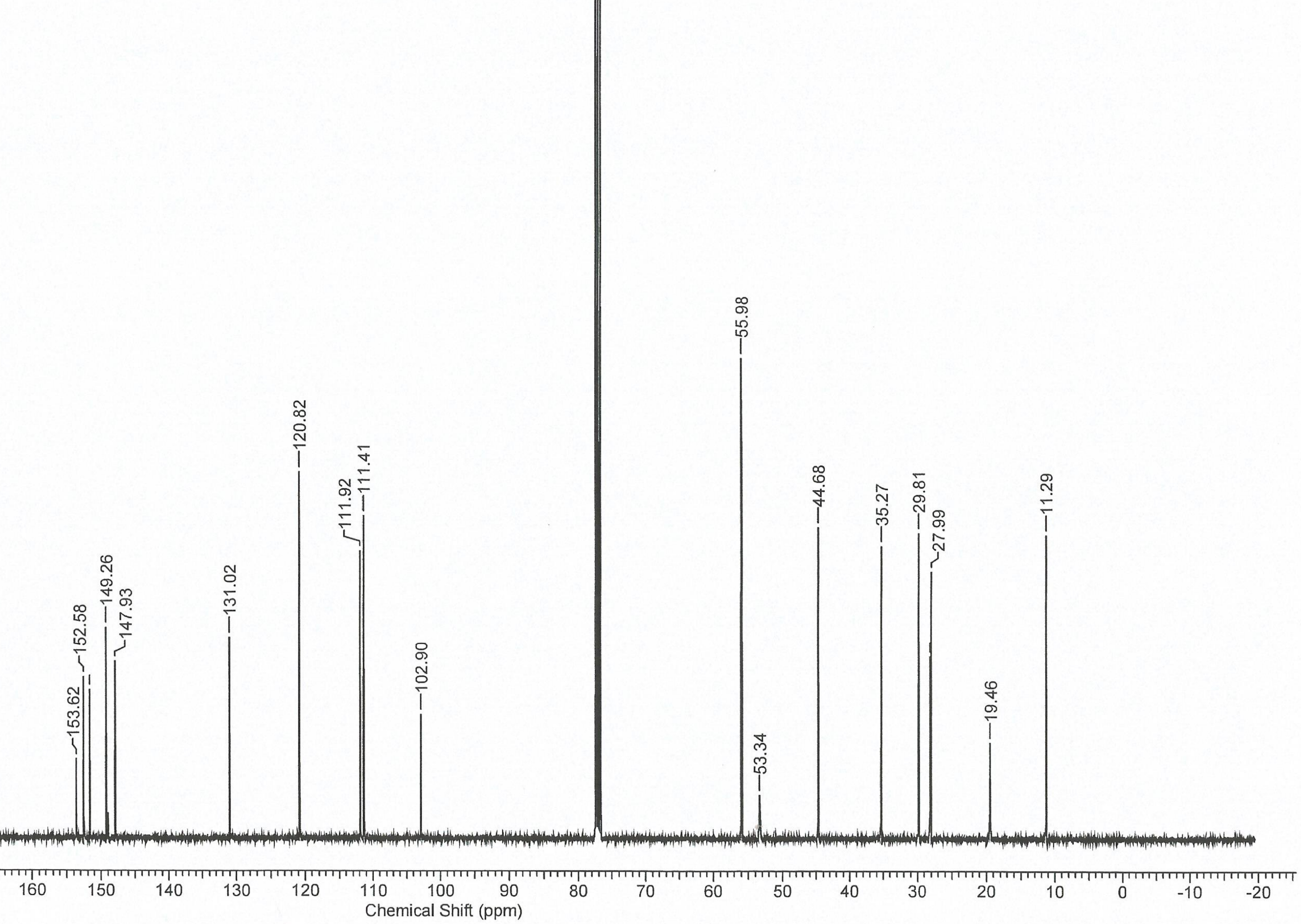

Compound 8h
^{1}H NMR - zoom of the aromatic region

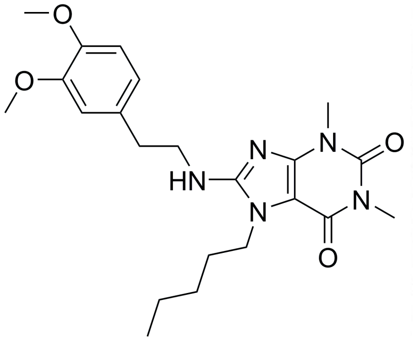

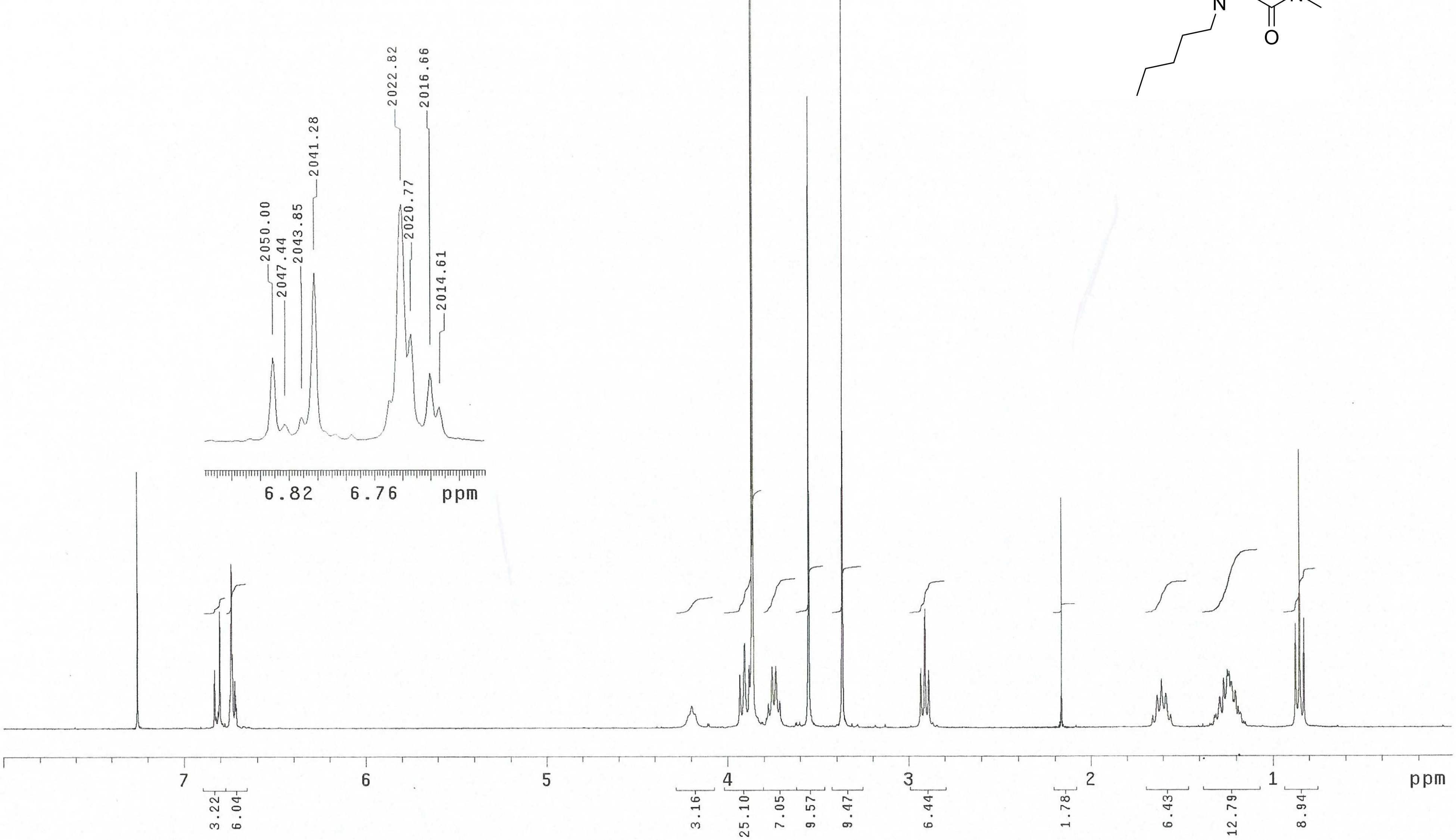

Compound 8h
^{1}H NMR - zoom of the aliphatic region

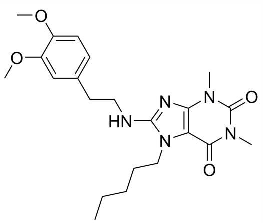

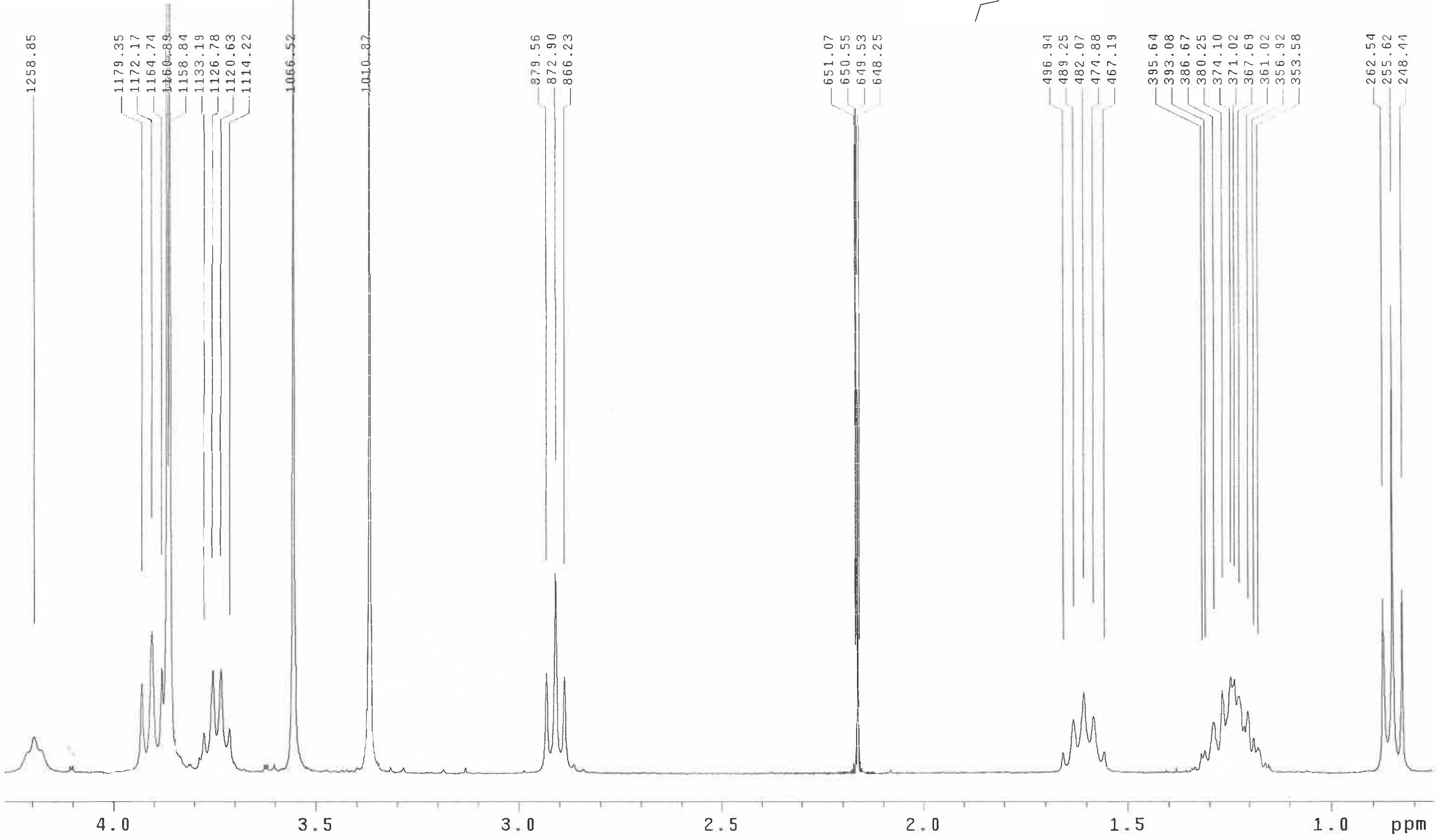

Table of peaks

INDEX	FREQUENCY	PPM	HEIGHT
1	2177.971	7.258	11.7
2	2050.002	6.832	10.1
3	2013.847	6.811	3.0
4	2041.283	6.802	20.1
5	2025.126	6.749	5.0
6	2022.818	6.741	28.6
7	2020.767	6.734	12.9
8	2016.661	6.720	8.3
9	2014.612	6.714	4.2
10	1258.853	4.195	3.8
11	1179.354	3.930	9.2
12	1172.173	3.906	14.7
13	1164.736	3.881	10.9
14	1160.889	3.869	138.6
15	1158.838	3.862	144.0
16	1133.192	3.776	4.2
17	1126.781	3.755	10.6
18	1120.626	3.734	10.8
19	1114.215	3.713	4.7
20	1066.515	3.554	109.8
21	1010.866	3.369	132.5
22	879.563	2.931	10.3
23	872.896	2.909	20.7
24	866.228	2.887	10.0
25	650.553	2.168	2.6
26	649.527	2.165	39.9
27	489.246	1.630	5.6
28	482.065	1.606	8.5
29	474.885	1.583	5.9
30	386.666	1.289	5.3
31	380.255	1.267	8.6
32	374.100	1.247	10.0
33	371.022	1.236	9.7
34	367.689	1.225	8.0
35	363.842	1.212	4.9
36	361.021	1.203	6.4
37	356.918	1.189	3.7
38	353.584	1.178	2.7
39	262.544	0.875	18.0
40	255.620	0.852	48.4
41	248.439	0.828	18.9

Compound 8h
^{13}C NMR

No.	(ppm)	(Hz)	Height
1	13.88	1397.2	0.2319
2	22.29	2243.0	0.2676
3	27.68	2785.9	0.2164
4	28.62	2880.5	0.2672
5	29.20	2938.5	0.2034
6	29.78	2997.2	0.1864
7	35.29	3551.8	0.1858
8	43.36	4363.9	0.1494
9	44.36	4464.4	0.1727
10	55.89	5624.2	0.2573
11	55.92	5627.1	0.2707
12	76.74	7722.3	0.9309
13	77.05	7753.8	1.0000
14	77.37	7786.1	0.9626
15	102.84	10349.3	0.1036
16	111.33	11203.9	0.1703
17	111.85	11256.0	0.1777
18	120.76	12152.5	0.2267
19	130.89	13171.4	0.1245
20	147.94	14887.3	0.1238
21	148.34	14928.4	0.0422
22	149.26	15020.8	0.1414
23	151.75	15271.0	0.1062
24	152.38	15334.8	0.0951
25	153.89	15486.7	0.1168

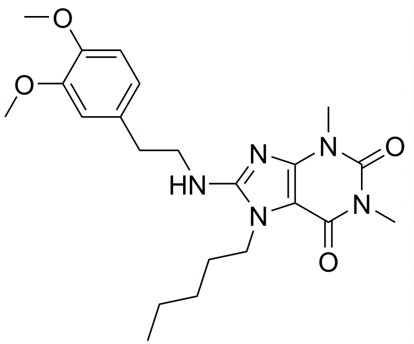

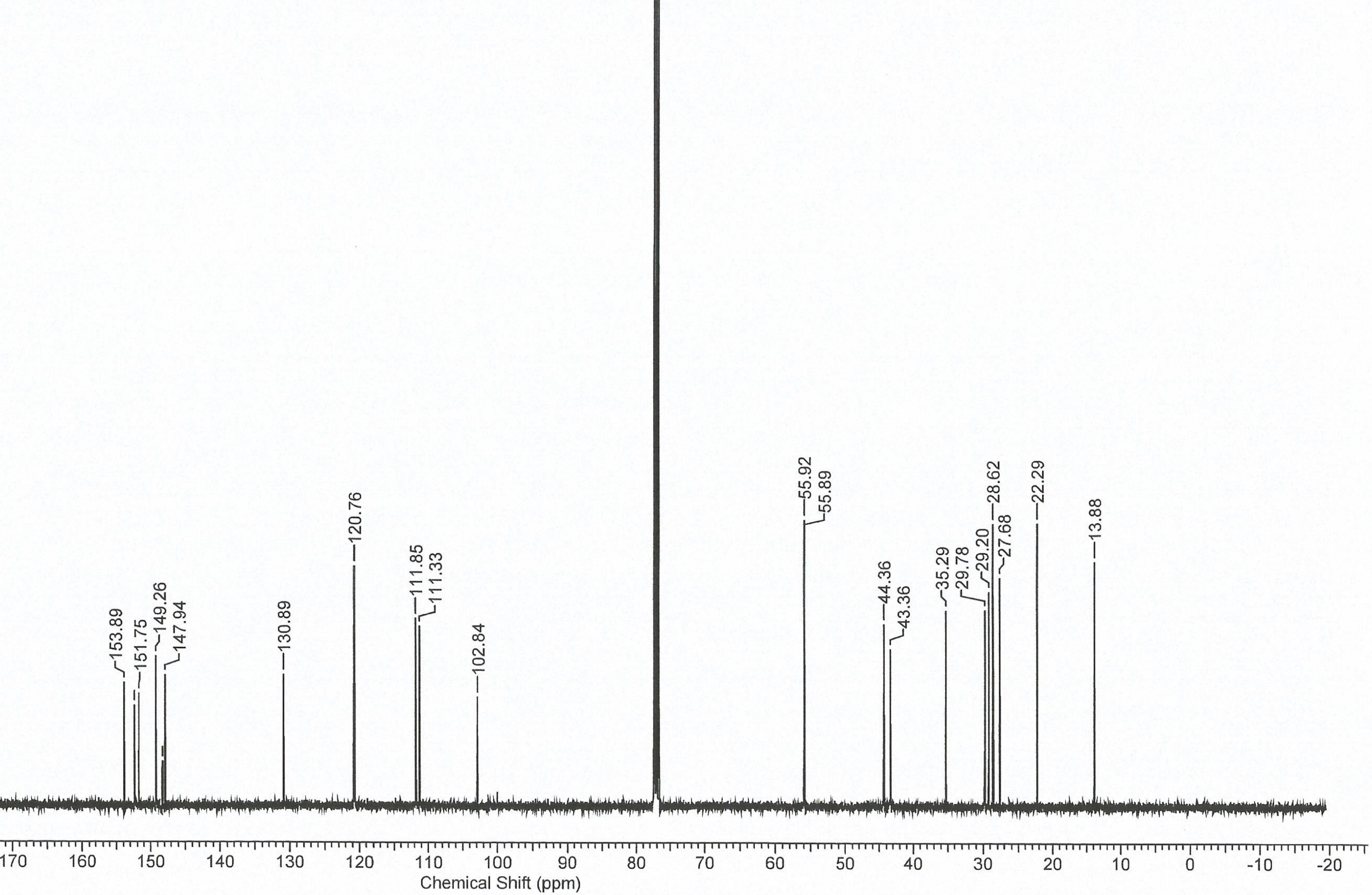

Compound 8i
^{1}H NMR

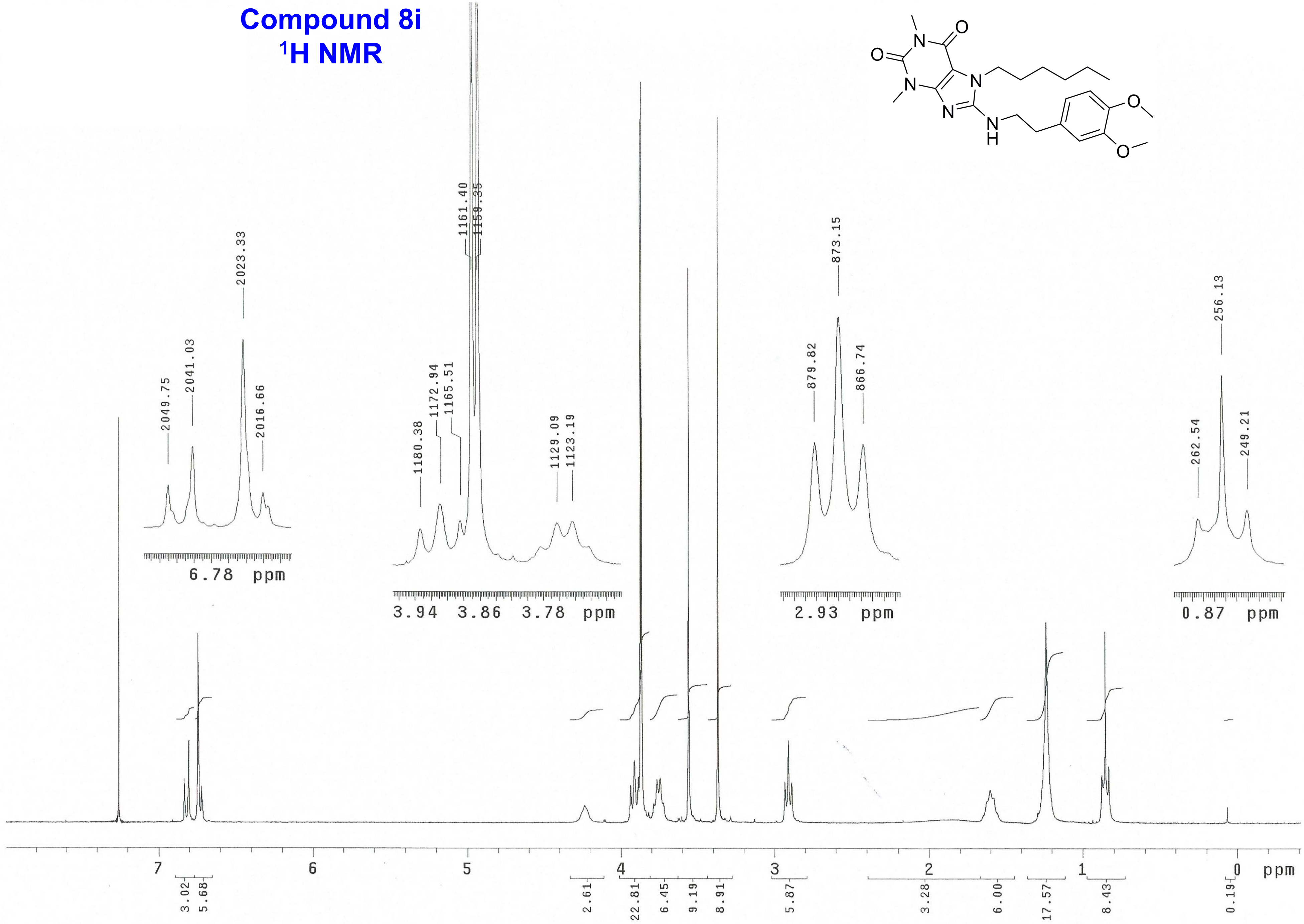

Table of peaks

INDEX	FREQUENCY	PPM	HEIGHT
1	2179.253	7.262	3.6
2	2178.227	7.259	78.8
3	2049.746	6.831	8.4
4	2041.026	6.802	15.9
5	2023.331	6.743	36.7
6	2016.664	6.720	7.1
7	2014.868	6.714	4.4
8	1270.650	4.234	3.3
9	1180.379	3.934	7.3
10	1172.942	3.909	12.1
11	1165.505	3.884	8.9
12	1161.402	3.870	136.7
13	1159.350	3.863	144.0
14	1135.244	3.783	3.8
15	1129.089	3.763	8.4
16	1123.191	3.743	8.7
17	1068.567	3.561	108.0
18	1011.122	3.370	137.2
19	879.820	2.932	7.9
20	873.152	2.910	16.0
21	866.741	2.888	7.8
22	480.783	1.602	6.3
23	370.766	1.236	39.0
24	262.544	0.875	9.3
25	256.133	0.854	37.2
26	249.209	0.830	11.0
27	19.173	0.064	3.1

Compound 8i
^{13}C NMR

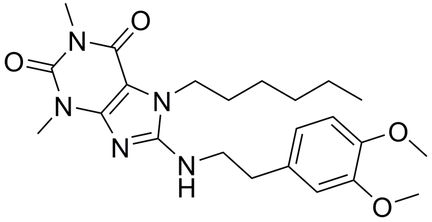

No.	(ppm)	(Hz)	Height
1	13.96	1405.3	0.1855
2	22.49	2262.9	0.1914
3	26.24	2640.7	0.1448
4	27.70	2787.4	0.1573
5	29.47	2965.6	0.1190
6	29.85	3003.8	0.1035
7	31.35	3154.9	0.1711
8	35.31	3553.2	0.1116
9	43.44	4371.2	0.0957
10	44.40	4468.0	0.1184
11	55.90	5624.9	0.1894
12	55.91	5626.4	0.1595
13	76.72	7720.8	0.9441
14	77.04	7752.4	1.0000
15	77.36	7784.6	0.9585
16	102.83	10347.8	0.0612
17	111.32	11202.5	0.1158
18	111.86	11256.7	0.1222
19	120.75	12151.7	0.1477
20	130.86	13168.5	0.0862
21	147.95	14888.8	0.0727
22	149.28	15022.3	0.0835
23	151.73	15268.8	0.0726
24	152.27	15323.1	0.0574
25	153.89	15486.7	0.0742

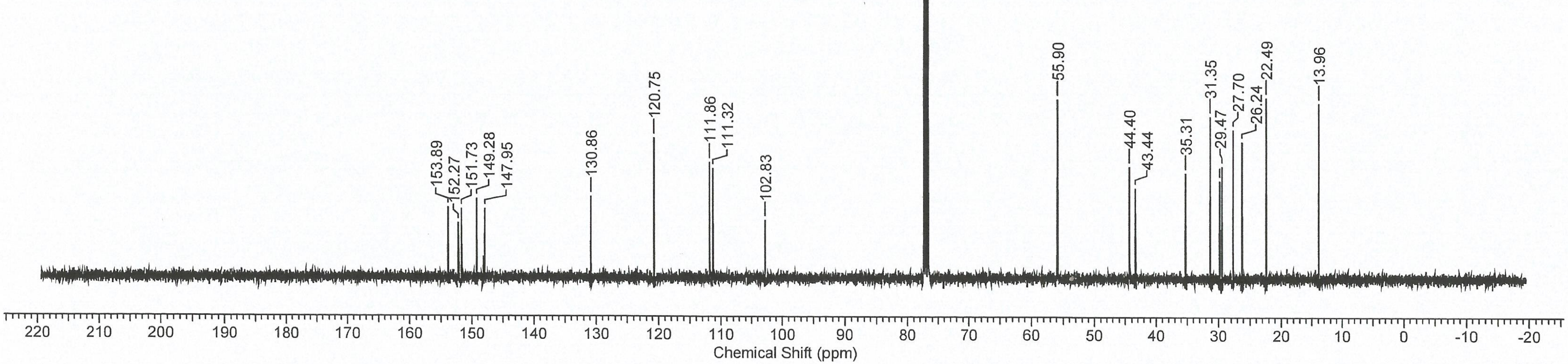

Compound 8j
^{1}H NMR

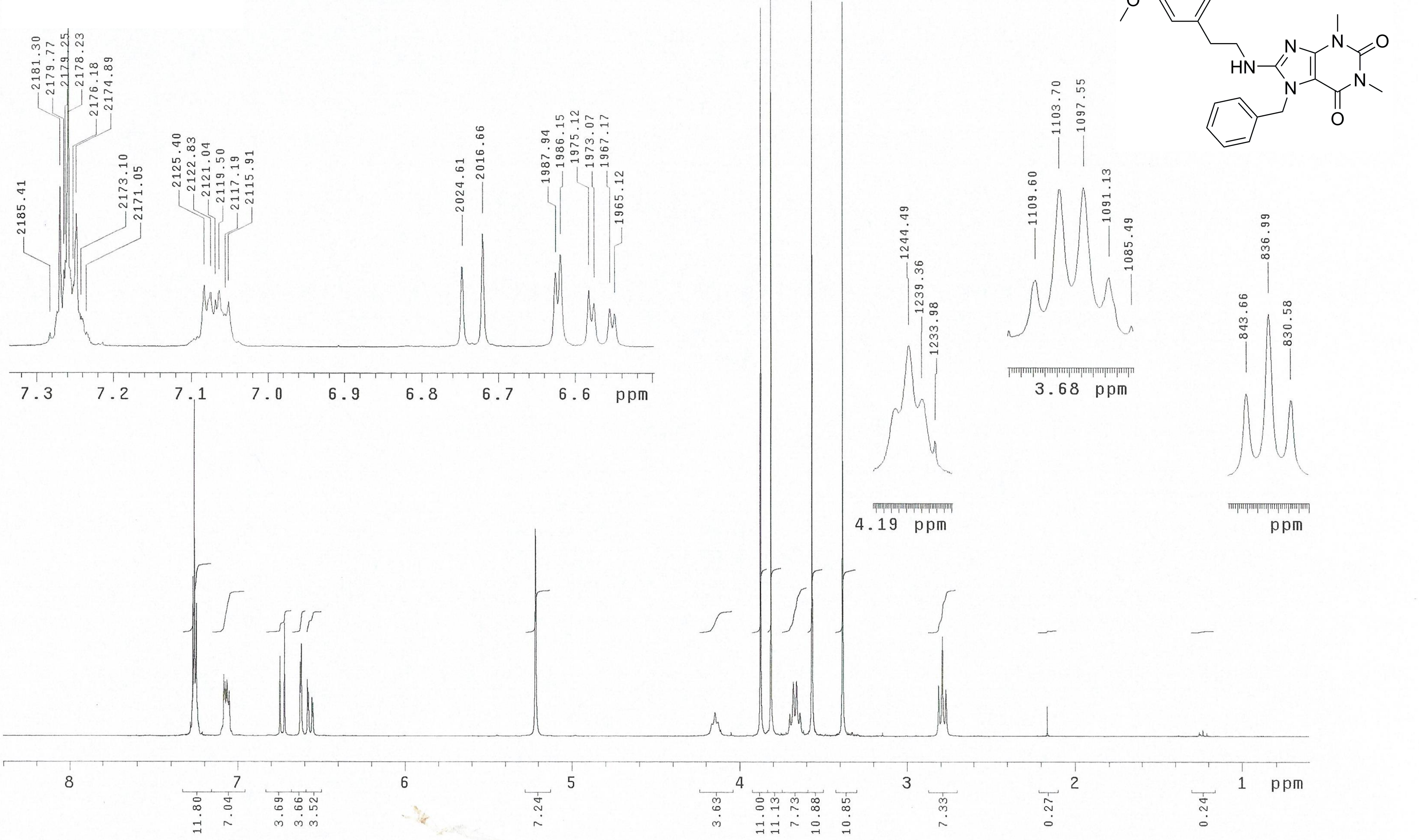

Table of peaks

INDEX	FREQUENCY	PPM	HEIGHT
1	2185.408	7.283	2.6
2	2182.587	7.273	6.8
3	2181.305	7.269	30.7
4	2179.766	7.264	14.8
5	2179.253	7.262	16.6
6	2178.227	7.259	64.9
7	2176.176	7.252	11.9
8	2174.893	7.248	25.5
9	2173.098	7.242	5.8
10	2172.585	7.240	5.4
11	2171.047	7.235	2.8
12	2125.398	7.083	11.9
13	2122.834	7.074	10.5
14	2121.039	7.068	9.1
15	2119.500	7.063	10.9
16	2117.192	7.055	6.4
17	2115.910	7.051	8.7
18	2024.614	6.747	15.4
19	2016.664	6.720	21.7
20	1987.941	6.625	14.3
21	1986.146	6.619	17.8
22	1975.119	6.582	10.9
23	1973.067	6.575	8.4
24	1967.169	6.556	7.5
25	1965.117	6.549	6.5
26	1565.311	5.216	39.9
27	1244.492	4.147	4.6
28	1239.363	4.130	2.7
29	1162.428	3.874	140.5
30	1143.963	3.812	144.0
31	1109.599	3.698	4.3
32	1103.701	3.678	10.5
33	1097.546	3.658	10.7
34	1091.135	3.636	4.5
35	1070.106	3.566	141.8
36	1018.046	3.393	2.7
37	1015.482	3.384	141.6
38	843.660	2.811	9.9
39	836.992	2.789	19.2
40	830.581	2.768	9.1
41	648.758	2.162	5.8

Compound 8j

^{13}C NMR

No.	(ppm)	(Hz)	Height
1	27.65	2086.4	0.0653
2	27.69	2089.7	0.0718
3	29.72	2242.9	0.0636
4	29.77	2246.2	0.0681
5	35.08	2647.0	0.2615
6	44.09	3327.1	0.1574
7	46.63	3518.9	0.1583
8	55.80	4210.5	0.1212
9	55.83	4213.3	0.1186
10	55.90	4218.3	0.1186
11	55.94	4221.0	0.1104
12	76.60	5780.7	1.0000
13	77.02	5812.2	0.9948
14	77.45	5844.3	0.9837
15	102.91	7765.5	0.1312
16	111.34	8401.9	0.3330
17	111.65	8425.6	0.2482
18	120.64	9104.0	0.2447
19	127.00	9583.9	0.5135
20	128.14	9669.6	0.2126
21	129.05	9738.2	0.4411
22	130.57	9853.2	0.2627
23	135.10	10194.8	0.2502
24	147.87	11158.5	0.1797
25	148.45	11202.2	0.1063
26	149.21	11259.7	0.2112
27	151.71	11448.2	0.1288
28	153.06	11550.5	0.2353
29	154.15	11632.8	0.1612

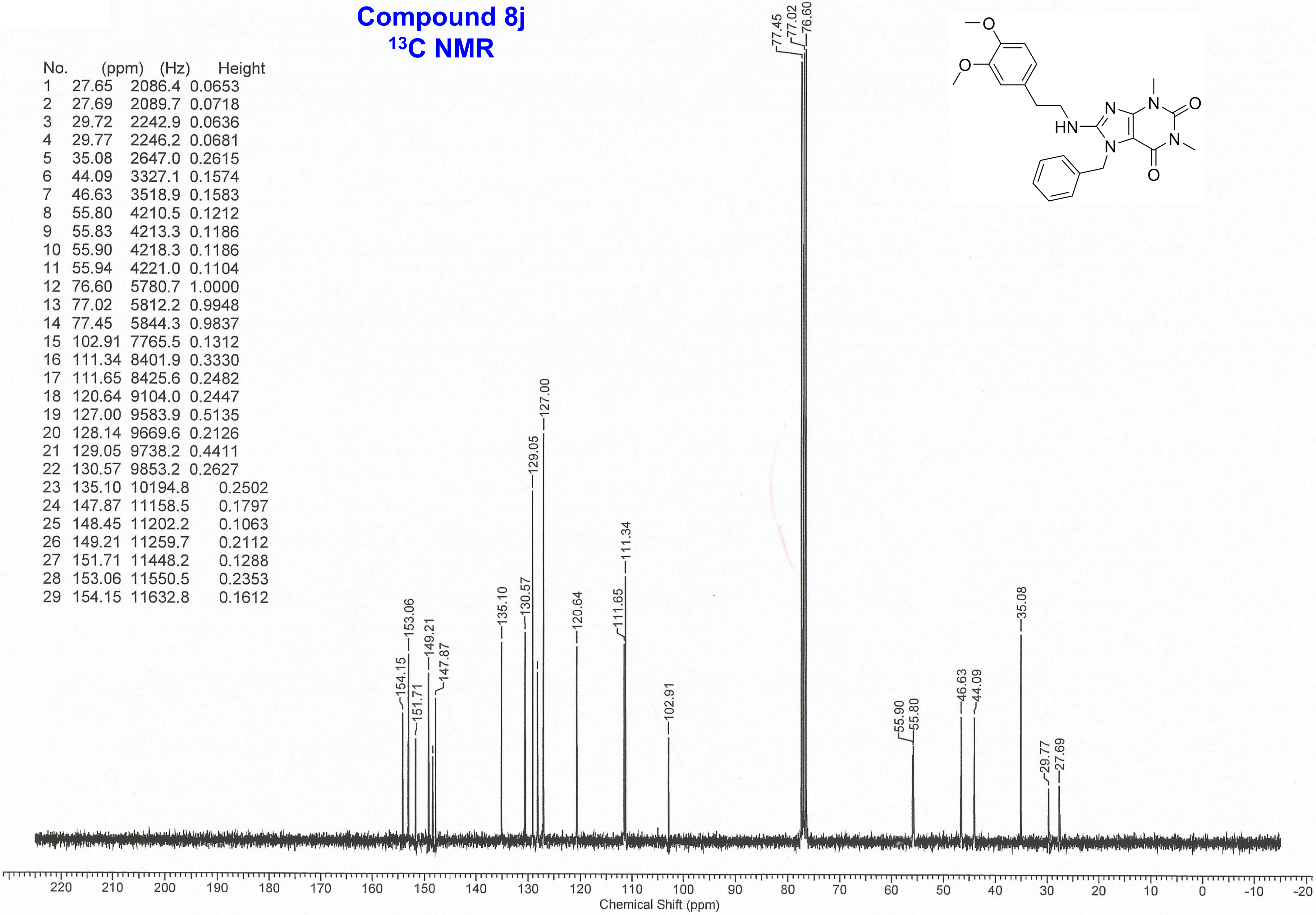

Compound 8k
^{1}H NMR

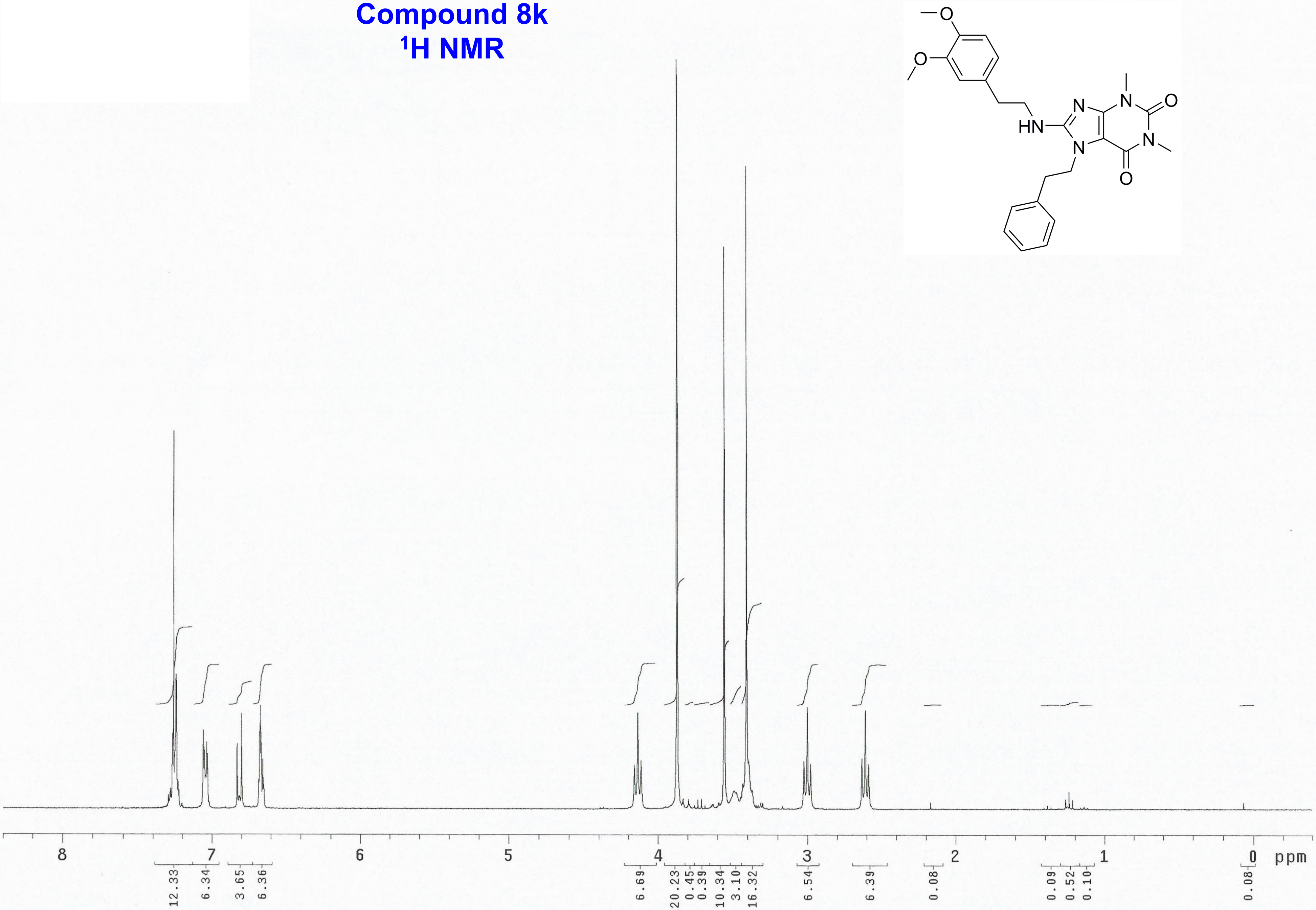

Compound 8k
^{1}H NMR - zoom of the aliphatic region

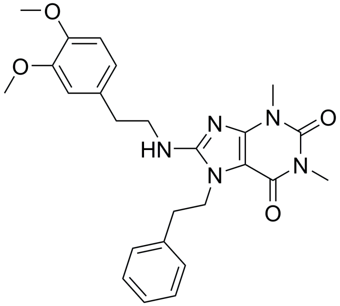

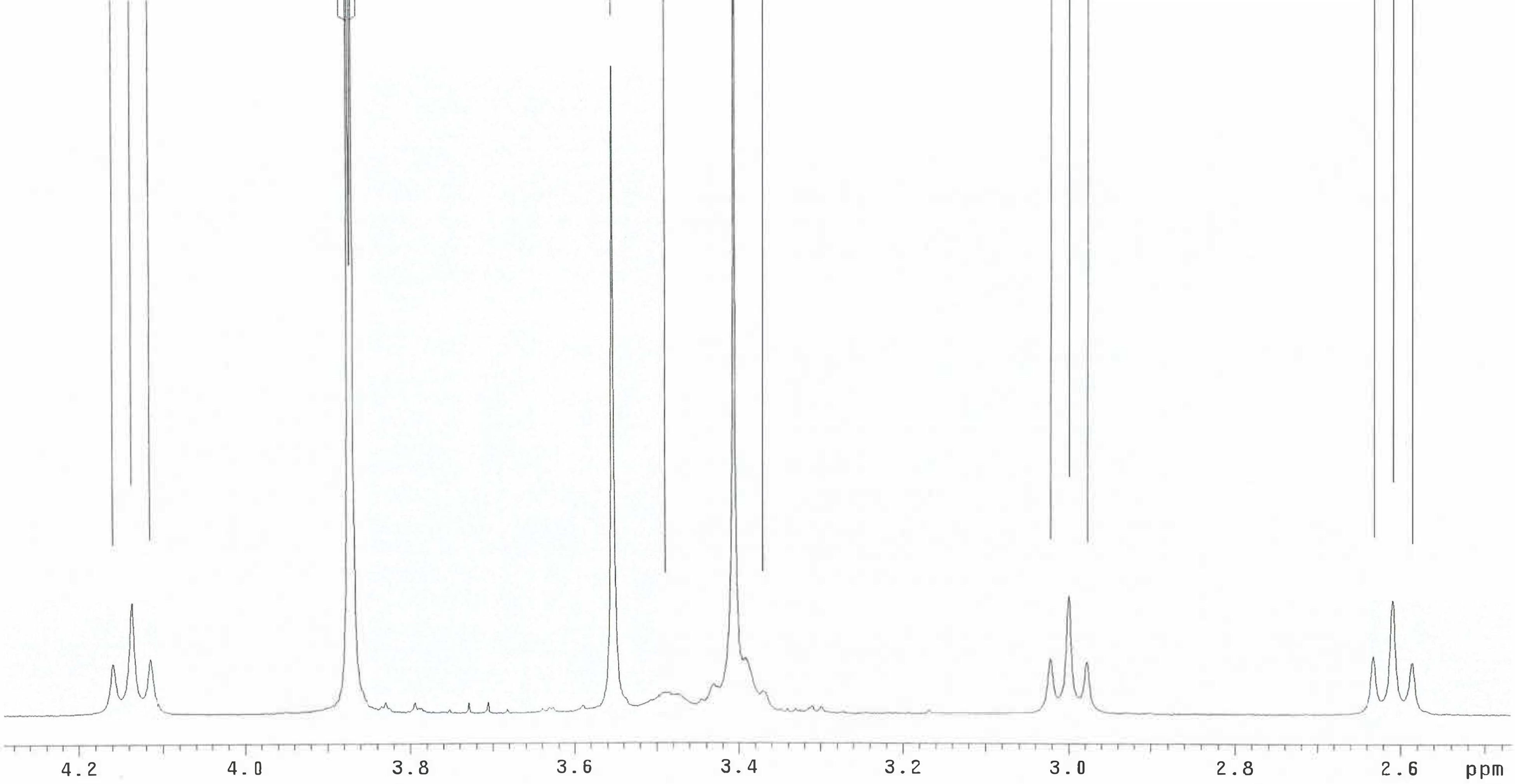

Compound 8k
^{1}H NMR - zoom of the aromatic region

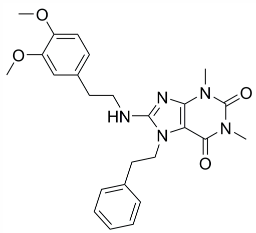

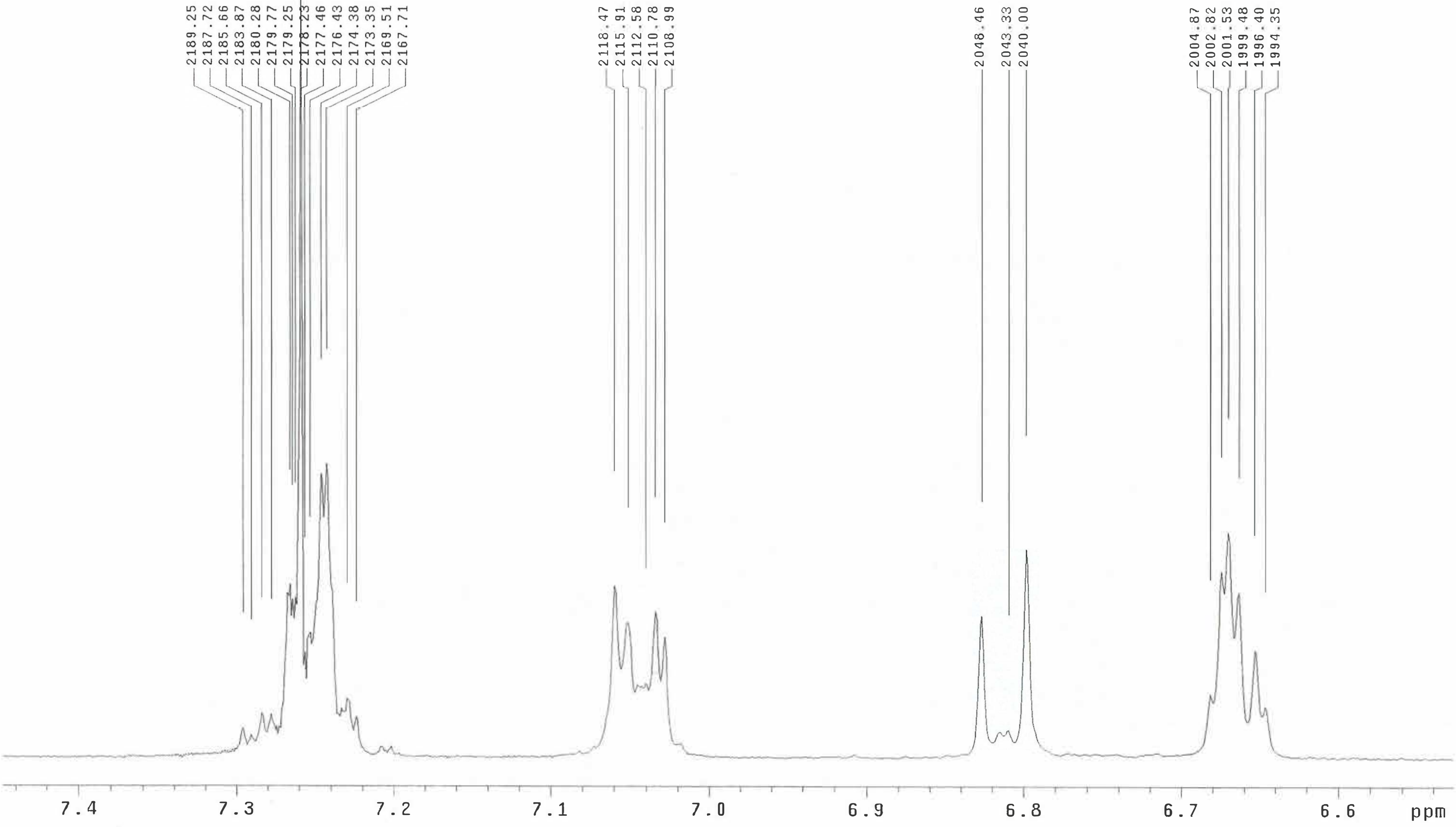

Table of peaks

INDEX	FREQUENCY	PPM	HEIGHT
1	2189.255	7.296	2.6
2	2185.664	7.284	3.9
3	2183.869	7.278	3.8
4	2182.843	7.274	2.7
5	2182.330	7.273	2.7
6	2180.792	7.267	14.4
7	2180.279	7.266	15.2
8	2179.766	7.264	13.8
9	2179.253	7.262	14.0
10	2178.227	7.259	72.8
11	2177.458	7.256	9.2
12	2176.432	7.253	11.0
13	2174.380	7.246	24.9
14	2173.355	7.243	25.8
15	2170.534	7.233	4.3
16	2169.508	7.230	5.2
17	2167.713	7.224	3.6
18	2118.474	7.060	15.1
19	2115.910	7.051	11.9
20	2114.115	7.045	6.4
21	2112.576	7.040	6.6
22	2110.781	7.034	12.8
23	2108.986	7.028	10.6
24	2048.463	6.826	12.5
25	2040.001	6.798	18.3
26	2004.867	6.681	5.6
27	2002.815	6.674	16.4
28	2001.533	6.670	19.8
29	1999.481	6.663	14.6
30	1996.404	6.653	9.5
31	1994.352	6.646	4.6
32	1247.826	4.158	8.5
33	1241.158	4.136	18.6
34	1234.747	4.115	9.3
35	1162.171	3.873	144.0
36	1161.146	3.869	141.1
37	1066.003	3.552	108.1
38	1047.025	3.489	3.5
39	1029.074	3.429	4.8
40	1029.587	3.431	4.9
41	1028.048	3.426	4.5
42	1021.893	3.405	123.5
43	1018.816	3.395	9.2
44	1017.790	3.392	9.3
45	1011.379	3.370	3.8
46	906.747	3.022	9.3
47	900.079	2.999	19.6
48	893.412	2.977	8.7
49	789.549	2.631	9.7
50	782.625	2.608	18.9
51	775.701	2.585	8.6
52	371.535	1.238	3.3

Compound 8k
^{13}C NMR

No.	(ppm)	(Hz)	Height
1	27.72	2789.6	0.2838
2	29.78	2996.5	0.2546
3	35.27	3549.6	0.2345
4	36.45	3667.7	0.2170
5	44.41	4469.5	0.2109
6	45.74	4603.0	0.1676
7	55.90	5624.9	0.2742
8	55.96	5631.5	0.2969
9	76.74	7722.3	0.9504
10	77.06	7754.6	1.0000
11	77.37	7786.1	0.9122
12	102.60	10325.1	0.1073
13	111.33	11203.9	0.2207
14	111.85	11256.0	0.2235
15	120.67	12143.7	0.2802
16	127.02	12782.6	0.2585
17	128.84	12965.3	0.4751
18	128.98	12980.0	0.5253
19	130.98	13181.0	0.1602
20	138.26	13913.1	0.1976
21	147.88	14881.4	0.1419
22	148.63	14957.0	0.0843
23	149.18	15012.0	0.1368
24	151.81	15276.8	0.1255
25	153.00	15397.1	0.1539
26	154.00	15497.7	0.1447

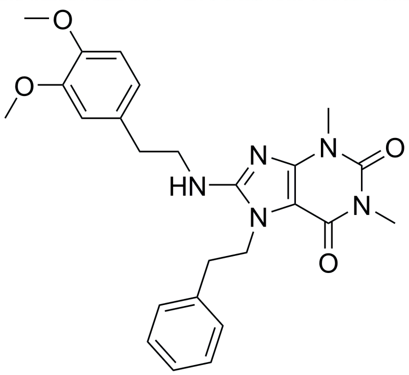

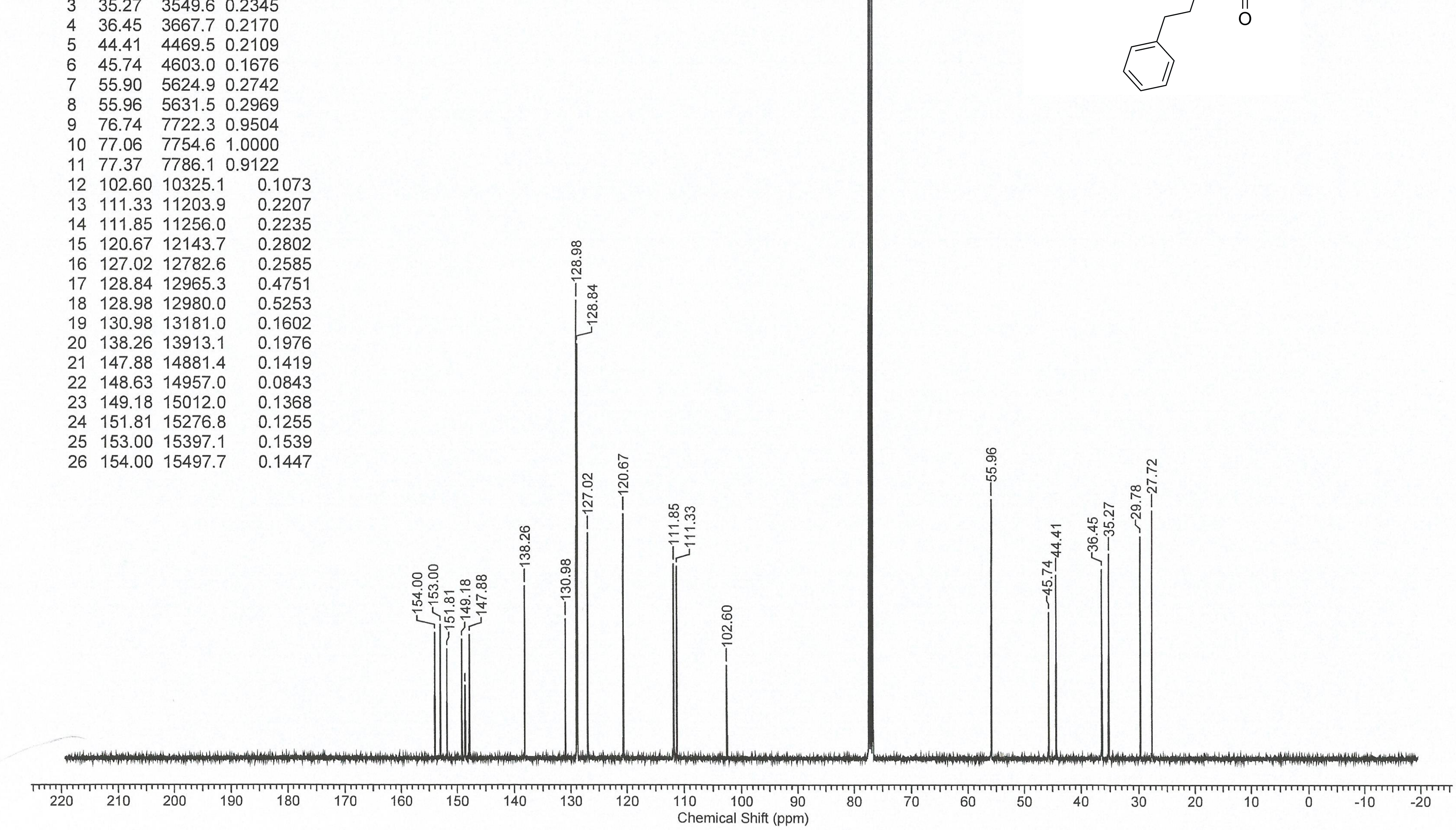

Compound 8l
^{1}H NMR

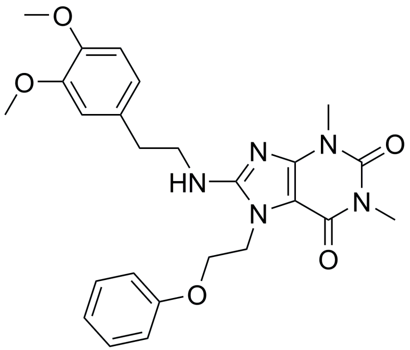

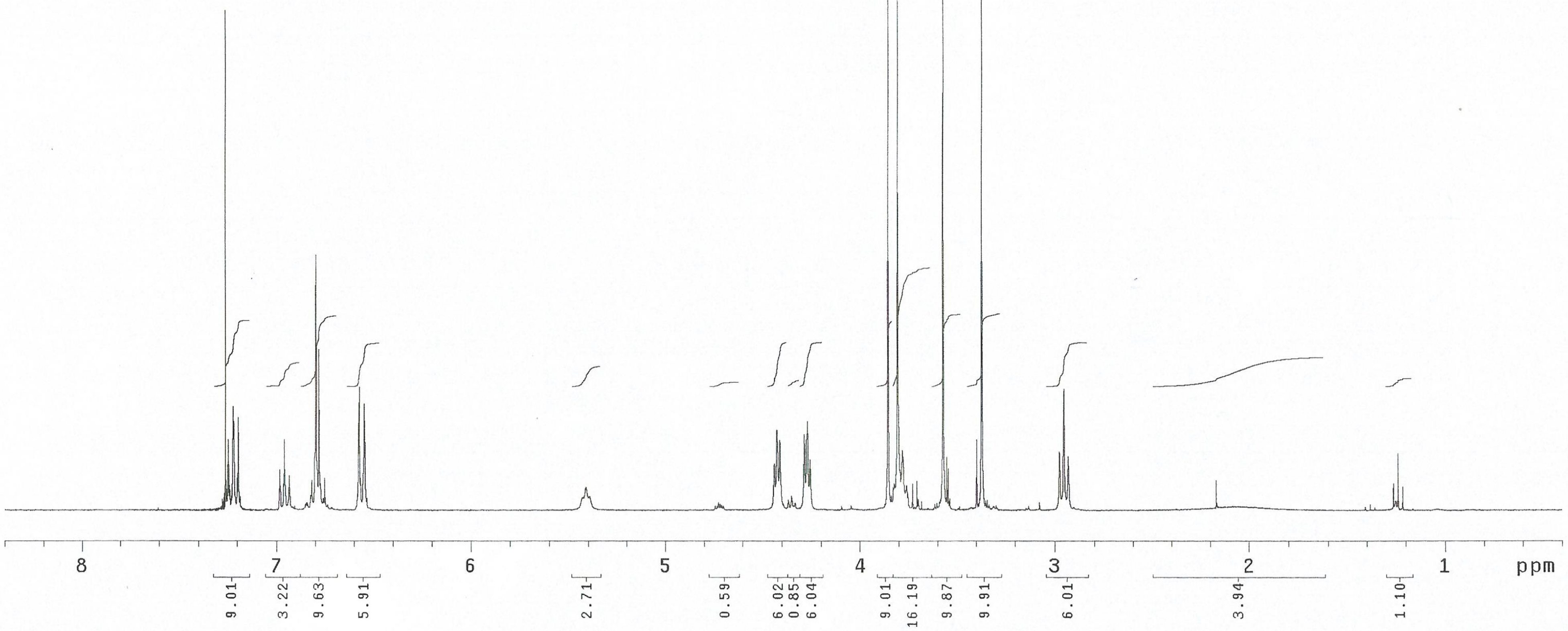

Compound 8l
^{1}H NMR - zoom of the aliphatic region

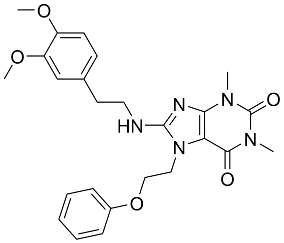

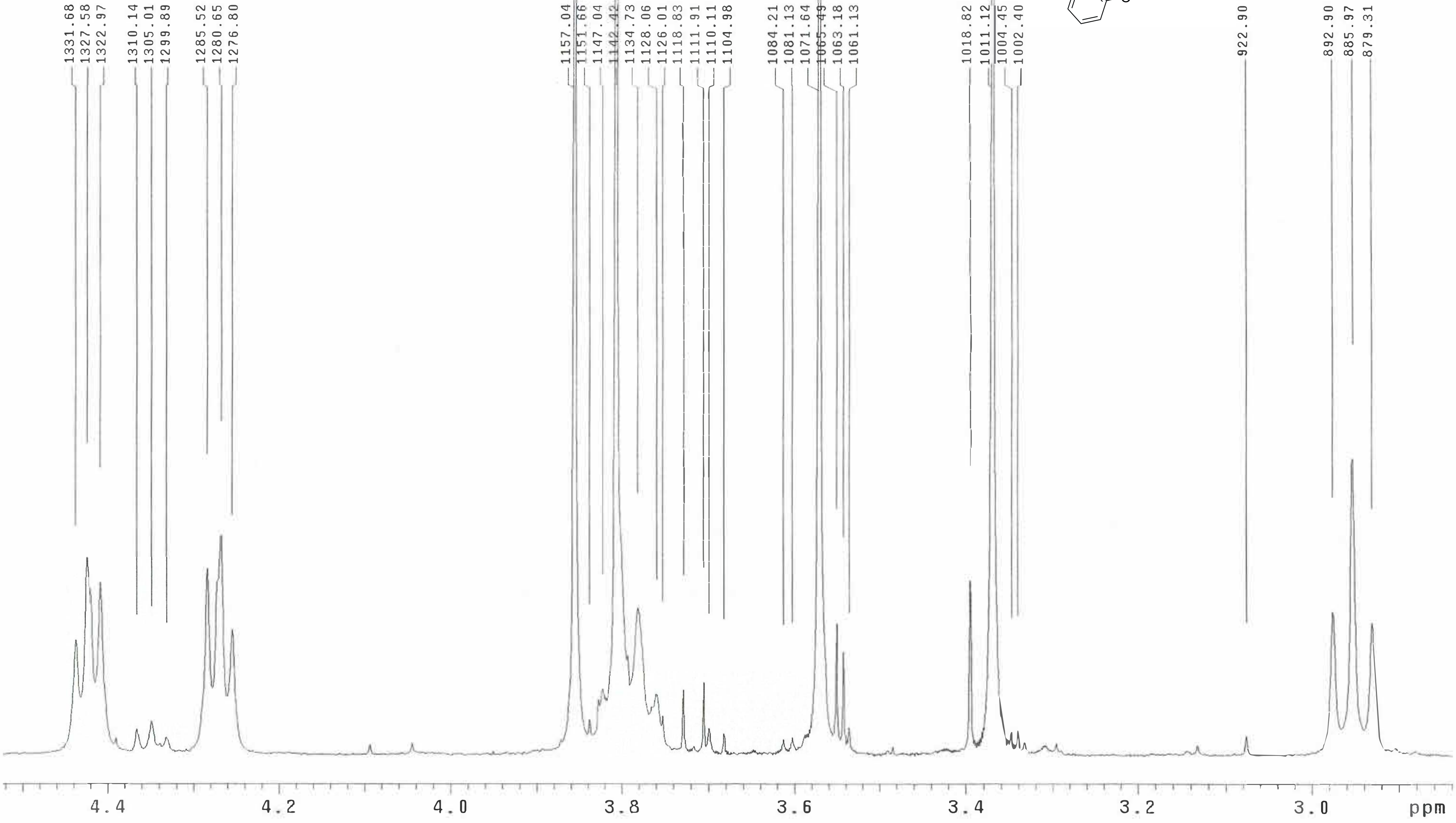

Compound 8l
^{1}H NMR - zoom of the aromatic region

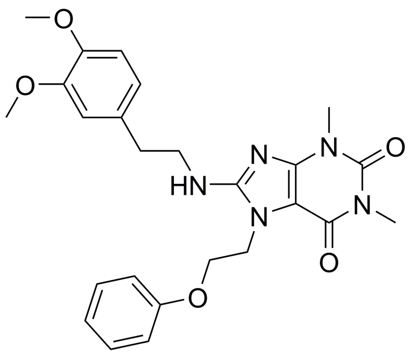

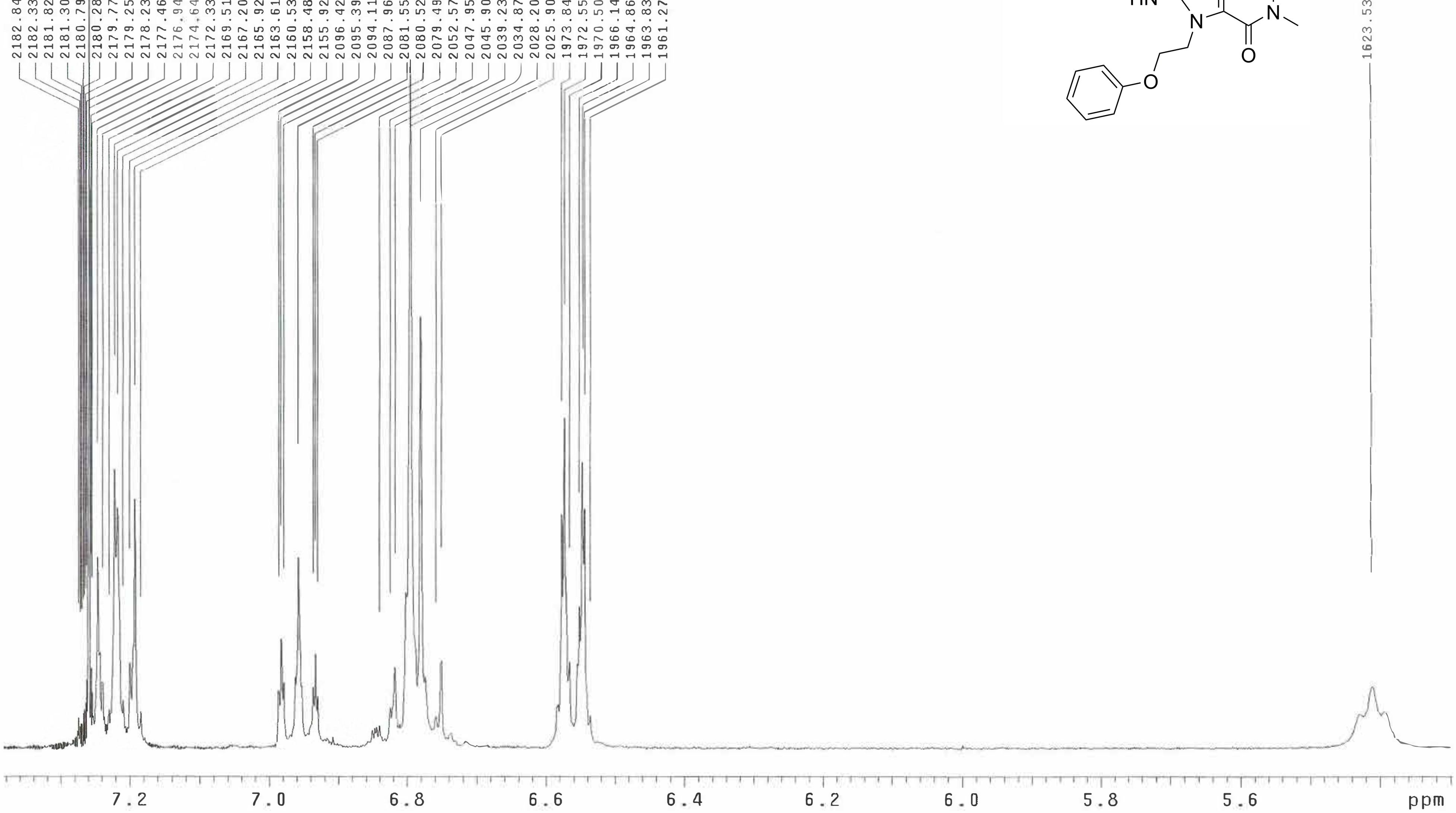

Table of peaks

INDEX	FREQUENCY	PPM	HEIGHT
1	2179.766	7.264	2.7
2	2179.253	7.262	4.1
3	2178.227	7.259	80.9
4	2177.458	7.256	4.8
5	2176.945	7.255	3.3
6	2174.637	7.247	11.4
7	2172.329	7.239	4.0
8	2167.200	7.222	16.8
9	2165.918	7.218	14.4
10	2163.610	7.210	2.9
11	2160.532	7.200	5.1
12	2158.481	7.193	15.0
13	2096.420	6.986	3.4
14	2095.394	6.983	6.5
15	2094.112	6.979	3.9
16	2089.239	6.962	4.2
17	2087.957	6.958	11.4
18	2081.545	6.937	3.7
19	2080.520	6.933	5.6
20	2079.494	6.930	3.1
21	2045.899	6.818	4.9
22	2041.026	6.802	9.3
23	2039.231	6.796	41.3
24	2034.872	6.781	26.0
25	2032.820	6.774	4.3
26	2025.896	6.751	5.2
27	1975.375	6.583	2.6
28	1973.836	6.578	14.0
29	1972.554	6.573	19.9
30	1970.503	6.567	5.2
31	1966.143	6.552	8.5
32	1964.861	6.548	17.2
33	1963.835	6.544	14.4
34	1623.525	5.410	3.7
35	1331.685	4.438	7.6
36	1327.582	4.424	13.0
37	1322.966	4.409	11.4
38	1285.524	4.284	12.3
39	1280.651	4.268	14.4
40	1276.805	4.255	8.2
41	1157.042	3.856	136.4
42	1148.580	3.828	3.7
43	1147.041	3.822	4.3
44	1142.425	3.807	144.0
45	1138.322	3.793	6.6
46	1134.731	3.781	9.7
47	1128.063	3.759	4.0
48	1126.012	3.752	2.6
49	1118.831	3.728	4.3
50	1111.907	3.705	4.8
51	1071.644	3.571	119.2
52	1065.490	3.551	8.7
53	1063.182	3.543	6.8
54	1018.816	3.395	11.5
55	1011.122	3.370	129.0
56	892.899	2.976	9.4
57	885.974	2.952	19.5
58	879.307	2.930	8.7
59	650.040	2.166	4.8
60	378.716	1.262	4.4
61	371.792	1.239	9.2
62	364.868	1.216	3.8

Compound 8l
^{13}C NMR

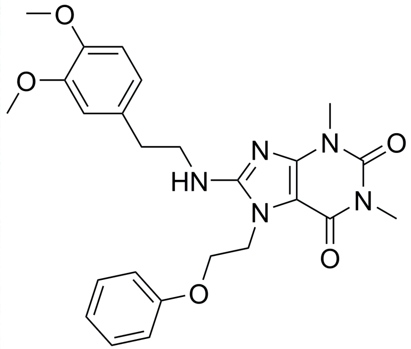

No.	(ppm)	(Hz)	Height
1	27.65	2782.2	0.1882
2	29.86	3005.3	0.1727
3	35.59	3581.1	0.1426
4	43.87	4415.2	0.1209
5	44.65	4493.0	0.1482
6	55.86	5621.3	0.2182
7	55.88	5623.5	0.2061
8	68.45	6888.2	0.1414
9	76.73	7721.5	0.9502
10	77.04	7753.1	1.0000
11	77.36	7785.4	0.9776
12	102.73	10338.3	0.0802
13	111.41	11212.0	0.1552
14	111.90	11261.1	0.1562
15	114.25	11497.4	0.3318
16	120.72	12148.1	0.1944
17	121.88	12264.7	0.1656
18	129.60	13041.6	0.4027
19	131.08	13190.5	0.1160
20	147.94	14888.0	0.0799
21	148.52	14946.0	0.0498
22	149.25	15019.3	0.1044
23	151.71	15266.6	0.0819
24	154.21	15518.9	0.0954
25	154.52	15549.7	0.1026
26	157.39	15838.8	0.0977

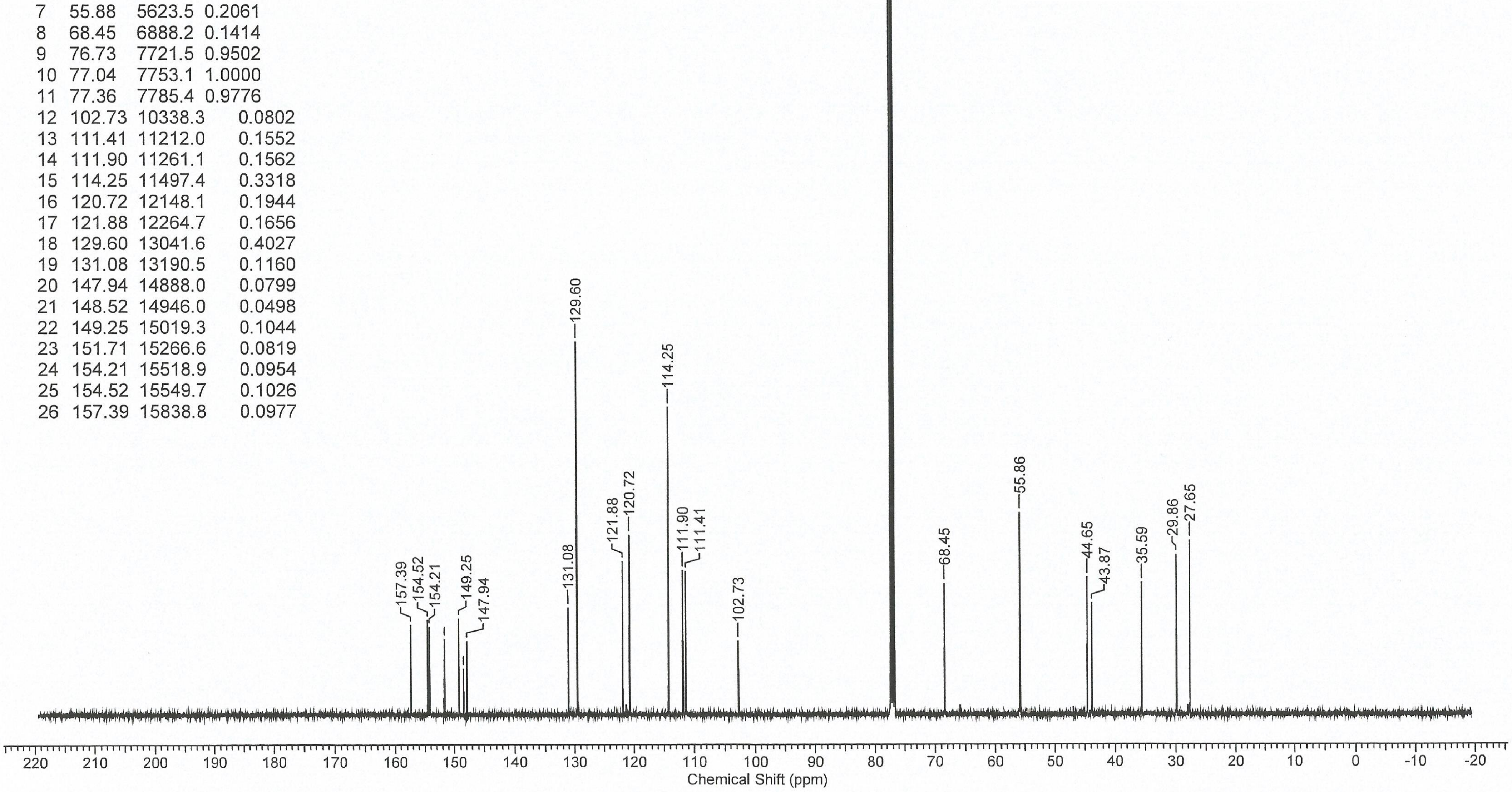

Compound 9

^{1}H NMR - zoom of the aromatic region

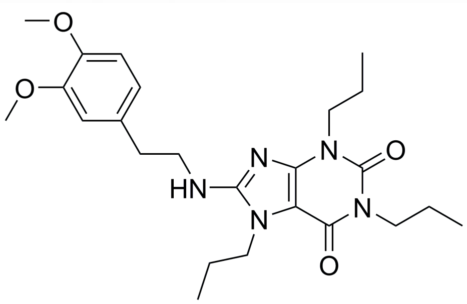

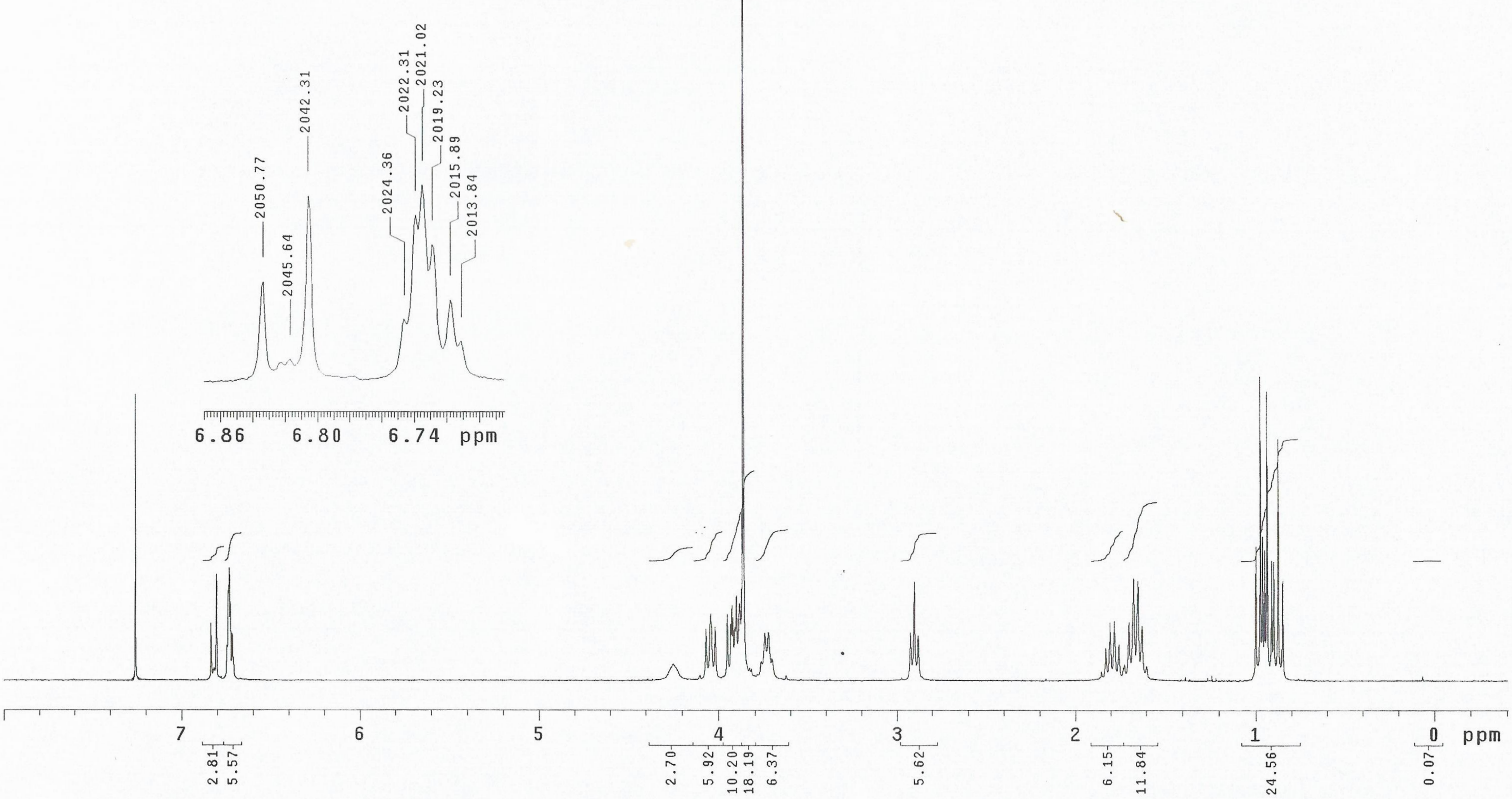

Compound 9
^{1}H NMR - zoom of the aliphatic region

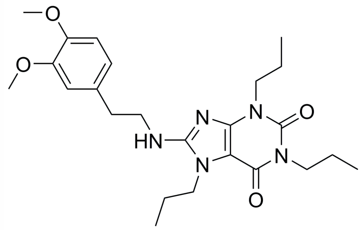

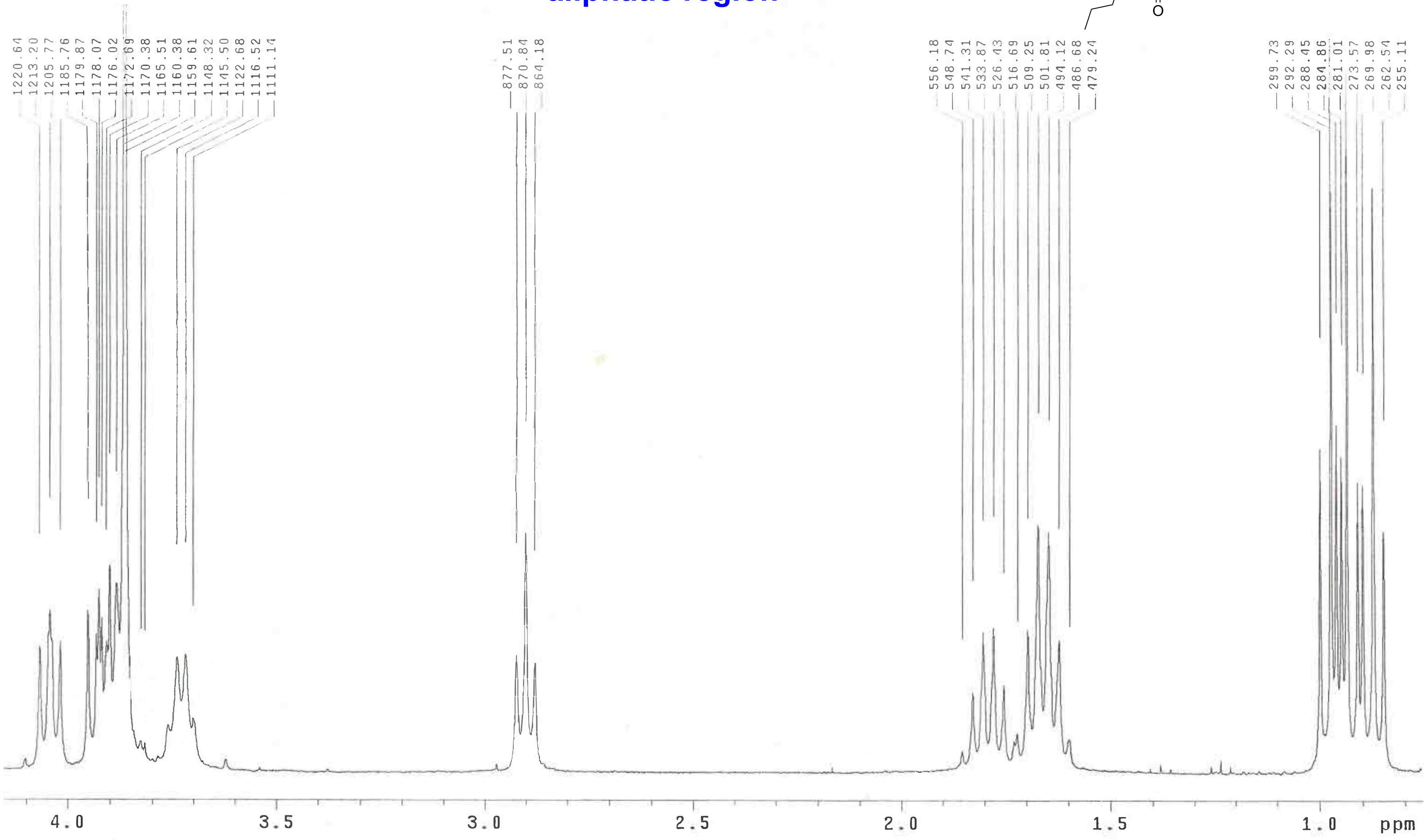

Table of peaks

INDEX	FREQUENCY	PPM	HEIGHT
1	2177.971	7.258	17.8
2	2050.771	6.834	9.6
3	2042.309	6.806	17.7
4	2024.357	6.746	6.0
5	2022.305	6.739	15.9
6	2021.023	6.735	18.8
7	2019.228	6.729	13.1
8	2015.894	6.718	7.8
9	2013.843	6.711	3.9
10	1274.497	4.247	2.8
11	1220.642	4.068	8.7
12	1213.205	4.043	11.2
13	1205.768	4.018	9.0
14	1185.765	3.952	11.1
15	1179.866	3.932	9.5
16	1178.071	3.926	12.6
17	1176.020	3.919	10.6
18	1172.686	3.908	9.0
19	1170.378	3.900	14.2
20	1165.505	3.884	13.0
21	1160.376	3.867	140.0
22	1159.607	3.864	144.0
23	1128.833	3.762	3.2
24	1122.678	3.741	7.9
25	1116.523	3.721	8.1
26	1111.138	3.703	3.7
27	877.512	2.924	8.1
28	870.844	2.902	16.5
29	864.176	2.880	7.5
30	548.742	1.829	5.5
31	541.305	1.804	9.7
32	533.868	1.779	10.0
33	526.431	1.754	6.1
34	516.686	1.722	2.7
35	509.249	1.697	9.9
36	501.812	1.672	17.1
37	494.119	1.647	16.7
38	486.681	1.622	9.1
39	299.729	0.999	22.4
40	292.292	0.974	50.9
41	288.445	0.961	24.1
42	284.855	0.949	21.9
43	281.008	0.936	48.5
44	273.571	0.912	20.1
45	269.981	0.900	19.9
46	262.544	0.875	40.4
47	255.107	0.850	16.7

Compound 9
^{13}C NMR

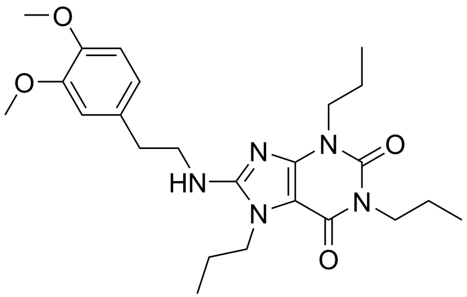

No.	(ppm)	(Hz)	Height
1	10.98	1105.2	0.2166
2	11.27	1133.9	0.2450
3	11.36	1143.4	0.2573
4	21.44	2158.0	0.4100
5	22.84	2298.8	0.1954
6	35.36	3558.4	0.1891
7	42.58	4284.6	0.1740
8	44.55	4483.4	0.1622
9	44.81	4509.1	0.2170
10	55.87	5622.7	0.2680
11	55.96	5631.5	0.2645
12	76.74	7722.3	1.0000
13	77.06	7754.6	0.9634
14	77.37	7786.1	0.9963
15	102.96	10361.0	0.0839
16	111.39	11209.1	0.1915
17	111.95	11265.5	0.1882
18	120.80	12156.1	0.2465
19	131.07	13189.8	0.1209
20	147.89	14882.9	0.1268
21	149.21	15015.7	0.1272
22	151.18	15213.8	0.1194
23	152.33	15329.7	0.0673
24	153.86	15483.7	0.1009

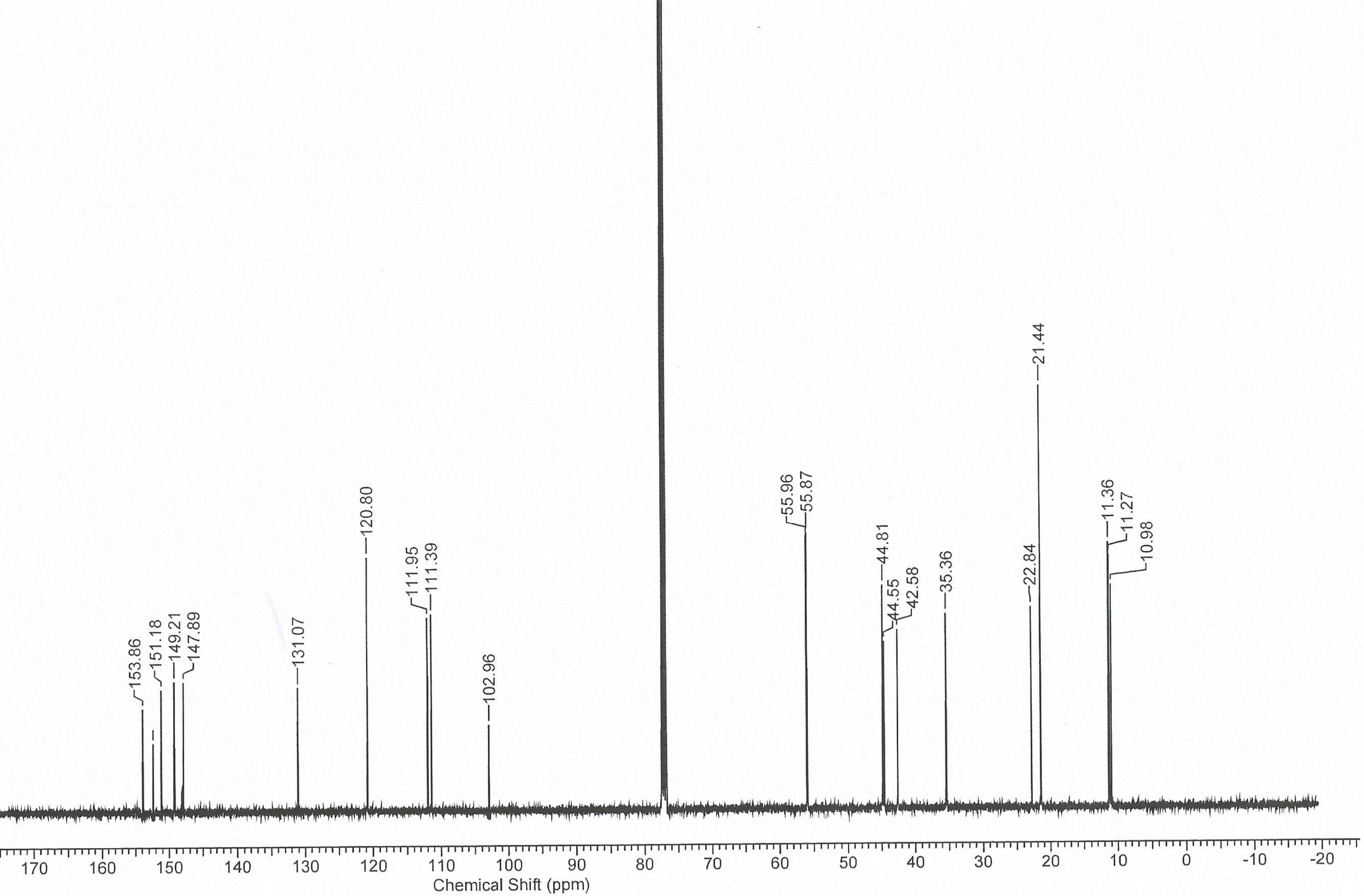

Compound 10
^{1}H NMR - zoom of the aromatic region

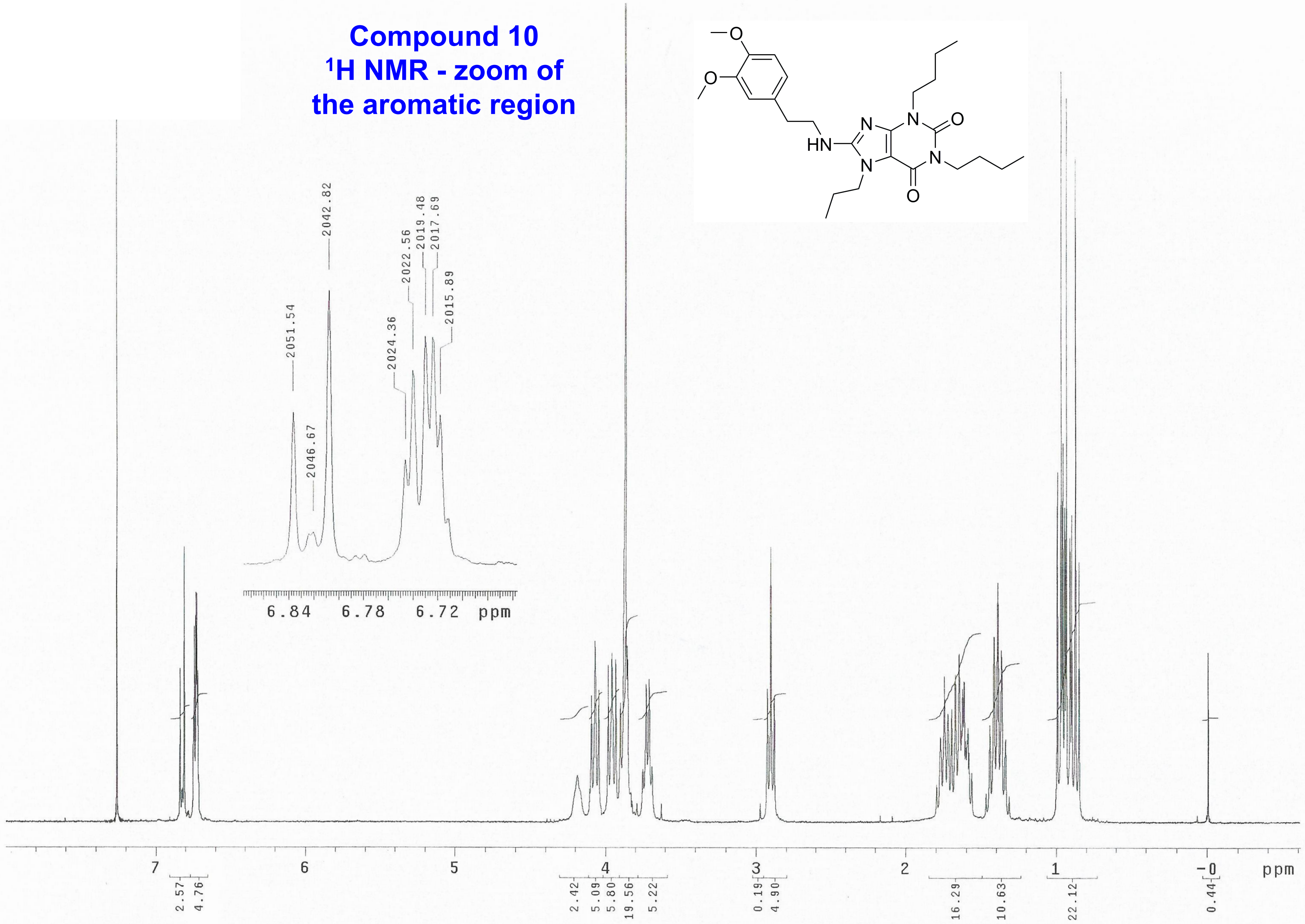

Compound 10
^{1}H NMR - zoom of the aliphatic region

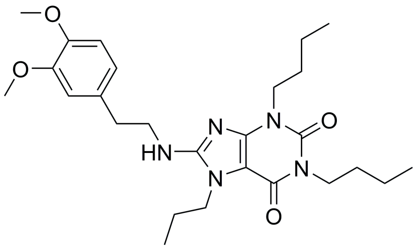

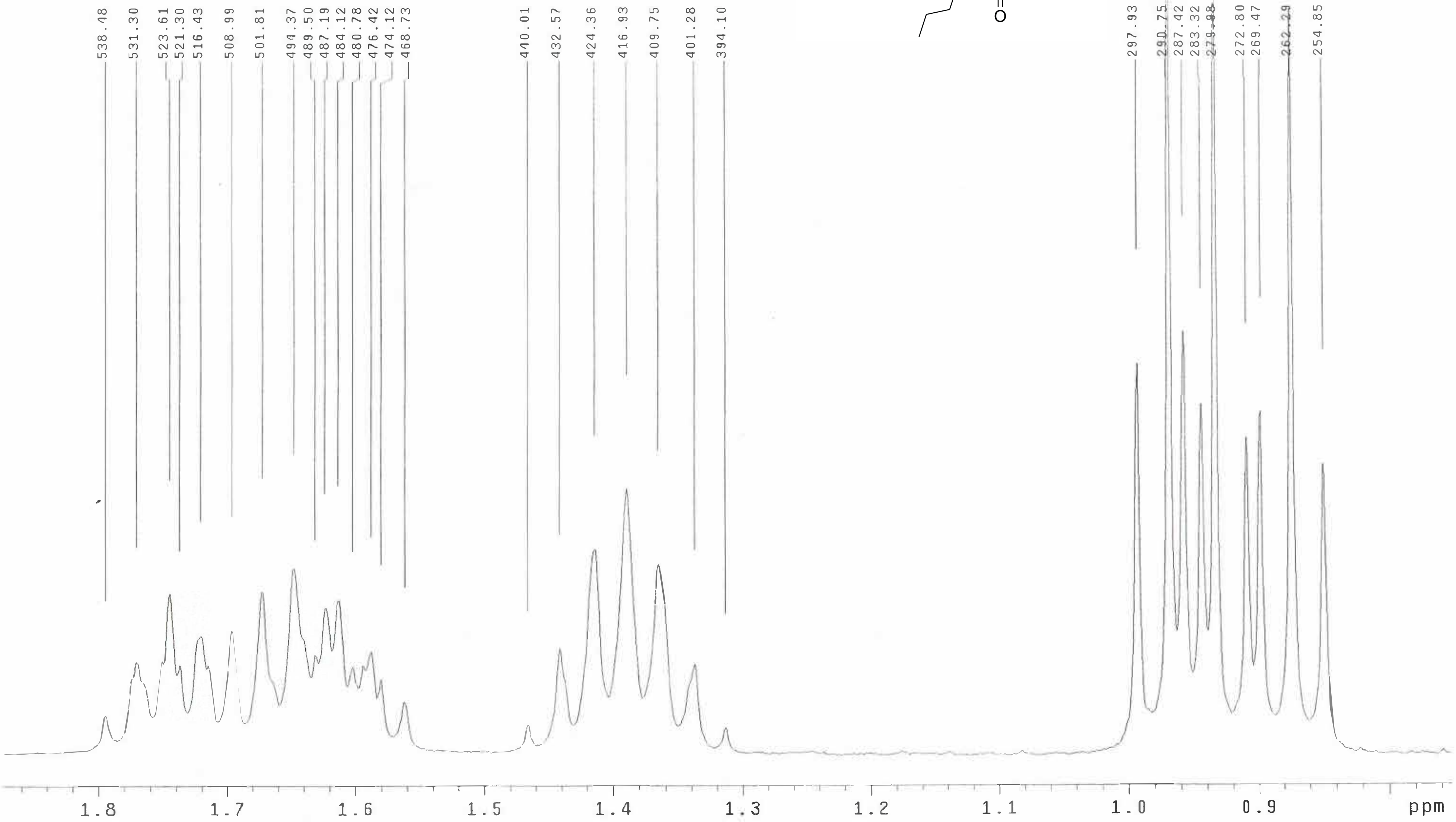

Compound 10
^{1}H NMR - zoom of the aliphatic region

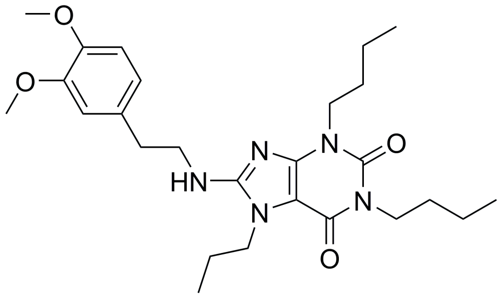

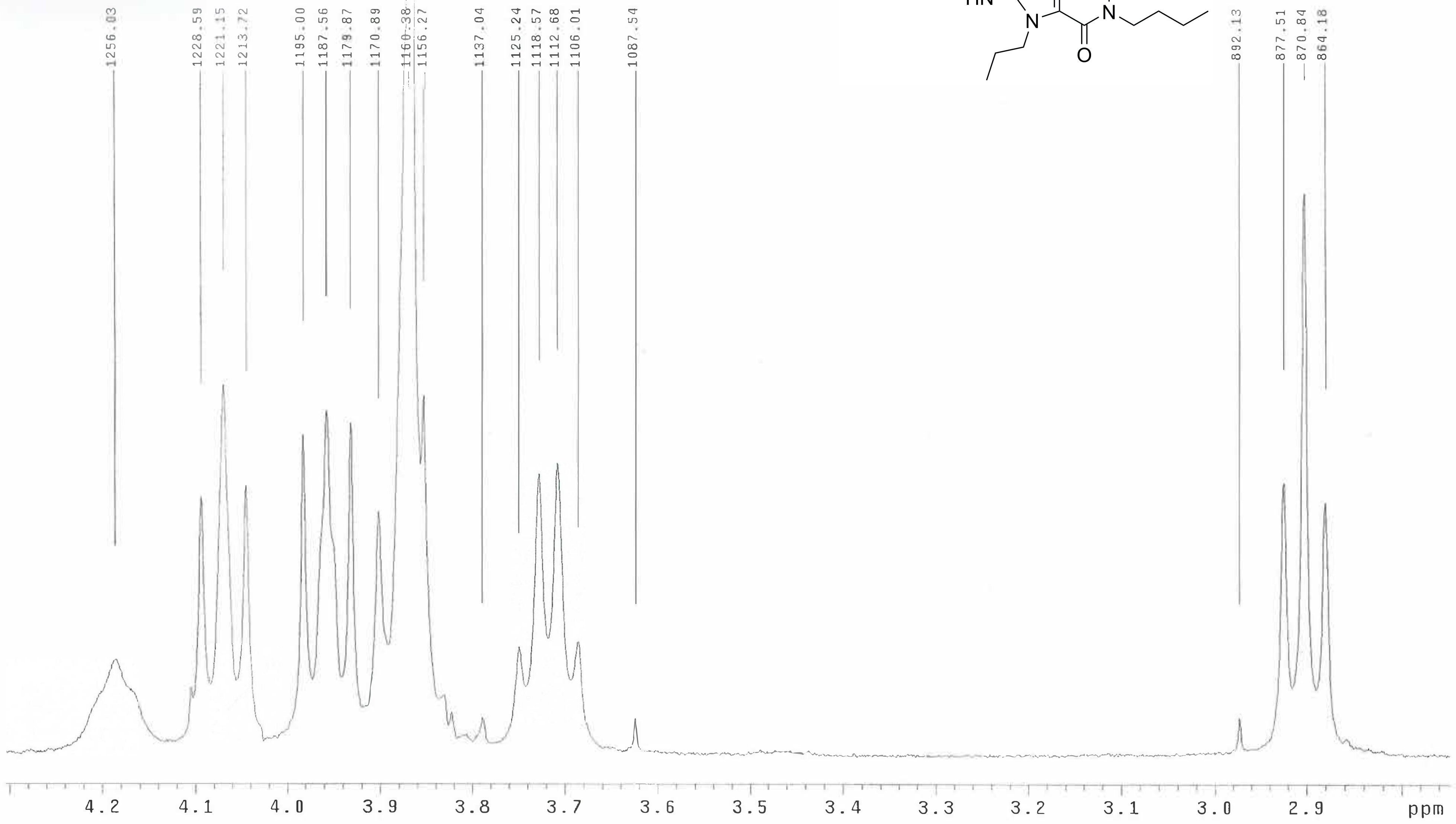

Table of peaks

INDEX	FREQUENCY	PPM	HEIGHT	INDEX	FREQUENCY	PPM	HEIGHT
1	2180.535	7.267	2.5	40	538.484	1.794	7.4
2	2180.022	7.265	3.4	41	531.304	1.771	16.6
3	2179.509	7.263	4.7	42	525.4[illegible]5	1.751	16.7
4	2178.997	7.261	8.0	43	523.610	1.745	28.4
5	2177.971	7.258	139.3	44	521.302	1.737	16.0
6	2176.689	7.254	5.6	45	516.430	1.721	21.1
7	2176.176	7.252	3.1	46	514.635	1.715	16.0
8	2051.541	6.837	29.9	47	508.993	1.696	22.1
9	2046.668	6.820	6.5	48	501.812	1.672	28.8
10	2042.821	6.808	53.6	49	494.375	1.647	32.9
11	2024.357	6.746	20.7	50	489.502	1.631	17.9
12	2022.562	6.740	38.1	51	487.194	1.624	26.0
13	2019.485	6.730	44.7	52	484.117	1.613	27.4
14	2017.689	6.724	44.5	53	480.783	1.602	15.8
15	2015.894	6.718	29.4	54	478.475	1.594	16.0
16	2013.843	6.711	9.2	55	476.423	1.588	18.4
17	1256.032	4.186	9.1	56	474.115	1.580	13.6
18	1231.669	4.104	6.5	57	468.730	1.562	9.6
19	1228.592	4.094	24.6	58	440.008	1.466	5.6
20	1221.155	4.069	35.3	59	432.570	1.442	18.9
21	1213.718	4.045	25.7	60	424.364	1.414	36.2
22	1194.997	3.982	30.5	61	416.927	1.389	46.7
23	1187.560	3.957	32.9	62	409.746	1.365	33.5
24	1179.866	3.932	31.7	63	401.284	1.337	16.2
25	1170.891	3.902	23.2	64	394.103	1.313	5.1
26	1160.376	3.867	682.1	65	297.934	0.993	68.3
27	1156.273	3.853	34.3	66	290.753	0.969	146.2
28	1149.349	3.830	5.8	67	287.420	0.958	74.0
29	1147.041	3.822	4.1	68	283.316	0.944	61.5
30	1137.039	3.789	3.6	69	279.983	0.933	141.0
31	1125.243	3.750	10.3	70	272.802	0.909	55.4
32	1118.575	3.728	26.8	71	269.468	0.898	60.0
33	1112.676	3.708	27.9	72	262.287	0.874	129.7
34	1106.009	3.686	10.8	73	254.850	0.849	50.8
35	1087.544	3.624	3.6	74	-2.369	-0.008	33.0
36	892.129	2.973	3.7				
37	877.512	2.924	26.0				
38	870.844	2.902	53.7				
39	864.176	2.880	24.2				

Compound 10
^{13}C NMR

No.	(ppm)	(Hz)	Height
1	10.99	1106.0	0.3240
2	13.82	1390.6	0.3496
3	13.85	1393.5	0.4031
4	20.01	2013.4	0.3322
5	20.33	2045.7	0.3007
6	22.85	2299.5	0.2810
7	30.21	3040.5	0.2939
8	30.31	3050.0	0.3083
9	35.36	3558.4	0.2392
10	40.94	4119.6	0.2108
11	43.05	4332.3	0.2029
12	44.54	4482.0	0.2417
13	44.77	4505.5	0.2001
14	55.86	5621.3	0.3328
15	55.96	5631.5	0.3430
16	76.74	7722.3	0.9906
17	77.05	7753.8	0.9913
18	77.37	7786.1	1.0000
19	103.00	10365.4	0.1196
20	111.39	11209.8	0.2564
21	111.93	11263.3	0.2450
22	120.79	12155.4	0.2770
23	131.08	13191.2	0.1820
24	147.89	14882.9	0.1445
25	148.20	14913.7	0.0471
26	149.21	15015.7	0.1473
27	151.16	15211.6	0.1359
28	152.38	15334.1	0.1266
29	153.85	15482.2	0.1282

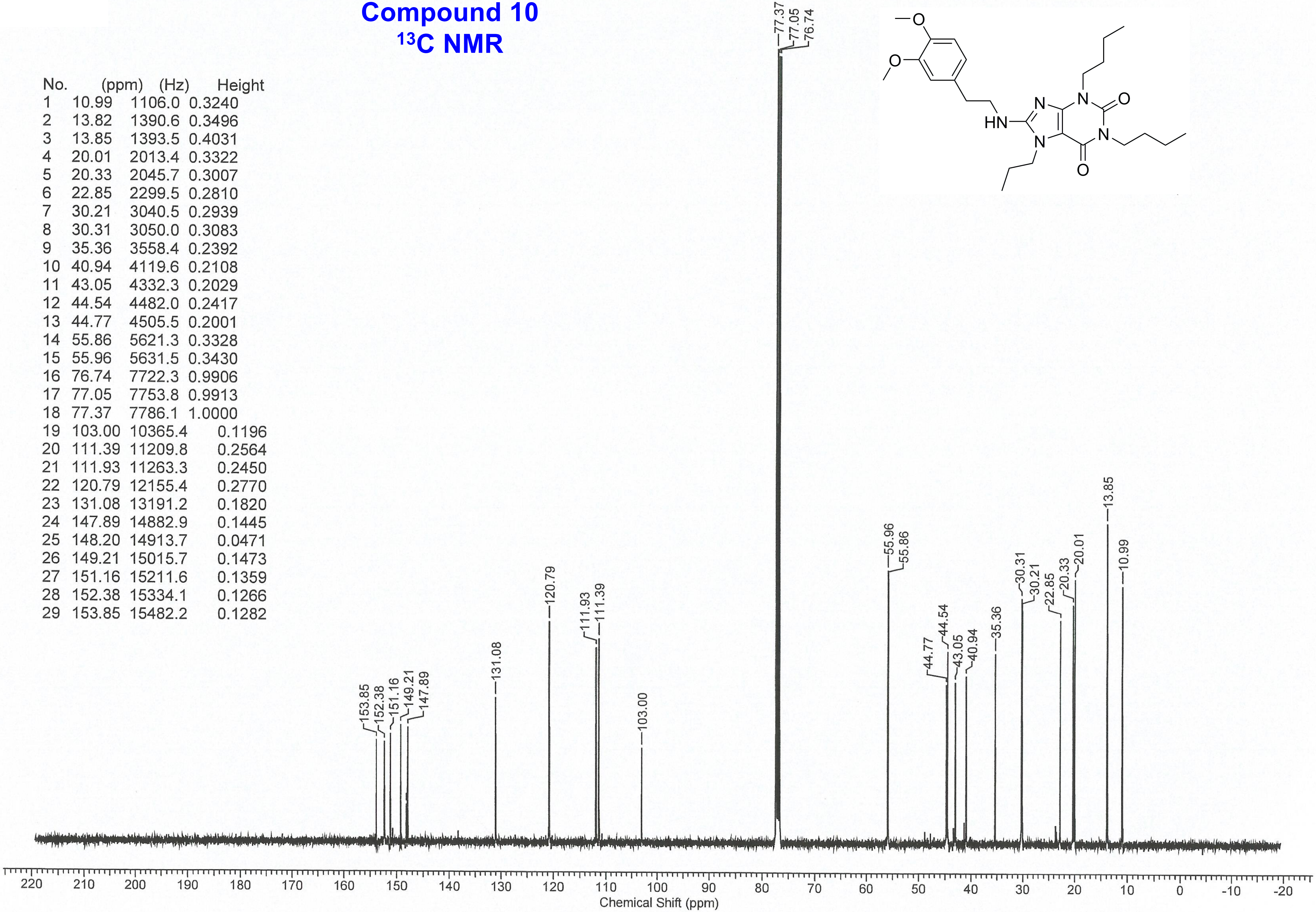

Compound 17b
^{1}H NMR - zoom of the aromatic region

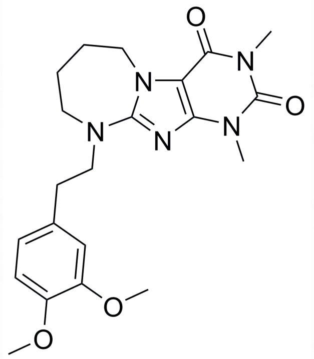

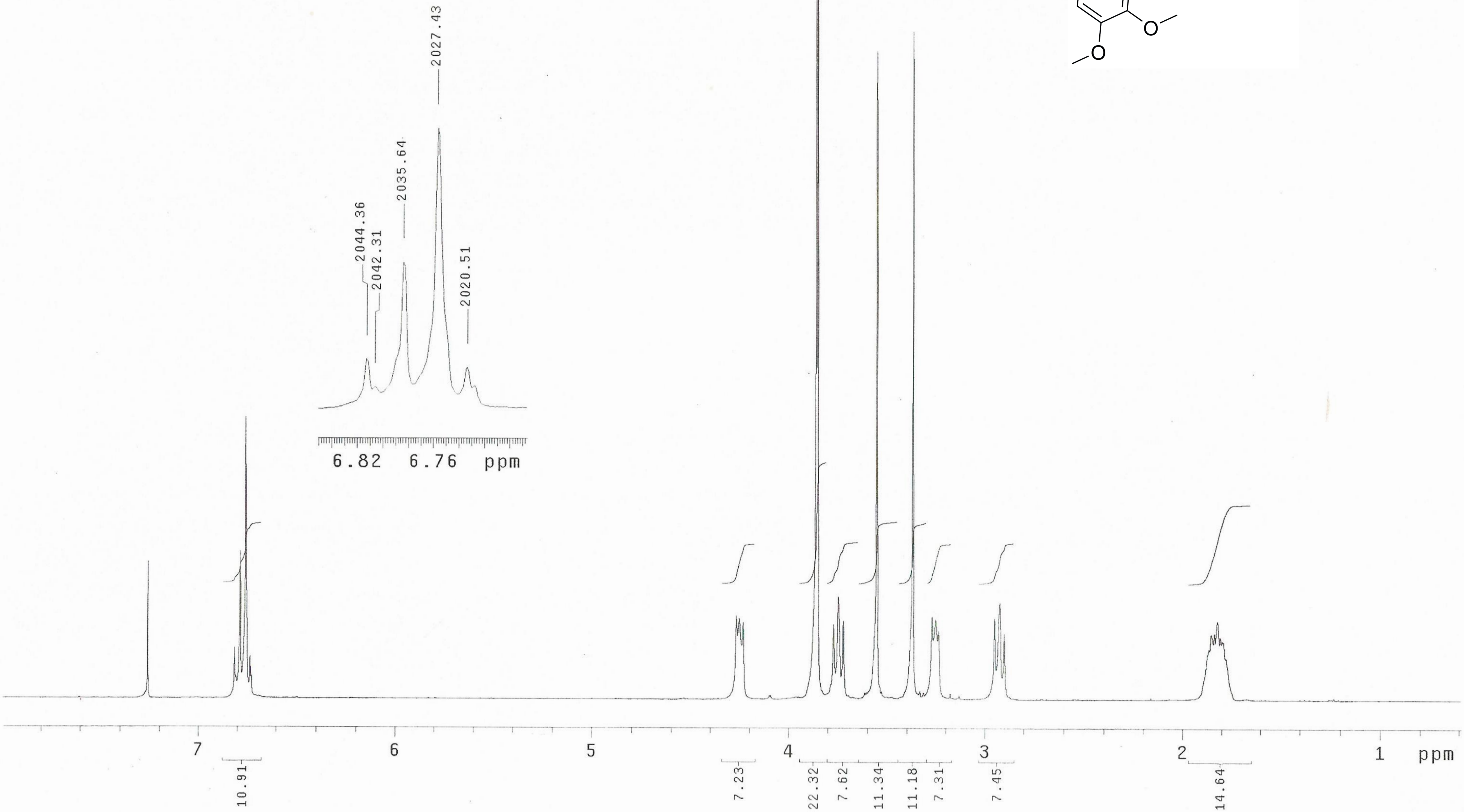

Compound 17b
^{1}H NMR - zoom of the aliphatic region

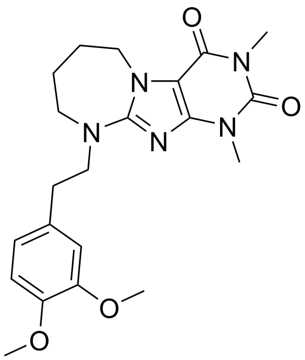

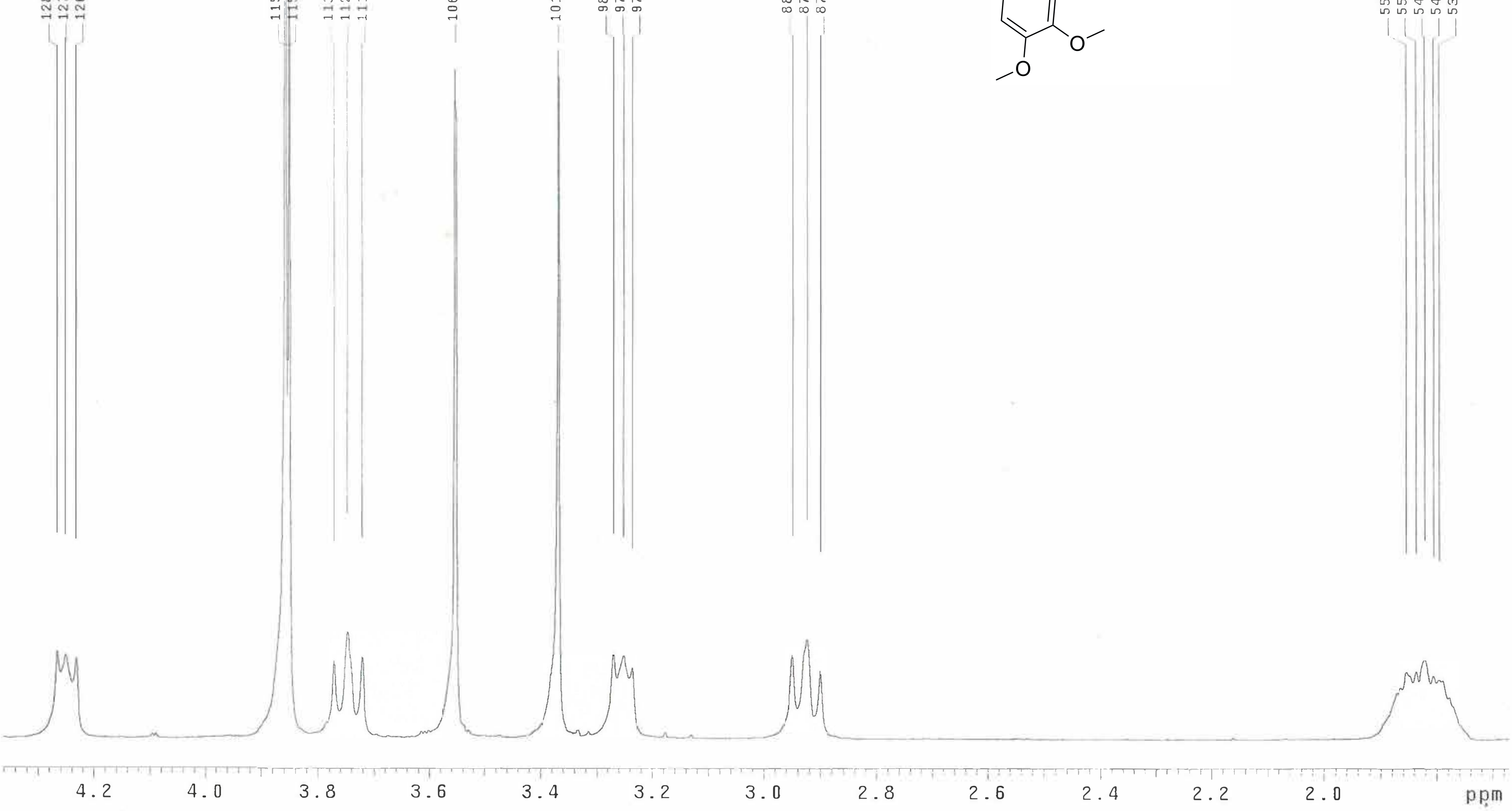

Table of peaks

INDEX	FREQUENCY	PPM	HEIGHT
1	2177.971	7.258	23.5
2	2044.360	6.813	8.7
3	2042.309	6.806	3.9
4	2035.641	6.784	25.4
5	2027.434	6.756	48.6
6	2020.510	6.733	7.2
7	2018.715	6.727	4.0
8	1280.138	4.266	14.4
9	1275.522	4.251	13.9
10	1269.880	4.232	13.3
11	1157.555	3.858	144.0
12	1155.504	3.851	136.7
13	1131.654	3.771	13.0
14	1124.730	3.748	17.8
15	1116.780	3.722	13.5
16	1066.259	3.553	111.9
17	1010.866	3.369	115.3
18	981.118	3.270	14.3
19	975.732	3.252	13.8
20	971.116	3.236	11.9
21	884.949	2.949	14.0
22	877.255	2.923	16.7
23	870.075	2.899	11.3
24	559.257	1.864	8.7
25	556.179	1.853	11.3
26	551.050	1.836	11.4
27	546.434	1.821	13.5
28	541.818	1.806	10.7
29	538.741	1.795	10.1
30	536.946	1.789	10.0
31	533.355	1.777	7.2

Compound 17b
^{13}C NMR

No.	(ppm)	(Hz)	Height
1	26.35	2651.7	0.2729
2	27.70	2787.4	0.2879
3	28.99	2917.2	0.2495
4	29.69	2987.6	0.2747
5	33.89	3410.2	0.2470
6	45.84	4612.6	0.2132
7	52.32	5265.5	0.2289
8	55.19	5553.8	0.2580
9	55.86	5621.3	0.2970
10	55.92	5627.9	0.2851
11	76.74	7722.3	0.9482
12	77.05	7753.8	1.0000
13	77.37	7786.1	0.9748
14	104.12	10477.7	0.1115
15	111.27	11197.3	0.2228
16	112.02	11272.9	0.2285
17	120.79	12155.4	0.3095
18	131.75	13258.0	0.2146
19	147.62	14855.0	0.1311
20	148.02	14895.4	0.0871
21	148.93	14987.1	0.1410
22	151.79	15275.4	0.1210
23	154.40	15538.0	0.1132
24	158.65	15965.0	0.1345

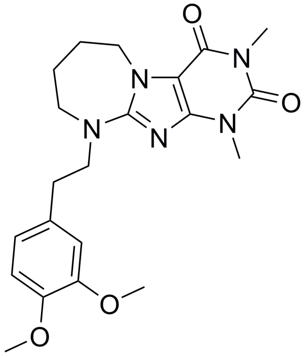

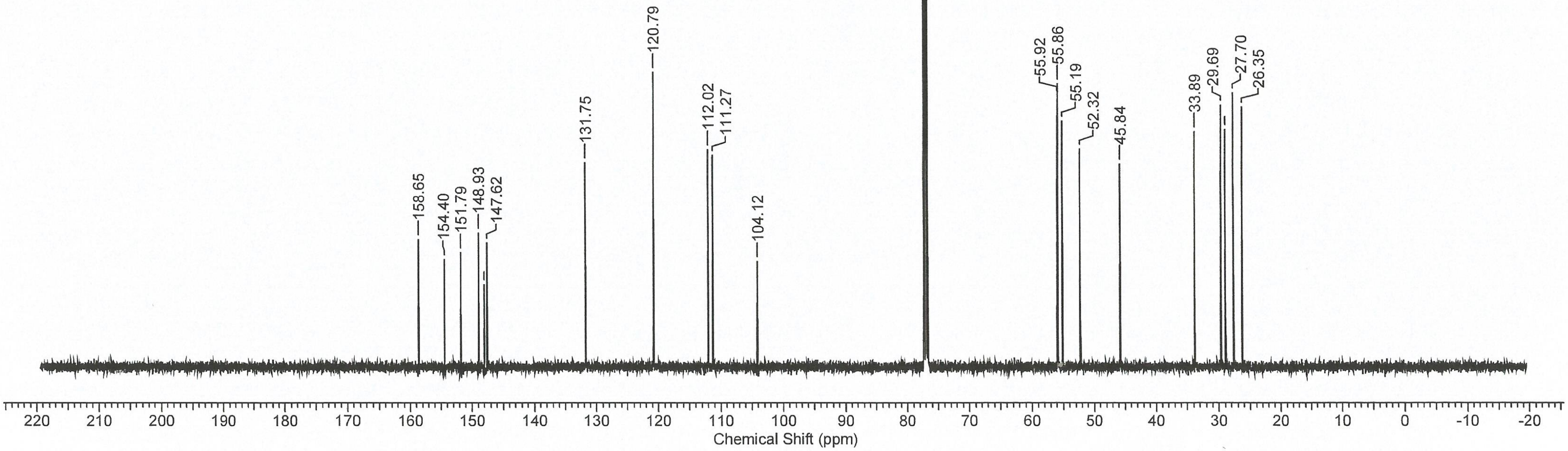

Compound 19
^{1}H NMR

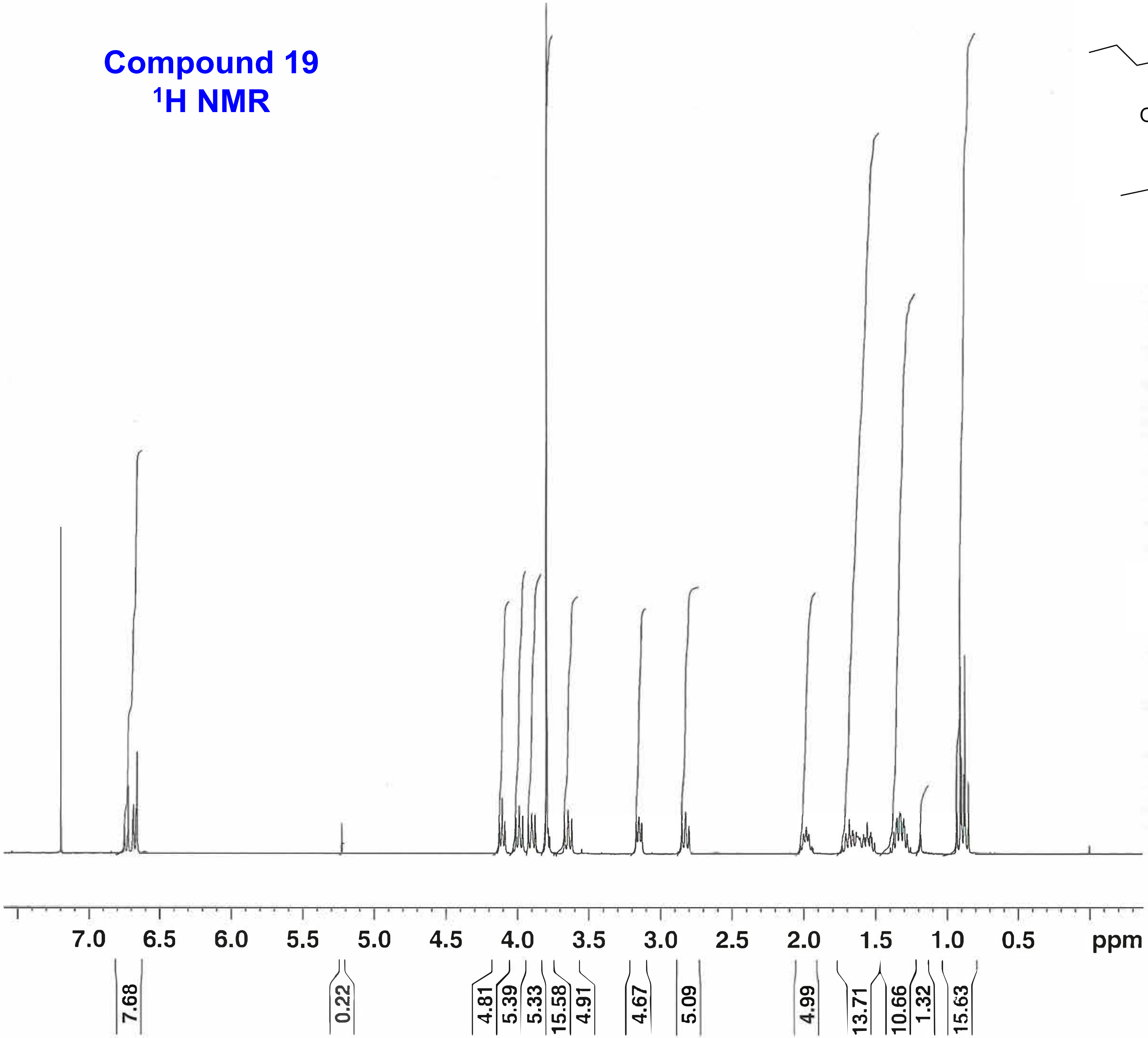

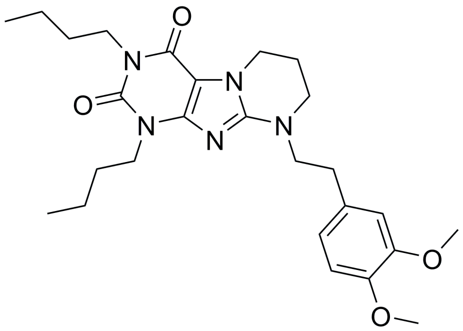

Compound 19
^{1}H NMR - zoom of the aliphatic region

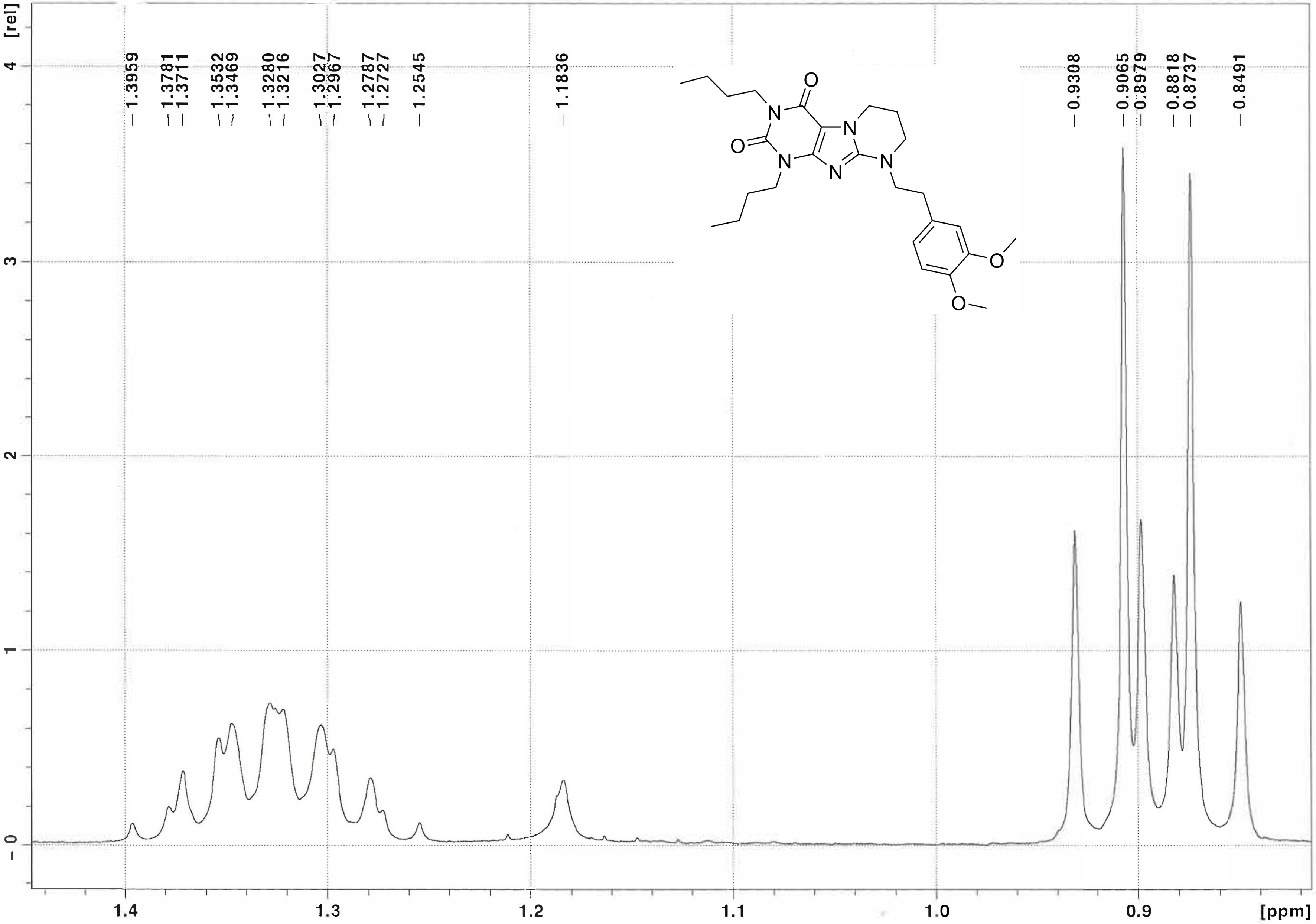

Compound 19
^{1}H NMR - zoom of the aliphatic region

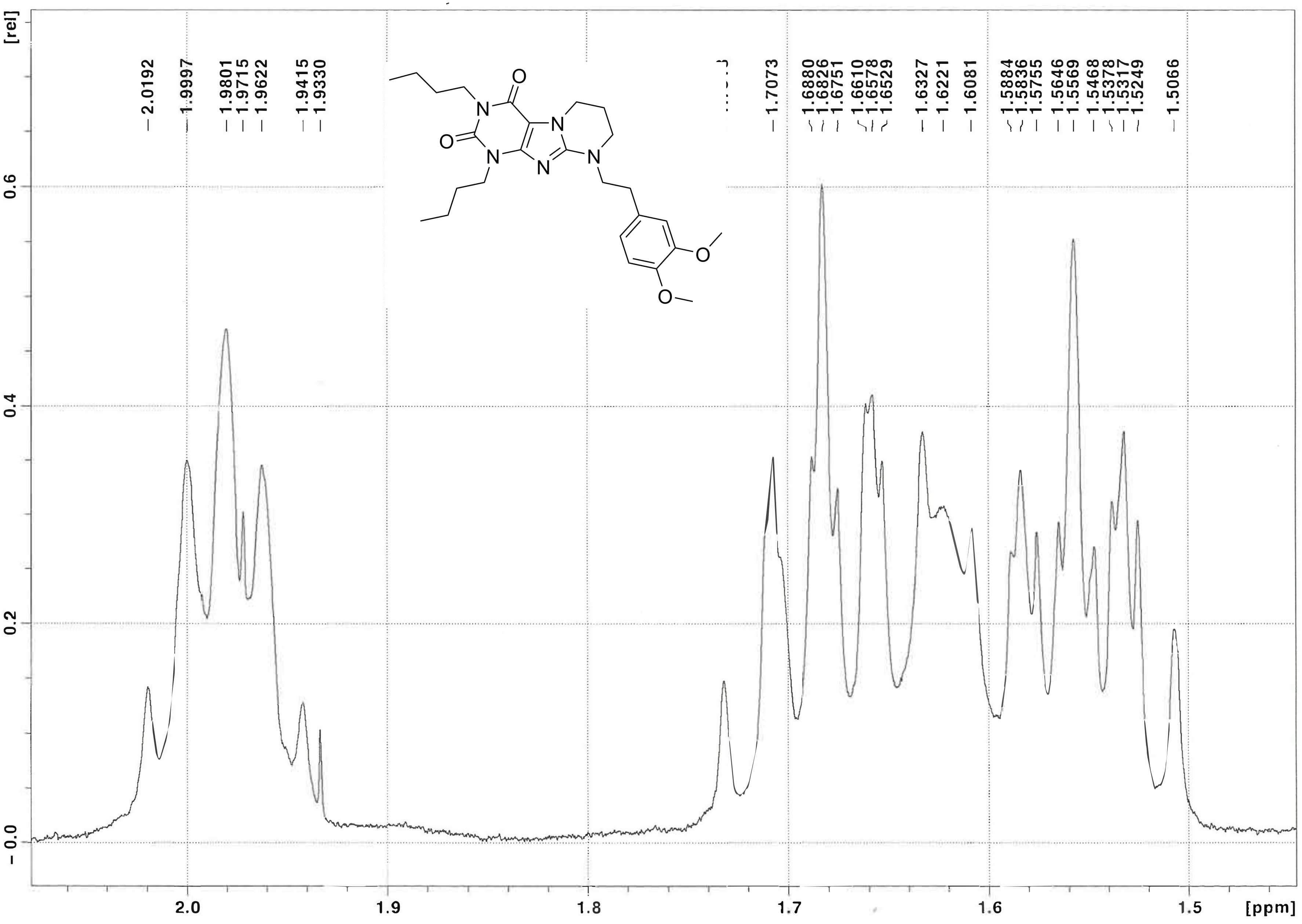

Compound 19
^{1}H NMR - zoom of the aliphatic region

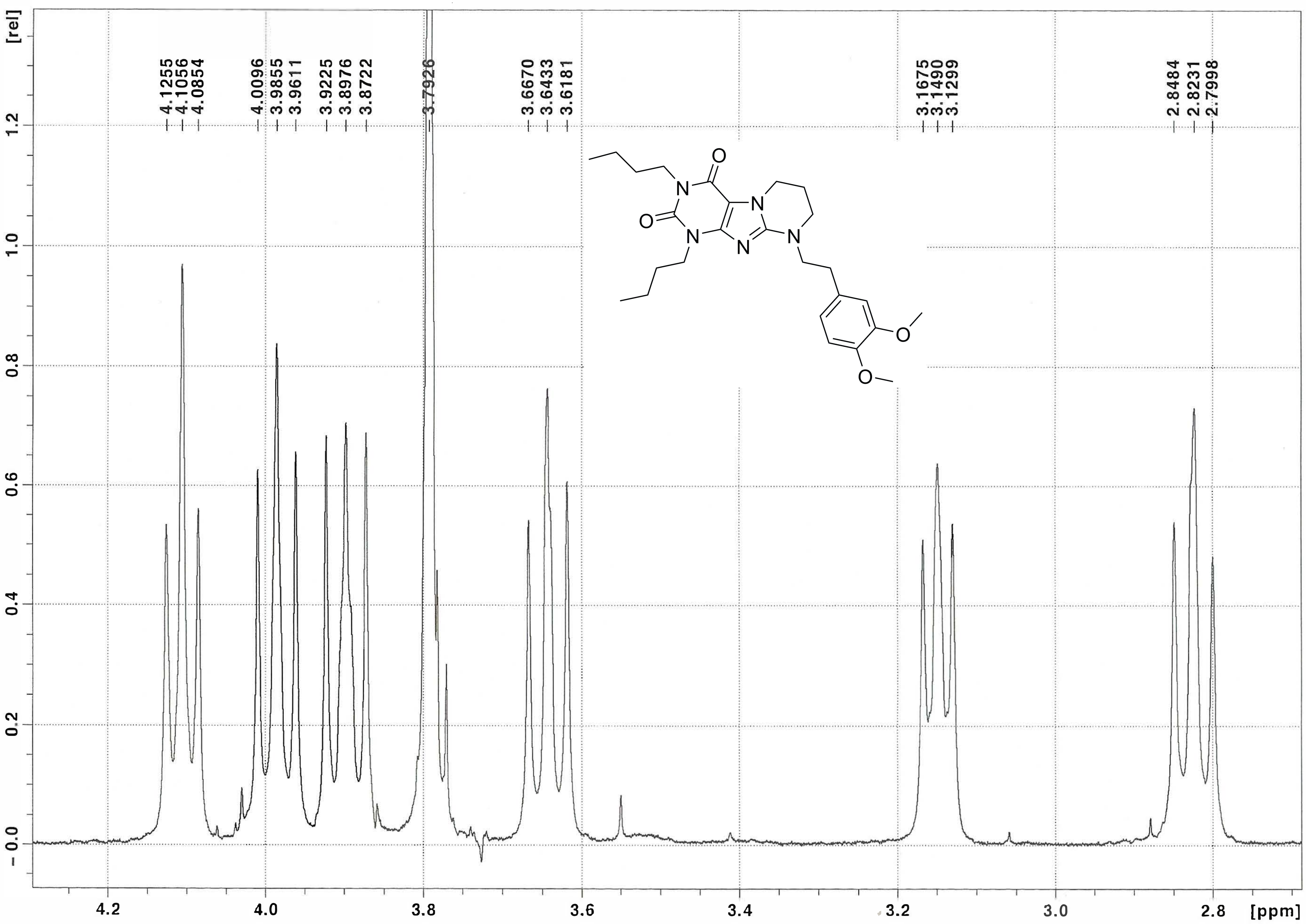

Compound 19
^{1}H NMR - zoom of the aromatic region

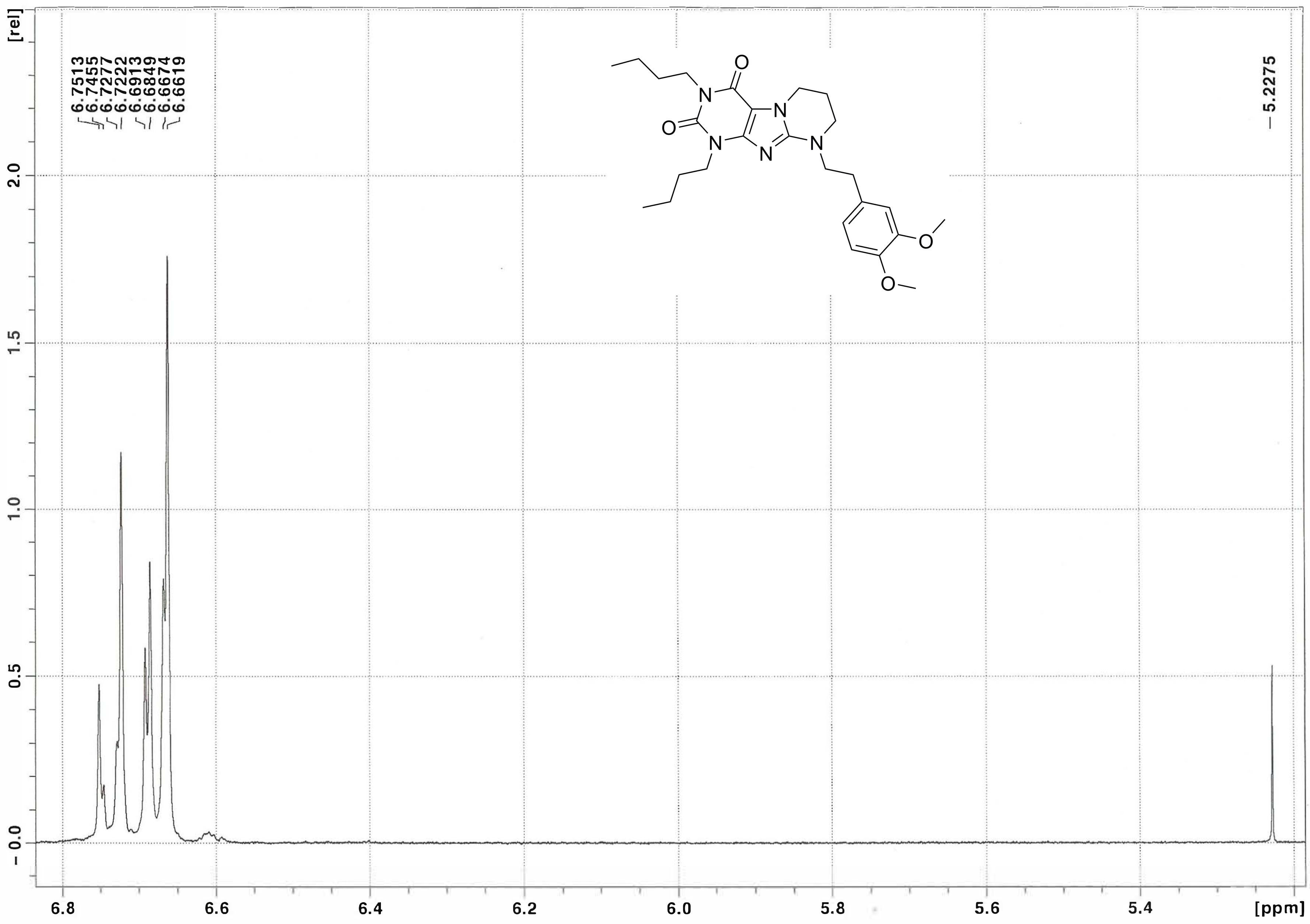

Table of peaks

Peak	ν(F1) [ppm]	ν(F1) [Hz]	Intensity
1	7.1972	2160.0958	5.74
2	6.7513	2026.2678	0.47
3	6.7455	2024.5271	0.17
4	6.7277	2019.1848	0.30
5	6.7222	2017.5341	1.18
6	6.6913	2008.2600	0.58
7	6.6849	2006.3392	0.84
8	6.6674	2001.0869	0.79
9	6.6619	1999.4362	1.76
10	5.2275	1568.9297	0.53
11	4.1255	1238.1864	0.53
12	4.1056	1232.2138	0.97
13	4.0854	1226.1512	0.56
14	4.0096	1203.4013	0.63
15	3.9855	1196.1682	0.84
16	3.9611	1188.8450	0.66
17	3.9225	1177.2600	0.69
18	3.8976	1169.7868	0.71
19	3.8722	1162.1635	0.69
20	3.7926	1138.2731	15.00
21	3.6670	1100.5768	0.54
22	3.6433	1093.4637	0.76
23	3.6181	1085.9004	0.61
24	3.1675	950.6619	0.51
25	3.1490	945.1094	0.64
26	3.1299	939.3770	0.54
27	2.8484	854.8904	0.54
28	2.8231	847.2971	0.73
29	2.7998	840.3040	0.48
30	2.0192	606.0225	0.14
31	1.9997	600.1700	0.35
32	1.9801	594.2875	0.47
33	1.9715	591.7063	0.30
34	1.9622	588.9151	0.35
35	1.9415	582.7024	0.13
36	1.9330	580.1513	0.10
37	1.7318	519.7652	0.15
38	1.7073	512.4120	0.35
39	1.6880	506.6195	0.35
40	1.6826	504.9988	0.60
41	1.6751	502.7478	0.32
42	1.6610	498.5160	0.40
43	1.6578	497.5556	0.41
44	1.6529	496.0849	0.35
45	1.6327	490.0223	0.38

Table of peaks

Peak	ν(F1) [ppm]	ν(F1) [Hz]	Intensity
46	1.6221	486.8409	0.31
47	1.6081	482.6391	0.29
48	1.5884	476.7265	0.27
49	1.5836	475.2859	0.34
50	1.5755	472.8549	0.28
51	1.5646	469.5834	0.29
52	1.5569	467.2724	0.55
53	1.5468	464.2411	0.27
54	1.5378	461.5400	0.31
55	1.5317	459.7092	0.38
56	1.5249	457.6683	0.29
57	1.5066	452.1759	0.20
58	1.3959	418.9515	0.11
59	1.3781	413.6092	0.20
60	1.3711	411.5083	0.38
61	1.3532	406.1360	0.55
62	1.3469	404.2451	0.63
63	1.3280	398.5727	0.73
64	1.3216	396.6518	0.70
65	1.3027	390.9794	0.62
66	1.2967	389.1786	0.49
67	1.2787	383.7763	0.35
68	1.2727	381.9755	0.18
69	1.2545	376.5131	0.11
70	1.1836	355.2339	0.34
71	0.9308	279.3610	1.62
72	0.9065	272.0679	3.59
73	0.8979	269.4867	1.68
74	0.8818	264.6547	1.39
75	0.8737	262.2236	3.46
76	0.8491	254.8404	1.25

Compound 19
^{13}C NMR

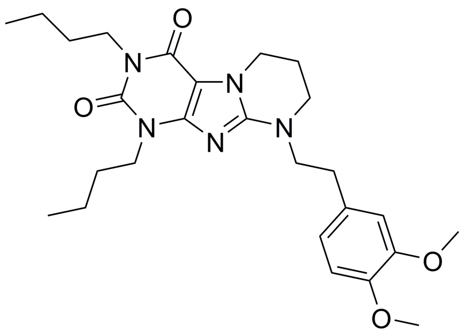

No.	(ppm)	(Hz)	Height
1	13.83	1391.3	0.1898
2	13.86	1395.0	0.2098
3	20.01	2013.4	0.2019
4	20.31	2044.2	0.1925
5	21.48	2161.6	0.1219
6	30.18	3036.8	0.1621
7	30.38	3057.3	0.1648
8	33.52	3372.8	0.1310
9	40.78	4104.2	0.1254
10	41.52	4178.3	0.1198
11	42.91	4318.4	0.1134
12	45.37	4565.6	0.1200
13	51.65	5198.0	0.1185
14	55.84	5619.8	0.1549
15	55.92	5627.9	0.1837
16	76.72	7720.8	0.9885
17	77.04	7752.4	0.9837
18	77.36	7784.6	1.0000
19	102.89	10353.7	0.0708
20	111.33	11203.2	0.1288
21	111.98	11268.5	0.1298
22	120.80	12156.1	0.1613
23	131.49	13232.3	0.1185
24	147.70	14863.8	0.0797
25	148.95	14989.3	0.0795
26	148.98	14992.2	0.0928
27	151.29	15224.8	0.0908
28	151.37	15232.8	0.0883
29	153.84	15481.5	0.0889

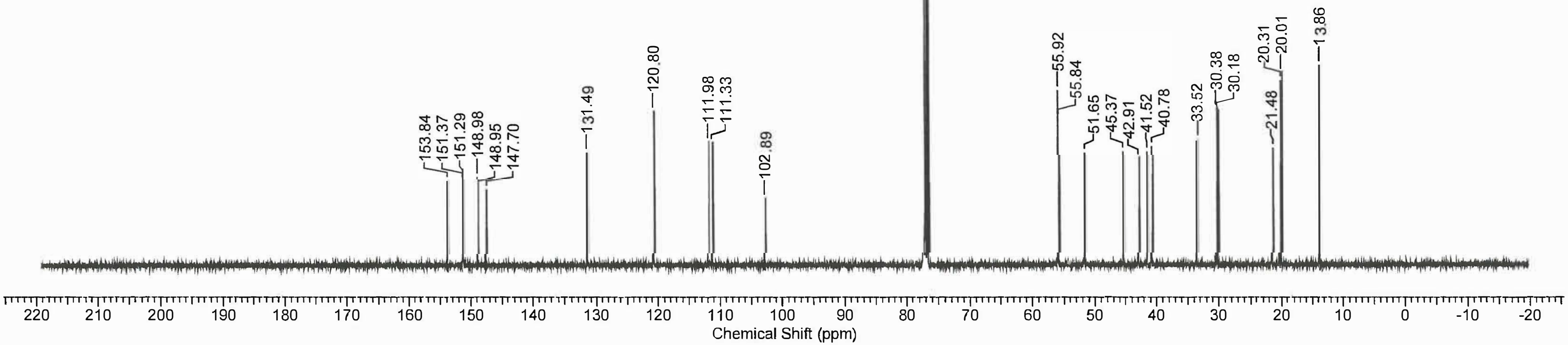

Supplement: Supplementary file 1 [file biomolecules-13-01079-s001.zip › biomolecules-2428451-supplementary.pdf]
